# Mutations and Protein Interaction Landscape Reveal Key Cellular Events Perturbed in Upper Motor Neurons with HSP and PLS

**DOI:** 10.3390/brainsci11050578

**Published:** 2021-04-29

**Authors:** Oge Gozutok, Benjamin Ryan Helmold, P. Hande Ozdinler

**Affiliations:** 1 Department of Neurology, Feinberg School of Medicine, Northwestern University, 303 E. Chicago Ave, Chicago, IL 60611, USA; oge.gozutok@northwestern.edu (O.G.); benhelmold2023@u.northwestern.edu (B.R.H.); 2 Center for Molecular Innovation and Drug Discovery, Center for Developmental Therapeutics, Chemistry of Life Processes Institute, Northwestern University, Evanston, IL 60611, USA; 3 Mesulam Center for Cognitive Neurology and Alzheimer’s Disease, Feinberg School of Medicine, Northwestern University, Chicago, IL 60611, USA; 4 Feinberg School of Medicine, Les Turner ALS Center at Northwestern University, Chicago, IL 60611, USA

**Keywords:** upper motor neurons, protein landscape, interaction domain, upstream regulator, lipid homeostasis, growth factors

## Abstract

Hereditary spastic paraplegia (HSP) and primary lateral sclerosis (PLS) are rare motor neuron diseases, which affect mostly the upper motor neurons (UMNs) in patients. The UMNs display early vulnerability and progressive degeneration, while other cortical neurons mostly remain functional. Identification of numerous mutations either directly linked or associated with HSP and PLS begins to reveal the genetic component of UMN diseases. Since each of these mutations are identified on genes that code for a protein, and because cellular functions mostly depend on protein-protein interactions, we hypothesized that the mutations detected in patients and the alterations in protein interaction domains would hold the key to unravel the underlying causes of their vulnerability. In an effort to bring a mechanistic insight, we utilized computational analyses to identify interaction partners of proteins and developed the protein-protein interaction landscape with respect to HSP and PLS. Protein-protein interaction domains, upstream regulators and canonical pathways begin to highlight key cellular events. Here we report that proteins involved in maintaining lipid homeostasis and cytoarchitectural dynamics and their interactions are of great importance for UMN health and stability. Their perturbation may result in neuronal vulnerability, and thus maintaining their balance could offer therapeutic interventions.

## 1. Introduction

Upper motor neurons (UMNs) are an important component of the motor neuron circuitry [1,2,3,4,5,6]. Their degeneration leads to hereditary spastic paraplegia (HSP) [7,8] and primary lateral sclerosis (PLS) [9,10,11], two rare motor neuron diseases identified by selective and progressive UMN loss in patients [12]. Since movement starts in the brain, the UMNs have the unique ability to convey the cerebral cortex’s input to spinal cord targets such that voluntary movement can be initiated and modulated [1,13]. Their degeneration severs the contact between the brain and spinal cord and leads to paralysis in patients. UMNs are one of the largest neurons in the brain and one of the most polarized: apical dendrites extend to the top layers of the brain and the axons project to the sacral regions of the spinal cord. For this amazingly elaborate neuron to be healthy and functional, numerous cellular events and canonical pathways must be active.

Almost all cellular events that take place inside the neurons require protein-protein interactions. Each protein binds to a distinct set of proteins and require interaction partners to perform its function. When genes that code for proteins are mutated, their protein products also become mutated. Depending on where the mutation is, the protein may lose some or all of its function. The mutation may not allow them to interact with their usual interaction partners, and thus fail to contribute to their normal cellular events. Not all proteins are equally essential for a given cellular event. Some protein’s activity may be compensated by others. Therefore, mutations in the genes that code for those proteins may not have an imminent impact on neurons. However, when a protein is so critical for the proper function of a given cellular event, even a slight reduction in its function, or a change in its ability to interact with other proteins would lead to debilitating and severe consequences for the neuron, leading to their vulnerability and progressive degeneration. Interestingly, most of the mutated genes identified in patients code for these significant players, with irreplaceable functions. In an effort to reveal the key cellular events that are essential for the health and function of UMNs, we must reveal the identity of key players and the cellular events they belong.

Neurons become vulnerable to degeneration when they fail to perform cellular events that are required for their proper function [14]. For example, a dopaminergic neuron is characterized by its ability to generate and secrete dopamine. Not all neurons are capable of doing this. Thus, when there is a mutation in a gene that codes for a protein that is critically important for the generation and secretion of dopamine, only the dopaminergic neurons will feel the burden, while other cells and neurons would continue to function normally. Likewise, when there is any alteration to the key cellular events that are required for the health and function of UMNs, then the UMNs will begin to display selective vulnerability, whereas other cells and neurons will remain active and healthy. Therefore, mutations detected in patients hold great potential to reveal the cellular events that are particularly important for UMNs, the canonical pathways that are primarily active, the upstream regulators, downstream effectors, and the interactome domains which are involved in cellular functions. Identification of this information will not only reveal how UMNs work, but why they become vulnerable to degeneration.

Here, we investigated the protein products of mutated genes detected in HSP and PLS patients and their interactions. The proteins are determined by a stringent inclusion criteria and IPA (ingenuity pathway analyses), a large-data management tool box is utilized to study the presence of canonical pathways they are involved in, as well as upstream regulators and key cellular events that are highlighted by the presence of these proteins. Our unbiased computational protein-protein interaction studies revealed the presence of a signature of cellular events that are particularly important for the health and function of UMNs, suggesting that when these are perturbed UMNs may become vulnerable to degeneration. We also uncovered the list of growth factors UMNs most respond to and potentially are important for their improved health.

## 2. Materials and Methods

Previously published results and open public resources and databases, such as OMIM and Pubmed were used to compile the mutations detected in HSP and PLS patients (end date March 2021). For determining the list of proteins that bind to the protein product of the mutated genes, large data management tool box ingenuity pathway analyses (IPA; QIAGEN Comp, LA, USA) was used as previously reported [15]. IPA enables analysis, integration, interpretation and understanding of large data from gene expression, miRNA, SNP microarrays, as well as metabolomics, proteomics, and RNAseq experiments. Previously published knowledge serves as the domain for the data platform. In this analysis, the binding partners for the protein products of each mutated gene related to HSP and PLS were determined by a stringent inclusion criterion. The protein had to be a direct binding partner, determined either by yeast-two-hybrid or co-immunoprecipitation experiments, or other protein assays that reveal direct protein-protein interactions. Only protein-protein interactions with experimental findings published in peer-reviewed journals were included. Results collected from non-cell environments, ex vivo chemical reactions, and those that used uncharacterized cells or sources were excluded. Only results from mammalian systems and with confirmed protein-protein binding assays were included. For each protein included in this study, there was at least one publication to confirm direct binding and interaction. To increase stringency and to eliminate false-positive results, proteins with more than 3 binding partners were selected, proteins with two or one binding partners were eliminated. Circos was used to generate circular representation of integrative data [16]. (http://circos.ca/ accessed on 27 April 2021)

### Statistical Analysis

Ingenuity Pathway Analysis (IPA) uses an array of statistical analyses to determine whether the analyzed data set has significant coverage with any of the previously determined canonical pathways, cellular events, protein-protein interaction domains, and pathways. (https://www.qiagenbioinformatics.com/products/ingenuity-pathway-analysis accessed on 27 April 2021). Statistical analyses use Fisher’s exact test. In summary, the significance value associated with functional analysis of a dataset is a measure of the likelihood that the association between the experimental group (i.e., HSP proteins) and the given pathway is due to random chance or not. For the IPA analysis, the ratio is calculated by taking the number of genes from the HSP-PLS protein list that participate in a Canonical Pathway and dividing it by the total number of genes in that Canonical Pathway. The ratio is therefore useful for determining which pathways overlap the most with the HSP-PLS protein list. The *p*-value measures how likely the observed association between a specific pathway and the dataset would be if it was only due to random chance. *p* < 0.05 or (−log *p*-value = 1.3) is considered significant and that the ratio of proteins in that canonical pathway cannot be explained by randomness. The *p*-value is calculated by considering: (1) the number of functions/pathways that participate in the cellular event; (2) the total number of molecules in the HSP-PLS protein list known to be associated with that pathway; (3) the total number of molecules in the selected reference set. The *p*-value calculation depends on the statistical null model, such as the “random” model. Fisher’s exact test is used to determine the likelihood of randomness. The activation z-score predicts the activation state of the upstream regulator, using the expression patterns of the genes/proteins that are downstream of an upstream regulator. The z-score calculation needs a minimum of 4 targets with an expected expression pattern. An absolute z-score of ≥ 2 is considered significant. An upstream regulator is “Activated” if the z-score is ≥ 2 and “Inhibited” if the z-score < 2.

## 3. Results

As the genetic component of HSP and PLS is better understood, many genes are identified to be either causative or associated with the diseases. Currently, 58 genes are reported to cause HSP, and 1 gene cause PLS when mutated, whereas mutations in 34 different genes are associated with HSP and 7 genes are associated or linked with PLS. (Table 1). Mutations in these genes have been well-reported and documented in HSP and PLS patients (please refer to Supplementary Table S1 for references cited for each mutation), and some animal models have already been generated to bring a mechanistic insight for the underlying causes of UMN degeneration [17,18,19,20,21].

In an effort to understand the dynamics of protein-protein interaction domains and how they are perturbed with respect to UMN diseases, we investigated the binding partners of proteins that are coded by the genes that are reported to be mutated in HSP and PLS patients. We applied a very stringent selection criteria when determining which proteins to include in this study. In total, 103 genes were investigated for their binding partners. 7 of them did not have any interactions according to our selection criteria. (Please refer to Section 2 and please see Supplementary Table S2, for the complete list of proteins, their binding partners and all the references cited for their interactions. The proteins that have 3 or more binding partners shown in red). We then collected the names of the binding partners for each protein that is coded by the mutated gene in HSP and PLS patients. Table 2 shows examples of proteins coded by the mutated genes and the proteins they are reported to have direct interaction. (Due to restrictions in space, we can only give an example of the proteins and their interaction partners and please see Supplementary Table S2 for the complete list with references.)

Investigation of protein-protein interactions among proteins that are causative or associated with HSP and PLS when their coding genes are mutated, yielded a total of 322 proteins, which had more than 3 binding partners. A protein with multiple different interaction partners, suggests its significant involvement in cellular events. When such proteins are mutated, cellular events that are mediated by their interaction would be adversely affected. Therefore, in an effort to identify “key” cellular events that are perturbed when mutated proteins are expressed in UMNs, we investigated proteins with the highest level of connections.

**Figure 1:**
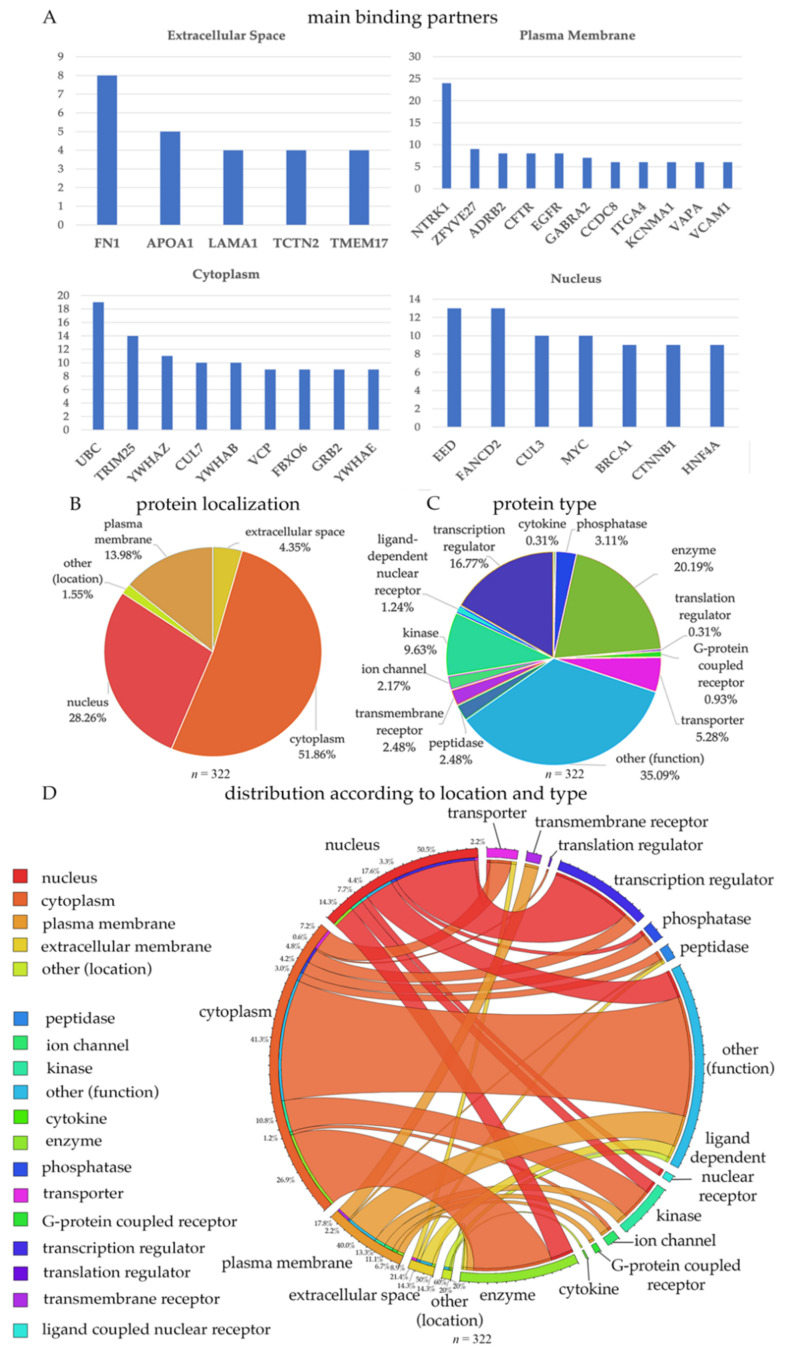
Binding partners of proteins coded by the mutated genes detected in HSP and PLS patients. (**A**) Bar graph representation of proteins with the highest number of bindings partners, located in the extracellular matrix, plasma membrane, cytoplasm and nucleus. Percent distribution of proteins based on location (**B**) and function (**C**). (**D**) Circle representation of proteins based on location and type.

Out of the 322 proteins that were identified, some were secreted proteins (present in the extracellular space; (*n* = 14)), some were present in the plasma membrane (*n* = 45), in the cytoplasm (*n* = 167), or in the nucleus (*n* = 91). For example, FN1, fibronectin 1 is an extracellular protein, which had direct interactions with 8 other proteins (Figure 1A). APOA1, LAMA1 and TCTN2 were other proteins with the highest number of interaction partners. LAMA1 is the alpha 1 subunits of laminin, and detection of these proteins may suggest defects in extracellular matrix and cell-cell interactions. Interestingly, numerous growth factor receptors emerged proteins located in the plasma membrane with numerous binding partners. For example, NTRK1 (TrkA receptor), CFTR (CNTF receptor), EGFR (EGF receptor) were among those with highest number of interactions, suggesting that NGF, CNTF, and EGF signaling occurs in UMNs.

Out of all ion channels and ion channel subunits, KCNMA1 (potassium-calcium-activated channel subfamily M alpha1 subunit) emerged as one that has the highest number of direct interactions. This subunit is important for controlling neuronal excitability and becomes active due to increased levels of cytosolic Ca^+2^, or Mg^2+^ in the cytoplasm [22,23,24,25]. Among cytoplasmic proteins, ubiquitin (UBC) had the highest level of interaction, followed by TRIM25, CUL7, YWHAZ, YWHAB, VCP, FBXO6, GRR2, and YWHAE. Interestingly, three different members of the 14-3-3 pathway (i.e., YWHAZ, YWHAB, and YWHAE) were also identified as cytoplasmic interacting proteins. These are largely conserved proteins with many important cellular functions, such as intracellular transport, metabolism, protein trafficking, and signal transduction [15,26]. TRIM25 is an important protein that is involved in innate immune defense especially after viral infection [27]. FBXO6 and VCP are both involved in the endoplasmic reticulum-associated degradation (ERAD) pathway for misfolded proteins [28,29,30,31,32,33]. In the nucleus, some of the most interacting proteins were FANCD2, CUL3, CTNNB1, and HNF4A.

About half of 332 proteins were in the cytoplasm (51%), 28.3% in the nucleus, 13.9% on the plasma membrane, and 4.3% were secreted proteins. A large population of proteins had multiple functions (other; 35.09%), 20.19% were enzymes, and 16.7% were transcription regulators (Figure 1B,C). The location and the function of proteins were merged (Figure 1D) to reveal the functional distribution of proteins located in different compartments of the neuron. According to our merged visualization of protein type and location, 26.9% of the cytoplasmic proteins were enzymes, 40% of the plasma membrane proteins were ligand dependent nuclear receptors and 50.5% of the nuclear proteins were transcription regulators.

**Figure 2:**
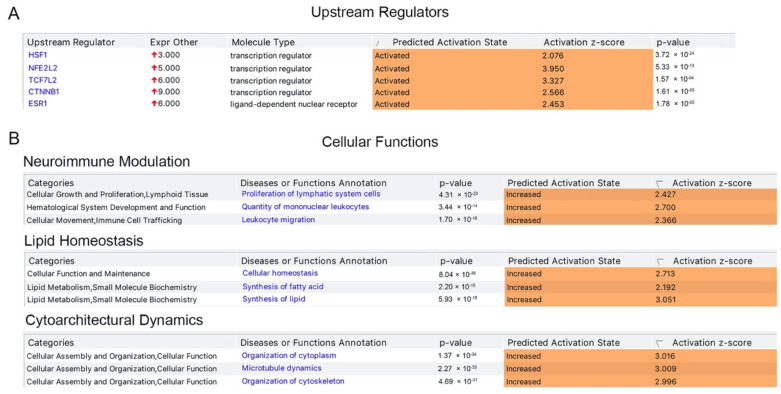
Upstream regulators and cellular functions associated with HSP and PLS. (**A**). Key upstream regulators, which are suggested to be activated in upper motor neurons. (**B**) Key cellular functions that are suggested to be active in upper motor neurons. Canonical pathways that are related to neuroimmune modulation, lipid homeostasis and cytoarchitectural dynamics are suggested to be activated with significance that cannot be explained by randomness.

We next investigated the presence of potential upstream regulator and found that a distinct set of transcriptional regulators were indeed responsible for the expression of numerous proteins present in the list, and thus had “activated” as their predicted activation state, with a positive activation z score and a significant *p* value, suggesting their potential importance for the health and function of UMNs. These transcription factors include HSF1, NFE2L2, TCF7L2, CTNNB1, and ESR1 (Figure 2A). Investigation of cellular functions these proteins are involved in revealed a signature of events. For example, canonical pathways that are related to the neuroimmunomodulation were significantly dominant with *p*-values ranging between 1.7 × 10^−18^ to 4.31 × 10^−20^, and a biased prediction to be in the “increased” state of activation (Figure 2B). Likewise, cellular events that are related to lipid homeostasis and maintaining cytoarchitectural dynamics displayed significant presence when compared to other cellular events. The presence of 322 proteins in cellular events related to lipid homeostasis and cytoarchitectural dynamics could not be explained by randomness and suggested their active involvement, especially in these cellular events.

**Figure 3:**
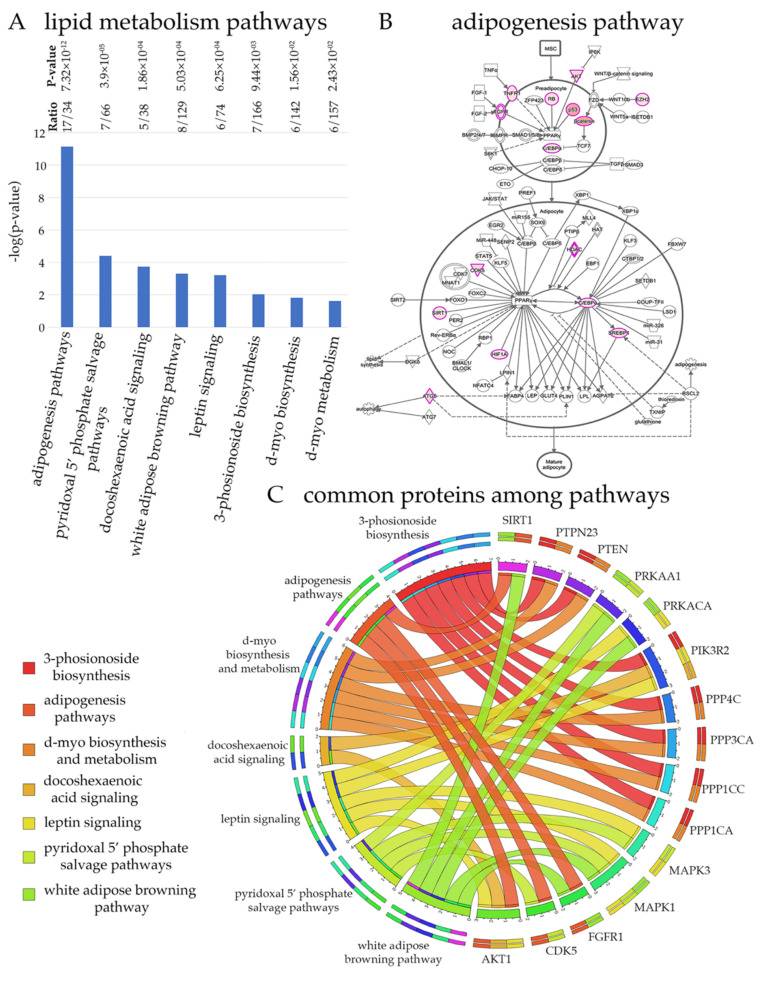
Maintaining lipid homeostasis is an important task for UMNs. (**A**) List of canonical pathways that are represented by HSP/PLS proteins. (**B**) Schematic drawing of adipogenesis pathway. Proteins with more than 3 binding partners are marked pink and proteins with more binding partners are depicted with increasing color intensity. (**C**) Circular representation of proteins commonly present among different canonical pathways related to lipid homeostasis.

Results from unbiased computational analyses suggest that maintaining lipid homeostasis is an important task for UMNs and that UMNs would be particularly vulnerable to alterations in lipid homeostasis (Figure 3). One of the best ways to understand the cellular events that are important for UMNs is to investigate the canonical pathways these 322 proteins mostly belong to (Figure 3A). If a canonical pathway is required for the health and function of UMNs, we would expect high level representation, and higher ratios of these proteins in that particular canonical pathway. If the canonical pathway is not related to the health or function of UMNs, these 322 key proteins would not be associated with them.

Many of the cellular events related to maintaining lipid homeostasis were represented. One of the most significant (*p* = 7.32 × 10^−12^) was the adipogenesis pathway (i.e 17 of the 34 proteins were present within the 322 HSP/PLS proteins, and with a *p* value that cannot be explained by randomness). In addition, leptin signaling, and white adipose browning pathway are highlighted for the high ratio levels, and significance. Visualization of the adipogenesis pathway also confirmed the presence of numerous proteins at key sites and with important functions (Figure 3B). The color intensity of overlapping proteins increases proportionally with the number of binding proteins and ranges from light pink to darker pink. When all the proteins present in canonical pathways related to lipid homeostasis were analyzed together in a circular fashion, the proteins that were present in different canonical pathways were identified (Figure 3C). Some proteins appeared to be more important as they were present in many different canonical pathways. For example, among all proteins, PIK3R2, MAPK1, and AKT1 were suggested to be particularly important for the modulation of lipid homeostasis related cellular events. Please refer to Supplementary Figure S1 for the detailed pathways for lipid homeostasis and Supplementary Table S3 for the list of proteins that are observed in each pathway.

**Figure 4:**
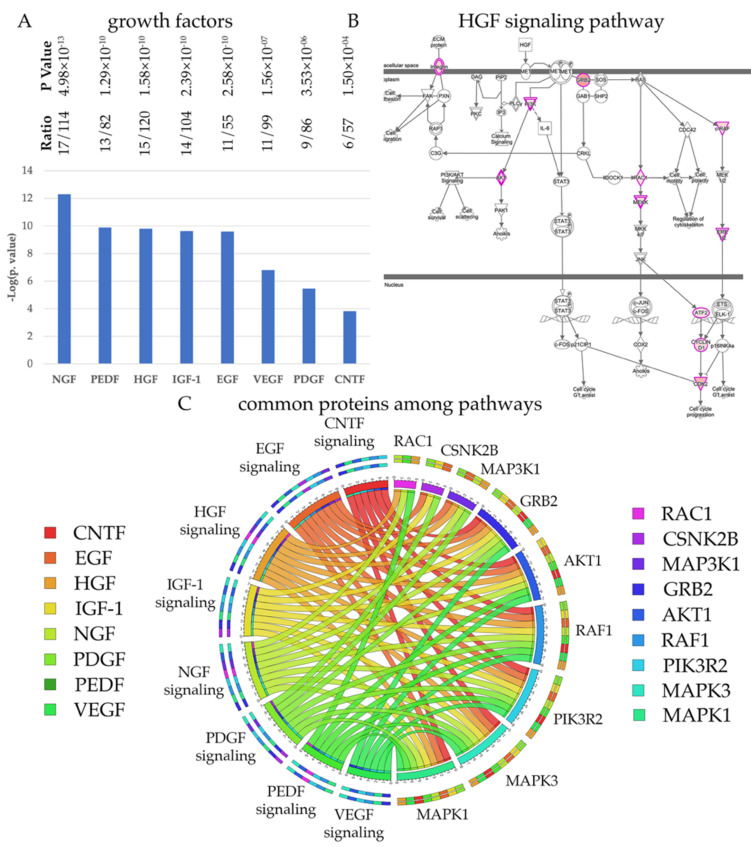
UMNs require a distinct set of growth factors. (**A**) Bar graphs of canonical pathways involved in growth factor mediated signaling. (**B**) Image of HGF signaling pathway, representing the extent of HSP/PLS protein involvement. Proteins with higher binding partners are marked with increasing color intensity. (**C**) Circular representation of HSP/PLS proteins that are commonly present among different canonical pathways.

Every neuron depends on growth factors for survival, but different neuron populations require a distinct set of growth factors. In an effort to understand whether UMNs have a preference among growth factors, we investigated the distribution of HSP/PLS proteins among cellular events that are related to growth factor mediated signaling (Figure 4). We find that the 322 HSP/PLS proteins are mostly involved in NGF, HGF, CNTF, IGF-1, EGF, PEDF, VEGF and PDGF signaling (Figure 4A), suggesting that UMNs would respond to these growth factors. For example, 15 of the 322 HSP/PLS proteins were present in the HGF signaling pathway and were located at key active and converging sites (Figure 4B). Interestingly, the circular graph also demonstrated that some of the kinases were shared among pathways and that RAF1, CSNK2B, MAPK1, MAPK3, AKT1, PIK3R2 were among the kinases that are exceptionally important for numerous signaling pathways (Figure 4C). It is also important to note that PIK3R2, MAPK1, and AKT1 were present and common between canonical pathways that are important for maintaining lipid homeostasis and growth factor mediated signaling, suggesting that their modulation with respect to UMN health and function would be of great interest. Please refer to Supplementary Figure S1 for detailed pathways for growth factors and Supplementary Table S4 for the list of proteins that are observed in each pathway.

## 4. Discussion

There are thousands of different neuron populations in our brain and they function within a circuitry. Initiation and modulation of voluntary movement is a result of a functional motor neuron circuitry. One of the most important neuron populations for the brain component of this circuitry is the UMNs. They have the unique function to receive, integrate and convey the cerebral cortex’s input to spinal cord targets.

When UMNs fail to perform their function, the motor neuron circuitry begins to fail, and this has a functional outcome in patients, such as spasticity and inability to initiate voluntary movement. An important key to this puzzle are the mutations detected in patients [16]. Each mutated gene codes for a protein, and for a disease to develop when that particular gene is mutated can only be possible when the protein product of that particular gene is exceptionally important for the health and the function of the neuron that degenerates.

The genetic components of rare diseases are beginning to emerge at a very rapid pace. Mutations in certain genes are detected in patients and some of these mutations are either directly linked or associated with the disease. It is still unclear, however, why a mutation in one gene leads to vulnerability of a distinct neuron population and degeneration of only a specific neuronal circuitry, when the same gene is present in the DNA of all cells in the body. Why does this particular mutation affect only a very select set of neurons, and how can this one mutation lead to neurodegeneration?

We think that this complex question of selective vulnerability can be answered by the concept of relevance and convergence. A mutation in a gene causes a disease only when the protein product of that gene plays an irreplaceable and very significant role in the neuron that has key function within the circuitry that degenerates. Even though all cells and neurons have the same DNA and they have equal access to the same genome, the genes they choose to express show a wide variation. Not all neurons express the same genes. In fact, the genes the neurons express have direct correlation and relation to the proteins they need for their health and specific function. Therefore, compiling genes that are mutated in HSP and PLS patients is the first step to understand the cellular events that are most relevant to UMNs.

Since proteins cannot function alone and must be part of a canonical pathway to undertake a cellular event, their binding partners, interactome domains and upstream regulators must be revealed to shed light onto the cellular events that are primarily involved in neuronal function and in neuronal vulnerability. We, therefore, investigated the list of proteins, which have been reported to have direct binding interactions that are experimentally observed and previously reported. This allowed us to begin to identify the protein landscape for UMNs, namely the proteins that are particularly important for their cellular function. For example, maintaining lipid homeostasis appears to be an important task for UMNs. Given the fact that the UMNs have very long axon and very elaborate apical dendrite, the extent of their cellular membrane is one of the most prominent among other cortical neurons. In addition, the membrane of numerous critically important organelles also requires specialized lipids. Therefore, the UMNs seem to greatly invest in cellular events and canonical pathways that are related to maintaining lipid balance [34], and perturbation of this balance could indeed be one of the reasons that is responsible for their vulnerability.

Maintaining cytoarchitectural integrity also emerges as one of the cellular events that is particularly important for the UMNs along with proper control of microtubule dynamics through activation of relevant canonical pathways. This ensures cytoarchitectural integrity is sustained and the intracellular dynamics are properly controlled. Since UMNs are one of the largest and most polarized neuron populations in the motor cortex, sustaining the stability and the cellular integrity of this large and delicate neuron population requires extra attention and activation of numerous key canonical pathways. Likewise, mutations that perturb microtubule dynamics and actin stability would thus make these neurons vulnerable to degeneration.

Neuroimmunomodulation and its contribution to neurodegeneration has been vastly studied [35,36,37,38,39,40,41,42]. Maintaining a healthy interaction between neurons and the astrocytes, microglia, infiltrating monocytes, and other cells that initiate and modulate neuroimmune response is of great importance for neuronal health. Once that interaction is perturbed and the vicious cycle is initiated, the neurons display a rather fast deterioration. Therefore, the UMN appear to keep the cellular events active to ensure a fine balance between neuroimmune cells, and it is possible that any mutation in genes that code for key proteins that maintain and sustain this balance are rather detrimental for the health and stability of UMNs.

Even though unbiased computational analysis and large-data management tool boxes are important to suggest key cellular events, such studies also come with potential caveats. The knowledge base is generated by curated articles that also contain in vitro and even in silico data. Unfortunately, some of these findings may not be replicated in vivo settings. For example, KLC2 was reported to bind to APP in vitro [43,44,45], but such interaction could not be confirmed in vivo [46]. Similarly, data from non-neuronal cells, cell lines, cancer cell lines are also included broadly in the curated data set, making it very hard to distinguish a true finding from noise, or a false call. Therefore, the initial selection criteria is exceptionally important. We applied one of the most stringent criteria, and selected findings with experimentally observed direct interaction, experiments that used neurons, mammalian systems and experiments that are peer reviewed, published and confirmed. We excluded findings in silico, in non-neuronal cells or non-mammalian systems, which refined our analyses moving forward. In an effort to improve stringency, we also included proteins with more than 3 binding partners and excluded the ones that had 2 or only 1 interaction. We therefore, suggest that the list of proteins included in this study are worthy of attention.

The unbiased computational protein-protein interaction domain analyses suggested the presence of a very distinct set of transcription factors that are upstream regulators of many proteins involved in key cellular events. Therefore, it is now of great importance to investigate whether modulation of their expression would enhance the expression profile of key contributors to UMN health and function and whether they would be potential targets for future gene therapy applications. Interestingly, recent studies have shown that expression of HSF1 (heat shock transcription factor 1), one of the master regulator of heat shock response, was able to reduce TDP-43 protein aggregation in neural stem cells [47]. HSF1 was also implicated in protein homeostasis, and mitochondrial function [48]. NFE2L2 (nuclear factor E2-related factor 2) has also been reported for its impact on reducing oxidative stress, inflammation [49]. TCF7L2 (Transcription factor 7-like 2) is a member of the T-cell factor/lymphoid enhancer factor family and it plays important role in cellular metabolism, especially in lipid dynamics. It down regulates glucogenesis and enhances lipid accumulation in different cells. It also influences adipogenesis [50]. These reports further suggest that the upstream regulators identified by the non-biased large data management tool box with high significance that cannot be explained by randomness, may offer a great insight into the UMN survival in diseases, such as HSP and PLS.

Growth factors are important for neurons to survive and thrive. However, not all neurons respond to the same set of growth factors. Our analyses suggest that UMNs indeed have a very distinct selection of growth factors that they respond to. For example, many of the proteins identified by interaction partners are involved in NGF, IGF1, VEGF, HGF, and CNTF mediated signaling and therefore suggest that UMNs respond to these growth factors. It is possible that a combination or a cocktail of these factors will need to be developed and tested for their ability to improve the health and the function of UMNs. Interestingly, the signaling cascade of events converge on key kinases, and especially the MAPK3, MAPK2, AKT1, and PIK3R2 emerge to be key enzymes with very important function especially for the UMNs. It would be important to investigate how modulation of their function would impact the health of diseased UMNs.

## 5. Conclusions

UMNs degenerate in HSP and PLS patients, even though these are two distinct and potentially unrelated neurodegenerative diseases. However, the gene mutations hold the clue for the cellular events that are primarily important for the health and stability of UMNs. Investigation of protein-protein interactions, begin to reveal the canonical pathways and the cellular events that are particularly crucial for UMNs. This information not only helps us understand how UMNs retain their cellular homeostasis, but also reveals key potential targets for future therapeutic interventions.

## Figures and Tables

**Table 1:** List of genes that are either directly linked or associated with HSP and PLS.

HSP CAUSATIVE GENES
ALS2, ALDH18A1, AP4B1, AP4E1, AP4M1, AP4S1, AP5Z1, ARL6IP1, ATAD3A, ATL1, B4GALNT1, BICD2, BSCL2, C19orf12, CAPN1, CCT5, CYP2U1, CYP7B1, DDHD1, DDHD2, ERLIN2, ENTPD1, EPT1, ERLIN1, FARS2, FA2H, GBA2, IBA57, KCNA2, KIAA0196/WASHC5, KIF1A, KIF5A, KY, L1CAM, LYST, NIPA1, NT5C2, PCYT2, PLP1, PNPLA6, RAB3GAP2, REEP1, REEP2, RTN2, SETX, SLC33A1, SPAST, SPG11, SPG21/ACP33, SPG7, SPG80/UBAP1, TECPR2, TFG, TUBB4A, VCP, VPS37A, ZFYVE26, ZFYVE27
HSP ASSOCIATED GENES
ABCD1, ADAR, ALDH3A2, AMPD2, ARSA, ARSI, ATP13A2, CPT1C, ELOVL1, FLRT1, GALC, GCH1, GRN, HSPD1, HSP60, IFIH1, KIF1C, MAG, MARS, MFN2, MT-ATP6, OPA1, OPA3, PGAP1, PLA2G6, POLR3A, SERAC1, SLC16A2, SPART, SPG20, TRPV4, USP8, WDR48, ZFR
HSP LINKED GENES
C12orf65, CYP27A1, GAD1, GJC2, KLC2, RIPK5/DSTYK, UCHL1
PLS GENES
CAUSATIVE: ALS2 ASSOCIATED: ALS15, C9orf72, DCTN1, ERLIN2, PARK2 LINKED: FIG4, SPG7

**Table 2:** Examples of proteins that are coded by the mutated genes detected in HSP and PLS patients, and their binding partners. Please see Supplementary Table S2 with the complete list, associated with references.

Protein	Binding Partners
ALDH18A1	ADRB2, AGTRAP, C1QBP, CDK2, CMTM5, CUL3, EED, G3BP1, GABRA2, GOLT1B, HDAC5, MOV10, MRPL58, MYC, NFATC2, NR3C1, NTRK1, NXF1, SHMT2, SIRT7, STAU1, TCF3, VCP
AP4E1	ALB, AP4B1, AP4M1, AP4S1, ARF1, ELAVL1, GOLIM4, LAMA1, MAP3K4, SUV39H2, TEPSIN, TFAP2A, TMEM17, XPO1, YME1L1
AP4S1	AP4B1, AP4E1, AP4M1, APP, CDC73, GOLIM4, GRB2, HLTF, LAMA1, MAP3K4, SUV39H2, TEPSIN, TFAP2A, YME1L1
ERLIN1	AMFR, C1QBP, CD2AP, CFTR, CHMP4B, COX15, CUL7, DUSP3, EDEM3, FAF2, FANCD2, FBXO6, GABRA2, GOLT1B, HNF4A, INSIG1, ITPR1, Ktn1, NTRK1, PKN2, RAB5C, RAB7A, RNF170, SPAST, STOM, SUZ12, SYVN1, TMED2, TRAF6, TRIM25, TSG101, UBC, VAPA, VDAC1
FARS2	AGTRAP, APPL1, CMTM5, CUL3, HNRNPA1, ISG15, KRT31, KRT40, KRTAP10-3, KRTAP10-7, KRTAP10-9, LOC100996763/NOTCH2NL, MID2, MRPL58, NXF1, PDHA1, SHMT2, STAT5A, TRIM27, TRIM54
GRN	AHCYL2, ATN1, BRCA1, CCDC8, CDK2, CDK9, CFTR, CSNK2B, CTTN, CUL7, EED, EGFR, FBXO6, GRIA2, HECW2, HSP90AA1, HSP90AB1, HSPA4, KRTAP10-7, MAPK1, NF2, NPM1, NTRK1, NXF1, PIK3R2, POT1, PPP2CA, PRKAA1, RAC1, SIRT3, TNFRSF1A, TUBA1C, VHL
KIF1A	APP, AR, COX15, DLG4, EGFR, FMR1, FXR2, KIF1BP, LOC100996763/NOTCH2NL, MDFI, MID2, MTUS2, NTRK1, PLSCR1, PPP2CA, PSMA3, RAC1, RBPMS, SIRT1, SIRT7, SP1, TRAF1, TRIM27, UBC
KIF1C	ARF1, BICD2, HSPA8, KIF1BP, KSR1, MYH9, PRKAA1, STAU1, TRIM25, USP21, YWHAB, YWHAE, YWHAG, YWHAQ, YWHAZ
KIF5A	ACTB, APP, BICD2, DCTN1, DLG4, FANCD2, FMR1, GRB2, GRIA2, HACD3, KIF5B, KIF5C, KLC2, MDM2, MYCL, STAU1, TSG101, YAP1, YWHAE, ZFYVE27
KLC2	AIMP2, APP, CDH1, EZH2, GRIA2, KCNMA1, KIF5B, KIF5C, MYCL, NTRK1, PIK3R3, PPP4C, SCAMP2, SOD1, VCP, WDR70, YWHAB, YWHAE, YWHAG, YWHAH, YWHAQ, YWHAZ
MARS	AIMP2, BRCA1, CCDC8, CDK9, COPS5, CRY2, CUL1, CUL3, CUL7, CYLD, DLST, EED, EGFR, ESR1, FANCD2, FBXO25, FBXO6, FN1, G3BP1, HACD3, HDAC5, HSP90AA1, HUWE1, IKBKG, ILK, ITGA4, Ktn1, MAP3K1, MAP3K3, MCC, MCM2, MDM2, MYC, NTRK1, PDHA1, PKN2, RARS, RNF2, TRAF6, VCAM1
PARK2	ABL1, BAX, CCND1, CCT2, CDK5, CTNNB1, CUL1, EGFR, HDAC6, HSPA9, HSPD1, IKBKG, PDHA1, RNF31, SNCA, STUB1, TARDBP, TCP1, TP53, TRAF2, TUBB, VDAC1
SPAST	ALB, ATL1, CD2AP, CHMP1A, CHMP1B, CHMP2A, CHMP2B, CHMP4B, CHMP5, CLTA, CLTC, ELAVL1, ERLIN1, HECW2, HNF4A, IST1, PTPN23, SOAT1, STOM, TSG101, VCP
TFG	BRCA1, CAND1, CFTR, CHMP1A, CHMP1B, CHMP2A, CHMP4B, CHMP5, CLTA, COPS5, CUL1, CUL2, CUL3, CUL4A, CUL7, EWSR1, FANCD2, GRB2, HSPA5, IKBKG, IST1, MAP3K3, MAPK13, MCM2, MTUS2, NEDD8, NFATC2, NTRK1, PLSCR1, PPP1CA, PSEN1, RARS, RBPMS, SNX3, STAT5B, TMEM17, TRIM25, TSG101, UNK, YWHAZ
ZFR	BRCA1, CAND1, CCDC8, CEBPA, COPS5, CUL3, CUL7, DLG4, EED, EGFR, ELAVL1, FBXW11, FMR1, FOXP3, HDAC11, HNRNPA1, MOV10, NXF1, OBSL1, RNF2, RPA2, RPAP1, SIRT7, SMAD2, STAU1, TARDBP, TRIM25, VCP
ZFYVE27	APP, ATL1, ATP2A2, ATP5F1A, ATP5F1C, ATP5PB, C1QBP, CANX, CCT3, CCT4, CCT5, FKBP8, GNB1, GRIA2, HACD3, HSPA9, KIF5B, KIF5C, NCAM1, NCDN, PLP1, PPP3CA, PRKACA, RAB7A, REEP5, RTN4, VAPA, VAPB, YWHAE, YWHAZ, ZFYVE27

## Supplementary Materials

The following are available online at https://www.mdpi.com/article/10.3390/brainsci11050578/s1, Table S1: HSP and PLS genes with references, Table S2: HSP and PLS genes with their binding partners associated with the references for each direct interaction, Table S3: Proteins involved in Lipid homeostasis related pathways, Table S4: Proteins involved in growth factor signaling pathways, Figure S1: A complete list of all canonical pathways involved in growth factor signaling and lipid homeostasis.
