# Supplementary material for: Mutations and Protein Interaction Landscape Reveal Key Cellular Events Perturbed in Upper Motor Neurons with HSP and PLS
Source: Brain Sci. 2021 Apr 29;11(5):578. doi: 10.3390/brainsci11050578 (PMC8146506; doi:10.3390/brainsci11050578)

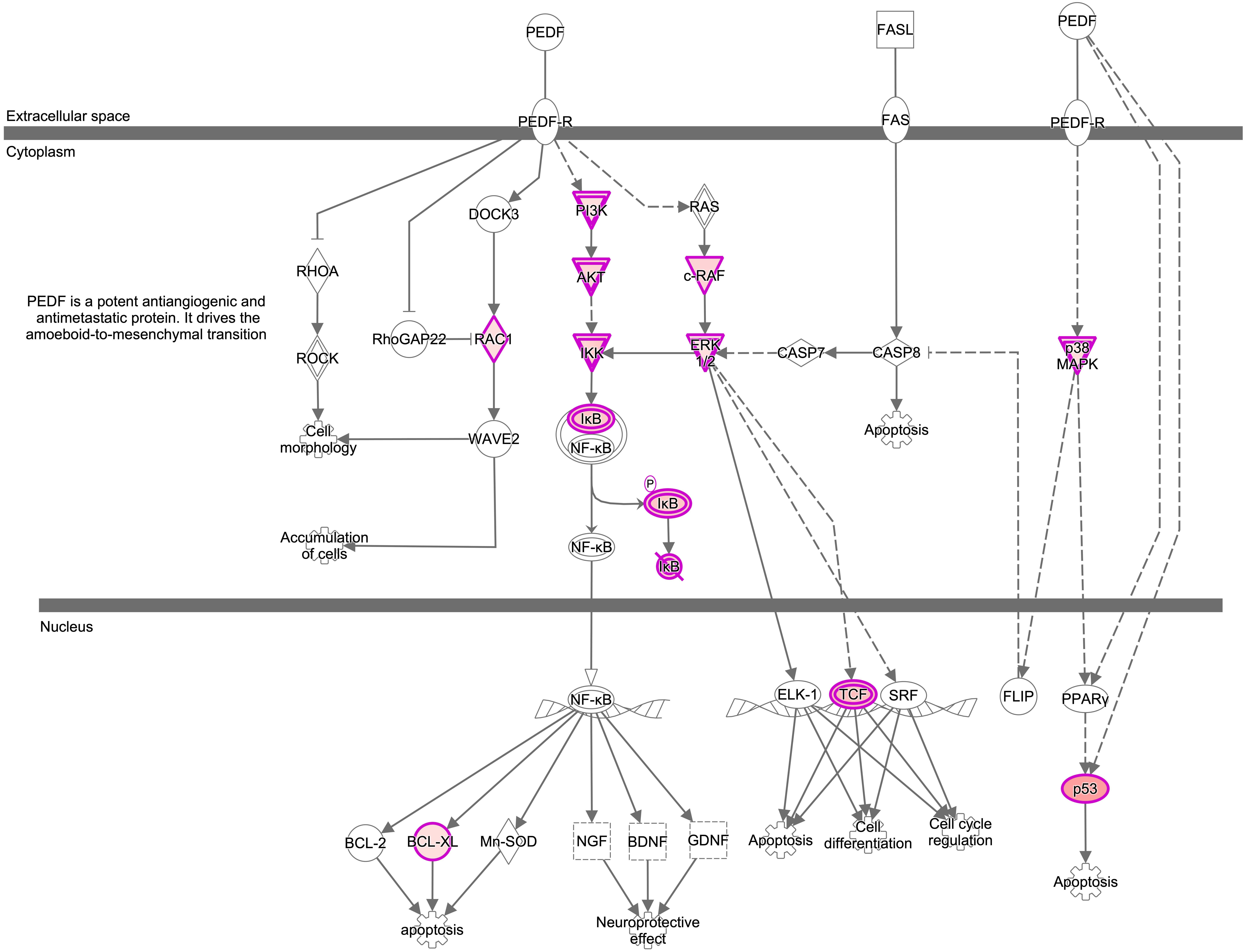
Extracellular space
Cytoplasm
Nucleus
PEDF
PEDF-R
FASL
FAS
PEDF
PEDF-R
RHOA
RhoGAP22
DOCK3
PI3K
RAS
AKT
c-RAF
PEDF is a potent antiangiogenic and antimetastatic protein. It drives the amoeboid-to-mesenchymal transition
ROCK
RAC1
IKK
ERK 1/2
CASP7
CASP8
p38 MAPK
IκB
NF-κB
Cell morphology
WAVE2
Apoptosis
P
IκB
Accumulation of cells
NF-κB
IκB
NF-κB
ELK-1
TCF
SRF
FLIP
PPARγ
p53
BCL-2
BCL-XL
Mn-SOD
NGF
BDNF
GDNF
Apoptosis
Cell differentiation
Cell cycle regulation
Apoptosis
apoptosis
Neuroprotective effect

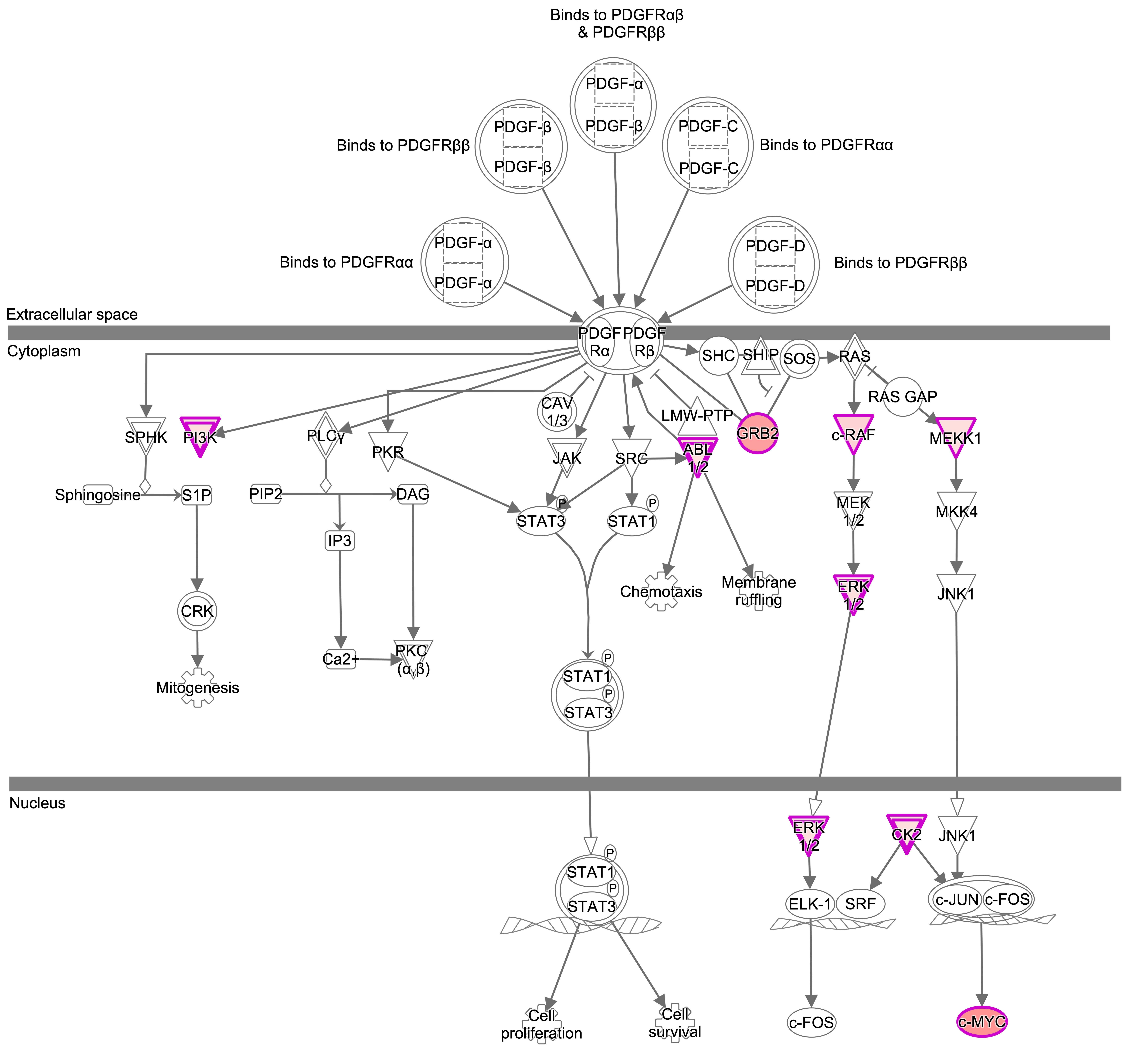

Binds to PDGFRαβ & PDGFRββ
PDGF-α
PDGF-β
Binds to PDGFRββ
PDGF-β
PDGF-β
PDGF-C
PDGF-C
Binds to PDGFRαα
Binds to PDGFRαα
PDGF-α
PDGF-α
PDGF-D
PDGF-D
Binds to PDGFRββ
Extracellular space
Cytoplasm
PDGF Rα
PDGF Rβ
SHC
SHIP
SOS
RAS
RAS GAP
CAV 1/3
LMW-PTP
GRB2
c-RAF
MEKK1
SPHK
PI3K
PLCγ
PKR
JAK
SRC
ABL 1/2
Sphingosine
S1P
PIP2
DAG
STAT3
STAT1
P
MEK 1/2
MKK4
IP3
Chemotaxis
Membrane ruffling
ERK 1/2
JNK1
CRK
Mitogenesis
Ca2+
PKC (α,β)
STAT1
STAT3
Nucleus
ERK 1/2
CK2
JNK1
ELK-1
SRF
c-JUN
c-FOS
Cell proliferation
Cell survival
c-FOS
c-MYC

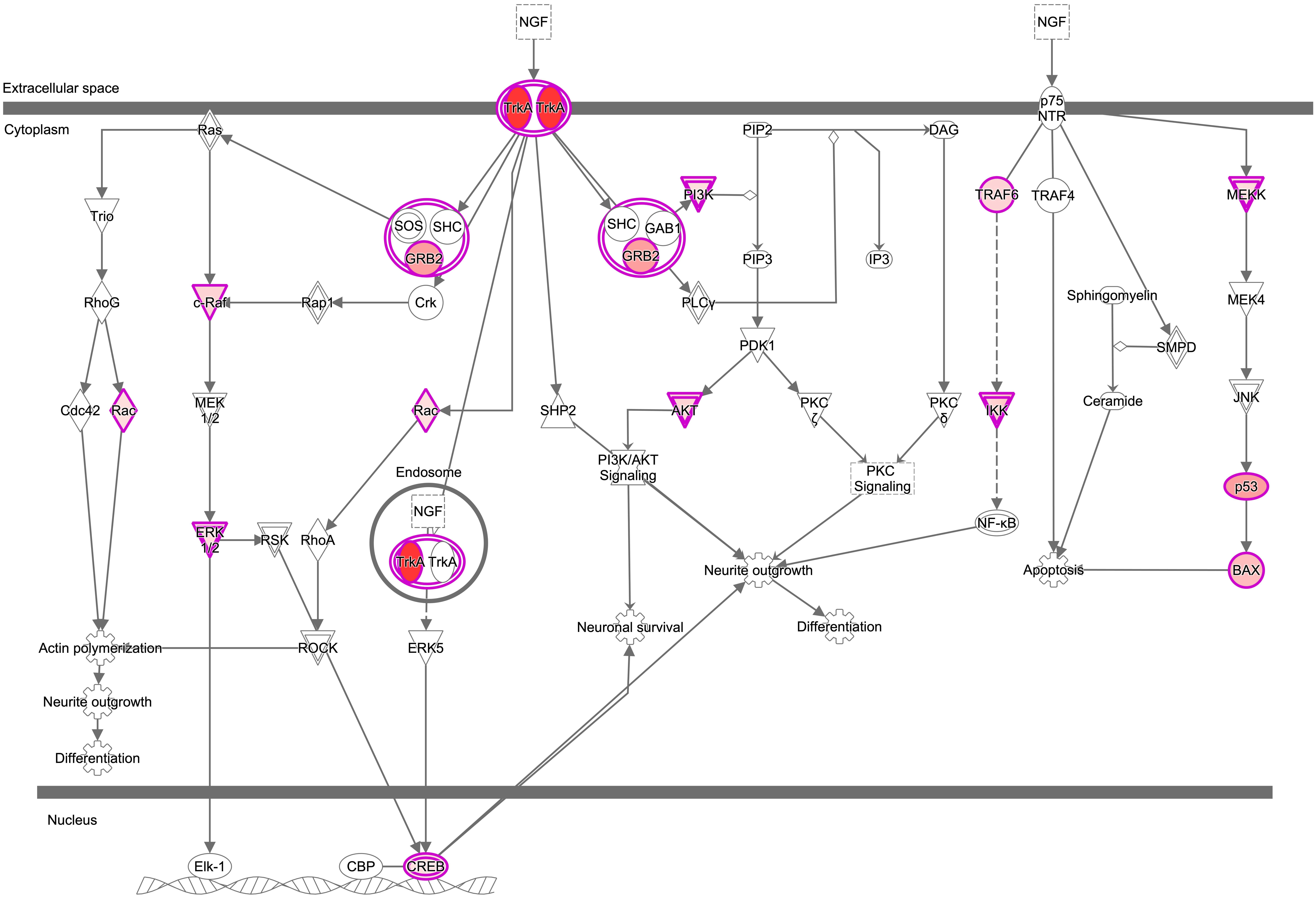

NGF
NGF
Extracellular space
TrkA
TrkA
p75
NTR
Cytoplasm
Ras
PIP2
DAG
PI3K
TRAF6
TRAF4
MEKK
Trio
SOS
SHC
GRB2
SHC
GAB1
GRB2
PIP3
IP3
RhoG
c-Raf
Rap1
Crk
PLCγ
Sphingomyelin
MEK4
PDK1
SMPD
Cdc42
Rac
MEK
1/2
Rac
SHP2
AKT
PKC
ζ
PKC
δ
IKK
Ceramide
JNK
Endosome
PI3K/AKT
Signaling
PKC
Signaling
p53
NGF
ERK
1/2
RSK
RhoA
TrkA
TrkA
NF-κB
Neurite outgrowth
Apoptosis
BAX
Neuronal survival
Differentiation
Actin polymerization
ROCK
ERK5
Neurite outgrowth
Differentiation
Nucleus
Elk-1
CBP
CREB

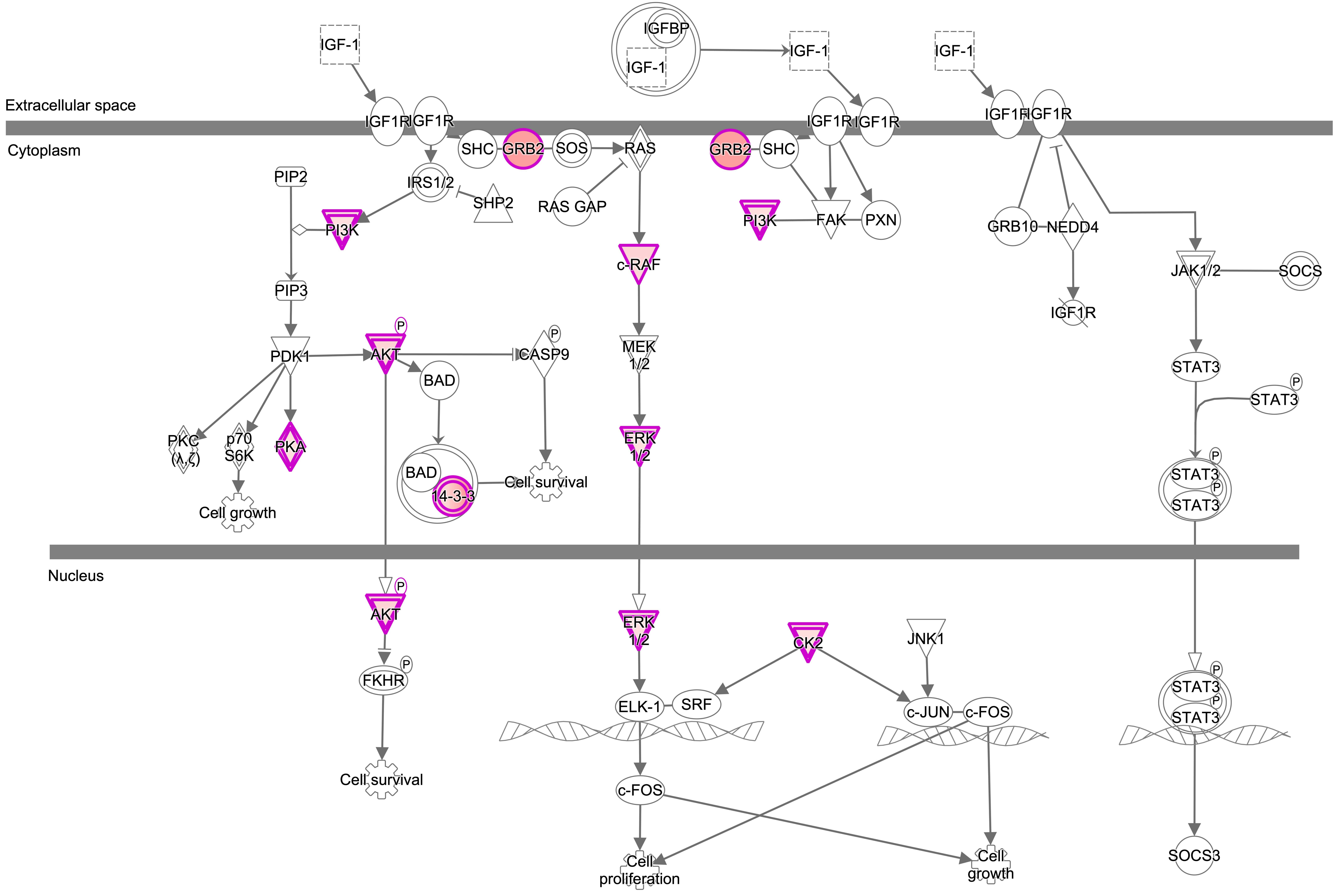
Extracellular space
Cytoplasm
Nucleus
IGF-1
IGFBP
IGF-1
IGF-1
IGF-1
IGF1R
IGF1R
IGF1R
IGF1R
IGF1R
IGF1R
SHC
GRB2
SOS
RAS
GRB2
SHC
PIP2
IRS1/2
SHP2
RAS GAP
PI3K
FAK
PXN
PI3K
GRB10
NEDD4
c-RAF
JAK1/2
SOCS
IGF1R
PIP3
P
AKT
PDK1
P
CASP9
MEK 1/2
STAT3
P
STAT3
BAD
PKC (λ,ζ)
p70 S6K
PKA
ERK 1/2
P
STAT3
P
STAT3
BAD
14-3-3
Cell survival
Cell growth
P
AKT
ERK 1/2
CK2
JNK1
P
STAT3
P
STAT3
P
FKHR
ELK-1
SRF
c-JUN
c-FOS
Cell survival
c-FOS
Cell proliferation
Cell growth
SOCS3

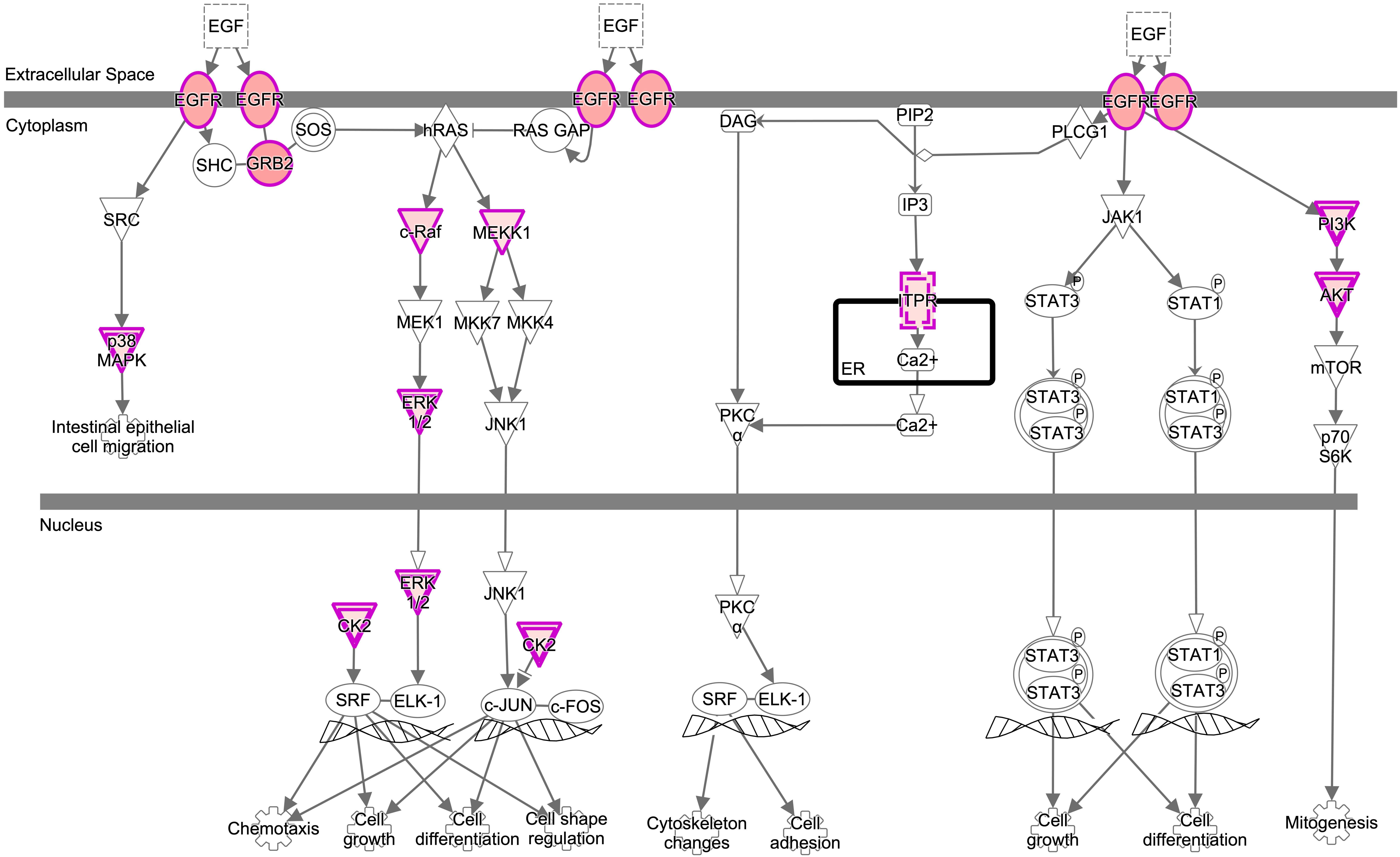

Extracellular Space
Cytoplasm
Nucleus
EGF
EGFR
SHC
GRB2
SOS
hRAS
RAS GAP
DAG
PIP2
PLCG1
IP3
ITPR
ER
Ca2+
SRC
p38 MAPK
Intestinal epithelial cell migration
c-Raf
MEKK1
MEK1
MKK7
MKK4
ERK 1/2
JNK1
PKC α
JAK1
STAT3
STAT1
P
PI3K
AKT
mTOR
p70 S6K
CK2
SRF
ELK-1
c-JUN
c-FOS
Chemotaxis
Cell growth
Cell differentiation
Cell shape regulation
Cytoskeleton changes
Cell adhesion
Mitogenesis

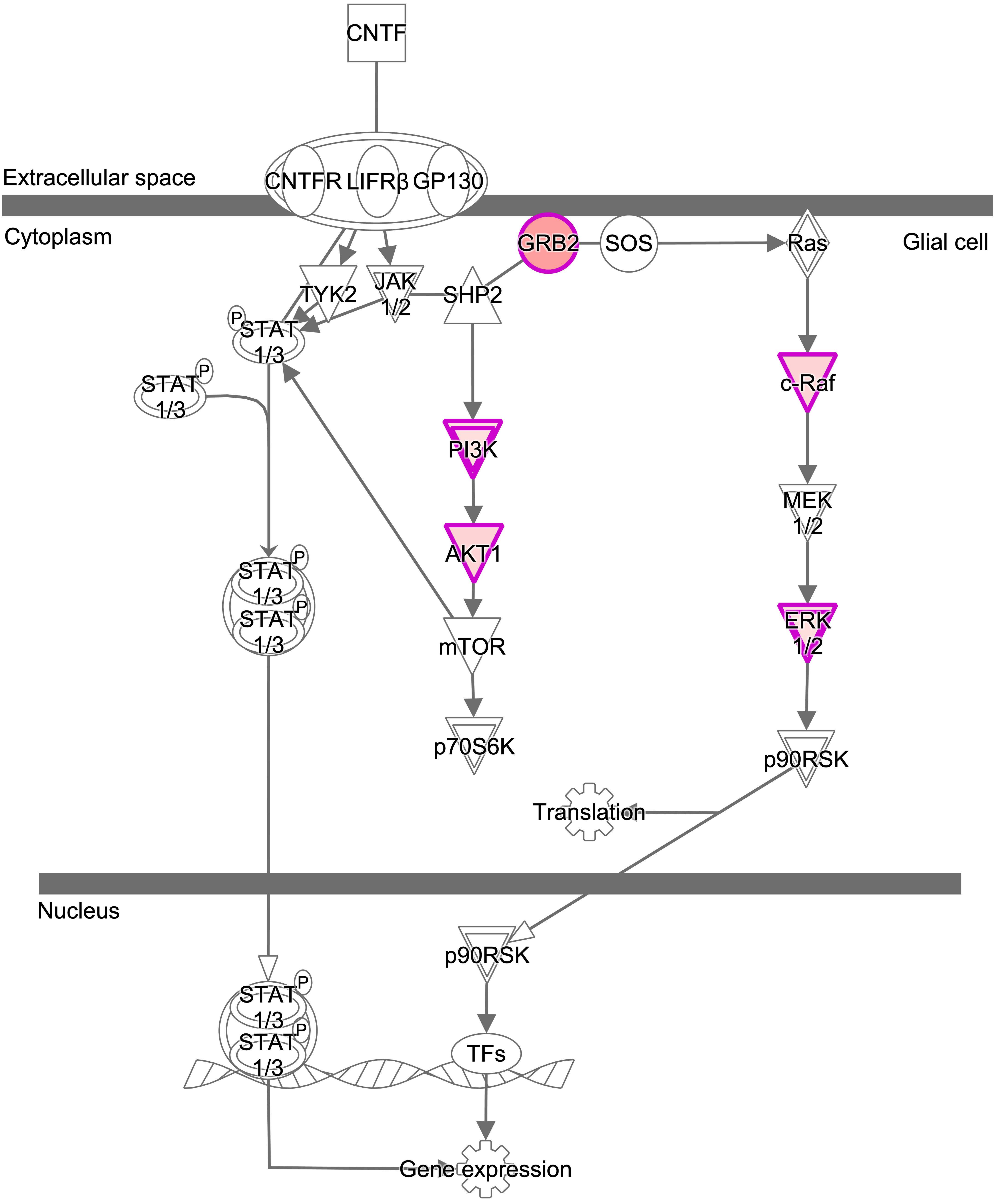
CNTF
Extracellular space
CNTFR
LIFRβ
GP130
Cytoplasm
GRB2
SOS
Ras
Glial cell
TYK2
JAK 1/2
SHP2
STAT 1/3
P
STAT 1/3
c-Raf
PI3K
MEK 1/2
AKT1
STAT 1/3
STAT 1/3
ERK 1/2
mTOR
p70S6K
p90RSK
Translation
Nucleus
p90RSK
STAT 1/3
STAT 1/3
TFs
Gene expression

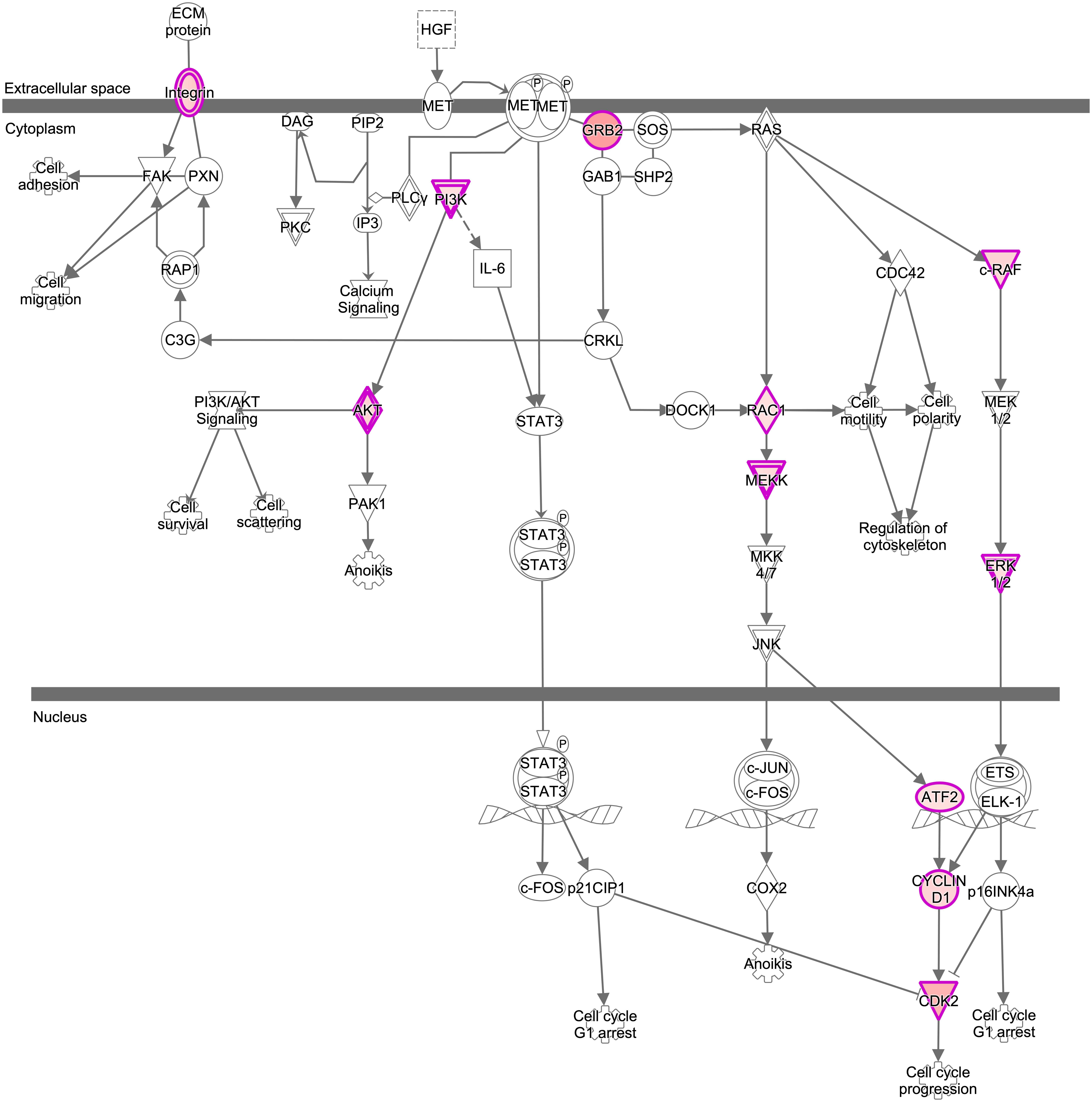
ECM protein
HGF
Extracellular space
Integrin
MET
MET
MET
P
P
Cytoplasm
DAG
PIP2
GRB2
SOS
RAS
GAB1
SHP2
Cell adhesion
FAK
PXN
PLCγ
PI3K
PKC
IP3
IL-6
RAP1
Cell migration
Calcium Signaling
CDC42
c-RAF
C3G
CRKL
PI3K/AKT Signaling
AKT
STAT3
DOCK1
RAC1
Cell motility
Cell polarity
MEK 1/2
MEKK
PAK1
Cell survival
Cell scattering
STAT3
STAT3
Regulation of cytoskeleton
Anoikis
MKK 4/7
ERK 1/2
JNK
Nucleus
STAT3
STAT3
c-JUN
c-FOS
ETS
ELK-1
ATF2
c-FOS
p21CIP1
COX2
CYCLIN D1
p16INK4a
Anoikis
CDK2
Cell cycle G1 arrest
Cell cycle G1 arrest
Cell cycle progression

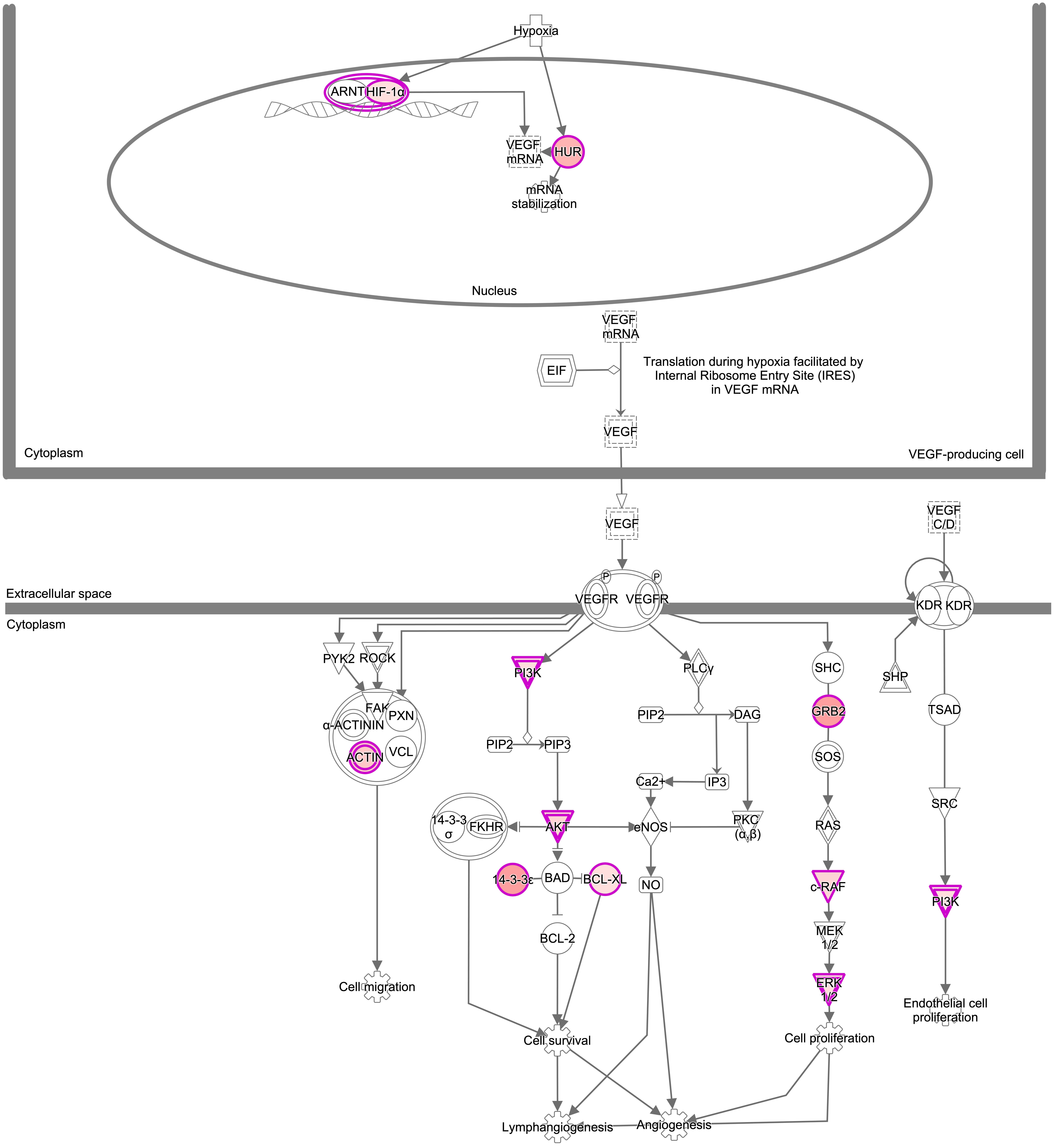

Hypoxia
ARNT
HIF-1α
VEGF mRNA
HUR
mRNA stabilization
Nucleus
VEGF mRNA
EIF
Translation during hypoxia facilitated by Internal Ribosome Entry Site (IRES) in VEGF mRNA
VEGF
Cytoplasm
VEGF-producing cell
VEGF
VEGF C/D
P
P
Extracellular space
VEGFR
VEGFR
KDR
KDR
Cytoplasm
PYK2
ROCK
PI3K
PLCγ
SHC
SHP
FAK
PXN
α-ACTININ
ACTIN
VCL
GRB2
TSAD
PIP2
DAG
PIP2
PIP3
SOS
Ca2+
IP3
SRC
RAS
14-3-3 σ
FKHR
AKT
eNOS
PKC (α,β)
14-3-3ε
BAD
BCL-XL
NO
c-RAF
PI3K
BCL-2
MEK 1/2
ERK 1/2
Cell migration
Endothelial cell proliferation
Cell survival
Cell proliferation
Lymphangiogenesis
Angiogenesis

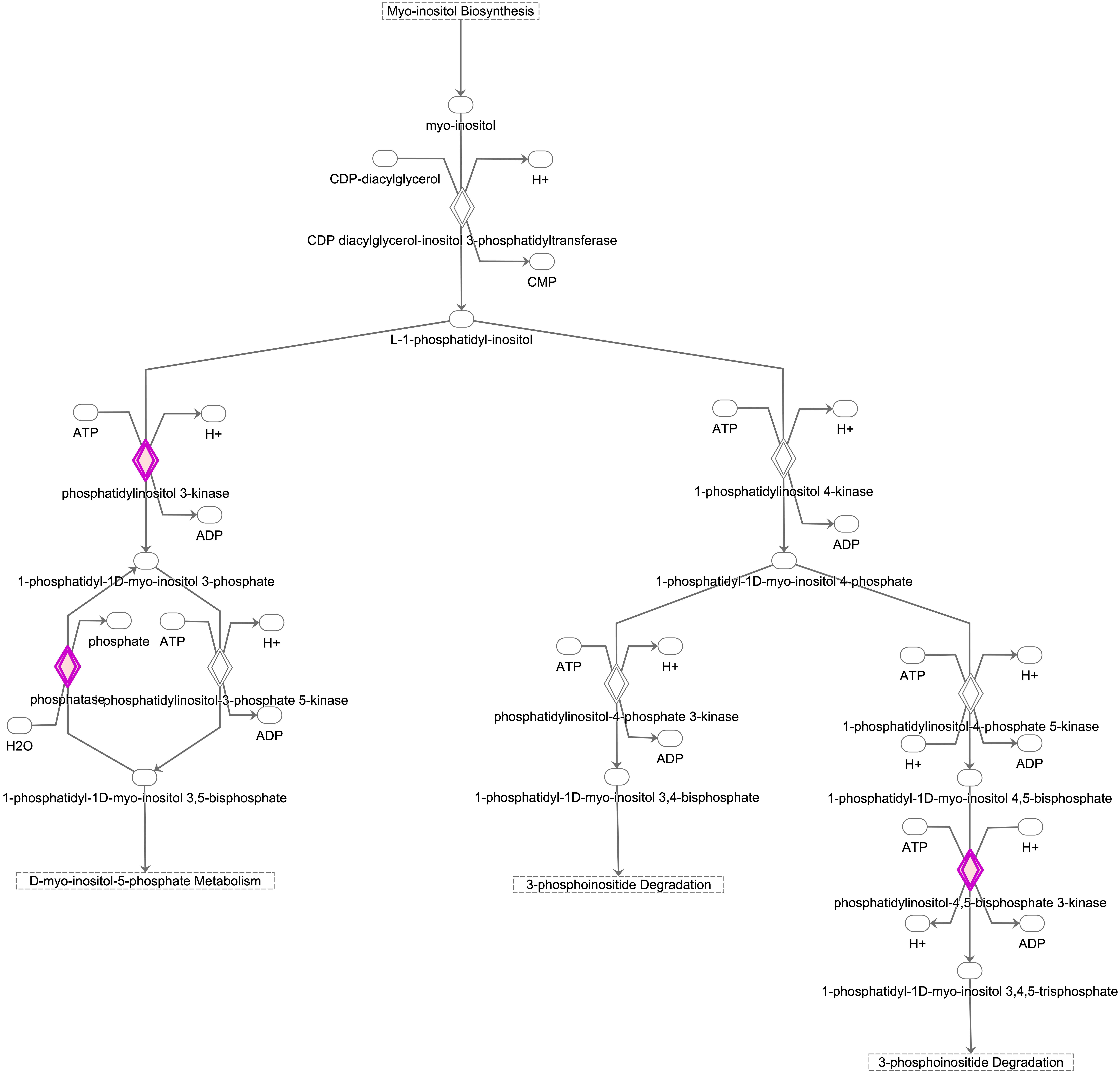
Myo-inositol Biosynthesis
myo-inositol
CDP-diacylglycerol
H+
CDP diacylglycerol-inositol 3-phosphatidyltransferase
CMP
L-1-phosphatidyl-inositol
ATP
H+
phosphatidylinositol 3-kinase
ADP
ATP
H+
1-phosphatidylinositol 4-kinase
ADP
1-phosphatidyl-1D-myo-inositol 3-phosphate
1-phosphatidyl-1D-myo-inositol 4-phosphate
phosphate
ATP
H+
phosphatase
phosphatidylinositol-3-phosphate 5-kinase
H2O
ADP
ATP
H+
phosphatidylinositol-4-phosphate 3-kinase
ADP
ATP
H+
1-phosphatidylinositol-4-phosphate 5-kinase
H+
ADP
1-phosphatidyl-1D-myo-inositol 3,5-bisphosphate
1-phosphatidyl-1D-myo-inositol 3,4-bisphosphate
1-phosphatidyl-1D-myo-inositol 4,5-bisphosphate
D-myo-inositol-5-phosphate Metabolism
3-phosphoinositide Degradation
ATP
H+
phosphatidylinositol-4,5-bisphosphate 3-kinase
H+
ADP
1-phosphatidyl-1D-myo-inositol 3,4,5-trisphosphate
3-phosphoinositide Degradation

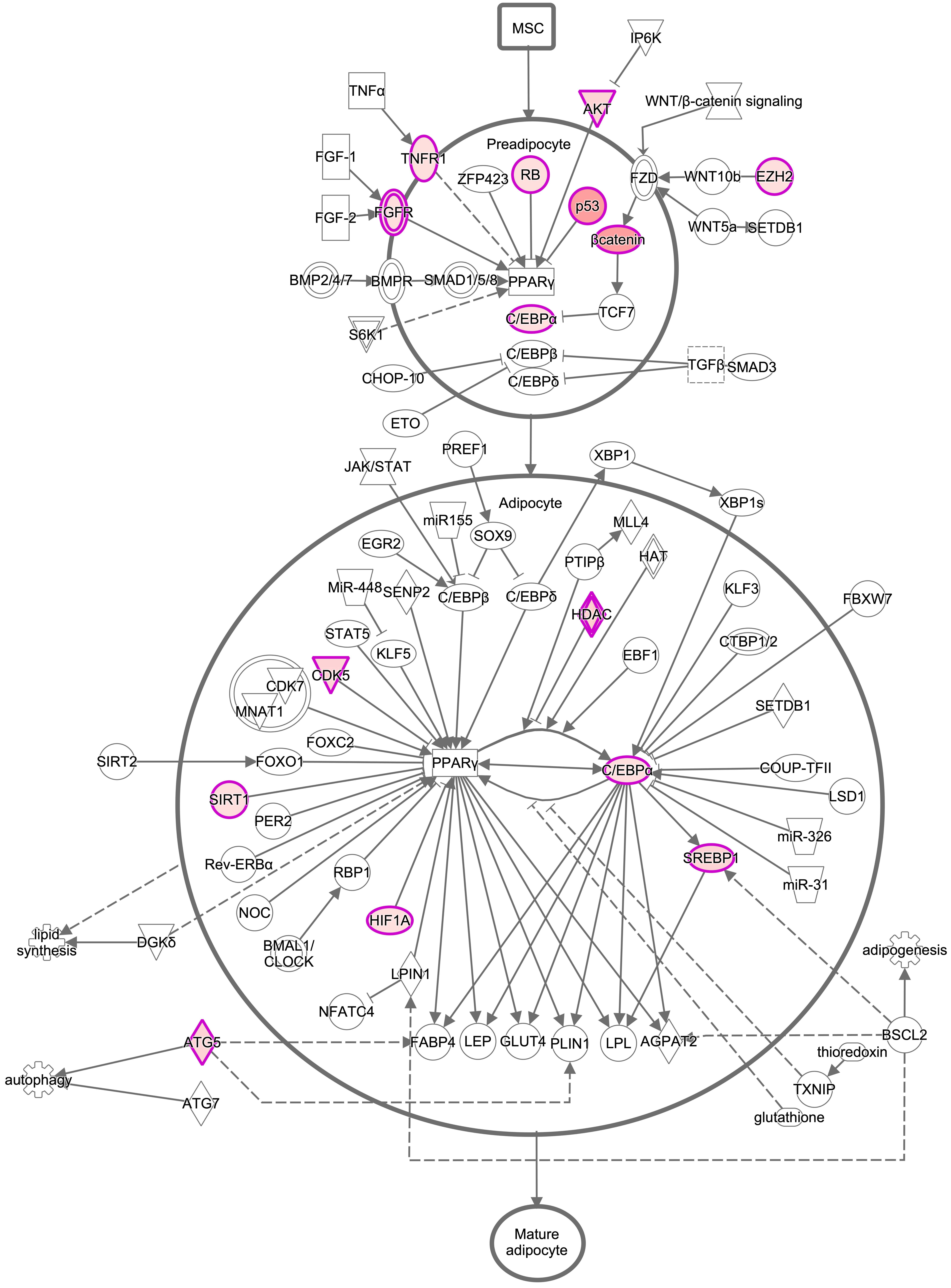

MSC
IP6K
TNFα
AKT
WNT/β-catenin signaling
Preadipocyte
FGF-1
TNFR1
ZFP423
RB
FZD
WNT10b
EZH2
p53
FGF-2
FGFR
βcatenin
WNT5a
SETDB1
BMP2/4/7
BMPR
SMAD1/5/8
PPARγ
C/EBPα
TCF7
S6K1
C/EBPβ
TGFβ
SMAD3
CHOP-10
C/EBPδ
ETO
PREF1
XBP1
JAK/STAT
XBP1s
Adipocyte
miR155
MLL4
SOX9
EGR2
HAT
PTIPβ
KLF3
MiR-448
SENP2
C/EBPβ
C/EBPδ
FBXW7
HDAC
STAT5
CTBP1/2
KLF5
EBF1
CDK5
CDK7
SETDB1
MNAT1
FOXC2
SIRT2
FOXO1
PPARγ
C/EBPα
COUP-TFII
SIRT1
LSD1
PER2
miR-326
SREBP1
Rev-ERBα
RBP1
miR-31
NOC
HIF1A
lipid synthesis
DGKδ
BMAL1/ CLOCK
adipogenesis
LPIN1
NFATC4
BSCL2
ATG5
FABP4
LEP
GLUT4
PLIN1
LPL
AGPAT2
thioredoxin
autophagy
TXNIP
ATG7
glutathione
Mature adipocyte

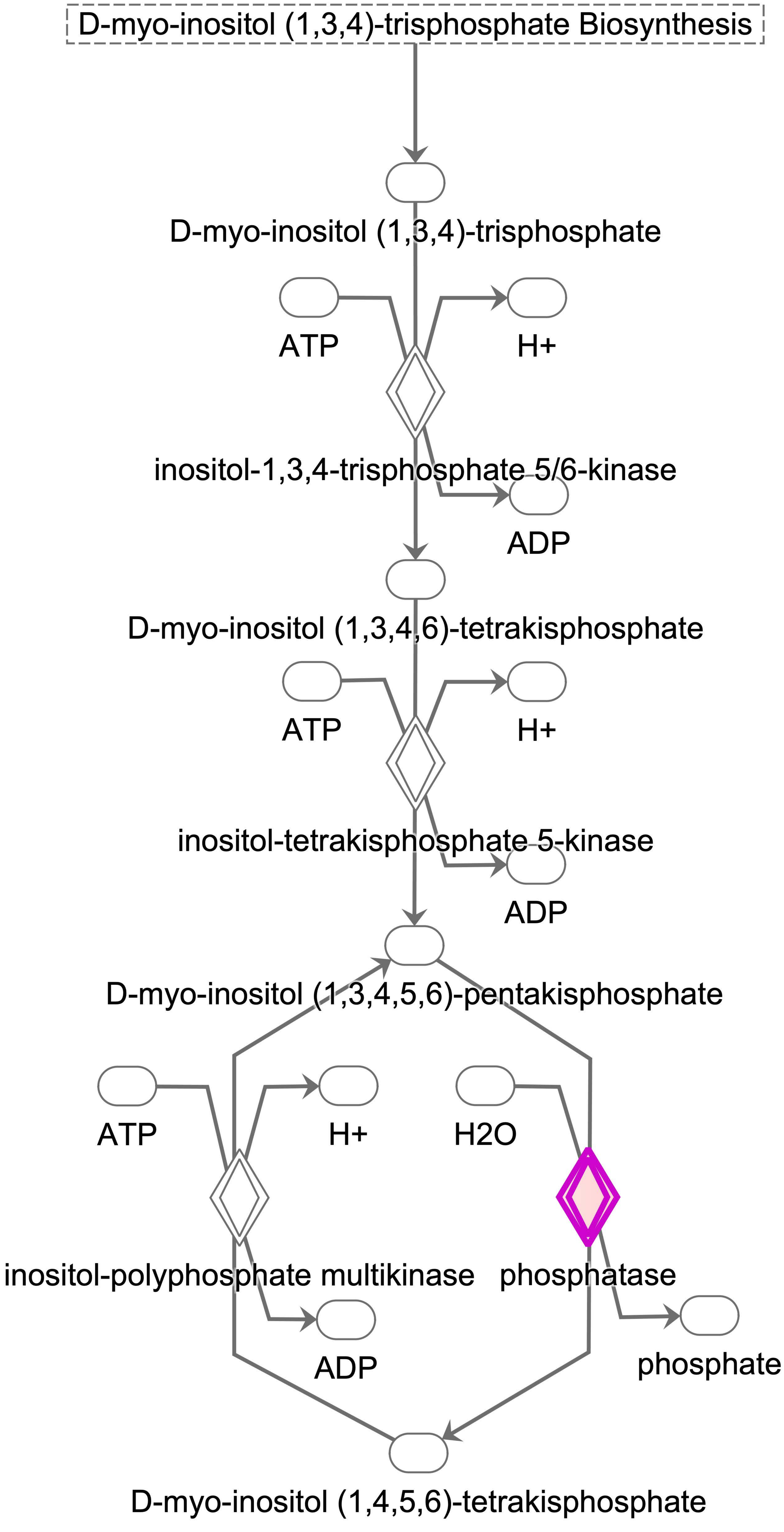
D-myo-inositol (1,3,4)-trisphosphate Biosynthesis
D-myo-inositol (1,3,4)-trisphosphate
ATP
H+
inositol-1,3,4-trisphosphate 5/6-kinase
ADP
D-myo-inositol (1,3,4,6)-tetrakisphosphate
ATP
H+
inositol-tetrakisphosphate 5-kinase
ADP
D-myo-inositol (1,3,4,5,6)-pentakisphosphate
ATP
H+
H2O
inositol-polyphosphate multikinase
phosphatase
ADP
phosphate
D-myo-inositol (1,4,5,6)-tetrakisphosphate

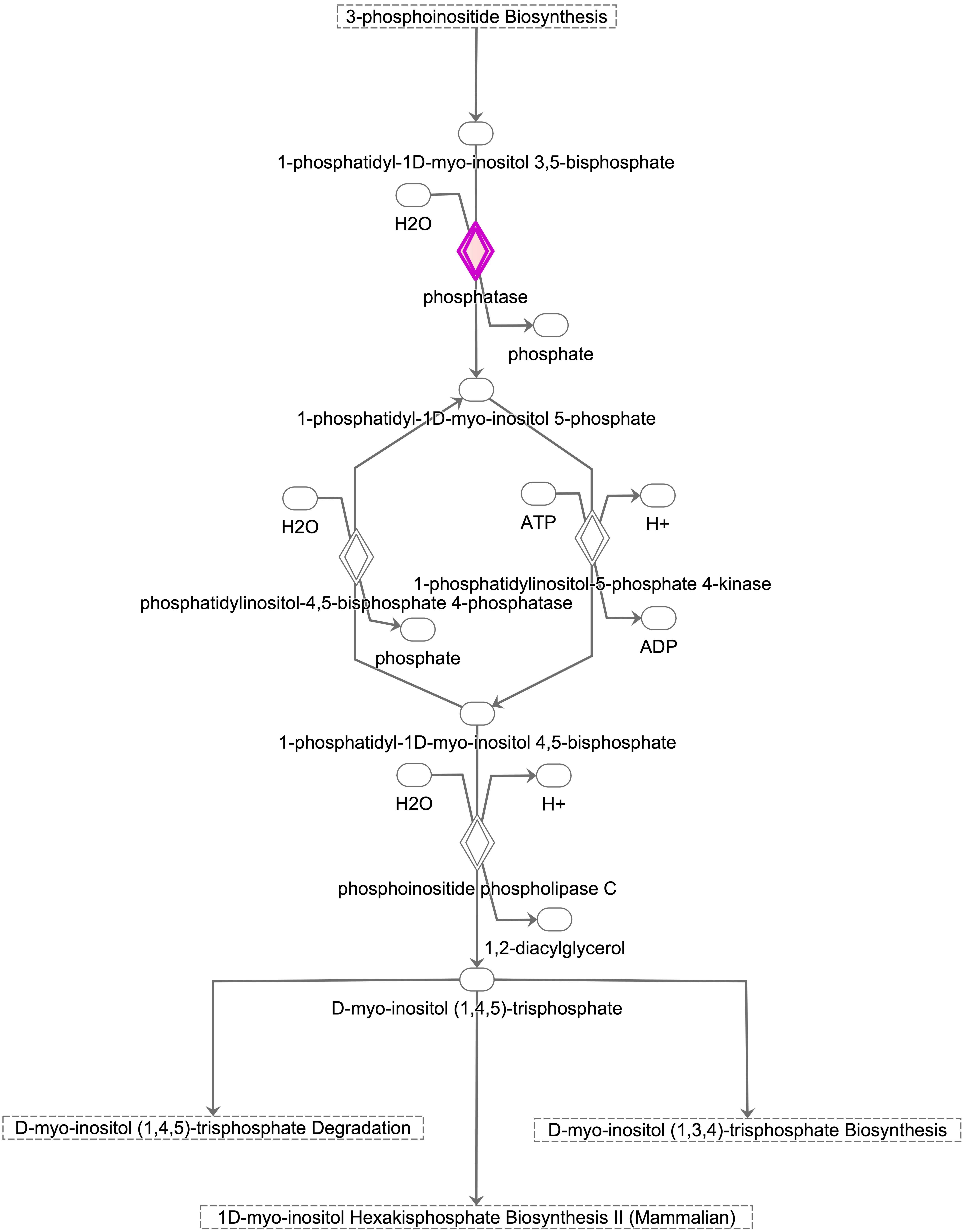
3-phosphoinositide Biosynthesis
1-phosphatidyl-1D-myo-inositol 3,5-bisphosphate
H2O
phosphatase
phosphate
1-phosphatidyl-1D-myo-inositol 5-phosphate
H2O
ATP
H+
1-phosphatidylinositol-5-phosphate 4-kinase
phosphatidylinositol-4,5-bisphosphate 4-phosphatase
ADP
phosphate
1-phosphatidyl-1D-myo-inositol 4,5-bisphosphate
H2O
H+
phosphoinositide phospholipase C
1,2-diacylglycerol
D-myo-inositol (1,4,5)-trisphosphate
D-myo-inositol (1,4,5)-trisphosphate Degradation
D-myo-inositol (1,3,4)-trisphosphate Biosynthesis
1D-myo-inositol Hexakisphosphate Biosynthesis II (Mammalian)

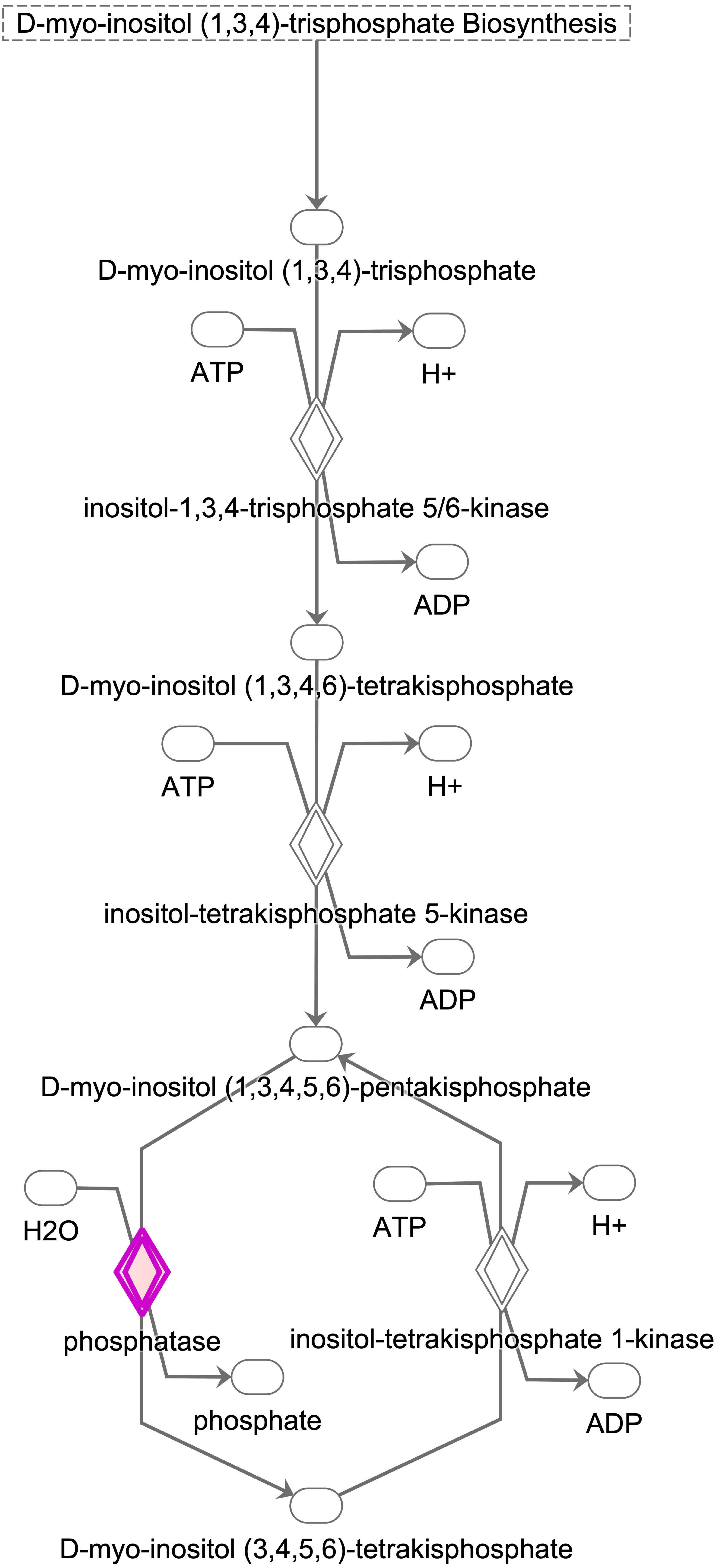

D-myo-inositol (1,3,4)-trisphosphate Biosynthesis
D-myo-inositol (1,3,4)-trisphosphate
ATP
H+
inositol-1,3,4-trisphosphate 5/6-kinase
ADP
D-myo-inositol (1,3,4,6)-tetrakisphosphate
ATP
H+
inositol-tetrakisphosphate 5-kinase
ADP
D-myo-inositol (1,3,4,5,6)-pentakisphosphate
H2O
ATP
H+
phosphatase
inositol-tetrakisphosphate 1-kinase
phosphate
ADP
D-myo-inositol (3,4,5,6)-tetrakisphosphate

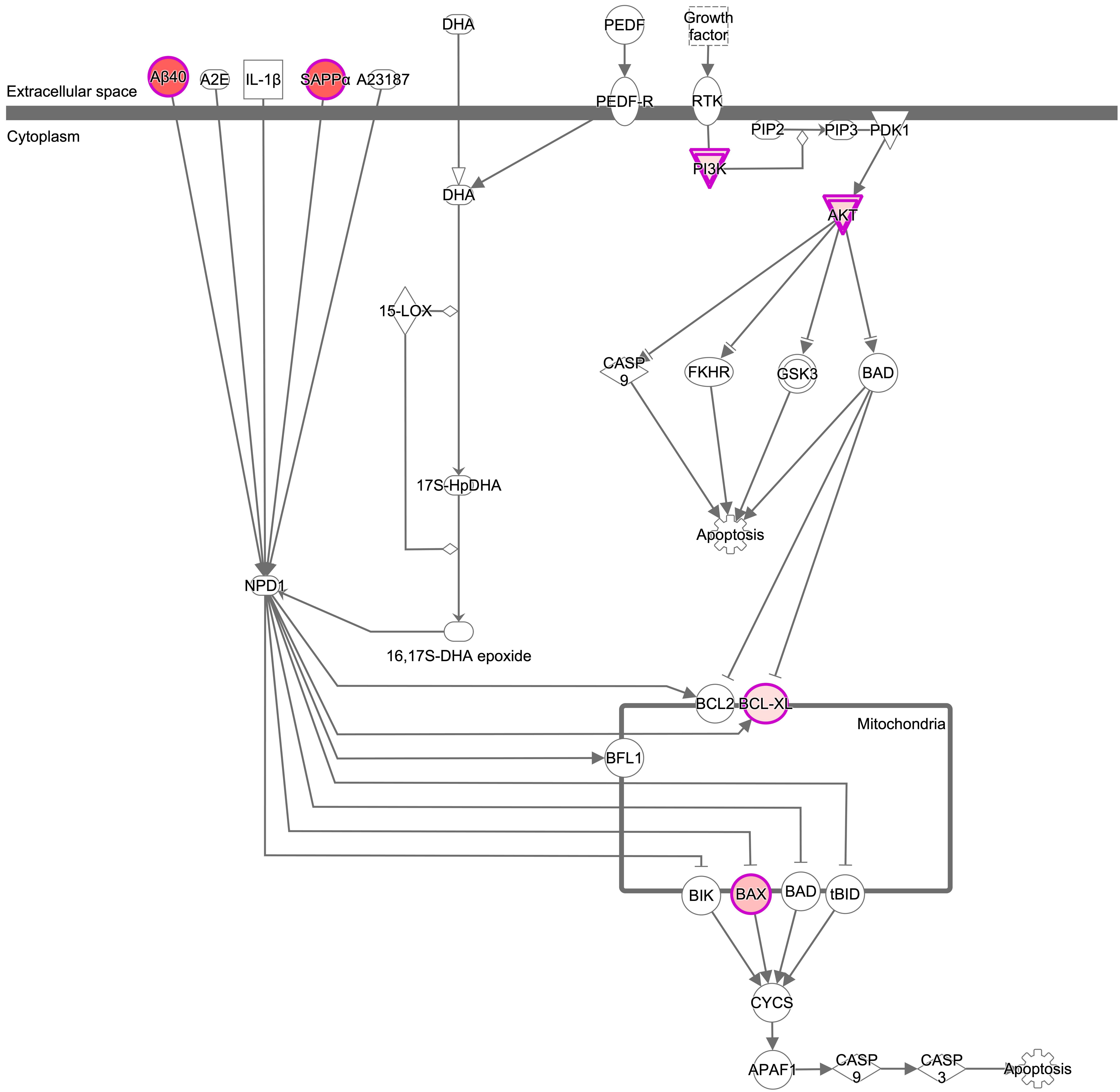

Extracellular space
Cytoplasm
Aβ40
A2E
IL-1β
SAPPα
A23187
DHA
PEDF
Growth factor
PEDF-R
RTK
PIP2
PIP3
PDK1
PI3K
DHA
AKT
15-LOX
CASP 9
FKHR
GSK3
BAD
17S-HpDHA
Apoptosis
NPD1
16,17S-DHA epoxide
BCL2
BCL-XL
Mitochondria
BFL1
BIK
BAX
BAD
tBID
CYCS
APAF1
CASP 9
CASP 3
Apoptosis

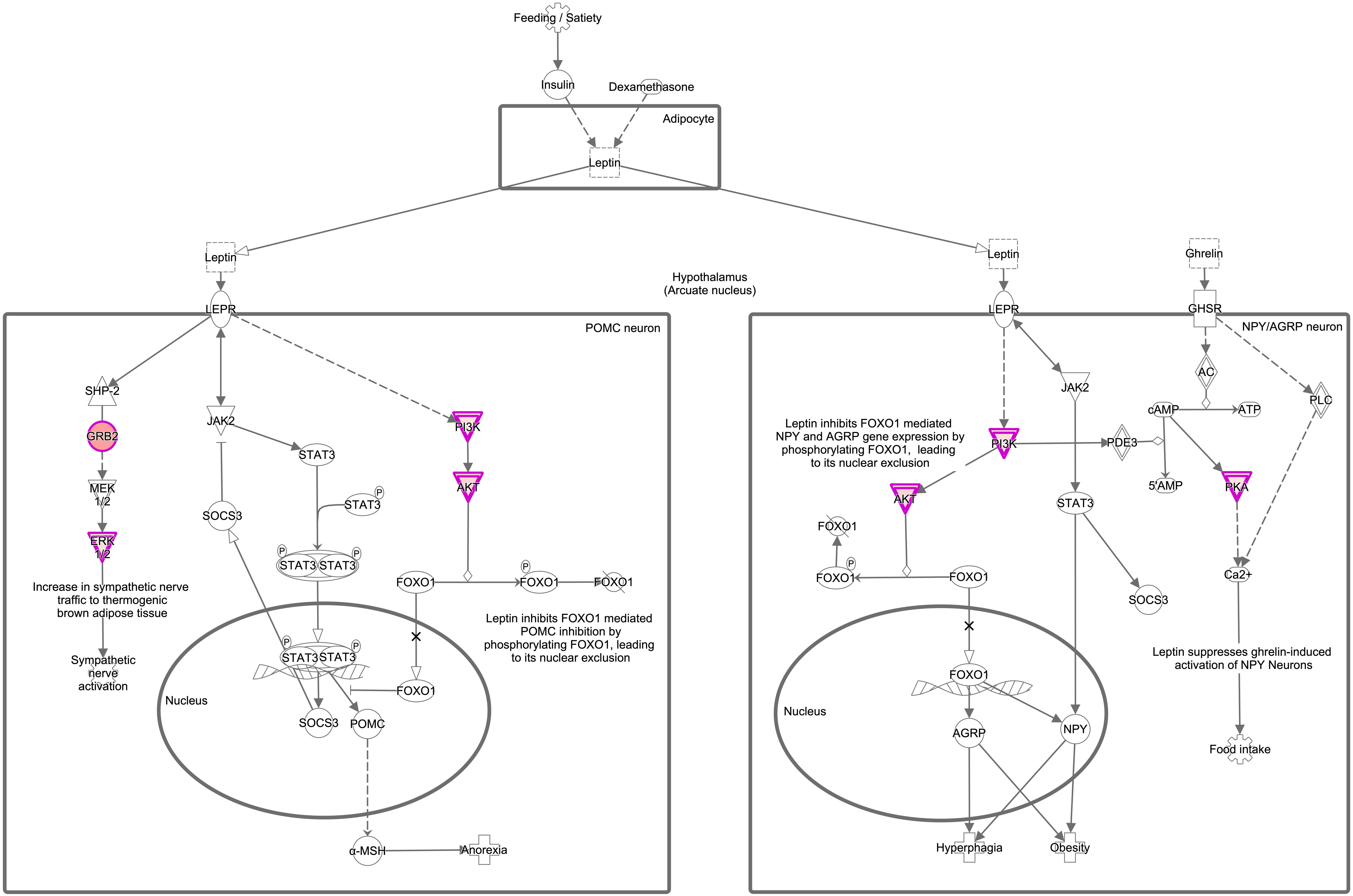
Feeding / Satiety
Insulin
Dexamethasone
Adipocyte
Leptin
Leptin
Hypothalamus
(Arcuate nucleus)
Leptin
Ghrelin
LEPR
POMC neuron
LEPR
GHSR
NPY/AGRP neuron
SHP-2
GRB2
MEK 1/2
ERK 1/2
Increase in sympathetic nerve traffic to thermogenic brown adipose tissue
Sympathetic nerve activation
JAK2
STAT3
STAT3
SOCS3
STAT3 STAT3
PI3K
AKT
FOXO1
FOXO1
FOXO1
Leptin inhibits FOXO1 mediated POMC inhibition by phosphorylating FOXO1, leading to its nuclear exclusion
STAT3 STAT3
FOXO1
Nucleus
SOCS3
POMC
α-MSH
Anorexia
AC
cAMP
ATP
PLC
JAK2
Leptin inhibits FOXO1 mediated NPY and AGRP gene expression by phosphorylating FOXO1, leading to its nuclear exclusion
PI3K
PDE3
5'AMP
PKA
AKT
STAT3
FOXO1
FOXO1
FOXO1
Ca2+
SOCS3
Leptin suppresses ghrelin-induced activation of NPY Neurons
FOXO1
Nucleus
AGRP
NPY
Food intake
Hyperphagia
Obesity

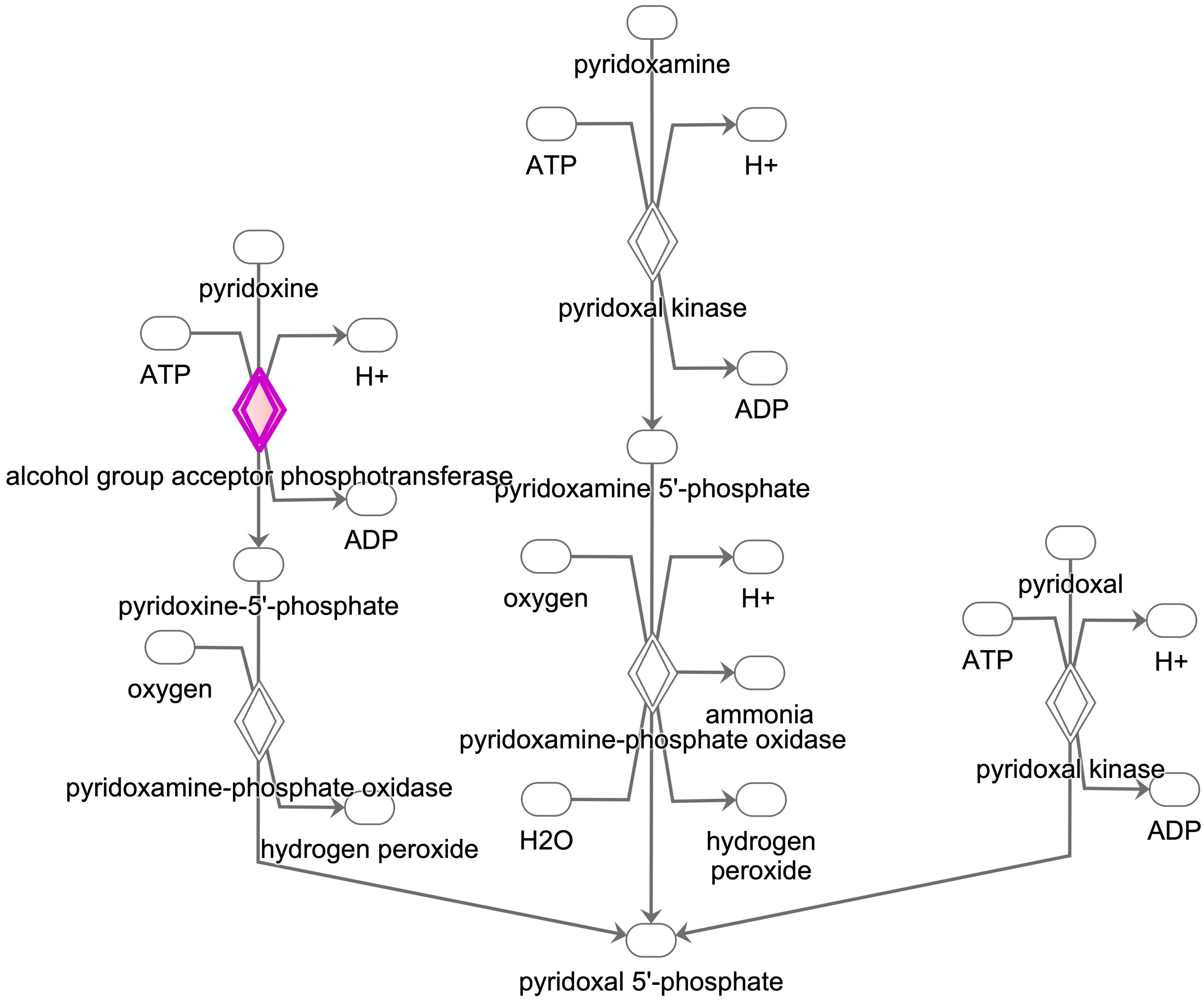
pyridoxamine
ATP
H+
pyridoxal kinase
ADP
pyridoxamine 5'-phosphate
pyridoxine
ATP
H+
alcohol group acceptor phosphotransferase
ADP
pyridoxine-5'-phosphate
oxygen
pyridoxamine-phosphate oxidase
hydrogen peroxide
oxygen
H+
ammonia
pyridoxamine-phosphate oxidase
H2O
hydrogen peroxide
pyridoxal
ATP
H+
pyridoxal kinase
ADP
pyridoxal 5'-phosphate

Beige cells contain fat droplets, are rich in mitochondria, and express high levels of UCP1, which allows protons to cross the inner mitochondrial membrane unproductively, resulting heat generation. Browning of white adipose tissue (WAT) can be activated through cold exposure, sympathetic nervous system activation, exercise, and nutritional factors.

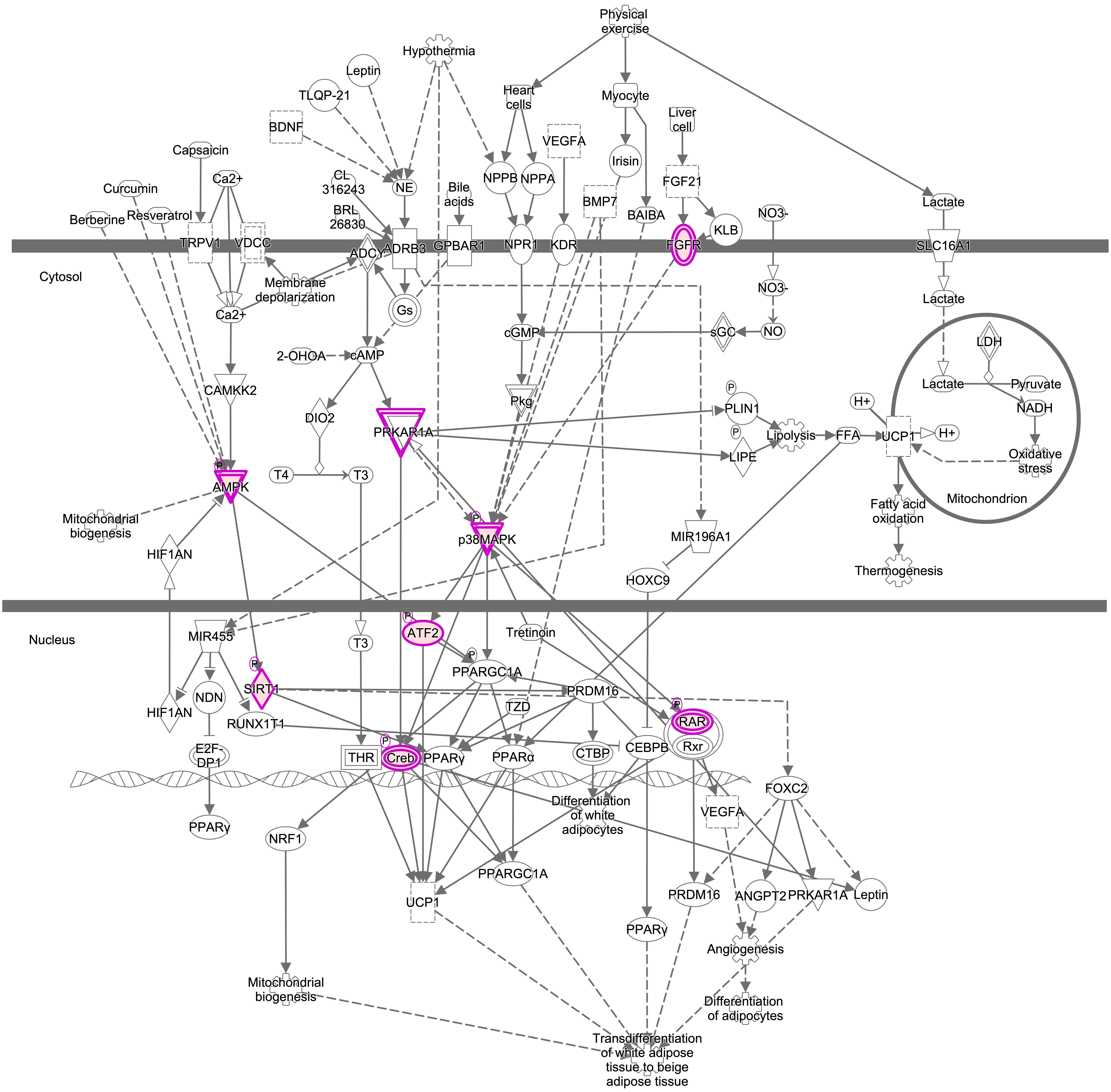

Supplement: Supplementary file 1 [file Supplementary figure 1- A complete list of all cannonical pathways involved in growth factor signaling and lipid homeostasis.pdf › Supplementary figure 1- A complete list of all cannonical pathways involved in growth factor signaling and lipid homeostasis.pdf]
