# Supplementary material for: Mutations and Protein Interaction Landscape Reveal Key Cellular Events Perturbed in Upper Motor Neurons with HSP and PLS
Source: Brain Sci. 2021 Apr 29;11(5):578. doi: 10.3390/brainsci11050578 (PMC8146506; doi:10.3390/brainsci11050578)
Supplement: Supplementary file 1 [file brainsci-11-00578-s001.zip › Supplementary Table 1-HSP and PLS genes with references .pdf]

**HSP CAUSATIVE GENES**
ALS2 (Alsin)[1,2], ***ALDH18A1*** (Aldehyde dehydrogenase 18 family member A1)[3,4], ***AP4B1*** (Adaptor Related Protein Complex 4 Subunit Beta 1)[5], ***AP4E1*** (Adaptor Related Protein Complex 4 Subunit Epsilon 1)[5,6], ***AP4M1***(Adaptor Related Protein Complex 4 Subunit Mu 1)[7], ***AP4S1*** (Adaptor Related Protein Complex 4 Subunit Sigma 1)[8], ***AP5Z1*** (Adaptor Related Protein Complex 5 Subunit Zeta 1)[9], ***ARL6IP1*** (ADP Ribosylation Factor Like GTPase 6 Interacting Protein 1)[1], ***ATAD3A*** (ATPase Family AAA Domain Containing 3A)[10], ***ATL1***(Atlastin GTPase 1)[11], ***B4GALNT1*** (Beta-1,4-N-Acetyl-Galactosaminyltransferase 1)[12], ***BICD2*** (BICD Cargo Adaptor 2)[13], ***BSCL2*** (BSCL2 Lipid Droplet Biogenesis Associated, Seipin)[14], ***C19orf12*** (Chromosome 19 Open Reading Frame 12)[15], ***CAPN1***(Calpain 1)[16], ***CCT5*** (Chaperonin Containing TCP1 Subunit 5)[17], ***CYP2U1*** (Cytochrome P450 Family 2 Subfamily U Member 1)[18], ***CYP7B1*** (Cytochrome P450 Family 7 Subfamily B Member 1)[19], ***DDHD1*** (DDHD Domain Containing 1)[20,21], ***DDHD2*** (DDHD Domain Containing 2)[18], ***ERLIN2*** (ER Lipid Raft Associated 2)[22], ***ENTPD1*** (Ectonucleoside Triphosphate Diphosphohydrolase 1)[1], ***EPT1*** (Ethanolaminephosphotransferase 1)[23], ***ERLIN1*** (ER Lipid Raft Associated 1)[1], ***FARS2*** (Phenylalanyl-TRNA Synthetase 2, Mitochondrial)[24], ***FA2H*** (Fatty Acid 2-Hydroxylase)[25], ***GBA2*** (Glucosylceramidase Beta 2)[18], ***IBA57*** (Iron-Sulfur Cluster Assembly Factor IBA57)[26], ***KCNA2*** (Potassium Voltage-Gated Channel Subfamily A Member 2)[27], ***KIAA0196/WASHC5*** (WASH Complex Subunit 5)[28], ***KIF1A*** (Kinesin Family Member 1A)[29], ***KIF5A*** (Kinesin Family Member 5A)[30], ***KY*** (Kyphoscoliosis Peptidase)[31], ***L1CAM*** (L1 Cell Adhesion Molecule)[32,33], ***LYST*** (Lysosomal Trafficking Regulator)[34], ***NIPA1*** (NIPA Magnesium Transporter 1)[35,36], ***NT5C2*** (5'-Nucleotidase, Cytosolic II)[1], ***PCYT2*** (Phosphate Cytidylyltransferase 2, Ethanolamine)[37], ***PLP1*** (Proteolipid Protein 1)[38], ***PNPLA6*** (Patatin Like Phospholipase Domain Containing 6)[39,40], ***RAB3GAP2*** (RAB3 GTPase Activating Non-Catalytic Protein Subunit 2)[41,42], ***REEP1*** (Receptor Accessory Protein 1)[43], ***REEP2*** (Receptor Accessory Protein 2)[44], ***RTN2*** (Reticulon 2)[45], ***SETX*** (Senataxin)[46],***SLC33A1*** (Solute Carrier Family 33 Member 1)[47], ***SPAST*** (Spastin)[48], ***SPG11*** (SPG11 Vesicle Trafficking Associated, Spatacsin)[49], ***SPG21/ACP33*** (SPG21 Abhydrolase Domain Containing, Maspardin)[50], ***SPG7*** (SPG7 Matrix AAA Peptidase Subunit, Paraplegin)[51], ***SPG80/UBAP1*** (Ubiquitin Associated Protein 1)[52], ***TECPR2*** (Tectonin Beta-Propeller Repeat Containing 2)[53], ***TFG*** (Trafficking From ER To Golgi Regulator)[54], ***TUBB4A*** (Tubulin Beta 4A Class IVa)[55], ***VCP*** (Valosin Containing Protein)[56], ***VPS37A*** (VPS37A Subunit Of ESCRT-I)[57], ***ZFYVE26*** (Zinc Finger FYVE-Type Containing 26)[58,59], ***ZFYVE27*** (Zinc Finger FYVE-Type Containing 27)[60]
**HSP ASSOCIATED GENES**
ABCD1 (ATP Binding Cassette Subfamily D Member 1)[61], ***ADAR*** (Adenosine Deaminase RNA Specific)[62], ***ALDH3A2*** (Aldehyde Dehydrogenase 3 Family Member A2)[63], ***AMPD2*** (Adenosine Monophosphate Deaminase 2)[1], ***ARSA*** (Arylsulfatase A)[64], ***ARSI*** (Arylsulfatase I precursor)[1], ***ATP13A2*** (ATPase Cation Transporting 13A2)[65], ***CPT1C*** (Carnitine Palmitoyltransferase 1C)[66], ***ELOVL1*** (ELOVL Fatty Acid Elongase 1)[64], ***FLRT1*** (Fibronectin Leucine Rich Transmembrane Protein 1)[1], ***GALC*** (Galactosylceramidase)[64], ***GCH1*** (GTP Cyclohydrolase 1)[67], ***GRN*** (Granulin Precursor)[68], ***HSPD1/ HSP60*** (Heat Shock Protein Family D (Hsp60) Member 1)[69,70], ***IFIH1*** (Interferon Induced With Helicase C Domain 1)[62], ***KIF1C*** (Kinesin Family Member 1C)[71], ***MAG*** (Myelin-associated glycoprotein)[1,72], ***MARS*** (Methionyl-TRNA Synthetase)[1], ***MFN2*** (Mitofusin-2)[73], ***MT-ATP6*** (Mitochondrially Encoded ATP Synthase Membrane Subunit 6)[74], ***OPA1*** (Dynamin-like 120 kDa protein)[73], ***OPA3*** (Outer Mitochondrial Membrane Lipid Metabolism Regulator OPA3)[75], ***PGAP1*** (Post-GPI Attachment To Proteins Inositol Deacylase 1)[1], ***PLA2G6*** (Phospholipase A2 Group VI)[76], ***POLR3A*** (RNA Polymerase III Subunit A)[77], ***SERAC1*** (Serine Active Site Containing 1)[64], ***SLC16A2*** (Solute Carrier Family 16 Member 2)[78,79], ***SPART*** (Spartin)[80], ***SPG20*** (Troyer syndrome)[63], ***TRPV4*** (Transient Receptor Potential Cation Channel Subfamily V Member 4)[81], ***USP8*** (Ubiquitin Specific Peptidase 8)[1], ***WDR48*** (WD Repeat Domain 48)[1], ***ZFR*** (Zinc Finger RNA Binding Protein)[1]
**HSP LINKED GENES**
C12orf65 (Probable peptide chain release factor C12orf65, mitochondrial)[82], ***CYP27A1*** (Cytochrome P450 Family 27 Subfamily A Member 1)[64], ***GAD1*** (Glutamate Decarboxylase 1)[83,84], ***GJC2*** (Gap Junction Protein Gamma 2)[85], ***KLC2*** (Kinesin Light Chain 2)[82], ***RIPK5/DSTYK*** (Dual serine/threonine and tyrosine protein kinase)[86], ***UCHL1*** (Ubiquitin C-Terminal Hydrolase L1)[87]
**PLS GENES**
CAUSATIVE: ***ALS2***[2,88] ASSOCIATED: ***ALS15*** (Ubiquilin 2)[89], ***C9orf72*** (C9orf72-SMCR8 Complex Subunit)[90], ***DCTN1*** (Dynactin Subunit 1)[90,91], ***ERLIN2*** (ER Lipid Raft Associated 2)[92], ***PARK2*** (Parkin)[90,93] LINKED: ***FIG4*** (FIG4 Phosphoinositide 5-Phosphatase)[94], ***SPG7*** (SPG7 Matrix AAA Peptidase Subunit, Paraplegin)[95]

1. Novarino, G.; Fenstermaker, A.G.; Zaki, M.S.; Hofree, M.; Silhavy, J.L.; Heiberg, A.D.; Abdellateef, M.; Rosti, B.; Scott, E.; Mansour, L.; et al. Exome sequencing links corticospinal motor neuron disease to common neurodegenerative disorders. Science 2014, 343, 506-511, doi:10.1126/science.1247363.
2. Wakil, S.M.; Ramzan, K.; Abuthuraya, R.; Hagos, S.; Al-Dossari, H.; Al-Omar, R.; Murad, H.; Chedrawi, A.; Al-Hassnan, Z.N.; Finsterer, J.; et al. Infantile-onset ascending hereditary spastic paraplegia with bulbar involvement due to the novel ALS2 mutation c.2761C>T. Gene 2014, 536, 217-220, doi:10.1016/j.gene.2013.11.043.
3. Coutelier, M.; Goizet, C.; Durr, A.; Habarou, F.; Morais, S.; Dionne-Laporte, A.; Tao, F.; Konop, J.; Stoll, M.; Charles, P.; et al. Alteration of ornithine metabolism leads to dominant and recessive hereditary spastic paraplegia. Brain 2015, 138, 2191-2205, doi:10.1093/brain/awv143.

4. Panza, E.; Escamilla-Honrubia, J.M.; Marco-Marin, C.; Gougeard, N.; De Michele, G.; Morra, V.B.; Liguori, R.; Salviati, L.; Donati, M.A.; Cusano, R.; et al. ALDH18A1 gene mutations cause dominant spastic paraplegia SPG9: loss of function effect and plausibility of a dominant negative mechanism. Brain 2016, 139, e3, doi:10.1093/brain/awv247.
5. Bauer, P.; Leshinsky-Silver, E.; Blumkin, L.; Schlipf, N.; Schroder, C.; Schicks, J.; Lev, D.; Riess, O.; Lerman-Sagie, T.; Schols, L. Mutation in the AP4B1 gene cause hereditary spastic paraplegia type 47 (SPG47). Neurogenetics 2012, 13, 73-76, doi:10.1007/s10048-012-0314-0.
6. Kong, X.F.; Bousfiha, A.; Rouissi, A.; Itan, Y.; Abhyankar, A.; Bryant, V.; Okada, S.; Ailal, F.; Bustamante, J.; Casanova, J.L.; et al. A novel homozygous p.R1105X mutation of the AP4E1 gene in twins with hereditary spastic paraplegia and mycobacterial disease. PLoS One 2013, 8, e58286, doi:10.1371/journal.pone.0058286.
7. Abou Jamra, R.; Philippe, O.; Raas-Rothschild, A.; Eck, S.H.; Graf, E.; Buchert, R.; Borck, G.; Ekici, A.; Brockschmidt, F.F.; Nothen, M.M.; et al. Adaptor protein complex 4 deficiency causes severe autosomal-recessive intellectual disability, progressive spastic paraplegia, shy character, and short stature. Am J Hum Genet 2011, 88, 788-795, doi:10.1016/j.ajhg.2011.04.019.
8. Fink, J.K. Hereditary spastic paraplegia: clinico-pathologic features and emerging molecular mechanisms. Acta Neuropathol 2013, 126, 307-328, doi:10.1007/s00401-013-1115-8.
9. Schlipf, N.A.; Schule, R.; Klimpe, S.; Karle, K.N.; Synofzik, M.; Wolf, J.; Riess, O.; Schols, L.; Bauer, P. AP5Z1/SPG48 frequency in autosomal recessive and sporadic spastic paraplegia. Mol Genet Genomic Med 2014, 2, 379-382, doi:10.1002/mgg3.87.
10. Cooper, H.M.; Yang, Y.; Ylikallio, E.; Khairullin, R.; Woldegebriel, R.; Lin, K.L.; Euro, L.; Palin, E.; Wolf, A.; Trokovic, R.; et al. ATPase-deficient mitochondrial inner membrane protein ATAD3A disturbs mitochondrial dynamics in dominant hereditary spastic paraplegia. Hum Mol Genet 2017, 26, 1432-1443, doi:10.1093/hmg/ddx042.
11. Meszarosova, A.U.; Grecmalova, D.; Brazdilova, M.; Dvorackova, N.; Kalina, Z.; Cermakova, M.; Vavrova, D.; Smetanova, I.; Stanek, D.; Seeman, P. Disease-Causing Variants in the ATL1 Gene Are a Rare Cause of Hereditary Spastic Paraplegia among Czech Patients. Ann Hum Genet 2017, 81, 249-257, doi:10.1111/ahg.12206.
12. Boukhris, A.; Schule, R.; Loureiro, J.L.; Lourenco, C.M.; Mundwiller, E.; Gonzalez, M.A.; Charles, P.; Gauthier, J.; Rekik, I.; Acosta Lebrigio, R.F.; et al. Alteration of ganglioside biosynthesis responsible for complex hereditary spastic paraplegia. Am J Hum Genet 2013, 93, 118-123, doi:10.1016/j.ajhg.2013.05.006.
13. Oates, E.C.; Rossor, A.M.; Hafezparast, M.; Gonzalez, M.; Speziani, F.; MacArthur, D.G.; Lek, M.; Cottenie, E.; Scoto, M.; Foley, A.R.; et al. Mutations in BICD2 cause dominant congenital spinal muscular atrophy and hereditary spastic paraplegia. Am J Hum Genet 2013, 92, 965-973, doi:10.1016/j.ajhg.2013.04.018.
14. Windpassinger, C.; Auer-Grumbach, M.; Irobi, J.; Patel, H.; Petek, E.; Horl, G.; Malli, R.; Reed, J.A.; Dierick, I.; Verpoorten, N.; et al. Heterozygous missense mutations in BSCL2 are associated with distal hereditary motor neuropathy and Silver syndrome. Nat Genet 2004, 36, 271-276, doi:10.1038/ng1313.
15. Landoure, G.; Zhu, P.P.; Lourenco, C.M.; Johnson, J.O.; Toro, C.; Bricceno, K.V.; Rinaldi, C.; Meilleur, K.G.; Sangare, M.; Diallo, O.; et al. Hereditary spastic paraplegia type 43 (SPG43) is caused by mutation in C19orf12. Hum Mutat 2013, 34, 1357-1360, doi:10.1002/humu.22378.
16. Gan-Or, Z.; Bouslam, N.; Birouk, N.; Lissouba, A.; Chambers, D.B.; Veriepe, J.; Androschuk, A.; Laurent, S.B.; Rochefort, D.; Spiegelman, D.; et al. Mutations in CAPN1 Cause Autosomal-Recessive Hereditary Spastic Paraplegia. Am J Hum Genet 2016, 98, 1038-1046, doi:10.1016/j.ajhg.2016.04.002.
17. Bouhouche, A.; Benomar, A.; Bouslam, N.; Chkili, T.; Yahyaoui, M. Mutation in the epsilon subunit of the cytosolic chaperonin-containing t-complex peptide-1 (Cct5) gene causes autosomal recessive mutilating sensory neuropathy with spastic paraplegia. J Med Genet 2006, 43, 441-443, doi:10.1136/jmg.2005.039230.
18. Citterio, A.; Arnoldi, A.; Panzeri, E.; D'Angelo, M.G.; Filosto, M.; Dilena, R.; Arrigoni, F.; Castelli, M.; Maghini, C.; Germiniasi, C.; et al. Mutations in CYP2U1, DDHD2 and GBA2 genes are rare causes of complicated forms of hereditary spastic paraparesis. J Neurol 2014, 261, 373-381, doi:10.1007/s00415-013-7206-6.
19. Lynch, D.S.; Koutsis, G.; Tucci, A.; Panas, M.; Baklou, M.; Breza, M.; Karadima, G.; Houlden, H. Hereditary spastic paraplegia in Greece: characterisation of a previously unexplored population using next-generation sequencing. Eur J Hum Genet 2016, 24, 857-863, doi:10.1038/ejhg.2015.200.
20. Maruyama, T.; Baba, T.; Maemoto, Y.; Hara-Miyauchi, C.; Hasegawa-Ogawa, M.; Okano, H.J.; Enda, Y.; Matsumoto, K.; Arimitsu, N.; Nakao, K.; et al. Loss of DDHD2, whose mutation causes spastic paraplegia, promotes reactive oxygen species generation and apoptosis. Cell Death Dis 2018, 9, 797, doi:10.1038/s41419-018-0815-3.
21. Tesson, C.; Nawara, M.; Salih, M.A.; Rossignol, R.; Zaki, M.S.; Al Balwi, M.; Schule, R.; Mignot, C.; Obre, E.; Bouhouche, A.; et al. Alteration of fatty-acid-metabolizing enzymes affects mitochondrial form and function in hereditary spastic paraplegia. Am J Hum Genet 2012, 91, 1051-1064, doi:10.1016/j.ajhg.2012.11.001.
22. Alazami, A.M.; Adly, N.; Al Dhalaan, H.; Alkuraya, F.S. A nullimorphic ERLIN2 mutation defines a complicated hereditary spastic paraplegia locus (SPG18). Neurogenetics 2011, 12, 333-336, doi:10.1007/s10048-011-0291-8.
23. Ahmed, M.Y.; Al-Khayat, A.; Al-Murshedi, F.; Al-Futaisi, A.; Chioza, B.A.; Pedro Fernandez-Murray, J.; Self, J.E.; Salter, C.G.; Harlalka, G.V.; Rawlins, L.E.; et al. A mutation of EPT1 (SELENOI) underlies a new disorder of Kennedy pathway phospholipid biosynthesis. Brain 2017, 140, 547-554, doi:10.1093/brain/aww318.

24. Yang, Y.; Liu, W.; Fang, Z.; Shi, J.; Che, F.; He, C.; Yao, L.; Wang, E.; Wu, Y. A Newly Identified Missense Mutation in FARS2 Causes Autosomal-Recessive Spastic Paraplegia. Hum Mutat 2016, 37, 165-169, doi:10.1002/humu.22930.
25. Dick, K.J.; Eckhardt, M.; Paisan-Ruiz, C.; Alshehhi, A.A.; Proukakis, C.; Sibtain, N.A.; Maier, H.; Sharifi, R.; Patton, M.A.; Bashir, W.; et al. Mutation of FA2H underlies a complicated form of hereditary spastic paraplegia (SPG35). Hum Mutat 2010, 31, E1251-1260, doi:10.1002/humu.21205.
26. Lossos, A.; Stumpfig, C.; Stevanin, G.; Gaussen, M.; Zimmerman, B.E.; Mundwiller, E.; Asulin, M.; Chamma, L.; Sheffer, R.; Misk, A.; et al. Fe/S protein assembly gene IBA57 mutation causes hereditary spastic paraplegia. Neurology 2015, 84, 659-667, doi:10.1212/WNL.0000000000001270.
27. Helbig, K.L.; Hedrich, U.B.; Shinde, D.N.; Krey, I.; Teichmann, A.C.; Hentschel, J.; Schubert, J.; Chamberlin, A.C.; Huether, R.; Lu, H.M.; et al. A recurrent mutation in KCNA2 as a novel cause of hereditary spastic paraplegia and ataxia. Ann Neurol 2016, 80, doi:10.1002/ana.24762.
28. Valdmanis, P.N.; Meijer, I.A.; Reynolds, A.; Lei, A.; MacLeod, P.; Schlesinger, D.; Zatz, M.; Reid, E.; Dion, P.A.; Drapeau, P.; et al. Mutations in the KIAA0196 gene at the SPG8 locus cause hereditary spastic paraplegia. Am J Hum Genet 2007, 80, 152-161, doi:10.1086/510782.
29. Erlich, Y.; Edvardson, S.; Hodges, E.; Zenvirt, S.; Thekkat, P.; Shaag, A.; Dor, T.; Hannon, G.J.; Elpeleg, O. Exome sequencing and disease-network analysis of a single family implicate a mutation in KIF1A in hereditary spastic paraparesis. Genome Res 2011, 21, 658-664, doi:10.1101/gr.117143.110.
30. Fichera, M.; Lo Giudice, M.; Falco, M.; Sturnio, M.; Amata, S.; Calabrese, O.; Bigoni, S.; Calzolari, E.; Neri, M. Evidence of kinesin heavy chain (KIF5A) involvement in pure hereditary spastic paraplegia. Neurology 2004, 63, 1108-1110, doi:10.1212/01.wnl.0000138731.60693.d2.
31. Yogev, Y.; Perez, Y.; Noyman, I.; Madegem, A.A.; Flusser, H.; Shorer, Z.; Cohen, E.; Kachko, L.; Michaelovsky, A.; Birk, R.; et al. Progressive hereditary spastic paraplegia caused by a homozygous KY mutation. Eur J Hum Genet 2017, 25, 966-972, doi:10.1038/ejhg.2017.85.
32. Jouet, M.; Rosenthal, A.; Armstrong, G.; MacFarlane, J.; Stevenson, R.; Paterson, J.; Metzenberg, A.; Ionasescu, V.; Temple, K.; Kenwrick, S. X-linked spastic paraplegia (SPG1), MASA syndrome and X-linked hydrocephalus result from mutations in the L1 gene. Nat Genet 1994, 7, 402-407, doi:10.1038/ng0794-402.
33. Munoz, A.; Cabrera-Lopez, J.C.; Santana-Rodriguez, A.; Toledo-Bravo de Laguna, L.; Santana-Artiles, A.; Sebastian-Garcia, I. [X-linked hereditary spastic paraplegia due to mutation in the L1CAM gene: three cases reports of CRASH syndrome]. Rev Neurol 2016, 62, 218-222.
34. Shimazaki, H.; Honda, J.; Naoi, T.; Namekawa, M.; Nakano, I.; Yazaki, M.; Nakamura, K.; Yoshida, K.; Ikeda, S.; Ishiura, H.; et al. Autosomal-recessive complicated spastic paraplegia with a novel lysosomal trafficking regulator gene mutation. J Neurol Neurosurg Psychiatry 2014, 85, 1024-1028, doi:10.1136/jnnp-2013-306981.
35. Svenstrup, K.; Moller, R.S.; Christensen, J.; Budtz-Jorgensen, E.; Gilling, M.; Nielsen, J.E. NIPA1 mutation in complex hereditary spastic paraplegia with epilepsy. Eur J Neurol 2011, 18, 1197-1199, doi:10.1111/j.1468-1331.2011.03359.x.
36. Martinez-Lage, M.; Molina-Porcel, L.; Falcone, D.; McCluskey, L.; Lee, V.M.; Van Deerlin, V.M.; Trojanowski, J.Q. TDP-43 pathology in a case of hereditary spastic paraplegia with a NIPA1/SPG6 mutation. Acta Neuropathol 2012, 124, 285-291, doi:10.1007/s00401-012-0947-y.
37. Vaz, F.M.; McDermott, J.H.; Alders, M.; Wortmann, S.B.; Kolker, S.; Pras-Raves, M.L.; Vervaart, M.A.T.; van Lenthe, H.; Luyf, A.C.M.; Elfrink, H.L.; et al. Mutations in PCYT2 disrupt etherlipid biosynthesis and cause a complex hereditary spastic paraplegia. Brain 2019, 142, 3382-3397, doi:10.1093/brain/awz291.
38. Cambi, F.; Tang, X.M.; Cordray, P.; Fain, P.R.; Keppen, L.D.; Barker, D.F. Refined genetic mapping and proteolipid protein mutation analysis in X-linked pure hereditary spastic paraplegia. Neurology 1996, 46, 1112-1117, doi:10.1212/wnl.46.4.1112.
39. Rainier, S.; Bui, M.; Mark, E.; Thomas, D.; Tokarz, D.; Ming, L.; Delaney, C.; Richardson, R.J.; Albers, J.W.; Matsunami, N.; et al. Neuropathy target esterase gene mutations cause motor neuron disease. Am J Hum Genet 2008, 82, 780-785, doi:10.1016/j.ajhg.2007.12.018.
40. Schabhuttl, M.; Wieland, T.; Senderek, J.; Baets, J.; Timmerman, V.; De Jonghe, P.; Reilly, M.M.; Stieglbauer, K.; Laich, E.; Windhager, R.; et al. Whole-exome sequencing in patients with inherited neuropathies: outcome and challenges. J Neurol 2014, 261, 970-982, doi:10.1007/s00415-014-7289-8.
41. Tinti, M.; Johnson, C.; Toth, R.; Ferrier, D.E.; Mackintosh, C. Evolution of signal multiplexing by 14-3-3-binding 2R-ohnologue protein families in the vertebrates. Open Biol 2012, 2, 120103, doi:10.1098/rsob.120103.
42. Tesson, C.; Koht, J.; Stevanin, G. Delving into the complexity of hereditary spastic paraplegias: how unexpected phenotypes and inheritance modes are revolutionizing their nosology. Hum Genet 2015, 134, 511-538, doi:10.1007/s00439-015-1536-7.
43. Beetz, C.; Schule, R.; Deconinck, T.; Tran-Viet, K.N.; Zhu, H.; Kremer, B.P.; Frints, S.G.; van Zelst-Stams, W.A.; Byrne, P.; Otto, S.; et al. REEP1 mutation spectrum and genotype/phenotype correlation in hereditary spastic paraplegia type 31. Brain 2008, 131, 1078-1086, doi:10.1093/brain/awn026.
44. Esteves, T.; Durr, A.; Mundwiller, E.; Loureiro, J.L.; Boutry, M.; Gonzalez, M.A.; Gauthier, J.; El-Hachimi, K.H.; Depienne, C.; Muriel, M.P.; et al. Loss of association of REEP2 with membranes leads to hereditary spastic paraplegia. Am J Hum Genet 2014, 94, 268-277, doi:10.1016/j.ajhg.2013.12.005.

45. Montenegro, G.; Rebelo, A.P.; Connell, J.; Allison, R.; Babalini, C.; D'Aloia, M.; Montieri, P.; Schule, R.; Ishiura, H.; Price, J.; et al. Mutations in the ER-shaping protein reticulon 2 cause the axon-degenerative disorder hereditary spastic paraplegia type 12. J Clin Invest 2012, 122, 538-544, doi:10.1172/JCI60560.
46. Elert-Dobkowska, E.; Stepniak, I.; Krysa, W.; Ziora-Jakutowicz, K.; Rakowicz, M.; Sobanska, A.; Pilch, J.; Antczak-Marach, D.; Zaremba, J.; Sulek, A. Next-generation sequencing study reveals the broader variant spectrum of hereditary spastic paraplegia and related phenotypes. Neurogenetics 2019, 20, 27-38, doi:10.1007/s10048-019-00565-6.
47. Lin, P.; Li, J.; Liu, Q.; Mao, F.; Li, J.; Qiu, R.; Hu, H.; Song, Y.; Yang, Y.; Gao, G.; et al. A missense mutation in SLC33A1, which encodes the acetyl-CoA transporter, causes autosomal-dominant spastic paraplegia (SPG42). Am J Hum Genet 2008, 83, 752-759, doi:10.1016/j.ajhg.2008.11.003.
48. Charvin, D.; Cifuentes-Diaz, C.; Fonknechten, N.; Joshi, V.; Hazan, J.; Melki, J.; Betuing, S. Mutations of SPG4 are responsible for a loss of function of spastin, an abundant neuronal protein localized in the nucleus. Hum Mol Genet 2003, 12, 71-78, doi:10.1093/hmg/ddg004.
49. Stevanin, G.; Azzedine, H.; Denora, P.; Boukhris, A.; Tazir, M.; Lossos, A.; Rosa, A.L.; Lerer, I.; Hamri, A.; Alegria, P.; et al. Mutations in SPG11 are frequent in autosomal recessive spastic paraplegia with thin corpus callosum, cognitive decline and lower motor neuron degeneration. Brain 2008, 131, 772-784, doi:10.1093/brain/awm293.
50. Simpson, M.A.; Cross, H.; Proukakis, C.; Pryde, A.; Hershberger, R.; Chatonnet, A.; Patton, M.A.; Crosby, A.H. Maspardin is mutated in mast syndrome, a complicated form of hereditary spastic paraplegia associated with dementia. Am J Hum Genet 2003, 73, 1147-1156, doi:10.1086/379522.
51. Warnecke, T.; Duning, T.; Schwan, A.; Lohmann, H.; Epplen, J.T.; Young, P. A novel form of autosomal recessive hereditary spastic paraplegia caused by a new SPG7 mutation. Neurology 2007, 69, 368-375, doi:10.1212/01.wnl.0000266667.91074.fe.
52. Nan, H.; Ichinose, Y.; Tanaka, M.; Koh, K.; Ishiura, H.; Mitsui, J.; Mizukami, H.; Morimoto, M.; Hamada, S.; Ohtsuka, T.; et al. UBAP1 mutations cause juvenile-onset hereditary spastic paraplegias (SPG80) and impair UBAP1 targeting to endosomes. J Hum Genet 2019, 64, 1055-1065, doi:10.1038/s10038-019-0670-9.
53. Oz-Levi, D.; Ben-Zeev, B.; Ruzzo, E.K.; Hitomi, Y.; Gelman, A.; Pelak, K.; Anikster, Y.; Reznik-Wolf, H.; Bar-Joseph, I.; Olender, T.; et al. Mutation in TECPR2 reveals a role for autophagy in hereditary spastic paraparesis. Am J Hum Genet 2012, 91, 1065-1072, doi:10.1016/j.ajhg.2012.09.015.
54. Beetz, C.; Johnson, A.; Schuh, A.L.; Thakur, S.; Varga, R.E.; Fothergill, T.; Hertel, N.; Bomba-Warczak, E.; Thiele, H.; Nurnberg, G.; et al. Inhibition of TFG function causes hereditary axon degeneration by impairing endoplasmic reticulum structure. Proc Natl Acad Sci U S A 2013, 110, 5091-5096, doi:10.1073/pnas.1217197110.
55. Vulinovic, F.; Krajka, V.; Hausrat, T.J.; Seibler, P.; Alvarez-Fischer, D.; Madoev, H.; Park, J.S.; Kumar, K.R.; Sue, C.M.; Lohmann, K.; et al. Motor protein binding and mitochondrial transport are altered by pathogenic TUBB4A variants. Hum Mutat 2018, 39, 1901-1915, doi:10.1002/humu.23602.
56. Shih, Y.T.; Hsueh, Y.P. The involvement of endoplasmic reticulum formation and protein synthesis efficiency in VCP- and ATL1-related neurological disorders. J Biomed Sci 2018, 25, 2, doi:10.1186/s12929-017-0403-3.
57. Zivony-Elboum, Y.; Westbroek, W.; Kfir, N.; Savitzki, D.; Shoval, Y.; Bloom, A.; Rod, R.; Khayat, M.; Gross, B.; Samri, W.; et al. A founder mutation in Vps37A causes autosomal recessive complex hereditary spastic paraparesis. J Med Genet 2012, 49, 462-472, doi:10.1136/jmedgenet-2012-100742.
58. Hanein, S.; Martin, E.; Boukhris, A.; Byrne, P.; Goizet, C.; Hamri, A.; Benomar, A.; Lossos, A.; Denora, P.; Fernandez, J.; et al. Identification of the SPG15 gene, encoding spastizin, as a frequent cause of complicated autosomal-recessive spastic paraplegia, including Kjellin syndrome. Am J Hum Genet 2008, 82, 992-1002, doi:10.1016/j.ajhg.2008.03.004.
59. Hughes, C.A.; Byrne, P.C.; Webb, S.; McMonagle, P.; Patterson, V.; Hutchinson, M.; Parfrey, N.A. SPG15, a new locus for autosomal recessive complicated HSP on chromosome 14q. Neurology 2001, 56, 1230-1233, doi:10.1212/wnl.56.9.1230.
60. Mannan, A.U.; Krawen, P.; Sauter, S.M.; Boehm, J.; Chronowska, A.; Paulus, W.; Neesen, J.; Engel, W. ZFYVE27 (SPG33), a novel spastin-binding protein, is mutated in hereditary spastic paraplegia. Am J Hum Genet 2006, 79, 351-357, doi:10.1086/504927.
61. Koutsis, G.; Lynch, D.S.; Tucci, A.; Houlden, H.; Karadima, G.; Panas, M. A novel ABCD1 mutation detected by next generation sequencing in presumed hereditary spastic paraplegia: A 30-year diagnostic delay caused by misleading biochemical findings. J Neurol Sci 2015, 355, 199-201, doi:10.1016/j.jns.2015.05.031.
62. Crow, Y.J.; Zaki, M.S.; Abdel-Hamid, M.S.; Abdel-Salam, G.; Boespflug-Tanguy, O.; Cordeiro, N.J.; Gleeson, J.G.; Gowrinathan, N.R.; Laugel, V.; Renaldo, F.; et al. Mutations in ADAR1, IFIH1, and RNASEH2B presenting as spastic paraplegia. Neuropediatrics 2014, 45, 386-393, doi:10.1055/s-0034-1389161.
63. Hedera, P. Hereditary Spastic Paraplegia Overview. In GeneReviews((R)), Adam, M.P., Ardinger, H.H., Pagon, R.A., Wallace, S.E., Bean, L.J.H., Mirzaa, G., Amemiya, A., Eds.; Seattle (WA), 1993.
64. Darios, F.; Mochel, F.; Stevanin, G. Lipids in the Physiopathology of Hereditary Spastic Paraplegias. Front Neurosci 2020, 14, 74, doi:10.3389/fnins.2020.00074.
65. Estrada-Cuzcano, A.; Martin, S.; Chamova, T.; Synofzik, M.; Timmann, D.; Holemans, T.; Andreeva, A.; Reichbauer, J.; De Rycke, R.; Chang, D.I.; et al. Loss-of-function mutations in the ATP13A2/PARK9 gene cause complicated hereditary spastic paraplegia (SPG78). Brain 2017, 140, 287-305, doi:10.1093/brain/aww307.

66. Rinaldi, C.; Schmidt, T.; Situ, A.J.; Johnson, J.O.; Lee, P.R.; Chen, K.L.; Bott, L.C.; Fado, R.; Harmison, G.H.; Parodi, S.; et al. Mutation in CPT1C Associated With Pure Autosomal Dominant Spastic Paraplegia. JAMA Neurol 2015, 72, 561-570, doi:10.1001/jamaneurol.2014.4769.
67. Fan, Z.; Greenwood, R.; Felix, A.C.; Shiloh-Malawsky, Y.; Tennison, M.; Roche, M.; Crooks, K.; Weck, K.; Wilhelmsen, K.; Berg, J.; et al. GCH1 heterozygous mutation identified by whole-exome sequencing as a treatable condition in a patient presenting with progressive spastic paraplegia. J Neurol 2014, 261, 622-624, doi:10.1007/s00415-014-7265-3.
68. Faber, I.; Prota, J.R.; Martinez, A.R.; Lopes-Cendes, I.; Franca, M.C.J. A new phenotype associated with homozygous GRN mutations: complicated spastic paraplegia. Eur J Neurol 2017, 24, e3-e4, doi:10.1111/ene.13194.
69. Bross, P.; Naundrup, S.; Hansen, J.; Nielsen, M.N.; Christensen, J.H.; Kruhoffer, M.; Palmfeldt, J.; Corydon, T.J.; Gregersen, N.; Ang, D.; et al. The Hsp60-(p.V98I) mutation associated with hereditary spastic paraplegia SPG13 compromises chaperonin function both in vitro and in vivo. J Biol Chem 2008, 283, 15694-15700, doi:10.1074/jbc.M800548200.
70. Hansen, J.J.; Durr, A.; Cournu-Rebeix, I.; Georgopoulos, C.; Ang, D.; Nielsen, M.N.; Davoine, C.S.; Brice, A.; Fontaine, B.; Gregersen, N.; et al. Hereditary spastic paraplegia SPG13 is associated with a mutation in the gene encoding the mitochondrial chaperonin Hsp60. Am J Hum Genet 2002, 70, 1328-1332, doi:10.1086/339935.
71. Dor, T.; Cinnamon, Y.; Raymond, L.; Shaag, A.; Bouslam, N.; Bouhouche, A.; Gaussen, M.; Meyer, V.; Durr, A.; Brice, A.; et al. KIF1C mutations in two families with hereditary spastic paraparesis and cerebellar dysfunction. J Med Genet 2014, 51, 137-142, doi:10.1136/jmedgenet-2013-102012.
72. Lossos, A.; Elazar, N.; Lerer, I.; Schueler-Furman, O.; Fellig, Y.; Glick, B.; Zimmerman, B.E.; Azulay, H.; Dotan, S.; Goldberg, S.; et al. Myelin-associated glycoprotein gene mutation causes Pelizaeus-Merzbacher disease-like disorder. Brain 2015, 138, 2521-2536, doi:10.1093/brain/awv204.
73. Mou, Y.; Li, X.J. Rescue axonal defects by targeting mitochondrial dynamics in hereditary spastic paraplegias. Neural Regen Res 2019, 14, 574-577, doi:10.4103/1673-5374.248108.
74. Verny, C.; Guegen, N.; Desquiret, V.; Chevrollier, A.; Prundean, A.; Dubas, F.; Cassereau, J.; Ferre, M.; Amati-Bonneau, P.; Bonneau, D.; et al. Hereditary spastic paraplegia-like disorder due to a mitochondrial ATP6 gene point mutation. Mitochondrion 2011, 11, 70-75, doi:10.1016/j.mito.2010.07.006.
75. Yahalom, G.; Anikster, Y.; Huna-Baron, R.; Hoffmann, C.; Blumkin, L.; Lev, D.; Tsabari, R.; Nitsan, Z.; Lerman, S.F.; Ben-Zeev, B.; et al. Costeff syndrome: clinical features and natural history. J Neurol 2014, 261, 2275-2282, doi:10.1007/s00415-014-7481-x.
76. Ozes, B.; Karagoz, N.; Schule, R.; Rebelo, A.; Sobrido, M.J.; Harmuth, F.; Synofzik, M.; Pascual, S.I.P.; Colak, M.; Ciftci-Kavaklioglu, B.; et al. PLA2G6 mutations associated with a continuous clinical spectrum from neuroaxonal dystrophy to hereditary spastic paraplegia. Clin Genet 2017, 92, 534-539, doi:10.1111/cge.13008.
77. Travaglini, L.; Aiello, C.; Stregapede, F.; D'Amico, A.; Alesi, V.; Ciolfi, A.; Bruselles, A.; Catteruccia, M.; Pizzi, S.; Zanni, G.; et al. The impact of next-generation sequencing on the diagnosis of pediatric-onset hereditary spastic paraplegias: new genotype-phenotype correlations for rare HSP-related genes. Neurogenetics 2018, 19, 111-121, doi:10.1007/s10048-018-0545-9.
78. Capri, Y.; Friesema, E.C.; Kersseboom, S.; Touraine, R.; Monnier, A.; Eymard-Pierre, E.; Des Portes, V.; De Michele, G.; Brady, A.F.; Boespflug-Tanguy, O.; et al. Relevance of different cellular models in determining the effects of mutations on SLC16A2/MCT8 thyroid hormone transporter function and genotype-phenotype correlation. Hum Mutat 2013, 34, 1018-1025, doi:10.1002/humu.22331.
79. Bialer, M.G.; Lawrence, L.; Stevenson, R.E.; Silverberg, G.; Williams, M.K.; Arena, J.F.; Lubs, H.A.; Schwartz, C.E. Allan-Herndon-Dudley syndrome: clinical and linkage studies on a second family. Am J Med Genet 1992, 43, 491-497, doi:10.1002/ajmg.1320430173.
80. Patel, H.; Cross, H.; Proukakis, C.; Hershberger, R.; Bork, P.; Ciccarelli, F.D.; Patton, M.A.; McKusick, V.A.; Crosby, A.H. SPG20 is mutated in Troyer syndrome, an hereditary spastic paraplegia. Nat Genet 2002, 31, 347-348, doi:10.1038/ng937.
81. Evangelista, T.; Bansagi, B.; Pyle, A.; Griffin, H.; Douroudis, K.; Polvikoski, T.; Antoniadi, T.; Bushby, K.; Straub, V.; Chinnery, P.F.; et al. Phenotypic variability of TRPV4 related neuropathies. Neuromuscul Disord 2015, 25, 516-521, doi:10.1016/j.nmd.2015.03.007.
82. Macedo-Souza, L.I.; Kok, F.; Santos, S.; Amorim, S.C.; Starling, A.; Nishimura, A.; Lezirovitz, K.; Lino, A.M.; Zatz, M. Spastic paraplegia, optic atrophy, and neuropathy is linked to chromosome 11q13. Ann Neurol 2005, 57, 730-737, doi:10.1002/ana.20478.
83. Lynex, C.N.; Carr, I.M.; Leek, J.P.; Achuthan, R.; Mitchell, S.; Maher, E.R.; Woods, C.G.; Bonthon, D.T.; Markham, A.F. Homozygosity for a missense mutation in the 67 kDa isoform of glutamate decarboxylase in a family with autosomal recessive spastic cerebral palsy: parallels with Stiff-Person Syndrome and other movement disorders. BMC Neurol 2004, 4, 20, doi:10.1186/1471-2377-4-20.
84. Mitchell, S.; Bundey, S. Symmetry of neurological signs in Pakistani patients with probable inherited spastic cerebral palsy. Clin Genet 1997, 51, 7-14, doi:10.1111/j.1399-0004.1997.tb02406.x.
85. Orthmann-Murphy, J.L.; Salsano, E.; Abrams, C.K.; Bizzi, A.; Uziel, G.; Freidin, M.M.; Lamantea, E.; Zeviani, M.; Scherer, S.S.; Pareyson, D. Hereditary spastic paraplegia is a novel phenotype for GJA12/GJC2 mutations. Brain 2009, 132, 426-438, doi:10.1093/brain/awn328.

86. Lee, J.Y.W.; Hsu, C.K.; Michael, M.; Nanda, A.; Liu, L.; McMillan, J.R.; Pourreyron, C.; Takeichi, T.; Tolar, J.; Reid, E.; et al. Large Intragenic Deletion in DSTYK Underlies Autosomal-Recessive Complicated Spastic Paraparesis, SPG23. Am J Hum Genet 2017, 100, 364-370, doi:10.1016/j.ajhg.2017.01.014.
87. Bilguvar, K.; Tyagi, N.K.; Ozkara, C.; Tuysuz, B.; Bakircioglu, M.; Choi, M.; Delil, S.; Caglayan, A.O.; Baranoski, J.F.; Erturk, O.; et al. Recessive loss of function of the neuronal ubiquitin hydrolase UCHL1 leads to early-onset progressive neurodegeneration. Proc Natl Acad Sci U S A 2013, 110, 3489-3494, doi:10.1073/pnas.1222732110.
88. Mintchev, N.; Zamba-Papanicolaou, E.; Kleopa, K.A.; Christodoulou, K. A novel ALS2 splice-site mutation in a Cypriot juvenile-onset primary lateral sclerosis family. Neurology 2009, 72, 28-32, doi:10.1212/01.wnl.0000338530.77394.60.
89. Silani, V.; Corcia, P.; Harms, M.B.; Rouleau, G.; Siddique, T.; Ticozzi, N. Genetics of primary lateral sclerosis. Amyotroph Lateral Scler Frontotemporal Degener 2020, 21, 28-34, doi:10.1080/21678421.2020.1837177.
90. Mitsumoto, H.; Nagy, P.L.; Gennings, C.; Murphy, J.; Andrews, H.; Goetz, R.; Floeter, M.K.; Hupf, J.; Singleton, J.; Barohn, R.J.; et al. Phenotypic and molecular analyses of primary lateral sclerosis. Neurol Genet 2015, 1, e3, doi:10.1212/01.NXG.0000464294.88607.dd.
91. Munch, C.; Sedlmeier, R.; Meyer, T.; Homberg, V.; Sperfeld, A.D.; Kurt, A.; Prudlo, J.; Peraus, G.; Hanemann, C.O.; Stumm, G.; et al. Point mutations of the p150 subunit of dynactin (DCTN1) gene in ALS. Neurology 2004, 63, 724-726, doi:10.1212/01.wnl.0000134608.83927.b1.
92. Al-Saif, A.; Bohlega, S.; Al-Mohanna, F. Loss of ERLIN2 function leads to juvenile primary lateral sclerosis. Ann Neurol 2012, 72, 510-516, doi:10.1002/ana.23641.
93. Cookson, M.R.; Lockhart, P.J.; McLendon, C.; O'Farrell, C.; Schlossmacher, M.; Farrer, M.J. RING finger 1 mutations in Parkin produce altered localization of the protein. Hum Mol Genet 2003, 12, 2957-2965, doi:10.1093/hmg/ddg328.
94. Osmanovic, A.; Rangnau, I.; Kosfeld, A.; Abdulla, S.; Janssen, C.; Auber, B.; Raab, P.; Preller, M.; Petri, S.; Weber, R.G. FIG4 variants in central European patients with amyotrophic lateral sclerosis: a whole-exome and targeted sequencing study. Eur J Hum Genet 2017, 25, 324-331, doi:10.1038/ejhg.2016.186.
95. Yang, Y.; Zhang, L.; Lynch, D.R.; Lukas, T.; Ahmeti, K.; Sleiman, P.M.; Ryan, E.; Schadt, K.A.; Newman, J.H.; Deng, H.X.; et al. Compound heterozygote mutations in SPG7 in a family with adult-onset primary lateral sclerosis. Neurol Genet 2016, 2, e60, doi:10.1212/NXG.0000000000000060.
