# Supplementary material for: Mutations and Protein Interaction Landscape Reveal Key Cellular Events Perturbed in Upper Motor Neurons with HSP and PLS
Source: Brain Sci. 2021 Apr 29;11(5):578. doi: 10.3390/brainsci11050578 (PMC8146506; doi:10.3390/brainsci11050578)
Supplement: Supplementary file 1 [file brainsci-11-00578-s001.zip › Supplementary Table 2- HSP and PLS genes with their binding partners associated with the references for each direct interaction.pdf]

1.	ABCD1	ABCD1, ABCD3, DNMT3A, DNMT3B, PEX19[1]
2.	ACP33/SPG21	AGTRAP [2,3], AKIRIN2, ARL6IP1, ATPAF2, CCDC102B, CCDC33, CD4[4], CMTM5[2], CRYAA/CRYAA2, CTPS2, CUTC, DTX2, DTX3L, EFHC2, FAM114A1, GGA2, GSK3A, IKZF3, INCA1, KRT15, LMNA[5], MID2[2], MOV10[6], MTMR9, PRPS1, RABAC1, REEP6, RTN4[7], S100B, SPRED2, TCF12, TCF4, TFG, TRAF1[2], TRAF2, TRIM23, TRIM54[2], TRIM9, ZNF263
3.	ADAR	ADARB1, ILF2, ILF3, IRF3, SIRT1[8], USP22
4.	ALDH18A1	ADRB2[9], AGTRAP[2], ALDH, BAG4, C1QBP[10], CBX1, CDK2[11], CERK, CMTM5[2], CUL3[12], DCP1A, DCP2, DDX6, EDC3, EDC4, EED[13], G3BP1[14], GABRA2[15], GOLT1B[10], HAUS1, HDAC5[16], LATS2, LLGL2, MAP2K1, MARF1, MICAL3, MOV10[6], MRPL58[17], MYC[18], NFATC2[19], NR3C1[20], NTRK1[21], NXF1[6], PATL1, PNRC1, PPP1R37, PTPRR, RALB, SENP3, SHMT2[6], SIRT7[22], STAU1[23], TBX3, TCF3[24], TES, TRIM69, TRMT6, TUBGCP5, UBR2, VCP[25], ZRANB1, glutamate 5-kinase, glutamate-5-semialdehyde dehydrogenase
5.	ALDH3A2	CCND1[26], CTNNB1[27], FEV, NR1I2, PPARA[28], PPARD, STAT5B[29], TCF7L2[30]
6.	ALS2	ALS2, C21orf2, CDC37[31,30], DNAAF2, GRIP1, HNF1A, HNF4A[32], KDM4A, NEK1, RAB17, RAB5A, RAB5B, RAB5C[33], RAC1[33-35], RBM6, Rab5, SDF2, SOD1[36], STXBP6, UXT, VCP[25], YWHAB[37,38]
7.	ALS15	HERC3, NFE2L2[39], TP53[40], UBE3A
8.	AMPD2	Ampd, AMPD2, CCDC198, CCNDBP1, CD44, CTBP1, DCAF8, DDX17, EPS8, GPBP1L1, GPR156, KCNMA1[41], LZTS2[42], MLX, N6AMT1, NEDD4, NME7, NRL, NTRK1[21], PDE4DIP, POT1[43], SDC1, SDC2, TCHP, TERF1, TRMTT112, TSC1, TUBG1[44], TUBGCP4, YWHAB[45]
9.	AP4B1	AP4E1[10,46,47], AP4M1[10,47,48], AP4S1[10,46], BUB1, BUB1B, FIBP, GOLIM4[10], HNF4A[32], LAMA1[10], MAP3K4[10], POC1A[49], TEPSIN[10], TFAP2A[10], TMEM17[49], XPO1[50], YME1L1[10], ZNF576
10.	AP4E1	ALB[51], AP4B1[10,52], AP4M1[10,47], AP4S1[10,47], ARF1[53], CCDC6, CEP104, ELAVL1[54], GOLIM4[10], LAMA1[10], MAP3K4[10], MTNR1B, OSBPL1A, POLG, POLG2, RPS3, STXBP1, SUV39H2[42,55], TEPSIN[10], TFAP2A[10], TMEM17[49], WRAP53, XPO1[50], YME1L1[10]
11.	AP4M1	ACY1, AKT2, AP4B1[10,47], AP4E1[10,47], AP4S1[10], ARF1[53], ARID2, ARPIN/C15orf38-AP3S2, CBX4, CDK11A, CHTF8, DDX52, GALNT2, GOLIM4[10], H3F3A/H3F3B, HMGA1, IMPDH2, KLF3, LAMA1[10], LAMP2, LCMT2, LDLR, MAP3K4[10], MED31, MRPL48, MRPS23, NPAT, RALA, SFTB2, SUV39H2[42], TEPSIN[10], TFAP2A[10], TFB1M, TFRC, TGOLN2, TIMM29, USP47, WDR76, XPO1[50], YME1L1[10], ZBTB22, ZNF318
12.	AP4S1	AFP, AP4B1[10], AP4E1[10,47], AP4M1[10], APP[56], APPBP2, CDC73[10], CEP104, GALNT2, GOLIM4[10], GRB2[57], GTF2I,

		HLTF[10], KDM5B, LAMA1[10], MAP3K4[10], MECP2, RPL10, SLC9A1, SUV39H2[42], TEAD3, TEPSIN[10], TFAP2A[10], TIMM29, TMPO, YME1L1[10]
13.	AP5Z1	ARID4A, ATG7, FOXK1, FOXK2, HCFC1, HCFC2, IRF2, IRF2BP1, IRF2BP2, IRF2BPL, LYPD4, MDFI[7], NCOR1, PPP3CA[58], PPP3CB, REL, SIN3A, SUDS3, TBL1XR1, UBA7, YWHAB[38]
14.	ARL6IP1	ACSF2, ARL6, ARL6IP1, ATP2B2, C12orf10, COL4A3BP, DIABLO, ELAVL1[54], FAF2[59], FBXW7, FDFT1, FPR2, FXR2[7,60], GAD2, GOLPH3, GOLPH3L, GORASP2, HGS, HMGCL, INPP5K, IPPK, LMNA[5], MTERF3, MYC[61], NAPB, NDRG4, NUDT3, PAM16, PBX3, RAB33A, RETREG3, RTN4[2,42], SENP2, SHMT2[2,62], SNX1, SNX10, SNX11, SNX12, SNX15, SNX3[2], SNX4, SPG21, SUCLA2, TFAM, TUFM, UNK[63], WIPI2, YWHAZ[64], ZFYVE21, ZNF391
15.	ARSA	TAL1, TCF3[65], TCF12, TFEB
16.	ARSI	AHCYL2[42], Aryl Sulfatase, HSPA5[42], KRT40[2], NOL11
17.	ATAD3A	AATK, ACAD9[66], ACOT9, AGAP2, AIFM1[67], AMOT, APOBEC3B, ATP5F1A[66], BRF2, BTRC[68], C15orf48, C20orf24, CALM1 (includes others)[67], CALU, CCDC8[69], CEP57, CFTR[70], CHMP2B[71], COA3, COQ2, COQ9, COX15[72], CUL1[12], CUL3[12], CUL7[69], CYLD[73], DERL1[10], DLST[72], DNAJC7, DYNLL1, DYNLL2, ECSIT[66], EED[13], EPB41L2, EPB41L3, ESR1[74], ESRRB, FANCD2[75], FBXO25[76], FBXO6[77], FBXW11[68], FMNL1, FN1[78], G3BP1[14], GAN[79], HDAC6[10], HDGF, HNRNPF, HSPD1[66], ILK[80], IRS4, ITGA4[81], Immunoglobulin[82], KIF23, KLHL7, KSR1[83], LGR4, MAP3K5, MAP3K6, MCM2[84], MEX3C, MICAL3, MKRN1, MLH1, MTF1, MYC[85], MYL6, NACC1, NDUFA4, NDUFAF1, NDUFS3, NF2[83], NTRK1[21], OBSL1[69], OCIAD1, PDHA1[72], PHKG2, PMS2, POC5, PPEF1, PPP4C[86], PRKN[87], PRPF40A, PSMD6, PTP4A3, PTPMT1, PTPN14, RACGAP1, RECQL4, RIPK3, RNF2[13], SH3KBP1, SHCBP1, SIRT7[22], SLC1A5, SLC25A3[66], SLC25A5[66], SOAT1[72], SPAG5, SPATA5, SRGAP2, SUZ12[13], SYNE1[67], TNFRSF1A[88], TNFRSF1B, TPM1, TRADD, TRAM1, TRIM25[89], TUBA1C[10], TUBB2B, TUBG1[10], TUBGCP2, UBE2A, UVRAG, VCAM1[78], VDAC1[72], WWC1, WWOX, YAP1[90], YME1L1[10], YWHAB[68], YWHAE[68], YWHAG[68], YWHAH[68], YWHAQ[68], YWHAZ[91,92], ZNF746
18.	ATL1	ATL1[93-95], GABRA2[15], LNX1, NTRK1[21], REEP5[96], RTN3, RTN4[96], SPAST[97,98], TMED2[99], ZFYVE27[100,101]
19.	ATP13A2	AAK1, ACKR3, ACTB[102], ATL3, ATP5MC2, BNIP3L, CCKBR, CCT8[10], CD79B[42], CMTM6, COL1A1[102], CXCR4, CYGB, DCAF7[102], EMC7, ESPL1, F2R, FAM111B, FAM192A, FAM8A1, FKBP8[102], FUNDC2, GAK, GET4, GPR21, HAVCR2, HDAC6[102], HIPK1, HLA-DPA1, HSPA8[102], ICAM2[102], LMAN2, LRP6, MCOLN3[42], MEX3B, MYCBP2, MYH9[10], NPY1R, NRXN1, OCA2, OSTC, PCDHA3, PCDHB10, PCDHB11,

		PCDHGB4, PCMTD2, PDIA6, PEX19[55], POPDC2, PTH1R, RNF170[42], RTEL1, SCN3B, SEC61B, SLC17A2, SLC39A4, SLC4A8, SPCS2, SYT11, TMCO3, TSPAN14, UBE2J2, VAMP2, WDR5B, WDR83OS, YIF1A, ZNRF4, phosphatidic acid, phosphatidylinositol 3,5-diphosphate
20.	B4GALNT1	(N-acetylneuraminyl)-galactosylglucosylceramide N-acetylgalactosaminyltransferase, B3GALT4, ITM2B, LMO2, NFE2L2[39], B4GALNT1, PCDH20, ST8SIA4, TCTN2[42], TMED6, TRIM25[89]
21.	BICD2	ACTR1A, DCTN1[103], LRPPRC, MYH9[103], NEK8, TP53[104], BICD2[105]
22.	BSCL2	ASIC4, ATAD1, B4GAT1, CANX[106], CD47, CHPT1, DNAJC13, EBP, ERGIC3, GABRA6, GABRE, GABRG2, GLP1R, GLRA2, GPR89A/GPR89B, HDGFL3, HOXA1, HTR3A, ICAM2[55], IL20RB, KIAA2013, MCOLN3[55], NSG1, PLEKHF2, POM121/POM121C, SC5D, SCAMP2[55], SLC35B2, SLC39A3, SMIM3, SMLR1, TMEM19, TMEM25, TMPPE, TSPAN17, TSPAN5, UPK1A, USE1, YBX2, ZDHHC17
23.	C12orf65	APP[56], CDK2[107], MYLK2
24.	C19orf12	APP[56], IKBKG[108], SMARCA4
25.	CAPN1	calpain, carazolol, cholic acid, p85 (pik3r), ACTC1, ACTN2, ADRB2[109], AES, AIFM1[110,111], AIMP1, AIMP2[112], ANKRD17, APOA1[109], APP[113], ARRB2, ATG5[114], BAG3, BAX[115], BCL2, BCL2L1[116], BI-167107[109], BID, CAMK2A, CAPN10, CAPN11, CAPN2, CAPN3, CAPN5, CAPN6, CAPN7, CAPN8, CAPN9, CAPNS1, CAPNS2, CARS2, CASP7, CAST, CD109, CD99L2, CDK5[117], CDK5R1, CDK5R2, CEP164, CLEC4G, COG1, COL1A1[118], COL3A1, CPEB3, CREG1, CTNNB1[119], CTSC, Calcineurin A, DARS, DES, ECHS1, ECT2, EED[13], EZR[120], F2R, FANCA, FANCC, FANCD2[75], FANCG, FBXL12, FERMT3, FHL2, FN1[78], FNTA, FNTB, GAS2, GPHN, GPT, GRIN2A, GRIN2B, HIF1A[121], HNRNPD, HSP90AB1[122], HTT[123-125], INA, ITGA4[81], KARS, KATNA1, KNG1, KTN1, LAMTOR1, LGALS3, MCM2[84], MIR124, MLPH, MYBPC3, NCS1, NDUFB7, NEFM, NES, NFE2L1, NFKBIA[126], NMT1, NSMF, NTRK1[21], PALB2, PAX3, PHLPP1, PIK3CA, PLEC, POLA2, PRKCA, PRKCB, PRKCI, PRMT5, PSEN2, PTGDS, PTPN5, PTPRN, QARS, RAD21, RARS[112], RCAN1, RP9, SELENOK, SH3BGR, SIRT7[22], SLC32A1, SLC4A1, SLC8A3, SLIT3, SNRNP70, SPTAN1, STAT3, STAT5A[127], STAT5B[127], SYNE1[118], TBC1D22B, TINAGL1, TIRAP, TLR2, TNNI3, TP53[128,129], TRIP12, TRPC5, UFSP2, VCAM1[78], VIM[42,118], ZIC1
26.	CCT5	ACAP2, ACTA1, ACTA2, ACTB[130-132], ACTR1B, ACTR2, AEBP2, AGO4, ALDH7A1, ALKBH3, ALKBH8, APPBP2, ARG1, ARMC6, ATF2[133], B9D1, BAG2, BBS12, BBS7, BRCA1[134], BRD2, BTRC[135], BUB1, BUB1B, BUB3, CAMSAP2, CCND1[26], CCT2[10,49,92,136], CCT3[10,49,92,136], CCT4[10,49,92,136],

		CCT6A[10,49,92,137], CCT7[10,49,92,137], CCT8[10,49,92,137], CDC20, CDC5L, CDC73[138], CDCA4, CDK1[10], CDK2[11], CDK5[80,92], CDK9[139], CETN2, CFTR[70], COP1, COQ6, CRBN, CRY2[140], CTTNBP2, CUL3[12], CUL4A[12], CUL7[69], CYLD[73], DCAF11, DCAF5, DCAF7[42,141], DCAF8, DCTN1[142], DDB2, DDX5, DKC1, DNAI2, DNM2, DOCK5, DOCK8, DSP, DTL, DYNC1H1, ECD, EED[13,143], EEF1AKMT3, EIPR1, ELP1, EPB41L2, EZH1, EZH2[143], FAM45A, FAM86B2/FAM86KP, FANCD2[75], FBXL19, FBXL6, FBXO25[76], FBXO6[77], FBXW4, FKBP5, FN1[78], FUS[144], GAN[79], GANAB, GNB1[10,145], GNB5, GNG12, GPN1[139], GRB2[146], H2AFX, HCFC2, HDAC1[10,42], HDAC3, HDAC5[16], HDAC6[147,148], HEY1, HLTF[10], HNRNPH1[139], HSP90AA1[149], HSPD1[10], HUWE1[150], IDH2, IGBP1, IKBKE[88,92], ILF3, ILK[10,80,92], IMMT, INTS7, INVS, IRAK1, ITGA4[81], JARID2, KBTBD6, KBTBD7, KDM4B, KIAA1109, KIF21A, KIF21B, KLHDC2, KLHDC8A, KLHL33, KSR1[83], LGALS3BP[128][128][128], MAP3K1[88], MAP3K3[88], MAPK13[151], MAPK3[152], MCM2[84], MCPH1[153], MDM2[154], MKKS, MKS1, MMP12, MOB4, MTF2, MTNR1A[155], MTR, MYC[18], NEDD1, NEK4[156], NIPSNAP1[10], NLE1, NOS2[157], NSMAF, NSUN4, NTRK1[21], OBSL1[69], ODF1, PACRG, PAN2, PCK1, PDCL, PDCL3, PDIA3, PDK3[10], PEX14, PEX7, PFDN1, PFDN2, PFDN4, POC1A[42], POLR2H, POU5F1, PPM1J, PPP2CA[10], PPP2CB, PPP2R1A, PPP2R1B, PPP2R2A, PPP2R2B, PPP2R2C[42], PPP2R2D, PPP4C[10], PPP4R1, PPP4R2, PPP4R3A, PPP4R3B, PPP6C[10], PRPF4, PTEN[158], PTP4A1, PTP4A2, PTPA, pubchem compound 1800613[159], pubchem compound 2378425[159], pubchem compound 4962364[159], RASIP1, RBBP4, RBBP7, RFWD3, RGS7, RIPK3, RNF2[13], RPAP2, RPAP3, RPL39, RPS6KB2, RUVBL1, SAMM50, SEC31B, SEH1L, SET, SGK2, SIRT6, SIRT7[10], SNW1, SPRR2E, SRRM1, SRRM2[160], SSSCA1, STK24, STRN, STRN3, SUMO3, SUV39H2[143], SUZ12[143], SYNE1[136], TANK, TBC1D17, TBK1, TBL1Y, TCP1[161], TEFM, TERT, THEG, THOC2, THOC3, TP53[162], TRAF2, TRAF3IP3, TRIM25[89], TRMT112, TSSK6, TTC27, TUBA1A, TUBA1C[10], TUBA3E, TUBB2A, TUBB2B, TUBB4B, TUBB6, TUBE1, TUBG1[10], TXNDC9, TYK2, UBC[163], UBR7, UNK[63], UTRN, VAPA[164], VAPB[164], VCAM1[78], VCP[25], VHL[165], WDR48, WDR61, WDR76, WDR77, WDR82, WDR83, WDR86, WDR92, WDTC1, WRAP53, XRN1, YAP1[90], ZFYVE27[164], ZNF207
27.	CPT1C	CPT1, Cpt, GATA2, GRIA, ZFYVE27[100]
28.	CYP2U1	Unspecific monooxygenase, CYP2U
29.	CYP27A1	No interaction found
30.	CYP7B1	CTNNB1[168], CYP7B, ESR1[169], GABPA, GHITM, HNF4A[170-172], IKZF1, NADH2 or NADPH2 1 atom incorporation: oxygen

		oxidoreductase, NFKBIA[173], NR0B2, NR1I3, NR5A2, PPARA[174], RORA, RORC, SP1[175], UBB[42]
31.	C9orf72	ATG13, RAB1A, RB1CC1, SMCR8, ULK1
32.	DCTN1	ARFIP2, BICD2[103], CADPS2, CALY, CAPZA2[42], CDK5[176], CEP290, CSNK1D[177], CYFIP1, DCTN2, Dst, DUOXA1, DYNC1H1[10], DYNC1I1, Dynein, ECPAS, EPB41, GRB2[178], HAP1[179], HTR1A, HTT[180], IFT140, KIF5B[181], MAP4, MAPRE1, MAPRE2, MAPRE3, NUMA1, PAFAH1B1, PIK3CB, PIM1, SAR1A, SEC23A, SNX1[182], SNX2, SNX6
33.	DDHD1	phosphatidic acid[183], AMOTL1, CALML3, CDADC1, COBLL1, DYNLL1, DYNLL2, IFFO1, SCML1, STAT6, TRIM25[89]
34.	DDHD2	triacylglycerol lipase, AARSD1, ALOX5, APP[56], BLVRA, C2orf68, CHI3L1, EEF1A2, EMILIN1, LRRC8E, MTNR1A[155], RAPGEF5, RCCD1, SEC23IP, TGM4
35.	ELOVL1	GATA3, SIRT6, SOX2, TCF7L2[184]
36.	ENTPD1	apyrase, nucleoside-diphosphatase, nucleoside-triphosphatase, ATF2[185], ATPase, BCL6, FOXP3[186,187], GFI1, PPARA[188], RANBP9[189], TCF3[190], TCF4
37.	EPT1	No interaction found
38.	ERLIN1	ADRA1D, AGR3, AMFR[59,191], ASB11, BMPR1A, C1QBP[10], C6orf120, CALML3, CD2AP[10], CFTR[70], CHMP4B[10], CKAP5, COX15[72], CUL7[69], DBN1, DUSP3[42], EDEM3[192], ERLIN2, FA2H, FAF2[59], FANCD2[75], FBXO6[77], GABRA2[15], GOLT1B[10], HNF4A[32], INSIG1[193], ITPR1[194], Ktn1[10], MYEF2, NTRK1[21], PKN2[10], RAB5C[10], RAB7A[10], RMDN3, RNF139, RNF170[42], RPGRIP1L, SEC22A, SPAST[10], STOM[195], SUZ12[196], SYVN1[59,191], TMED2[10], TMEM199, TMEM63B, TMUB2, TRAF6[88], TRIM25[10], TSG101[10], UBAC2, UBC[163], UFL1, VAPA[10], VDAC1[72], XRCC3
39.	ERLIN2	ADRA1D, ADRB2[9], AMFR[191], AMIGO1, APH1A, ARSK, ATP2A2[191], BMPR1A, C1QBP[10], CACNA2D1, CANX[191], CASP8, CCDC47, CCT2[198], CD109, CD3D, CEP128, CFTR[70], CLN3, CNTRL, COX15[72], CSE1L, CYLD[73], DAO, DCTN1[49], DERL1[199], DUSP3[59], EDEM3[192], EED[13], EEF1A1, ELAVL1[54], ERLIN1[59], EWSR1[200], FAF2[59], FAP, FBXO6[77], FKBP14, FN1[78], GALNS, GOLT1B[10], GSTM5, HAX1[191], HDAC11[201], HMGCR, HNRNPM, HSP90B1, HSPA4[191], HSPA5[191], Immunoglobulin[82], INSIG1[191], ITPR1[199], ITPR3, KCNMA1[202], KLHL21, Ktn1[10], MMS19, MRPL58[17], NCSTN, NEK4[156], NF2[83], NPHS2, NTRK1[21], PPP6R3, PSEN1[203], PSENEN, PSMD2, RAB5C[10], RAB7A[10], RGPD4(includes others), RHOT1, RIC3, RMDN3, RNF139, RNF170[42], RNF185, RNF5, RPGRIP1L, SCAP, SCLT1, SEC61B, SOAT1[72], SREBF1[193], SREBF2, SRPRB, STK3, STOM[195], SYVN1[199], TCF3[24], TCP1[191], TCTN1, TIMM50, TMED10, TMED2[10], TMEM259, TMEM63B, TMEM67, TMUB1, TMUB2, TRIM28, TUBA1A, TUBA8,

		TUBB[59], UBAC2, UBC[191], UBL4A[204], UFD1, UFL1, VAPA[10], VCP[191], VDAC1[72]
40.	FA2H	CREB3, ERLIN1[2], LMNA[5], TCF7L2[184], TCTN3[49], UBQLN4, ZFYVE27[100]
41.	FARS2	ADAMTSL4, ADD3, AGTRAP[2], APPL1[2], CALCOCO2, CHCHD2, CHST15, CMTM5[2], CUL3[12], DLAT, ENO1, ERMP1, G3BP2, GAMT, GDF15, HIST1H1A, HIST1H1E, HIST1H1T, HMBOX1, HNRNPA1[42,55], ICAM5, IKZF3, ILKAP, ISG15[205], KRT13, KRT31[2], KRT40[2], KRT83, KRTAP10-3[2], KRTAP10-5, KRTAP10-7[2], KRTAP10-9, LOC100996763/NOTCH2NL[2], MID2[2], MKRN3, MRPL38, MRPL58[17], NEDD1, NXF1[6], PDHA1[10], PDHB, PDHX, phenylalanine-tRNA ligase, R3HDM2, RCBTB2, RNF13, SHMT2[6], SPICE1, STAT5A[10], STX11, TADA2A, TFCP2, TOMM40, TRIM27[2], TRIM54[2], USP45, ZC3H3, ZCRB1
42.	FIG4	SLC2A4, SNCAIP, PIKFYVE, VAC14
43.	FLRT1	ADGRL1, ADGRL2, ADGRL3, BLZF1, FGFR1[206,207], FLRT2, FLRT3, PCDH7, PCDHGC3, PIK3CA, PIK3CB, PIK3R1, PIK3R2[42], PIK3R3[42], RADIL, TCAF2
44.	GAD1	ABL1[208], AGTRAP[2], ASCL1, ATN1[209] , Calmodulin, CMTM5[2], CREB1, CTNNB1[27] , DLX2, DNMT1, EGR1, FGFR1[42], FYN[208], GAD, GAD1, GAD2, GRB2[208], GSX2, HAAO, Histone h3, HTT[210], JUN, KLF3, LHX1, LHX5, MAL2, NCK1, PITX2, PRKACA[211], PRKCE, PTF1A, STK3, UBC[212], ZFYVE27[100]
45.	GALC	CBX5, CTNNB1[213], STAT5A[214]
46.	GBA2	Beta-glucosidase, glucosylceramidase, AFG3L2, APP[56], BAG5, ELANE[215], GBA, ILVBL, LAMTOR5, MRPL44, MRPS22, MTNR1A[155], PPFIA1, PRKACA[216], SRP68, TMEM216
47.	GCH1	ATF4, CEBPA[217], NUPR1, PAX7, REKA, THRB
48.	GJC2	ALB[51], CONNEXIN, TCF7L2[184], UBC[218]
49.	GRN	ABI2, ACACA, ACTG1, ADAMTS7, ADAMTSL1, AGRP, AHCYL2[42], ARFGAP1, ATN1[219], ATP1B3, ATXN7, BMT2, BRCA1[134], C1orf106, CACNA1A, CACNG5, CCDC33, CCDC69, CCDC8[69], CCNG1, CCNT1, CD68, CDK2[11], CDK9[220], CELA2B, CFTR[70], CLEC4M, COMP, COX6B1, CRKL, CRY1, CSNK2B[80], CTBP1, CTBP2, CTSO, CTTN[221], CUL7[69], DBN1, DCP1B, DEFA1 (includes others), DLK1, DLX2, DNAJC10, ECM1, EDC3, EED[222], EGFL7, EGFR[223], ELANE[224] , FAM131C, FAM19A3, FAM207A, FANCL, FBXO6[42], FLYWCH2, FRAT1, GFI1B, GLRX3, GNB2, GRIA1, GRIA2[221], GRIA3, GRN, HECW2[225], HK3, HOXA1, HSP90AA1[80], HSP90AB1[80], HSPA4[226], HSPG2, HSPH1, KCNQ2, KRT18, KRTAP10-7[2], KRTAP26-1, LCOR, LEP, MAPK1[80], MASTL, MDK, mir-103, NAB2, NAPSA, NF2[83], NFKBIA[173] , NLK, NOTCH1, NPM1[227], NTRK1[21], NXF1[6], OTUD5, OTX1, P-TEFb, PDX1, PHLPP2, PIK3R2[107], PKP2, PLA2G10, POT1[107], PPP2CA[42],

		PRKAA1[228], PRKAB2, PRTN3, RAC1[107], RAPGEF6, RPL3, RPS6KA1, RPS6KA2, RPS6KA3, SAV1, SERPING1, SGTA, SIRT3[229], SLPI, SMAD9, SORT1, SPACA4, SPRY2, STN1, SYTL1, TAT, TGM2, TLE3, TLK2, TLR9, Tnf receptor, TNFRSF1A[230], TNFRSF1B, TOP3B, TRIB3, TUBA1C[143], VHL[165], WDR48, WFDC2, YY1, ZC3HC1, ZFP41, ZNF408, ZNF517
50.	HSPD1/HSP60	ABHD18, ABL1[231], ACAD9[232] , ACSF2, ACSF3, ACSS1, ACTL8, adenosine triphosphate[233], ADRB2[9], AGER, AHSA1, ALDH2, ALK, ALOXE3, AMBRA1, AMFR[234], APP[56], ASB4, ASB9, ATAD3A, ATF2[133], ATP synthase[235], ATP5F1A[66], ATP5PB[42], AURKA, AURKB, BAK1, BAX[236], BCAT2, BCKDHA, BCKDHB, BCL2L1[237], BCL6, BCOR, BCORL1, BCR, BIRC5, BPHL, BRCA1[107], C1QBP[238], CA2, CAND1[12], CASP3, CBX8, CCAR2, CCDC124, CCDC18, CCDC8[69], CCND1[26], CCT2[10], CCT3[10], CCT4[10], CCT5[10], CCT6A[10], CCT7[10], CCT8[10], CD4[239], CDC20, CDC5L, CDC73[138], CDH1[240], CDK2[10], CDK9[139], CDKN2AIP, CEP70, CFTR[70], CKAP5, CLEC7A, CLU, Collagen type X, COPS5[12], creatine kinase, CRKL, CRNKL1, CRY2[140], CTSH, CUL1[241], CUL2[12], CUL3[12,241], CUL4A[12], CUL7[69], CWC15, CYLD[73], DARS2, DCAF4, DCAF4L2, DCAF5, DCAF7[242], DCLRE1B, DDB2, DHFR, DKK3, E2F1, ECSIT[66], EDEM3[192], EED[243], EEF1AKMT3, ELANE[215], ERG, ESR1[244], ESRRB, ETFBKMT, FAHD2B, FAM107A, FANCD2[75], FBXO15, FBXO17, FBXO32[245], FBXO6[77], FBXO7, FDX1, FECH, FN1[78], FNBP1L, FOXO1, FOXRED1, FUS[144], G3BP1[14], GAN[79], GAPDH[246][246][246], GBA[10][10][10], GFM1, GJA1, GLB1L3, GLUL, GNB1[10], GNG12, GORASP1, GRB2[146], GSTK1, GTPBP8, HACE1, HARS2, HAX1[10], HDAC1[10], HDAC5[16], HDAC6[147], HDHD5, HIST1H3E, HIST2H2BE, HMG20A, HNRNPA1[247], HNRNPH1[139], HRAS, HSD17B10, HSF1[248], HSP, HSP90AA1[249], HSPA1A/HSPA1B[249], HSPA9[250], HSPD1[232], HSPE1[251], HTT[252], HUWE1[150], IBA57, IDH3G, IKBKG[88], IL5, Immunoglobulin[82], ISG15[205], ITGA3, ITGA4[81], ITGB1[253], KANSL2, KCNAB2, KCNMA1[202], KDM2B, KLHL33, KRT31[2], KRT40[2], KRTAP10-3[2], KRTAP10-8, KRTAP5-9, LALBA, LAMB3, LGALS3BP, LGR4, lipopolysaccharide, LOC100996763/NOTCH2NL[2], LRRC28, LRRK2, LZTS2[2], MAPK3[152], MAPK6, MARS2, MCM2[84], MCPH1[153], METTL17, METTL8, MFHAS1, MORC4, MOV10[6], MPST, MRM2, MRPL16, MRPL37, MRPL4, MRPL58[17], MRPS30, MTG1, MTG2, MUC13, MYC[18], MYCL[254], NANOG, NARS2, NCKIPSD, NDUFAF1, NDUFS3, NDUFS7, NEDD8[12], NEU4, NFS1, NIPSNAP1[10], NIPSNAP2, NIPSNAP3A, NOS1AP, NOS2[255], NPHP1, NPHP4, NPM1[256], NR3C1[257], NSUN4, NTRK1[21], NUDT19, NXF1[6], OBSL1[69], OXCT2, OXNAD1, PAN2, PARD3, PARK7, PCGF1, PCK1, PCK2, PDCL, PDE3A,

		PDK3[42], PFDN1, PHB2, PLCG2, PLG, PLPBP, POT1[10], POU5F1, PPP2R2C[86], PRAMEF17, PRCC, PRDM1, PRDX1, PRKDC, PRKN[87], PRMT1, PRNP[258], Proinsulin, PSMA3[259], PSMF1, PTEN[260], pubchem compound 129051172, pubchem compound 16227198, pubchem compound 1800613[159], pubchem compound 2378425[159], pubchem compound 4962364[159], PUS1, PUSL1, QTRT1, RAD52, RAF1[261], RASPGEF3, RASL10B, RC3H1, RFWD3, RGS20, RING1, RNF2[10], RPAP1[139], RPS6KB2, RPUSD3, RSC1A1, RYBP, SAMD3, SCML1, SDR39U1, SF3A2, SF3B5, SIRT1[229], SIRT3[42], SIRT4, SKA1, SKA2, SKA3, SKI, SKP1, SLC25A3[66], SLC25A39, SLC25A41, SLC25A43, SLC25A5[66], SLC39A10, SMAD2[262], SMPD3, SMURF1[263], SNCA[264], SNW1, SPART, SPN, SRRM2[160], SRRT, STARD3, STAU1[23], STUB1[265], TAZ, TCF3[10], TCP1[10], TEDC2, TERF1, TERF2[10], TERF2IP, TFB1M, THBS3, TINF2, TLR2, TLR4, TMCC2, TMEM70, TNRC6A, TP53[250], TRIB2, TRIM25[89], TRIM27[10], TRIM43/TRIM43B, TRIM63[266], TRMT1L, TRMT2B, TRMU, TRUB2, TSG101[10], TST, TTC39B, TUBA1C[10], TUBG1[10], TUBGCP3, U2AF2[267][267][267,256,247], UBC[163], UBL4A[204], UNK[63], USP7, VCAM1[78], VCP[128], VHL[268], WBP11, WDR5, XRCC3, YAF2, YARS2, YBEY, YBX1, YWHAZ[64], ZBTB48, ZNF703
51.	IBA57	AK3, ARHGAP22, ECHDC2, EIF4A1, GTF2E2, HSPD1[42], IMPDH1, LUC7L, MINDY3, MINOS1, MRRF, NIT1, OXLD1, RAB3A, TMEM184A
52.	IFIH1	ARL5B, ATG12, ATG5[269], CC2DA, DHX58, IRF3, IRF5, IRF7, MAVS, NR3C1[270], PCBP2, SOX11, STAT1, STAT4, STAT6, TKFC, TRIM24
53.	KCNA2	Adam22, ATN1[209], CACNA1A, CACNA1B, CACNA1E, CAMLG, CNTN2, CNTNAP1, CNTNAP2, CTTN[271], dalfampridine, DLG2, DLG4[272], DLGAP1, DRD2, FYN[273], GABRA2[15], KCNA1, KCNA2, KCNA3, KCNA4, KCNA5, KCNA6, KCNA7, KCNAB1, KCNAB2, KCNB1, LGI1, NEU1, PTK2B, PTPRA, RARA[274], RAS homolog, RGPD4 (includes others), RHOA, RTN4[275], SIGMAR1, SREBF1[276], UBC[218]
54.	KIAA0196/WASHC5	CAPZA1, CAPZA2, CEP128, EED[196], FKBP15, HNF4A[32], NTRK1[21], PTPRR, RPA1, RPA2[277], RPA3, SNX1, SNX27, TBC1D5, VNN2, VPS26A, VPS26B, VPS29, VPS35, WASHC1, WASHC2A/WASHC2C, WASHC3, WASHC4, XPO1[50]
55.	KIF1A	AARSD1, ADAMTSL4, adenosine triphosphate[278], APLP2, APP[279], AR[280], ARHGEF7, ARL5B, CEP152, CETN1, COX15[72], CPNE8, DCX, DLG4[281], DOCK5, DQX1, EGFR[282], ESR1[283], FAM19A3, FMR1[284], FXR2[280], GAS7, GATD1, GIT1, Histone h4, KIF1A, KIF1B, KIF1BP[42], LOC100996763/NOTCH2NL[280], LYPD4, MDFI[280], MED1, MID2[280], MKRN3, MTUS2[280], NTRK1[21], P38 MAPK, PLSCR1[280], PPARG, PPFIA1, Ppfia4, PPP2CA[285], PSMA3[280], PSMC3, RAC1[285], RASAL1, RBPMS[280], RETREG1, RFX6,

		RGS20, RHOA, RIMS1, RNF166, RNF31, RP2, RXRA, SIRT1[286], SIRT7[22], SP1[262], SPRY2, SYT4, TNS2, TRAF1[280], TRIM27[280], TRIM35, TRIP6, TSPOAP1, TUBA1A, TUBB6, UBC[287]
56.	KIF1C	14-3-3, ABLIM1, ADNP2, AGAP1, AHCY, ALYREF, ANKRD34A, ANXA7, ARF1[288], ARF3, BICD2[289], BICDL1, CAMSAP2, CBY1, CCDC9, CDC25B, CDC25C, CDK16, CEP170, CGN, CHTOP, CLASP2, DCLK1, DENND1A, DENND4C, DYNC2H1, EIF4A3, EIF4E2, EPB41L3, EVI5, FAM110B, FAM53C, GAPDH, GIGYF1, GIGYF2, HDAC4, HNRNPD, HSPA8[10], INO80B, INPP5E, INTS7, JUNB, KCTD3, KIF13B, KIF1B, KIF1BP[42], KIF1C, KSR1[195], LAMC3, LIMA1, LRFN1, LYST, MAGI1, MAP3K21, MAPKAP1, MAST3, MKNK1, MMGT1, MYH9[290], MYO1A, MYO1C, NADK, NBEAL1, NF1, NUP93, PHLDB2, PLXNA3, POLR2G, PPM1H, PRDM5, PRKAA1[228], PRPF4B, PTPN21, RAB6A, RAN, RANBP2, RANGAP1, RASAL2, ROBO2, RTKN, SH3PXD2A, SIPA1L1, SLX4, SRGAP2, SRSF12, SSBP1, STAU1[23], SUB1, SYDE1, TESK2, THOC1, THOC2, THOC5, THRAP3, TIAM1, TRIM25[89], UBE2N, USP21[195], UTY, YWHAB[45], YWHAE[107], YWHAG[291], YWHAQ[107], YWHAZ[107], ZBTB21, ZNF33A, ZNF638
57.	KIF5A	ABCD1, ACTB[292], Alyref, ANKRD27, APP[279], ARC, ATIC, BICD2[289], BMP1, CAMK2A, CHRNA9, COG6, DCTN1[293], DDX1, DDX3X, DISC1, DLG4[294], DLGAP1, DNM1, DTNB, DTNBP1, EXOC1, FANCD2[75], FMR1[294], Fus, GABA-A receptor, GABARAP, GABARAPL1, GABARAPL2, GLRA1, GPHN, GRB2[295], GRIA2[296], GRIP1, HACD3[292], HAP1[297], HNRNPU, INSR, ITSN1, KCNC1, KCNE3, KIF5B[298], KIF5C[298], KLC1, KLC2[299], LNX2, MAP4K4, MDM2[262], MED4, MTO1, MYCL[42], NCOA2, NONO, PCBD1, PIN1, PRKAA2, PURA, PURB, Rab11, RAPGEF2, RTCB, RTRAF, RXRB, SALL4, SFPQ, SMN1/SMN2, SNPH, STAU1[300], SYNCRIP, SYNE4, TBC1D5, TFCP2L1, TK1, TNIK, TP53BP2, TRAF3IP1, TRAK1, TRAK2, TRIP6, TSG101[301], VPS26B, VPS29, VPS35, YAP1[302], YWHAE[303], ZFYVE27[304]
58.	KLC2	AATF, AHI1, AIMP2[305], AKT1S1, ALDOA, AP3D1, APP[279], ARX, ATF6, ATF7IP, AZIN1, BRAT1, BSDC1, C9orf78, CCDC84, CDH1[306], CENPJ, CEP152, CEP44, CEP85, CHD8, CHRNA9, CLK1, CLSTN1, COG6, COIL, CROCC, DISC1, DPY30, DPYSL2, DTNBP1, DYNC1I1, EID1, ERCC4, EXOC3, EZH2[13], FAT1, FGFR1OP, FXR1, FYCO1, GEMIN6, GEMIN7, GPKOW, GRIA1, GRIA2[307], GRIN1, guanosine 5′-O-(3-thiotriphosphate), HAP1[308], HEATR3, HEXIM1, INO80, KCNMA1[41], KIDINS220, KIF5A, KIF5B[10], KIF5C[309], KLC1, KLC3, KLC4, KPTN, LSS, LYN, LYSMD2, MAML1, MAP2K7, MAPK8IP3, MAPT, MARK2, mir-10, MIS12, MOCS3, MRPS2, MTA3, MYCL[42], NAALADL2, NBEAL1, NCAPD2, NCAPG, NCAPH, NCBP1, NCOR1, NHS, NIPBL, NOLC1, NTRK1[21], NUP43, OASL, OFD1, OGT, ORC3, PARD3[310,308,296,287], PIAS1, PIK3R3[305], PLD1, PLEKHM2,

		PLPBP, POLR2C, POU1F1, PPIL2, PPP1R13L, PPP4C[10], PRKAG3, RAB11B, RAB11FIP1, RAB11FIP2, RASSF1, RBFOX2, RBM15B, RBM4, RHOG, S100A8, SCAMP2[10], SERPINB2, SFN, SH3KBP1, SLC38A10, SMC2, SMC4, SMC6, SNIP1, SOD1[10], SPART, SQSTM1, SRP14, SYNE4, TBC1D4, THEM4, TIMM8B, TJAP1, TMCO1, TOE1, TRAK1, TRAK2, TSC2, TUBA1A, TUBGCP3[128,127,125], UBFD1, VCP[10], WDR70[10], XPR1, YWHAB[45], YWHAE[310], YWHAG[37], YWHAH[310], YWHAQ[37], YWHAZ[64], ZC3H3, ZC3HAV1, ZNF346, ZNF503
59.	KY	No interaction found
60.	L1CAM	ADAM10, ALCAM, ANK2, ANK3, AP2A1, AP2A2, AP2M1, BARX1, BARX2, Beta adaptin, CANX[311], CASKIN1, CD47, Ck2, CNTN1, CNTN2, CTNNB1[119], DCX, DGUOK, DNAAF2, EGFR[312], EPHA4, ERBB, ERBB2, ERBB3, ERK, EZR[313], FBXO6[77], FGFR1[314], GRIN1, HAX1[42], Histone h3, Integrin, ITGA2B, ITGA5, ITGAV, ITGB1[315], ITGB3, LGALS4, MAPK1[316], NCAN, Neuropilin, NFE2L2[317], NRP1, NTRK1[21], NUFIP1, NUMB, PAX8, PEA15, PLG, PRNP[318], PSEN1[319], PTPRZ1, RABGEF1, RAF1[316], RANBP9[320], RELN, REST, Rsk, SNAI1, SNAI2, SNAP91, SOX10[321], SRC, TOB2, TP63, TRIM25[89]
61.	LYST	AHR, AP3D1, ARHGEF17, ATN1[322], BAIAP2L1, BRD3, CALM1 (includes others)[322], CAPZA2, CCDC9, CELF4, CENPJ, CEP85L, CNTROB, COL4A5, CSNK1D, CSNK2B[322], CTTN[10], DAP3, DDX60L, DNAJC13, DNAJC14, DYNC1LI1, EFS, ELMO2, ESRRA, ESS2, FKBP8[10], GAR1, HDLBP, HGS, HNRNPH2, HNRNPM, INO80, KIF1C, KLC3, KPNB1, LLGL2, MBNL1, MED12, MLH1, MRPL17, MRPS11, MRPS31, MYH13, NANP, NCDN[322], NR3C1[323], NR3C2, OSBPL2, PCBP4, PLXNA3, PMS1, POC1A[10], PPIA, PSMD6, PYCR1, RB1CC1, RTL8C, SAP30, SLC25A1, STX7, TACC1, TACC2, TNNI3, TNPO1, VAMP2, VTA1, YWHAB[322], YWHAQ[322], YWHAZ[322], ZNF521
62.	MAG	3'-sialyllactose, APP[56], CACNA1B, COL1A1[324], COL2A1, COL3A1, COL4A1, COL5A1, COL6A1, COL9A1, DCC, EGR2, FN1[325], FYN[326], ganglioside, ganglioside GD1a, ganglioside GQ1balpha, ganglioside GT1, ganglioside GT1a alpha, HLA-A[327], HNRNPA1[328], Igm, IQCB1[255], LILRB3, LRP1[329], MAP1B, MYC[330] , NGFR, POU3F1, PRNP[318], PSEN1[319], RTN4R, RTN4RL2, sialic acids, SOX10[331] , TCF7L2[184], TNR, trisialaoganglioside GT1a, tubulin, UBC[218]
63.	MARS	AIMP1, AIMP2[332], ATF4, BRCA1[134], BRF2, CASK, CCDC8[69], CDK9[333], COPS5[332], COQ2, CRY1, CRY2[140], CUL1[12], CUL3[12], CUL7[69], CYLD[73], DARS, DLST[72], EED[196], EEF1A1, EEF1E1, EGFR[334], EPRS, ESR1[335], FANCD2[75], FBXO25[76], FBXO6[77], FN1[78], FOXA1, G3BP1[14], GIPC1, HACD3[31], HDAC5[16], HNRNPUL1, HSP90AA1[336], HUWE1[150], IARS, IKBKG[88], ILK[37], ITGA4[81], KARS, Ktn1[10], LARS, LENG1, MAP3K1[88], MAP3K3[88], MCC[37],

		MCM2[84], MDM2[262], methionine-tRNA ligase, MTA1, MYC[37], NFKB2, NTRK1[21], PDHA1[72], PKN2[10], PPM1F, PRKAB1, QARS, RARS[42], RELA, RNF2[13], RPS27, SDCBP, SDCCAG8, TFE3, TNFRSF10D, TNNT1, TRAF6[37], VCAM1[78], WRAP73, ZNF746
64.	MFN2	BAK1, BAX[337], DNMT3B, EIF2AK3, GABRA2[15], HNRNPA1[338], HRAS, HTT[339], KDM5A, MFN2, NLRP3, PPARGC1A, RAF1[340], RAS, RB1[341], STOML2
65.	MT-ATP6	ACAD9[342], ATP synthase, ATP5F1A[342], ATP5F1B, ATP5F1C[342], ATP5F1D, ATP5MG, ATP5PB[342], ATP5PD, ATP5PO[342], BRI3BP, CHMP2B[10], CTC1, DNAJA2, ECSIT[342], FANCC, FOXRED1, IQGAP3, Oxphos, SP1[343], STAT3, TEN1, VDAC1[342], VDAC2, VDAC3
66.	NIPA1	No interaction found
67.	NT5C2	5′-nucleotidase, ASB2, ATP5PO[2], CALM1 (includes others)[344], CEP19, DUSP3[345], FBF1, FMR1[294], FXR2[7], HSPBP1, MOB1B, MOB3B, MOB3C, nicotinate D-ribonucleotide, NME7, NMN, NOC4L, NT5C2, NTRK1[21], nucleoside phosphotransferase, NUDT18, SDCBP, SNX15, SOCS1, TRIM25[89]
68.	OPA1	AHR, APOA1[109], GABRA2[15], HISTONE H3, KDM5A, NME4, RB1[341], SLC1A, TP53[346]
69.	OPA3	APP[56], ATP5F1A[347], ATP5F1C[347], ATP5PF, ATP5PO[347], C1QBP[347], CLPB, COX5A, ECH1, HNF4A[32], IDE, KDM1A, LAMC1, MTCH2, NRDC, NRG1, NXF1[6], OPA3, PCDHGB1, PCDHGB4, PHYHIPL, PRMT6, SCO1, SCO2, SLC15A1, SRF, STARD7, STOML2, SUV39H1, TIMM23, TNFRSF17, UBC[287], YME1L1[347]
70.	PARK2	ABL1[348], ACTA2, ACTG1, Alpha tubulin, AMBRA1, BAX[349], CASP3, CCND1[350], CCT2[351], CDK5[352], Cox5b, CTNNB1[353], CUL1[354], Cyclin E, EDNRB, EGFR[355], ELK1, ENKUR, FAM117B, FBXW7, FUBP1, GLO1, GPR37, HCFC1, HDAC6[356], Hsp70, HSPA9[357], HSPD1[87], IKBKG[358], IL1B, IL6, ITGB1BP2, KDR, LDHAL6B, MAOA, MAOB, MAPK8, MCL1, NADH dehydrogenase, PDHA1[359], PGLYRP3, PICK1, PKM, PRDX1, PRDX2, PRDX6, PRKCSH, PSMC1, PSMD3, PTPN5, RNF31[360], RNF41, SEPTIN4, SEPTIN5, SLC25A1, SLC6A3, SMCHD1, SNCA[361], SNCAIP, STUB1[362], SUMO1, TARDBP[363], TCP1[351], THAP11, TNF, TOMM40, TP53[364], TRAF2[365], TRIB3, TSG101[366], TUBA1B, TUBB[351], TUBB2A, UBE2G2, UBE2J1, UBE2L3, VDAC1[367], WDR61
71.	PCYT2	No interaction found
72.	PGAP1	ATP1B4, BTNL8, HTR3C[42][42][42,41,39], ISLR, ITM2B, MAP1LC3A, MCOLN3[42], MYC[37], NCEH1, NRG1, PLTP, PON2, SCGB1D1, SIRT3[229], TCTN2[42], UPK1A
73.	PLA2G6	1-palmitoyl-2-oleoylglycero-3-phosphoglycerol[109], ADRB2[109], APOA1[109], ARF1[368], BAG3, BI-167107[109], CALM1 (includes others)[369], Calmodulin, carazolol[109], cholic acid[109], Cpla2, L-

		alpha-palmitoyloleolyl-phosphatidylcholine[109], NEK4[156] , PLA, PLA2G6, quinacrine, SREBF1[370]
74.	PLP1	AKT1[371], APH1A, APH1B, BCL2L13, CALR, cholesterol, CLN8, CREB3, CREB3L1, DLG4[294], FAM241A, FGFR1OP2, GATD1, Histone h4, HLA-A[327], HTT[252], ITGAV, ITGB5, LMNA[5], LRP1[372], Mbp, MBP, MECP2, MHC Class II (complex), MHC II, MYC[373] , NKX2-1, NKX2-2, NRAS, OTOF, phosphate, phytic acid, PLP1[374], PPARD, PRNP[318], PSEN1[319], PTPRN, REEP5[42], REEP6, RNF114, RTN2, SIKE1, SLC1A2, SLMAP, SOX10[375], SOX8, STRIP1, STRN, STRN4, TCF7L2[184], UBC[218], ZFYVE27[100], ZNF202
75.	PNPLA6	ARMCX5-GPRASP2/GPRASP2, ATAD5, BAX[10], BCL2L1[376] , BDP1, C18orf21, CD79B[42], CDH5, EDRF1, EED[222], EMD, ERCC6, EVA1C, FAF2[377], FANCD2[75], GDI1, GEMIN2, GRK5, HAVCR2, HLA-DPA1, HNF4A[32], ILK[10], KCNS3, Ktn1[10], LIG3, LRRTM1, lysophospholipase, MMGT1, MOV10[6], MRAP2, NIN, NSFL1C, NTRK1[21], NXF1[6], PCDHB16, PDCD1, PPP6C[10], PRSS23, RAB5C[10], SERPINB12, SRP72, TEX264, TSPAN17, UBE2A, YY1, ZNRF4
76.	POLR3A	ASB13, ASB7, CDC26, CDH5, EVA1C, EWSR1[378], GPN1[139], GPN3, GTF2B, HSP90AB1[31], IKBKG[378], MAF1, NEDD4, NTRK1[21], NUDCD3, PCBD1, PFDN2, PIH1D1, PKP2, POLR1C, POLR2E, POLR2F, POLR2H, POLR3D, POLR3E, POLR3GL, POLR3H, PPP1CA[42], PPP1CC[42], PPP2R2C[42], PRKCZ, PTGES3, RAE1, RPAP1[139], RPAP2, RPAP3, RUVBL1, RUVBL2, SKAP1, SLC7A6OS, URI1, UXT, WDR92, ZNHIT2, ZW10
77.	RAB3GAP2	1-palmitoyl-2-oleoylglycero-3-phosphoglycerol[109], ADRB2[109], APOA1[109], ARF6, BI-167107[109], carazolol[109], cholic acid[109], EED[196], EXOSC6, GSTK1, L-alpha-palmitoyloleolyl-phosphatidylcholine[109], LMAN1, MAP1LC3A, MTNR1B, NTRK1[21], PAXIP1, PTP4A3, RAB3GAP1, SHC1, SIRT2, TES, TRIM25[89], VAPA[379], VAPB[42], VCP[25], ZFYVE27[100]
78.	REEP1	CAV3, ELAVL1[54], KCNA10, KDM5A, KDM5B, LYPD3, NCAM1[42], NKX2-3, Olfr992, PLSCR1[42], RB1[341], REEP2, REEP4, RUNX3, YWHAE[310], YWHAG[42], YWHAZ[42]
79.	REEP2	CAV3, DSG4, GLP1R, KCNIP2, REEP1, TCTN2[49], TCTN3[49], UBC[218]
80.	RIPK5/DSTYK	ARHGAP25, ATP5PB[37], B3GNT3, BSG, CCL22, CD79B[42], CDH13, CDK13, CEP170, CLTC[10], CTDSP1, DCAF15, DCTN1[42], DDT, DEF8, DKKL1, FRMD1, GRPR, HSPE1[37], IKBKE[37], IL1R2, KEAP1, LAMP3, LSM12, MAD2L1, MIF, MTPN, NCR3LG1, P2RX2, PLA2G10, PSMB1, PSMB3, RAB14[37], RAB2B, RAB5C[37], RPS9, SATB1, SLAMF1, TRIM25[89], UBC[218], UBE2K
81.	RTN2	ATP1B3, ATP1B4, FANCG, FBXO32[245], FPR2, GYPB, ICAM2[42], IPPK, JPH1, LPAR1, MCRS1, MYC[380], PLP1[42], RTN1, RTN4[42], SPAST[381], SYPL2, TMEM128, TNKS, VTN
82.	SERAC1	No interaction found

83.	SETX	ABI1, ACTB[382], BARD1, BRCA1[382], ENSA, ESR2, EXOSC9, KPNA2
84.	SLC16A2	DLK1, NFE2L2[39], NFKBIA[173], NR1H2, NR1H3, NT5E, PTH1R, RARA[383], RARB, RXRA, TEX29
85.	SLC33A1	APLNR, ARHGEF7, ATP2A2[10], ATP5F1A[10], ATP5F1E, ATP5PO[10], CANX[10], CEPT1, CKAP2, CKAP4, CLCN7, CLIC1, DOCK6, EEF1D, EEF1G, ELMO3, ELOB, EVC2, FANCD2[75], GIT1, HAX1[10], HNF4A[32], HSP90AB1[10], HSP90B1, IMMT, KIAA0368, MACO1, MAT2A, MCAT, METTL8, MKI67, MSL1, MST1R, MYO1E, NCLN, NF2[10], NUDT18, PATJ, PDF, PFN1, PHGDH, RAB14[10], RAD50, RAD51, RBM42, SEMA3B, SLC27A2, SLC7A1, SNUPN, TAGLN2, TAP1, TCTN2[49], TCTN3[49], TMEM17[49], TMEM216, TPM4, TRIR, USP24, ZNF598, ZW10
86.	SPART	ACSL3, ACSL4, AIFM1[384], APP[56], APPL1[385], CETN2, CISD3, CLTC[384], COPA, COQ9, DYNC1H1, EPS15, HADHA, HECW2[42], HSPA5[384], HSPA9[384], HSPD1[384], HTT[386] , IST1[387], ITCH, KIF5B[384], KLC2[384], KPNB1, LMNB1, MAP3K1[388], MED20, NCL, PLIN3, POLK, PPP6R1, SEC16A2, SMAD2[262], SMURF1[42], SMURF2, TUBA1A, UBC[163], VIM[384], WWOX, WWP1, WWP2, ZFYVE9
87.	SPAST	ALB[51], AP3B1, ATL1[98], ATP6AP1, ATP6V0A1, ATP6V0D1, ATP6V1A, ATP6V1B2, ATP6VG1, CAPN7, CAV1, CCDC115, CD2AP[10], CENPF, CHMP1A[10], CHMP1B[387], CHMP2A[42], CHMP2B[10], CHMP3, CHMP4B[10], CHMP5[10], CLTA[10], CLTC[10], CPM, CTSZ, ELAVL1[54], EMX2, ERLIN1[10], FLOT1, FLOT2, FOSL2, GPBP1, HECW2[225], HIST2H2AB, HNF4A[32], HOXA10, IST1[387], KIDINS220, LCK, LIG1, LMTK2, LYPLAL1, MBOAT7, MISP, MITD1, MRPS10, MVB12A, N6AMT1, NECTIN3, NLRP3, NUP107, NUP133, NUP43, NUP85, NUP98, ONECUT1, PARP10, PDCD6, PDCD6IP, PTPN23[10], RAD9A, RNF111, RTN2, SARAF, SEMA3B, SNTB2, SOAT1[10], SRBD1, SS18L2, STN1, STOM[10], TGS1, TM9SF1, TMED9, TSG101[10], TUBA3E, VCP[25], VPS37C, VPS4B, VPS9D1, VTA1, WDR45
88.	SPG20	FANCD2[75], NOVA1, SLC39A3, SQSTM1
89.	SPG7	AP3S1, APLNR, APOE, ART3, CCNDBP1, CD79A, CERK, CPA5, ELF3, EMC2, FAF2[42], FAM109A, FAM174A, FANCD2[75], FLOT1, GPR55, HDAC10, HNRNPK, HTR3C, ISLR, ITM2A, JUNB, KHDRBS2, KIF1BP[10], KRT40[2], KRTAP10-3[2], KRTAP10-7[2], KRTAP10-9[2], KRTAP10-12, KRTAP5-9, LOC100996763/NOTCH2NL[2], LPAR4, LPCAT4, LRIF1, LZTS2[2], MAIP1, MAPK6, MAS1, MDFI[2], MTUS2[2], NDC80, NDUFB9, PDK1, PLSCR1[7], PNMA1, PSME3, PSTPIP1, RALY, RBPMS[2], RIF1, RING1, SDF4, SLC2A12, SPATS1, STAMBP, TPX2, TRIM28
90.	SPG11	No interaction found
91.	TECPR2	ALB[10], ANTXR1, BLMH, C1orf198, CDC42BPB, DNAJB5, DST, GABARAP, GABARAPL1, GABARAPL2, MAP1LC3C, MSH5,

		MYH10, NAPG, NUDCD3, PPM1A, PUF60, RAD54L, VPS16, VPS18, VPS41, WDFY3, WDR70[10]
92.	TFG	ABI3BP, AMER2, ANXA1, ANXA11, ARHGEF16, ARL15, ASB18, ATP5PD, ATP6V0A1, ATP6V1B2, AURKB, BICC1, BOLL, BRCA1[134], CAND1[12], CEP126, CEP55, CFTR[70], CHMP1A[10], CHMP1B[10], CHMP2A[10], CHMP3, CHMP4A, CHMP4B[10], CHMP5[10], CHST14, CLINT1, CLTA[10], COPS5[12], COPS6, CRMP1, CRYAB, CSTF2, CUL1[12], CUL2[12], CUL3[12], CUL4A[12], CUL4B, CUL5, CUL7[69], DCUN1D1, DGCR8, DPM3, DSG4, DUSP14, EIF1AX, ERG28, ERGIC1, EWSR1[2], FANCD2[75], FAU, FBXL6, FBXO11, FLOT2, GBP2, GPRASP1, GRB2[146], HEPHL1, HIST1H2AG, HNRNPCL1/HNRNPCL2, HNRNPF, HSPA5[389], IFI16, IGHG1, IKBKG[390], IST1[10], ITSN1, KLHL13, LRRC15, MAGED1, MAP3K3[88], MAPK13[151], MAPK1IP1L, MCM2[84], MCRIP2, MITD1, MTUS2[10], MYOT, NAT10, NCOA4, NDC80, NEDD8[12], NEPRO, NFATC2[391], NIPBL, NTRK1[21], PDCD6IP, PEF1, PHF20, PIN1, PKP1, PLBD2, PLSCR1[7], PPP1CA[392], PSEN1[319], PTPN6, RAD21, RARS[10], RBPMS[2], RCAN2, RCL1, RIPPLY2, RNF135, RPL10A, RPL4, S100A3, S100A7, SCLY, SCP2, SEC13, SEC24A, SEC31A, SELENBP1, SNX3[10], SPG21, SPINK5, SS18L1, STAT5B, TANK, TMEM17[393], TOP1MT, TOP2A, TRAF3, TRIM25[394], TRIM68, TSG101[10], UMAD1, UNK[63], URB1, VOPP1, VPS37C, VSIG8, YWHAZ[91]
93.	TRPV4	AQP4, AQP5, CTNNB1[395], FYN[396], MAP7
94.	TUBB4A	GOLGA2, HSF1[397], HSPA8[398], KSR1[83], LRRK2, NSD2, SREBF1[399], STAU1[400], YWHAG[38]
95.	UBAP1	TSG101[401] , TNIP2, VPS28, VPS37A
96.	UCHL1	ADRA2A, ADRA2B, ADRA2C, ADRB2[402], AKT1[371], AKT2, ANG, APP[403], ATG5[37], CBX1, CCDC14, CDK1[404], CDK2[11], CDK4, CDK5[404], CDK6, CDKN1B, CDKN2A, COPS5[405], CTNNB1[406], CUL7[69], DDB1, DERL1[407], DLST[72], DUB, EGFR[408], EIF1B, EIF6, ERN1, GABRA2[15], HSP90AA1[404], HSPA8[404], HTT[409] , IKBKE[37], IQCB1[255], KCNMA1[202], KRT17, KRT4, LAMP2, MCC[37], MDM2[410], MTNR1A[155], N-alpha-ubiquitinyl-L-lysine, NCAM1[411], NEDD8[162], NMRAL1, NTRK1[21], NTRK2, phosphatidic acid[412], PLA2G2A, PMAIP1, polygalacturonase, PRKN[413], PTOV1, RANBP9[405], REST, RPTOR, SHARPIN, SMN1/SMN2, SNCA[414], SOD1[72], SOX4, TERF2[415], TERF2IP, TINF2, TNFRSF1A[416], TNIK, TP53[415], TRAF3, TRAF6[37], TRIM54[417], TRIM63[417], TUBA1A, UBB[162], UBC[418], UBE2I, Ubiquitin[419], USP15, USP21[417], USP28, VHL[37]
97.	USP8	AKT1[420], BACE, BIRC6, CBL, CBY1, CD2AP[10], CD83, CDC25C, CDH1[306], CFLAR, CHMP1A[421], CHMP1B[421], CHMP2A[422], CHMP2B[422], CHMP4B[422], CHMP4C, CHMP5[422], CHMP6, CHMP7, Dnajb3, DNM2, DUB, EEA1, EGFR[423], EPAS1, EPS15, ERBB4, FZD4, GAD, GRAP2, GRB2[424], HIF1A[425], HIST2H2AC,

		HIST2H2BE, IRF3, IRF5, IRF7, KCNN4, KIF23, KRT31[426], KRT36, KRT85, LCP2, LRIG1, MAGEL2, MCPH1[427], NBR1, NTRK1[21], Otub1, OTUB1, PJA1, PLA2G2A, PSMD4, PTEN[428], PTPN23[429], RAB3IP, RASGRF1, RNF128, RNF41, SCNN1A, SCNN1B, SCNN1G, SFN, SMO, SNCA[430], SPP1, SRC, STAM, STAM2, TARDBP[431], TRIM54[417], TRIM55, TRIM63[417], UBC[421], USP15, USP21[417], USP22, USP28, USP8, VAMP8, YWHAB[37], YWHAE[303], YWHAG[37], YWHAQ[37], YWHAZ[37]
98.	VCP	26s Proteasome, ACACA, ACAP2, ACTB[128], ACTN1, ADD1, ADRB2[9], AKAP12, Akt, AKT1[432], ALDH18A1, ALDOA, ALMS1, ALS2, AMFR[433], ANAPC7, ANKHD1/ANKHD1-EIF4EBP3, ANKLE2, ANKRD13A, ANKRD13B, ANKRD13D, ANKZF1, ANXA2, ANXA5, ANXA7, AP1B1, AP2A2, APOA1[434], APOB, APPL1[25], AR[435], ARF6, ARFGAP2, ARFGEF2, ARHGAP17, ARHGEF2, ARIH1, ARIH2, ARPC2, ARRB1, ARRB2, ASPSCR1, ATF7IP, ATG5[2], ATG9A, ATM, ATR, ATXN1, ATXN10, ATXN3, ATXN7, AUP1, AVPR2, BAD, BAG2, BAG6, BAIAP2L1, BAX[25], BCAR3, BCCIP, BCLAF1, BID, BRAT1, BRCA1[134], BRSK2, BSG, BTRC[436], BUD23, BZW2, C11orf68, CAAP1, CAB39, CACNA1C, CANX[25], Casp12, CASP7, CASP9, CASR, CAV1, CB-5083, CCDC134, CCDC8[69], CCHCR1, CCNB1, CCT2[198], CCT3[25], CCT4[25], CCT5[25], CCT6A[25], CCT7[25], CCT8[25], CD247, CD3D, CD4[437], CDC25A, CDC27, CDC37, CDC42EP1, CDC42EP4, CDC73[138], CDK1[25], CDK2[128], CDK2AP1, CDK4, CDKN1A, CDKN1B, CDKN2AIP, CEBPA[438], CENPH, CEP162, CEP19, CEP55, CEP85, CFTR[439], CHEK1, CHEK2, CHMP4B[10], CHRNA3, CIDEC, CIP2A, CLASP1, Clathrin, CLN6, CLTA[440], CLTC[441], CLUAP1, CNOT10, CNOT2, CNOT8, CNOT9, COG4, COG5, COIL, COMMD1, COMMD6, COMT, COPE, COPS3, COPS5[12], COPS7A, COQ8A, CRBN, CRMP1, CRY2[140], CSK, CSNK2B[25], CTNNA1, CTNNBL1, CTNND1, CUL1[436], CUL2[12], CUL3[12], CUL4A[442], CUL5, CUL7[25], CYLD[73], CYP3A7-CYP3A51P, DAP3, DCAF7[128], DDIAS, DDX54, DERL1[439], DERL2, DGAT2, DGCR6/LOC102724770, DIAPH3, DIO2, DLD, DNAJB1, DNAJB11, DNAJB9, DNAJC10, DNM1L, DNM3, DOCK7, DSP, DTNB, DUSP1, DUSP9, DYNC1I2, DYNC1LI1, ECD, EEA1, EED[196], EEF1A2, EIF3E, EIF4A2, EIF5A, ELAVL1[443], EPOR, EPPK1, ERCC6, ERCC8, ERI3, ERLIN2, ESPL1, ESR1[335], ESS2, EZH2[13], F7, FAF1, FAF2[444], FAM104A, FAM189B, FANCD2[75], FANCI, FBF1, FBXL2, FBXO2, FBXO32[245], FBXO6[445,446], FBXW11[10], FCHSD2, FERMT2, FHOD1, FKBP15, FMR1[78], FN1[447], FOXP3[144], FUS[14], FXR1, G3BP1[448], G3BP2, GABRA1, GBF1, GET4, GGA1, GGA2, GIGYF2, GLB1, GNPNAT1, GOLPH3L, GRB2[448], GRIN2D, GRWD1, GTF3C1, GTF3C3, GTF3C5, GZMK, H2AFJ, H2AFV, HAUS1, HDAC5[16], HDAC6[419], HDLBP, HEATR1, HEATR3, HELLS, HERPUD1, HEXIM1, HEY1,

		HIF1A[449], HIP1R, HLA-A[450], HLA-B, HLA-DRB1, HLTF[10], HMGCR, HNF1A[449,451,427,399], HNRNPA1[25], HNRNPH1[128], HNRNPH2, HNRNPH3, HOOK1, HS1BP3, HSBP1, HSF1[452], Hsp70, Hsp90, HSP90AA1[25], HSP90AB1[25], HSPA1A/HSPA1B[25], HSPA4[25], HSPA5[25], HSPA8[25], HSPB1, HSPD1[128], HSPE1[25], HTRA2, HTT[25], HUWE1[25], HYOU1, ICK, IFT74, IFT88, IGHM, IKBKE[37], IL2RB, IL9R, INF2, INSIG1[453], INSIG2, IQCB1[25], IQGAP1, IQGAP2, IQGAP3, IRS4, ISG15[205], ITGA4[81], ITGB1[445], ITGB4, ITPR1[194], ITPR3, JAK3, KCMF1, KCNMA1[202], KDM3A, KDM3B, KIAA1468, KIAA2013, KIF1BP[25], KIF20A, KLC2[10], L3MBTL1, LMAN1, LMNA[25], LMNB2, LNX1, LRIG1, LYAR, MAP2K1, MAP3K3[88], MAP7D3, MAPK1[25], MAPK13[25], MAPK3[25], MAPK8IP2, MAPT, MARK2, MCC[37], MCM2[84], MDM2[25], MDN1, MFN2, MMP12, MRPS18B, MRPS23, MSH4, MTOR, NAPA, NBEA, NCAPD2, NCAPG, NCAPH, NCDN[25], NCOA1, NCSTN, NDRG1, NEK2, NEPRO, NF1, NFE2L2[454], NFKB1, NFKB2, NFKBIA[446], NFKBIB, NFKBIE, NGLY1, NIPSNAP1[128], NIPSNAP2, NMD3, NME2, NOS2[157], NPHP4, NPLOC4, NPM1[227], NSF, NSFL1C[455], NTRK1[21], NUB1, NUDT21, NUMA1, NUP107, NUP205, NUP54, NUP58, NUP62, OBSL1[69], ONECUT1, OPTN, OS9, OTULIN, PACRG, PARK7 [264,253,244], PDCD10, PDCD4, PDCD6, PDK3[10], PDXDC1, PEX19[456], PFN1, PHKG2, PIK3C2B, PIK3R2[25], PIK3R3[448], PKD2, PKM, PKN2[25], PLAA, PLEC, PLK1, PLPP3, PNO1, POLR2A, POLR2B, POLR3C, PPFIBP1, PPM1B, PPP1CA[457], PPP1CC[392], PPP1R18, Ppp2c, PPP2CA[458], PPP2CB, PPP2R1A, PPP2R1B, PPP2R2A, PPP3CA[128], PPP6C[25], PPT1, PRKAA1[459], PRKAG1, PRKAR2A, PRKCD, PRKDC, PRKN[42], PRPF19, PRPF31, PRPF4, PSAP, PSMA1, PSMA2, PSMA6, PSMA7, PSMC1, PSMC4, PSMC5, PSMD4, PTCRA, PTEN[158], PTGS2, PTPN22, PTPN23[25], PTPN3, PTPN9, PTPRO, RAB10, RAB11B, RAB14[25], RAB3GAP1, RAB3GAP2, RAB7A[25], RABGAP1, RAD23A, RAF1[25], RARA[274], RBFOX2, RBM15B, RBM23, RFC3, RFC5, RHBDD1, RHBDL3, RIF1, RNF103, RNF126, RNF19A, RNF2[25], RNF31, RNF7, RNF8, RPA2[460], RPL13, RPL18A, RPL22, RPL23, RPL24, RPL30, RPL6 RPL9, RPN1, RPS11, RPS13, RPS25, RPS3, RPS3A, RPS4X, RPS6, RPS6KA1, RPS8, RPS9, RRBP1, RRP12, RSU1, RUVBL2, RXRB, RYR2, SAP18, SCAMP2[10], SCD, SCFD1, SCHIP1, SDCCAG3, SEC16A, SEC22B, SEC61A1, SELENOK, SELENOS, SEM1, SENP3, SERPINA1, SESN2, SFTPC, SGK1, SH2D1A, SH2D2A, SIK2, SIRT7[22], SKP1, SLC17A2, SLC38A10, SLC3A2, SLIRP, SLX4, SMAD1, SMARCA5, SMARCC1, SMC2, SMC4, SMURF1[263], SNX3[25], SOD1[72], SON, SPAST[25], SPC24, SPRTN, SPTAN1, SRRM2[160], SRSF11, SRSF3, ST13, STAG2, STAT1, STAT5a/b[461], STMN1, STUB1[462], STX5, STXBP5L, SUMO1, SUMO2, SUPT16H, SUPT6H, SUZ12[222], SVIP, SYF2,

		SYMPK, SYVN1[463], TAF6L, TARDBP[464], TAX1BP1, TBC1D10B, TBC1D9B, TCP1[25], TELO2, TERF2[25], TFE3, TIMM44, TMED10, TMEM129, TMEM33, TMEM67, TMPRSS13, TNFRSF14, TNKS1BP1, TNPO3, TOM1, TOM1L1, TOMM34, TOP1, TP53[25], TP53BP1, TP63, TPD52L2, TRA, TRAF6[37], TRIM13, TRIM21, TRIM25[25], TRIO, TRIP12, TSG101[25], TSN, TTC26, TTC4, TTK, TUBA1C[128], TUBB[128], TUBB4B, TUBGCP2, U2AF2[267,256,[247], UBA5, UBB[25], UBC[465], UBD, UBE2J1, UBE2M, UBE2S, UBE4A, UBE4B, UBL4A[466], UBL7, UBOX5, UBQLN1, UBR1, UBR4, UBR5, UBR7, UBXN1, UBXN10, UBXN11, UBXN2A, UBXN2B, UBXN4, UBXN6, UBXN7, UBXN8, UFD1, ULK3, UPP1, USP10, USP13, UTRN, VAPA[379], VAPB[379], VBP1, VCAM1[78], VCL, VCP[467], VCPIP1, VCPKMT, vesnarinone, VHL[449], VIL1, VIM[25], VPS18, VPS50, VPS53, WAC, WAPL, WDR43, WDR82, WDYHV1, WNK1, WRAP73, WRN, WRNIP1, YAP1[90], YOD1, YWHAB[25], YWHAE[25], YWHAG[25], YWHAH[25], YWHAQ[25], YWHAZ[25], ZFAND2B, ZFR, ZNF326, ZNF778
99.	VPS37A	ALG2, APP[56], ARRDC1, ESCRT1, KDM1A, MVB12A, MVB12B, TRIM42, TSG101[2], UBAP1, UBC[287], VPS28, XPO1[50]
100.	WDR48	AHCYL1, AHCYL2[426], AP3M1, AR[468], ARHGEF10, ARID2, ATAD5, BCR[426][426][426][426][426][426][426][424][426][402][374], BIRC6, BOP1, BRCA1[426], C11orf58, CCT2[10], CCT3[10], CCT4[10], CCT5[10], CCT6A[10], CCT7[10], CCT8[10], CDK10, CEP350, COL11A2, COPS8, CRTAP, CSNK2A1, CSNK2A2, CSKN2B, CTNND1, CUL3[469], CUL4B, DKC1, DMWD, EIF3A, EIF3F, EIF3H, EZR[426], FAM122B, FANCI, FBXL7, FBXW5, GMPS, GPALPP1, GPN1[426], GPN3, GPS1, GRN, H3F3A/H3F3B, HSPA4[67], HSPH1, ILKAP, ITCH, IWS1, JMJD6, KDM4A, LTV1, MYCBP2, NAF1, NELFE, NFATC1, NFATC2[19], NHP2, NOLC1, NOP10, NPTX1, NTRK1[21], OPTN, ORC3, P3H1, PAXIP1, PCNA, PDHA1[426], PHLPP1, PHLPP2, PKN2[426], POLR1C, POLR2G, POLR2I, PPIB, PPP1R9A, PPP1R9B, PSMA3[259], PSME3[426,424,i 402,374], RAD51, RAD51AP1, RASSF8, RDX, RNF19B, RPL3, SET, SHQ1, SLC9A3R1, SLC9A3R2, SNX27, STK11, SUPT6H, SURF2, TARBP1, TCOF1, TCP1[10], TP53[426], TRMT11, TSPYL6, TUBB1, UBC[470], UBR5, UBXN1, UIMC1, UNC13B, USH1C, USP1, USP12, USP46, VAT1, VSX2, WDR20, WDR70[426], WRN, YLPM1, YWHAB[426], YWHAE[303], YWHAH[426], ZBTB2, ZBTB33, ZNF639
101.	ZFR	ADARB1, APOBEC3D, BMI1, BRCA1[134], CAND1[12], CBX8, CCDC8[69], CDX1, CEBPA[438], COPS5[12], CUL3[12], CUL7[69], DLG4[281], EED[13], EGFR[282], ELAVL1[54], ELAVL2, ETS1, FBXW11[42], FGF8, FMR1[294], FOXB1, FOXE1, FOXL2, FOXP3[471], FOXQ1, FOXS1, HDAC11[201], HNRNPA1[42], HNRNPR, IFIT5, ILF2, KRR1, MEPCE, MOV10[6], NANOG, NXF1[6], OBSL1[69], PCED1B, PDE4DIP, PRPF38B, RASGRF2,

		RBM4B, RNF2[13], RNPS1, RPA1, RPA2[277], RPA3, RPAP1[139], SIRT7[22], SMAD2[262], STAU1[23], TARDBP[472], TLN1, TRA2A, TRIM25[89], USP33, USP45, VCP[25], YBX1, ZNF169, ZYX
102.	ZFYVE26	ABHD5, ALAS1, CELF2, CEP44, CSK, CSNK1D, CTNNA2, DAB1, FARP1, GOLGA2, HNRNPC, ITIH6, KIAA1522, KRT15, KRT40[2], KRTAP10-3[2], KRTAP10-7[2], KRTAP10-9[2], LRP1[10], MDFI[2], NOP58, NSFL1C[10], OPRM1, PAPSS1, PCBP2, phosphatidylinositol-3-phosphate, PLD3, PNMA1, PNMA5, PNPLA8, PPP6C[10], PRICKLE3, PYM1, RABGAP1L, RAD52, RAD54L2, SAV1, SMC3, SPAG5, SSX2IP, TADA2A, TDO2, TFIP11, TRAF1[2], TRIM21, TYW3, USHBP1, USP11, ZC3H8
103.	ZFYVE27	ACAT1, ACP2, ADGRL1, APOB, APP[56], ARL8A, ASPH, ATL1[100], ATP1A1, ATP1A2, ATP1A3, ATP1B1, ATP1B2, ATP2A1, ATP2A2[100], ATP2B1, ATP2B2, ATP2B4, ATP5F1A[42], ATP5F1B, ATP5F1C[42], ATP5PB[42], BDH1, BRI3BP, C1QBP[100], CANX[100], CCDC136, CCT3[164], CCT4[164], CCT5[164], Cdc42, CDS2, COPB2, COPG2, CPT1C, CYP46A1, DNAJA2, DPP6, ERP44, FA2H, FKBP8[474], GAD1, GNA11, GNA13, GNAI1, GNAI2, GNAO1, GNAQ, GNB1[100], GNB2, GOLGA5, GPM6A, GPR37L1, GPX4, GRIA2[100], HACD3[100], HK1, HMOX2, HSPA9[100], KIF5A, KIF5B[100], KIF5C[100], MAP6D1, MBOAT2, MFN1, MOSPD2, MT-ATP8, NCAM1[100], NCDN[100], NCEH1, NLGN2, NLGN3, NSF, OPALIN, P2RY12, PGRMC1, phosphatidylinositol 3,4-diphosphate, phosphatidylinositol 4,5-diphosphate, phosphatidylinositol 3,4,5-triphosphate, PIP4P2, PLD3, PLP1[100], PLXDC2, PPP3CA[100], PRKACA[100], PRKACB, PRKAR2B, PSMD11, PURA, RAB10, RAB11A, RAB2A, RAB2B, RAB33B, RAB39B, RAB3A, RAB3B, RAB3GAP2, RAB6A, RAB7A[475], RCN2, REEP5[100], RPS5, RTN1, RTN3, RTN4[100], SCAMP5, SDCCAG3, SDHA, SEPT5, SLC19A2, SLC1A3, SLC1A6, SLC25A22, SLC3A2, SLC6A11, SLC7A2, SNAP25, SORT1, SURF4, SYNGR1, SYNGR2, SYNGR3, SYP, TIMM50[100,99,97], TMEM184B, TUBAL3, VAPA[164], VAPB[164], YWHAE[100], YWHAZ[100], ZFAND2B, ZFYVE27[100]

1. Gloeckner, C.J.; Mayerhofer, P.U.; Landgraf, P.; Muntau, A.C.; Holzinger, A.; Gerber, J.K.; Kammerer, S.; Adamski, J.; Roscher, A.A. Human adrenoleukodystrophy protein and related peroxisomal ABC transporters interact with the peroxisomal assembly protein PEX19p. *Biochem Biophys Res Commun* **2000**, *271*, 144-150, doi:10.1006/bbrc.2000.2572.
2. Rolland, T.; Tasan, M.; Charloteaux, B.; Pevzner, S.J.; Zhong, Q.; Sahni, N.; Yi, S.; Lemmens, I.; Fontanillo, C.; Mosca, R.; et al. A proteome-scale map of the human interactome network. *Cell* **2014**, *159*, 1212-1226, doi:10.1016/j.cell.2014.10.050.
3. Yu, H.; Tardivo, L.; Tam, S.; Weiner, E.; Gebreab, F.; Fan, C.; Svrzikapa, N.; Hirozane-Kishikawa, T.; Rietman, E.; Yang, X.; et al. Next-generation sequencing to generate interactome datasets. *Nat Methods* **2011**, *8*, 478-480, doi:10.1038/nmeth.1597.

4. Zeitlmann, L.; Sirim, P.; Kremmer, E.; Kolanus, W. Cloning of ACP33 as a novel intracellular ligand of CD4. *J Biol Chem* **2001**, *276*, 9123-9132, doi:10.1074/jbc.M009270200.
5. Dittmer, T.A.; Sahni, N.; Kubben, N.; Hill, D.E.; Vidal, M.; Burgess, R.C.; Roukos, V.; Misteli, T. Systematic identification of pathological lamin A interactors. *Mol Biol Cell* **2014**, *25*, 1493-1510, doi:10.1091/mbc.E14-02-0733.
6. Castello, A.; Fischer, B.; Eichelbaum, K.; Horos, R.; Beckmann, B.M.; Strein, C.; Davey, N.E.; Humphreys, D.T.; Preiss, T.; Steinmetz, L.M.; et al. Insights into RNA biology from an atlas of mammalian mRNA-binding proteins. *Cell* **2012**, *149*, 1393-1406, doi:10.1016/j.cell.2012.04.031.
7. Rual, J.F.; Venkatesan, K.; Hao, T.; Hirozane-Kishikawa, T.; Dricot, A.; Li, N.; Berriz, G.F.; Gibbons, F.D.; Dreze, M.; Ayivi-Guedehoussou, N.; et al. Towards a proteome-scale map of the human protein-protein interaction network. *Nature* **2005**, *437*, 1173-1178, doi:10.1038/nature04209.
8. Powell, M.J.; Casimiro, M.C.; Cordon-Cardo, C.; He, X.; Yeow, W.S.; Wang, C.; McCue, P.A.; McBurney, M.W.; Pestell, R.G. Disruption of a Sirt1-dependent autophagy checkpoint in the prostate results in prostatic intraepithelial neoplasia lesion formation. *Cancer Res* **2011**, *71*, 964-975, doi:10.1158/0008-5472.CAN-10-3172.
9. Roy, S.J.; Glazkova, I.; Frechette, L.; Iorio-Morin, C.; Binda, C.; Petrin, D.; Trieu, P.; Robitaille, M.; Angers, S.; Hebert, T.E.; et al. Novel, gel-free proteomics approach identifies RNF5 and JAMP as modulators of GPCR stability. *Mol Endocrinol* **2013**, *27*, 1245-1266, doi:10.1210/me.2013-1091.
10. Hein, M.Y.; Hubner, N.C.; Poser, I.; Cox, J.; Nagaraj, N.; Toyoda, Y.; Gak, I.A.; Weisswange, I.; Mansfeld, J.; Buchholz, F.; et al. A human interactome in three quantitative dimensions organized by stoichiometries and abundances. *Cell* **2015**, *163*, 712-723, doi:10.1016/j.cell.2015.09.053.
11. Neganova, I.; Vilella, F.; Atkinson, S.P.; Lloret, M.; Passos, J.F.; von Zglinicki, T.; O'Connor, J.E.; Burks, D.; Jones, R.; Armstrong, L.; et al. An important role for CDK2 in G1 to S checkpoint activation and DNA damage response in human embryonic stem cells. *Stem Cells* **2011**, *29*, 651-659, doi:10.1002/stem.620.
12. Bennett, E.J.; Rush, J.; Gygi, S.P.; Harper, J.W. Dynamics of cullin-RING ubiquitin ligase network revealed by systematic quantitative proteomics. *Cell* **2010**, *143*, 951-965, doi:10.1016/j.cell.2010.11.017.
13. Cao, Q.; Wang, X.; Zhao, M.; Yang, R.; Malik, R.; Qiao, Y.; Poliakov, A.; Yocum, A.K.; Li, Y.; Chen, W.; et al. The central role of EED in the orchestration of polycomb group complexes. *Nat Commun* **2014**, *5*, 3127, doi:10.1038/ncomms4127.
14. Jain, S.; Wheeler, J.R.; Walters, R.W.; Agrawal, A.; Barsic, A.; Parker, R. ATPase-Modulated Stress Granules Contain a Diverse Proteome and Substructure. *Cell* **2016**, *164*, 487-498, doi:10.1016/j.cell.2015.12.038.
15. Nakamura, Y.; Morrow, D.H.; Modgil, A.; Huyghe, D.; Deeb, T.Z.; Lumb, M.J.; Davies, P.A.; Moss, S.J. Proteomic Characterization of Inhibitory Synapses Using a Novel pHluorin-tagged gamma-Aminobutyric Acid Receptor, Type A (GABAA), alpha2 Subunit Knock-in Mouse. *J Biol Chem* **2016**, *291*, 12394-12407, doi:10.1074/jbc.M116.724443.
16. Greco, T.M.; Yu, F.; Guise, A.J.; Cristea, I.M. Nuclear import of histone deacetylase 5 by requisite nuclear localization signal phosphorylation. *Mol Cell Proteomics* **2011**, *10*, M110 004317, doi:10.1074/mcp.M110.004317.
17. Richter, R.; Rorbach, J.; Pajak, A.; Smith, P.M.; Wessels, H.J.; Huynen, M.A.; Smeitink, J.A.; Lightowlers, R.N.; Chrzanowska-Lightowlers, Z.M. A functional peptidyl-tRNA hydrolase, ICT1, has been recruited into the human mitochondrial ribosome. *EMBO J* **2010**, *29*, 1116-1125, doi:10.1038/emboj.2010.14.
18. Koch, H.B.; Zhang, R.; Verdoodt, B.; Bailey, A.; Zhang, C.D.; Yates, J.R., 3rd; Menssen, A.; Hermeking, H. Large-scale identification of c-MYC-associated proteins using a combined TAP/MudPIT approach. *Cell Cycle* **2007**, *6*, 205-217, doi:10.4161/cc.6.2.3742.

19. Gabriel, C.H.; Gross, F.; Karl, M.; Stephanowitz, H.; Hennig, A.F.; Weber, M.; Gryzik, S.; Bachmann, I.; Hecklau, K.; Wienands, J.; et al. Identification of Novel Nuclear Factor of Activated T Cell (NFAT)-associated Proteins in T Cells. *J Biol Chem* **2016**, *291*, 24172-24187, doi:10.1074/jbc.M116.739326.
20. Lempiainen, J.K.; Niskanen, E.A.; Vuoti, K.M.; Lampinen, R.E.; Goos, H.; Varjosalo, M.; Palvimo, J.J. Agonist-specific Protein Interactomes of Glucocorticoid and Androgen Receptor as Revealed by Proximity Mapping. *Mol Cell Proteomics* **2017**, *16*, 1462-1474, doi:10.1074/mcp.M117.067488.
21. Emdal, K.B.; Pedersen, A.K.; Bekker-Jensen, D.B.; Tsafou, K.P.; Horn, H.; Lindner, S.; Schulte, J.H.; Eggert, A.; Jensen, L.J.; Francavilla, C.; et al. Temporal proteomics of NGF-TrkA signaling identifies an inhibitory role for the E3 ligase Cbl-b in neuroblastoma cell differentiation. *Sci Signal* **2015**, *8*, ra40, doi:10.1126/scisignal.2005769.
22. Tsai, Y.C.; Greco, T.M.; Boonmee, A.; Miteva, Y.; Cristea, I.M. Functional proteomics establishes the interaction of SIRT7 with chromatin remodeling complexes and expands its role in regulation of RNA polymerase I transcription. *Mol Cell Proteomics* **2012**, *11*, 60-76, doi:10.1074/mcp.A111.015156.
23. Milev, M.P.; Ravichandran, M.; Khan, M.F.; Schriemer, D.C.; Mouland, A.J. Characterization of staufen1 ribonucleoproteins by mass spectrometry and biochemical analyses reveal the presence of diverse host proteins associated with human immunodeficiency virus type 1. *Front Microbiol* **2012**, *3*, 367, doi:10.3389/fmicb.2012.00367.
24. Teachenor, R.; Beck, K.; Wright, L.Y.; Shen, Z.; Briggs, S.P.; Murre, C. Biochemical and phosphoproteomic analysis of the helix-loop-helix protein E47. *Mol Cell Biol* **2012**, *32*, 1671-1682, doi:10.1128/MCB.06452-11.
25. Yu, C.C.; Yang, J.C.; Chang, Y.C.; Chuang, J.G.; Lin, C.W.; Wu, M.S.; Chow, L.P. VCP phosphorylation-dependent interaction partners prevent apoptosis in Helicobacter pylori-infected gastric epithelial cells. *PLoS One* **2013**, *8*, e55724, doi:10.1371/journal.pone.0055724.
26. Jirawatnotai, S.; Hu, Y.; Michowski, W.; Elias, J.E.; Becks, L.; Bienvenu, F.; Zagozdzon, A.; Goswami, T.; Wang, Y.E.; Clark, A.B.; et al. A function for cyclin D1 in DNA repair uncovered by protein interactome analyses in human cancers. *Nature* **2011**, *474*, 230-234, doi:10.1038/nature10155.
27. Schwartz, D.R.; Wu, R.; Kardia, S.L.; Levin, A.M.; Huang, C.C.; Shedden, K.A.; Kuick, R.; Misek, D.E.; Hanash, S.M.; Taylor, J.M.; et al. Novel candidate targets of beta-catenin/T-cell factor signaling identified by gene expression profiling of ovarian endometrioid adenocarcinomas. *Cancer Res* **2003**, *63*, 2913-2922.
28. Sanderson, L.M.; Degenhardt, T.; Koppen, A.; Kalkhoven, E.; Desvergne, B.; Muller, M.; Kersten, S. Peroxisome proliferator-activated receptor beta/delta (PPARbeta/delta) but not PPARalpha serves as a plasma free fatty acid sensor in liver. *Mol Cell Biol* **2009**, *29*, 6257-6267, doi:10.1128/MCB.00370-09.
29. Clodfelter, K.H.; Holloway, M.G.; Hodor, P.; Park, S.H.; Ray, W.J.; Waxman, D.J. Sex-dependent liver gene expression is extensive and largely dependent upon signal transducer and activator of transcription 5b (STAT5b): STAT5b-dependent activation of male genes and repression of female genes revealed by microarray analysis. *Mol Endocrinol* **2006**, *20*, 1333-1351, doi:10.1210/me.2005-0489.
30. Boj, S.F.; van Es, J.H.; Huch, M.; Li, V.S.; Jose, A.; Hatzis, P.; Mokry, M.; Haegebarth, A.; van den Born, M.; Chambon, P.; et al. Diabetes risk gene and Wnt effector Tcf7l2/TCF4 controls hepatic response to perinatal and adult metabolic demand. *Cell* **2012**, *151*, 1595-1607, doi:10.1016/j.cell.2012.10.053.
31. Taipale, M.; Tucker, G.; Peng, J.; Krykbaeva, I.; Lin, Z.Y.; Larsen, B.; Choi, H.; Berger, B.; Gingras, A.C.; Lindquist, S. A quantitative chaperone interaction network reveals the architecture of cellular protein homeostasis pathways. *Cell* **2014**, *158*, 434-448, doi:10.1016/j.cell.2014.05.039.

32. Odom, D.T.; Zizlsperger, N.; Gordon, D.B.; Bell, G.W.; Rinaldi, N.J.; Murray, H.L.; Volkert, T.L.; Schreiber, J.; Rolfe, P.A.; Gifford, D.K.; et al. Control of pancreas and liver gene expression by HNF transcription factors. *Science* **2004**, *303*, 1378-1381, doi:10.1126/science.1089769.
33. Topp, J.D.; Gray, N.W.; Gerard, R.D.; Horazdovsky, B.F. Alsin is a Rab5 and Rac1 guanine nucleotide exchange factor. *J Biol Chem* **2004**, *279*, 24612-24623, doi:10.1074/jbc.M313504200.
34. Otomo, A.; Kunita, R.; Suzuki-Utsunomiya, K.; Ikeda, J.E.; Hadano, S. Defective relocalization of ALS2/alsin missense mutants to Rac1-induced macropinosomes accounts for loss of their cellular function and leads to disturbed amphisome formation. *FEBS Lett* **2011**, *585*, 730-736, doi:10.1016/j.febslet.2011.01.045.
35. Govek, E.E.; Newey, S.E.; Van Aelst, L. The role of the Rho GTPases in neuronal development. *Genes Dev* **2005**, *19*, 1-49, doi:10.1101/gad.1256405.
36. Kanekura, K.; Hashimoto, Y.; Niikura, T.; Aiso, S.; Matsuoka, M.; Nishimoto, I. Alsin, the product of ALS2 gene, suppresses SOD1 mutant neurotoxicity through RhoGEF domain by interacting with SOD1 mutants. *J Biol Chem* **2004**, *279*, 19247-19256, doi:10.1074/jbc.M313236200.
37. Ewing, R.M.; Chu, P.; Elisma, F.; Li, H.; Taylor, P.; Climie, S.; McBroom-Cerajewski, L.; Robinson, M.D.; O'Connor, L.; Li, M.; et al. Large-scale mapping of human protein-protein interactions by mass spectrometry. *Mol Syst Biol* **2007**, *3*, 89, doi:10.1038/msb4100134.
38. Jin, J.; Smith, F.D.; Stark, C.; Wells, C.D.; Fawcett, J.P.; Kulkarni, S.; Metalnikov, P.; O'Donnell, P.; Taylor, P.; Taylor, L.; et al. Proteomic, functional, and domain-based analysis of in vivo 14-3-3 binding proteins involved in cytoskeletal regulation and cellular organization. *Curr Biol* **2004**, *14*, 1436-1450, doi:10.1016/j.cub.2004.07.051.
39. Kwak, M.K.; Wakabayashi, N.; Itoh, K.; Motohashi, H.; Yamamoto, M.; Kensler, T.W. Modulation of gene expression by cancer chemopreventive dithiolethiones through the Keap1-Nrf2 pathway. Identification of novel gene clusters for cell survival. *J Biol Chem* **2003**, *278*, 8135-8145, doi:10.1074/jbc.M211898200.
40. Kleijnen, M.F.; Shih, A.H.; Zhou, P.; Kumar, S.; Soccio, R.E.; Kedersha, N.L.; Gill, G.; Howley, P.M. The hPLIC proteins may provide a link between the ubiquitination machinery and the proteasome. *Mol Cell* **2000**, *6*, 409-419, doi:10.1016/s1097-2765(00)00040-x.
41. Gorini, G.; Ponomareva, O.; Shores, K.S.; Person, M.D.; Harris, R.A.; Mayfield, R.D. Dynamin-1 co-associates with native mouse brain BKCa channels: proteomics analysis of synaptic protein complexes. *FEBS Lett* **2010**, *584*, 845-851, doi:10.1016/j.febslet.2009.12.061.
42. Huttlin, E.L.; Bruckner, R.J.; Paulo, J.A.; Cannon, J.R.; Ting, L.; Baltier, K.; Colby, G.; Gebreab, F.; Gygi, M.P.; Parzen, H.; et al. Architecture of the human interactome defines protein communities and disease networks. *Nature* **2017**, *545*, 505-509, doi:10.1038/nature22366.
43. Lee, O.H.; Kim, H.; He, Q.; Baek, H.J.; Yang, D.; Chen, L.Y.; Liang, J.; Chae, H.K.; Safari, A.; Liu, D.; et al. Genome-wide YFP fluorescence complementation screen identifies new regulators for telomere signaling in human cells. *Mol Cell Proteomics* **2011**, *10*, M110 001628, doi:10.1074/mcp.M110.001628.
44. Boldt, K.; van Reeuwijk, J.; Lu, Q.; Koutroumpas, K.; Nguyen, T.M.; Texier, Y.; van Beersum, S.E.; Horn, N.; Willer, J.R.; Mans, D.A.; et al. An organelle-specific protein landscape identifies novel diseases and molecular mechanisms. *Nat Commun* **2016**, *7*, 11491, doi:10.1038/ncomms11491.
45. Couzens, A.L.; Knight, J.D.; Kean, M.J.; Teo, G.; Weiss, A.; Dunham, W.H.; Lin, Z.Y.; Bagshaw, R.D.; Sicheri, F.; Pawson, T.; et al. Protein interaction network of the mammalian Hippo pathway reveals mechanisms of kinase-phosphatase interactions. *Sci Signal* **2013**, *6*, rs15, doi:10.1126/scisignal.2004712.
46. Simmen, T.; Honing, S.; Icking, A.; Tikkanen, R.; Hunziker, W. AP-4 binds basolateral signals and participates in basolateral sorting in epithelial MDCK cells. *Nat Cell Biol* **2002**, *4*, 154-159, doi:10.1038/ncb745.

47. Hirst, J.; Bright, N.A.; Rous, B.; Robinson, M.S. Characterization of a fourth adaptor-related protein complex. *Mol Biol Cell* **1999**, *10*, 2787-2802, doi:10.1091/mbc.10.8.2787.
48. Dell'Angelica, E.C.; Mullins, C.; Bonifacino, J.S. AP-4, a novel protein complex related to clathrin adaptors. *J Biol Chem* **1999**, *274*, 7278-7285.
49. Gupta, G.D.; Coyaud, E.; Goncalves, J.; Mojarad, B.A.; Liu, Y.; Wu, Q.; Gheiratmand, L.; Comartin, D.; Tkach, J.M.; Cheung, S.W.; et al. A Dynamic Protein Interaction Landscape of the Human Centrosome-Cilium Interface. *Cell* **2015**, *163*, 1484-1499, doi:10.1016/j.cell.2015.10.065.
50. Kirli, K.; Karaca, S.; Dehne, H.J.; Samwer, M.; Pan, K.T.; Lenz, C.; Urlaub, H.; Gorlich, D. A deep proteomics perspective on CRM1-mediated nuclear export and nucleocytoplasmic partitioning. *Elife* **2015**, *4*, doi:10.7554/eLife.11466.
51. Zhou, M.; Lucas, D.A.; Chan, K.C.; Issaq, H.J.; Petricoin, E.F., 3rd; Liotta, L.A.; Veenstra, T.D.; Conrads, T.P. An investigation into the human serum "interactome". *Electrophoresis* **2004**, *25*, 1289-1298, doi:10.1002/elps.200405866.
52. Takatsu, H.; Futatsumori, M.; Yoshino, K.; Yoshida, Y.; Shin, H.W.; Nakayama, K. Similar subunit interactions contribute to assembly of clathrin adaptor complexes and COPI complex: analysis using yeast three-hybrid system. *Biochem Biophys Res Commun* **2001**, *284*, 1083-1089, doi:10.1006/bbrc.2001.5081.
53. Boehm, M.; Aguilar, R.C.; Bonifacino, J.S. Functional and physical interactions of the adaptor protein complex AP-4 with ADP-ribosylation factors (ARFs). *EMBO J* **2001**, *20*, 6265-6276, doi:10.1093/emboj/20.22.6265.
54. Abdelmohsen, K.; Srikantan, S.; Yang, X.; Lal, A.; Kim, H.H.; Kuwano, Y.; Galban, S.; Becker, K.G.; Kamara, D.; de Cabo, R.; et al. Ubiquitin-mediated proteolysis of HuR by heat shock. *EMBO J* **2009**, *28*, 1271-1282, doi:10.1038/emboj.2009.67.
55. Huttlin, E.L.; Ting, L.; Bruckner, R.J.; Gebreab, F.; Gygi, M.P.; Szpyt, J.; Tam, S.; Zarraga, G.; Colby, G.; Baltier, K.; et al. The BioPlex Network: A Systematic Exploration of the Human Interactome. *Cell* **2015**, *162*, 425-440, doi:10.1016/j.cell.2015.06.043.
56. Olah, J.; Vincze, O.; Virok, D.; Simon, D.; Bozso, Z.; Tokesi, N.; Horvath, I.; Hlavanda, E.; Kovacs, J.; Magyar, A.; et al. Interactions of pathological hallmark proteins: tubulin polymerization promoting protein/p25, beta-amyloid, and alpha-synuclein. *J Biol Chem* **2011**, *286*, 34088-34100, doi:10.1074/jbc.M111.243907.
57. Rinn, J.L.; Wang, J.K.; Allen, N.; Brugmann, S.A.; Mikels, A.J.; Liu, H.; Ridky, T.W.; Stadler, H.S.; Nusse, R.; Helms, J.A.; et al. A dermal HOX transcriptional program regulates site-specific epidermal fate. *Genes Dev* **2008**, *22*, 303-307, doi:10.1101/gad.1610508.
58. Li, S.; Wang, L.; Berman, M.; Kong, Y.Y.; Dorf, M.E. Mapping a dynamic innate immunity protein interaction network regulating type I interferon production. *Immunity* **2011**, *35*, 426-440, doi:10.1016/j.immuni.2011.06.014.
59. Christianson, J.C.; Olzmann, J.A.; Shaler, T.A.; Sowa, M.E.; Bennett, E.J.; Richter, C.M.; Tyler, R.E.; Greenblatt, E.J.; Harper, J.W.; Kopito, R.R. Defining human ERAD networks through an integrative mapping strategy. *Nat Cell Biol* **2011**, *14*, 93-105, doi:10.1038/ncb2383.
60. Venkatesan, K.; Rual, J.F.; Vazquez, A.; Stelzl, U.; Lemmens, I.; Hirozane-Kishikawa, T.; Hao, T.; Zenkner, M.; Xin, X.; Goh, K.I.; et al. An empirical framework for binary interactome mapping. *Nat Methods* **2009**, *6*, 83-90, doi:10.1038/nmeth.1280.
61. Guo, Q.M.; Malek, R.L.; Kim, S.; Chiao, C.; He, M.; Ruffy, M.; Sanka, K.; Lee, N.H.; Dang, C.V.; Liu, E.T. Identification of c-myc responsive genes using rat cDNA microarray. *Cancer Res* **2000**, *60*, 5922-5928.
62. Yang, X.; Coulombe-Huntington, J.; Kang, S.; Sheynkman, G.M.; Hao, T.; Richardson, A.; Sun, S.; Yang, F.; Shen, Y.A.; Murray, R.R.; et al. Widespread Expansion of Protein Interaction Capabilities by Alternative Splicing. *Cell* **2016**, *164*, 805-817, doi:10.1016/j.cell.2016.01.029.

63. Murn, J.; Zarnack, K.; Yang, Y.J.; Durak, O.; Murphy, E.A.; Cheloufi, S.; Gonzalez, D.M.; Teplova, M.; Curk, T.; Zuber, J.; et al. Control of a neuronal morphology program by an RNA-binding zinc finger protein, Unkempt. *Genes Dev* **2015**, *29*, 501-512, doi:10.1101/gad.258483.115.
64. Meek, S.E.; Lane, W.S.; Piwnica-Worms, H. Comprehensive proteomic analysis of interphase and mitotic 14-3-3-binding proteins. *J Biol Chem* **2004**, *279*, 32046-32054, doi:10.1074/jbc.M403044200.
65. Palomero, T.; Odom, D.T.; O'Neil, J.; Ferrando, A.A.; Margolin, A.; Neuberg, D.S.; Winter, S.S.; Larson, R.S.; Li, W.; Liu, X.S.; et al. Transcriptional regulatory networks downstream of TAL1/SCL in T-cell acute lymphoblastic leukemia. *Blood* **2006**, *108*, 986-992, doi:10.1182/blood-2005-08-3482.
66. West, A.P.; Brodsky, I.E.; Rahner, C.; Woo, D.K.; Erdjument-Bromage, H.; Tempst, P.; Walsh, M.C.; Choi, Y.; Shadel, G.S.; Ghosh, S. TLR signalling augments macrophage bactericidal activity through mitochondrial ROS. *Nature* **2011**, *472*, 476-480, doi:10.1038/nature09973.
67. Yadav, L.; Tamene, F.; Goos, H.; van Drogen, A.; Katainen, R.; Aebersold, R.; Gstaiger, M.; Varjosalo, M. Systematic Analysis of Human Protein Phosphatase Interactions and Dynamics. *Cell Syst* **2017**, *4*, 430-444 e435, doi:10.1016/j.cels.2017.02.011.
68. Hauri, S.; Wepf, A.; van Drogen, A.; Varjosalo, M.; Tapon, N.; Aebersold, R.; Gstaiger, M. Interaction proteome of human Hippo signaling: modular control of the co-activator YAP1. *Mol Syst Biol* **2013**, *9*, 713, doi:10.1002/msb.201304750.
69. Hanson, D.; Stevens, A.; Murray, P.G.; Black, G.C.; Clayton, P.E. Identifying biological pathways that underlie primordial short stature using network analysis. *J Mol Endocrinol* **2014**, *52*, 333-344, doi:10.1530/JME-14-0029.
70. Wang, X.; Venable, J.; LaPointe, P.; Hutt, D.M.; Koulov, A.V.; Coppinger, J.; Gurkan, C.; Kellner, W.; Matteson, J.; Plutner, H.; et al. Hsp90 cochaperone Aha1 downregulation rescues misfolding of CFTR in cystic fibrosis. *Cell* **2006**, *127*, 803-815, doi:10.1016/j.cell.2006.09.043.
71. Chassefeyre, R.; Martinez-Hernandez, J.; Bertaso, F.; Bouquier, N.; Blot, B.; Laporte, M.; Fraboulet, S.; Coute, Y.; Devoy, A.; Isaacs, A.M.; et al. Regulation of postsynaptic function by the dementia-related ESCRT-III subunit CHMP2B. *J Neurosci* **2015**, *35*, 3155-3173, doi:10.1523/JNEUROSCI.0586-14.2015.
72. Malty, R.H.; Aoki, H.; Kumar, A.; Phanse, S.; Amin, S.; Zhang, Q.; Minic, Z.; Goebels, F.; Musso, G.; Wu, Z.; et al. A Map of Human Mitochondrial Protein Interactions Linked to Neurodegeneration Reveals New Mechanisms of Redox Homeostasis and NF-kappaB Signaling. *Cell Syst* **2017**, *5*, 564-577 e512, doi:10.1016/j.cels.2017.10.010.
73. Elliott, P.R.; Leske, D.; Hrdinka, M.; Bagola, K.; Fiil, B.K.; McLaughlin, S.H.; Wagstaff, J.; Volkmar, N.; Christianson, J.C.; Kessler, B.M.; et al. SPATA2 Links CYLD to LUBAC, Activates CYLD, and Controls LUBAC Signaling. *Mol Cell* **2016**, *63*, 990-1005, doi:10.1016/j.molcel.2016.08.001.
74. Tarallo, R.; Bamundo, A.; Nassa, G.; Nola, E.; Paris, O.; Ambrosino, C.; Facchiano, A.; Baumann, M.; Nyman, T.A.; Weisz, A. Identification of proteins associated with ligand-activated estrogen receptor alpha in human breast cancer cell nuclei by tandem affinity purification and nano LC-MS/MS. *Proteomics* **2011**, *11*, 172-179, doi:10.1002/pmic.201000217.
75. Zhang, T.; Du, W.; Wilson, A.F.; Namekawa, S.H.; Andreassen, P.R.; Meetei, A.R.; Pang, Q. Fancd2 in vivo interaction network reveals a non-canonical role in mitochondrial function. *Sci Rep* **2017**, *7*, 45626, doi:10.1038/srep45626.
76. Teixeira, F.R.; Yokoo, S.; Gartner, C.A.; Manfiolli, A.O.; Baqui, M.M.; Assmann, E.M.; Maragno, A.L.; Yu, H.; de Lanerolle, P.; Kobarg, J.; et al. Identification of FBXO25-interacting proteins using an integrated proteomics approach. *Proteomics* **2010**, *10*, 2746-2757, doi:10.1002/pmic.200900419.
77. Liu, B.; Zheng, Y.; Wang, T.D.; Xu, H.Z.; Xia, L.; Zhang, J.; Wu, Y.L.; Chen, G.Q.; Wang, L.S. Proteomic identification of common SCF ubiquitin ligase FBXO6-interacting glycoproteins in three kinds of cells. *J Proteome Res* **2012**, *11*, 1773-1781, doi:10.1021/pr2010204.

78. Humphries, J.D.; Byron, A.; Bass, M.D.; Craig, S.E.; Pinney, J.W.; Knight, D.; Humphries, M.J. Proteomic analysis of integrin-associated complexes identifies RCC2 as a dual regulator of Rac1 and Arf6. *Sci Signal* **2009**, *2*, ra51, doi:10.1126/scisignal.2000396.
79. Johnson-Kerner, B.L.; Garcia Diaz, A.; Ekins, S.; Wichterle, H. Kelch Domain of Gigaxonin Interacts with Intermediate Filament Proteins Affected in Giant Axonal Neuropathy. *PLoS One* **2015**, *10*, e0140157, doi:10.1371/journal.pone.0140157.
80. Varjosalo, M.; Sacco, R.; Stukalov, A.; van Drogen, A.; Planyavsky, M.; Hauri, S.; Aebersold, R.; Bennett, K.L.; Colinge, J.; Gstaiger, M.; et al. Interlaboratory reproducibility of large-scale human protein-complex analysis by standardized AP-MS. *Nat Methods* **2013**, *10*, 307-314, doi:10.1038/nmeth.2400.
81. Byron, A.; Humphries, J.D.; Craig, S.E.; Knight, D.; Humphries, M.J. Proteomic analysis of alpha4beta1 integrin adhesion complexes reveals alpha-subunit-dependent protein recruitment. *Proteomics* **2012**, *12*, 2107-2114, doi:10.1002/pmic.201100487.
82. Yang, G.; Holl, T.M.; Liu, Y.; Li, Y.; Lu, X.; Nicely, N.I.; Kepler, T.B.; Alam, S.M.; Liao, H.X.; Cain, D.W.; et al. Identification of autoantigens recognized by the 2F5 and 4E10 broadly neutralizing HIV-1 antibodies. *J Exp Med* **2013**, *210*, 241-256, doi:10.1084/jem.20121977.
83. Zhou, L.; Lyons-Rimmer, J.; Ammoun, S.; Muller, J.; Lasonder, E.; Sharma, V.; Ercolano, E.; Hilton, D.; Taiwo, I.; Barczyk, M.; et al. The scaffold protein KSR1, a novel therapeutic target for the treatment of Merlin-deficient tumors. *Oncogene* **2016**, *35*, 3443-3453, doi:10.1038/onc.2015.404.
84. Drissi, R.; Dubois, M.L.; Douziech, M.; Boisvert, F.M. Quantitative Proteomics Reveals Dynamic Interactions of the Minichromosome Maintenance Complex (MCM) in the Cellular Response to Etoposide Induced DNA Damage. *Mol Cell Proteomics* **2015**, *14*, 2002-2013, doi:10.1074/mcp.M115.048991.
85. Watson, J.D.; Oster, S.K.; Shago, M.; Khosravi, F.; Penn, L.Z. Identifying genes regulated in a Myc-dependent manner. *J Biol Chem* **2002**, *277*, 36921-36930, doi:10.1074/jbc.M201493200.
86. Glatter, T.; Wepf, A.; Aebersold, R.; Gstaiger, M. An integrated workflow for charting the human interaction proteome: insights into the PP2A system. *Mol Syst Biol* **2009**, *5*, 237, doi:10.1038/msb.2008.75.
87. Zanon, A.; Rakovic, A.; Blankenburg, H.; Doncheva, N.T.; Schwienbacher, C.; Serafin, A.; Alexa, A.; Weichenberger, C.X.; Albrecht, M.; Klein, C.; et al. Profiling of Parkin-binding partners using tandem affinity purification. *PLoS One* **2013**, *8*, e78648, doi:10.1371/journal.pone.0078648.
88. Bouwmeester, T.; Bauch, A.; Ruffner, H.; Angrand, P.O.; Bergamini, G.; Croughton, K.; Cruciat, C.; Eberhard, D.; Gagneur, J.; Ghidelli, S.; et al. A physical and functional map of the human TNF-alpha/NF-kappa B signal transduction pathway. *Nat Cell Biol* **2004**, *6*, 97-105, doi:10.1038/ncb1086.
89. Choudhury, N.R.; Heikel, G.; Trubitsyna, M.; Kubik, P.; Nowak, J.S.; Webb, S.; Granneman, S.; Spanos, C.; Rappsilber, J.; Castello, A.; et al. RNA-binding activity of TRIM25 is mediated by its PRY/SPRY domain and is required for ubiquitination. *BMC Biol* **2017**, *15*, 105, doi:10.1186/s12915-017-0444-9.
90. Enzo, E.; Santinon, G.; Pocaterra, A.; Aragona, M.; Bresolin, S.; Forcato, M.; Grifoni, D.; Pession, A.; Zanconato, F.; Guzzo, G.; et al. Aerobic glycolysis tunes YAP/TAZ transcriptional activity. *EMBO J* **2015**, *34*, 1349-1370, doi:10.15252/embj.201490379.
91. Angrand, P.O.; Segura, I.; Volkel, P.; Ghidelli, S.; Terry, R.; Brajenovic, M.; Vintersten, K.; Klein, R.; Superti-Furga, G.; Drewes, G.; et al. Transgenic mouse proteomics identifies new 14-3-3-associated proteins involved in cytoskeletal rearrangements and cell signaling. *Mol Cell Proteomics* **2006**, *5*, 2211-2227, doi:10.1074/mcp.M600147-MCP200.
92. So, J.; Pasculescu, A.; Dai, A.Y.; Williton, K.; James, A.; Nguyen, V.; Creixell, P.; Schoof, E.M.; Sinclair, J.; Barrios-Rodiles, M.; et al. Integrative analysis of kinase networks in TRAIL-induced

apoptosis provides a source of potential targets for combination therapy. *Sci Signal* **2015**, *8*, rs3, doi:10.1126/scisignal.2005700.
93. Byrnes, L.J.; Singh, A.; Szeto, K.; Benvin, N.M.; O'Donnell, J.P.; Zipfel, W.R.; Sondermann, H. Structural basis for conformational switching and GTP loading of the large G protein atlastin. *EMBO J* **2013**, *32*, 369-384, doi:10.1038/emboj.2012.353.
94. Liu, T.Y.; Bian, X.; Sun, S.; Hu, X.; Klemm, R.W.; Prinz, W.A.; Rapoport, T.A.; Hu, J. Lipid interaction of the C terminus and association of the transmembrane segments facilitate atlastin-mediated homotypic endoplasmic reticulum fusion. *Proc Natl Acad Sci U S A* **2012**, *109*, E2146-2154, doi:10.1073/pnas.1208385109.
95. Zhu, P.P.; Patterson, A.; Lavoie, B.; Stadler, J.; Shoeb, M.; Patel, R.; Blackstone, C. Cellular localization, oligomerization, and membrane association of the hereditary spastic paraplegia 3A (SPG3A) protein atlastin. *J Biol Chem* **2003**, *278*, 49063-49071, doi:10.1074/jbc.M306702200.
96. Hu, J.; Shibata, Y.; Zhu, P.P.; Voss, C.; Rismanchi, N.; Prinz, W.A.; Rapoport, T.A.; Blackstone, C. A class of dynamin-like GTPases involved in the generation of the tubular ER network. *Cell* **2009**, *138*, 549-561, doi:10.1016/j.cell.2009.05.025.
97. Park, S.H.; Zhu, P.P.; Parker, R.L.; Blackstone, C. Hereditary spastic paraplegia proteins REEP1, spastin, and atlastin-1 coordinate microtubule interactions with the tubular ER network. *J Clin Invest* **2010**, *120*, 1097-1110, doi:10.1172/JCI40979.
98. Evans, K.; Keller, C.; Pavur, K.; Glasgow, K.; Conn, B.; Lauring, B. Interaction of two hereditary spastic paraplegia gene products, spastin and atlastin, suggests a common pathway for axonal maintenance. *Proc Natl Acad Sci U S A* **2006**, *103*, 10666-10671, doi:10.1073/pnas.0510863103.
99. Namekawa, M.; Muriel, M.P.; Janer, A.; Latouche, M.; Dauphin, A.; Debeir, T.; Martin, E.; Duyckaerts, C.; Prigent, A.; Depienne, C.; et al. Mutations in the SPG3A gene encoding the GTPase atlastin interfere with vesicle trafficking in the ER/Golgi interface and Golgi morphogenesis. *Mol Cell Neurosci* **2007**, *35*, 1-13, doi:10.1016/j.mcn.2007.01.012.
100. Hashimoto, Y.; Shirane, M.; Matsuzaki, F.; Saita, S.; Ohnishi, T.; Nakayama, K.I. Protrudin regulates endoplasmic reticulum morphology and function associated with the pathogenesis of hereditary spastic paraplegia. *J Biol Chem* **2014**, *289*, 12946-12961, doi:10.1074/jbc.M113.528687.
101. Chang, J.; Lee, S.; Blackstone, C. Protrudin binds atlastins and endoplasmic reticulum-shaping proteins and regulates network formation. *Proc Natl Acad Sci U S A* **2013**, *110*, 14954-14959, doi:10.1073/pnas.1307391110.
102. Usenovic, M.; Knight, A.L.; Ray, A.; Wong, V.; Brown, K.R.; Caldwell, G.A.; Caldwell, K.A.; Stagljar, I.; Krainc, D. Identification of novel ATP13A2 interactors and their role in alpha-synuclein misfolding and toxicity. *Hum Mol Genet* **2012**, *21*, 3785-3794, doi:10.1093/hmg/dds206.
103. Splinter, D.; Razafsky, D.S.; Schlager, M.A.; Serra-Marques, A.; Grigoriev, I.; Demmers, J.; Keijzer, N.; Jiang, K.; Poser, I.; Hyman, A.A.; et al. BICD2, dynactin, and LIS1 cooperate in regulating dynein recruitment to cellular structures. *Mol Biol Cell* **2012**, *23*, 4226-4241, doi:10.1091/mbc.E12-03-0210.
104. Campaner, S.; Spreafico, F.; Burgold, T.; Doni, M.; Rosato, U.; Amati, B.; Testa, G. The methyltransferase Set7/9 (Setd7) is dispensable for the p53-mediated DNA damage response in vivo. *Mol Cell* **2011**, *43*, 681-688, doi:10.1016/j.molcel.2011.08.007.
105. Redwine, W.B.; DeSantis, M.E.; Hollyer, I.; Htet, Z.M.; Tran, P.T.; Swanson, S.K.; Florens, L.; Washburn, M.P.; Reck-Peterson, S.L. The human cytoplasmic dynein interactome reveals novel activators of motility. *Elife* **2017**, *6*, doi:10.7554/eLife.28257.
106. Ito, D.; Suzuki, N. Molecular pathogenesis of seipin/BSCL2-related motor neuron diseases. *Ann Neurol* **2007**, *61*, 237-250, doi:10.1002/ana.21070.

107. Wang, J.; Huo, K.; Ma, L.; Tang, L.; Li, D.; Huang, X.; Yuan, Y.; Li, C.; Wang, W.; Guan, W.; et al. Toward an understanding of the protein interaction network of the human liver. *Mol Syst Biol* **2011**, *7*, 536, doi:10.1038/msb.2011.67.
108. Fenner, B.J.; Scannell, M.; Prehn, J.H. Expanding the substantial interactome of NEMO using protein microarrays. *PLoS One* **2010**, *5*, e8799, doi:10.1371/journal.pone.0008799.
109. Chung, K.Y.; Day, P.W.; Velez-Ruiz, G.; Sunahara, R.K.; Kobilka, B.K. Identification of GPCR-interacting cytosolic proteins using HDL particles and mass spectrometry-based proteomic approach. *PLoS One* **2013**, *8*, e54942, doi:10.1371/journal.pone.0054942.
110. Polster, B.M.; Basanez, G.; Etxebarria, A.; Hardwick, J.M.; Nicholls, D.G. Calpain I induces cleavage and release of apoptosis-inducing factor from isolated mitochondria. *J Biol Chem* **2005**, *280*, 6447-6454, doi:10.1074/jbc.M413269200.
111. Cao, G.; Xing, J.; Xiao, X.; Liou, A.K.; Gao, Y.; Yin, X.M.; Clark, R.S.; Graham, S.H.; Chen, J. Critical role of calpain I in mitochondrial release of apoptosis-inducing factor in ischemic neuronal injury. *J Neurosci* **2007**, *27*, 9278-9293, doi:10.1523/JNEUROSCI.2826-07.2007.
112. Lei, H.Y.; Zhou, X.L.; Ruan, Z.R.; Sun, W.C.; Eriani, G.; Wang, E.D. Calpain Cleaves Most Components in the Multiple Aminoacyl-tRNA Synthetase Complex and Affects Their Functions. *J Biol Chem* **2015**, *290*, 26314-26327, doi:10.1074/jbc.M115.681999.
113. Siman, R.; Card, J.P.; Davis, L.G. Proteolytic processing of beta-amyloid precursor by calpain I. *J Neurosci* **1990**, *10*, 2400-2411.
114. Yousefi, S.; Perozzo, R.; Schmid, I.; Ziemiecki, A.; Schaffner, T.; Scapozza, L.; Brunner, T.; Simon, H.U. Calpain-mediated cleavage of Atg5 switches autophagy to apoptosis. *Nat Cell Biol* **2006**, *8*, 1124-1132, doi:10.1038/ncb1482.
115. Wood, D.E.; Thomas, A.; Devi, L.A.; Berman, Y.; Beavis, R.C.; Reed, J.C.; Newcomb, E.W. Bax cleavage is mediated by calpain during drug-induced apoptosis. *Oncogene* **1998**, *17*, 1069-1078, doi:10.1038/sj.onc.1202034.
116. Gil-Parrado, S.; Fernandez-Montalvan, A.; Assfalg-Machleidt, I.; Popp, O.; Bestvater, F.; Holloschi, A.; Knoch, T.A.; Auerswald, E.A.; Welsh, K.; Reed, J.C.; et al. Ionomycin-activated calpain triggers apoptosis. A probable role for Bcl-2 family members. *J Biol Chem* **2002**, *277*, 27217-27226, doi:10.1074/jbc.M202945200.
117. Seo, J.; Giusti-Rodriguez, P.; Zhou, Y.; Rudenko, A.; Cho, S.; Ota, K.T.; Park, C.; Patzke, H.; Madabhushi, R.; Pan, L.; et al. Activity-dependent p25 generation regulates synaptic plasticity and Abeta-induced cognitive impairment. *Cell* **2014**, *157*, 486-498, doi:10.1016/j.cell.2014.01.065.
118. Jiang, L.Q.; Wen, S.J.; Wang, H.Y.; Chen, L.Y. Screening the proteins that interact with calpain in a human heart cDNA library using a yeast two-hybrid system. *Hypertens Res* **2002**, *25*, 647-652.
119. Lin, Y.M.; Ono, K.; Satoh, S.; Ishiguro, H.; Fujita, M.; Miwa, N.; Tanaka, T.; Tsunoda, T.; Yang, K.C.; Nakamura, Y.; et al. Identification of AF17 as a downstream gene of the beta-catenin/T-cell factor pathway and its involvement in colorectal carcinogenesis. *Cancer Res* **2001**, *61*, 6345-6349.
120. McRobert, E.A.; Young, A.N.; Bach, L.A. Advanced glycation end-products induce calpain-mediated degradation of ezrin. *FEBS J* **2012**, *279*, 3240-3250, doi:10.1111/j.1742-4658.2012.08710.x.
121. Olmos, G.; Arenas, M.I.; Bienes, R.; Calzada, M.J.; Aragones, J.; Garcia-Bermejo, M.L.; Landazuri, M.O.; Lucio-Cazana, J. 15-Deoxy-Delta(12,14)-prostaglandin-J(2) reveals a new pVHL-independent, lysosomal-dependent mechanism of HIF-1alpha degradation. *Cell Mol Life Sci* **2009**, *66*, 2167-2180, doi:10.1007/s00018-009-0039-x.
122. Averna, M.; De Tullio, R.; Pedrazzi, M.; Bavestrello, M.; Pellegrini, M.; Salamino, F.; Pontremoli, S.; Melloni, E. Interaction between calpain-1 and HSP90: new insights into the regulation of localization and activity of the protease. *PLoS One* **2015**, *10*, e0116738, doi:10.1371/journal.pone.0116738.

123. Tarlac, V.; Storey, E. Role of proteolysis in polyglutamine disorders. *J Neurosci Res* **2003**, *74*, 406-416, doi:10.1002/jnr.10746.
124. Kim, M.; Roh, J.K.; Yoon, B.W.; Kang, L.; Kim, Y.J.; Aronin, N.; DiFiglia, M. Huntingtin is degraded to small fragments by calpain after ischemic injury. *Exp Neurol* **2003**, *183*, 109-115.
125. Gafni, J.; Ellerby, L.M. Calpain activation in Huntington's disease. *J Neurosci* **2002**, *22*, 4842-4849.
126. Shumway, S.D.; Maki, M.; Miyamoto, S. The PEST domain of IkappaBalpha is necessary and sufficient for in vitro degradation by mu-calpain. *J Biol Chem* **1999**, *274*, 30874-30881.
127. Oda, A.; Wakao, H.; Fujita, H. Calpain is a signal transducer and activator of transcription (STAT) 3 and STAT5 protease. *Blood* **2002**, *99*, 1850-1852, doi:10.1182/blood.v99.5.1850.
128. Fogeron, M.L.; Muller, H.; Schade, S.; Dreher, F.; Lehmann, V.; Kuhnel, A.; Scholz, A.K.; Kashofer, K.; Zerck, A.; Fauler, B.; et al. LGALS3BP regulates centriole biogenesis and centrosome hypertrophy in cancer cells. *Nat Commun* **2013**, *4*, 1531, doi:10.1038/ncomms2517.
129. Wang, C.F.; Huang, Y.S. Calpain 2 activated through N-methyl-D-aspartic acid receptor signaling cleaves CPEB3 and abrogates CPEB3-repressed translation in neurons. *Mol Cell Biol* **2012**, *32*, 3321-3332, doi:10.1128/MCB.00296-12.
130. Yokota, S.; Yanagi, H.; Yura, T.; Kubota, H. Cytosolic chaperonin-containing t-complex polypeptide 1 changes the content of a particular subunit species concomitant with substrate binding and folding activities during the cell cycle. *Eur J Biochem* **2001**, *268*, 4664-4673.
131. McCormack, E.A.; Llorca, O.; Carrascosa, J.L.; Valpuesta, J.M.; Willison, K.R. Point mutations in a hinge linking the small and large domains of beta-actin result in trapped folding intermediates bound to cytosolic chaperonin CCT. *J Struct Biol* **2001**, *135*, 198-204, doi:10.1006/jsbi.2001.4385.
132. Hynes, G.M.; Willison, K.R. Individual subunits of the eukaryotic cytosolic chaperonin mediate interactions with binding sites located on subdomains of beta-actin. *J Biol Chem* **2000**, *275*, 18985-18994, doi:10.1074/jbc.M910297199.
133. Lau, E.; Kluger, H.; Varsano, T.; Lee, K.; Scheffler, I.; Rimm, D.L.; Ideker, T.; Ronai, Z.A. PKCepsilon promotes oncogenic functions of ATF2 in the nucleus while blocking its apoptotic function at mitochondria. *Cell* **2012**, *148*, 543-555, doi:10.1016/j.cell.2012.01.016.
134. Ertych, N.; Stolz, A.; Valerius, O.; Braus, G.H.; Bastians, H. CHK2-BRCA1 tumor-suppressor axis restrains oncogenic Aurora-A kinase to ensure proper mitotic microtubule assembly. *Proc Natl Acad Sci U S A* **2016**, *113*, 1817-1822, doi:10.1073/pnas.1525129113.
135. Coyaud, E.; Mis, M.; Laurent, E.M.; Dunham, W.H.; Couzens, A.L.; Robitaille, M.; Gingras, A.C.; Angers, S.; Raught, B. BioID-based Identification of Skp Cullin F-box (SCF)beta-TrCP1/2 E3 Ligase Substrates. *Mol Cell Proteomics* **2015**, *14*, 1781-1795, doi:10.1074/mcp.M114.045658.
136. Pichlmair, A.; Kandasamy, K.; Alvisi, G.; Mulhern, O.; Sacco, R.; Habjan, M.; Binder, M.; Stefanovic, A.; Eberle, C.A.; Goncalves, A.; et al. Viral immune modulators perturb the human molecular network by common and unique strategies. *Nature* **2012**, *487*, 486-490, doi:10.1038/nature11289.
137. Freund, A.; Zhong, F.L.; Venteicher, A.S.; Meng, Z.; Veenstra, T.D.; Frydman, J.; Artandi, S.E. Proteostatic control of telomerase function through TRiC-mediated folding of TCAB1. *Cell* **2014**, *159*, 1389-1403, doi:10.1016/j.cell.2014.10.059.
138. Herr, P.; Lundin, C.; Evers, B.; Ebner, D.; Bauerschmidt, C.; Kingham, G.; Palmai-Pallag, T.; Mortusewicz, O.; Frings, O.; Sonnhammer, E.; et al. A genome-wide IR-induced RAD51 foci RNAi screen identifies CDC73 involved in chromatin remodeling for DNA repair. *Cell Discov* **2015**, *1*, 15034, doi:10.1038/celldisc.2015.34.
139. Jeronimo, C.; Forget, D.; Bouchard, A.; Li, Q.; Chua, G.; Poitras, C.; Therien, C.; Bergeron, D.; Bourassa, S.; Greenblatt, J.; et al. Systematic analysis of the protein interaction network for the human transcription machinery reveals the identity of the 7SK capping enzyme. *Mol Cell* **2007**, *27*, 262-274, doi:10.1016/j.molcel.2007.06.027.

140. Papp, S.J.; Huber, A.L.; Jordan, S.D.; Kriebs, A.; Nguyen, M.; Moresco, J.J.; Yates, J.R.; Lamia, K.A. DNA damage shifts circadian clock time via Hausp-dependent Cry1 stabilization. *Elife* **2015**, *4*, doi:10.7554/eLife.04883.
141. Miyata, Y.; Shibata, T.; Aoshima, M.; Tsubata, T.; Nishida, E. The molecular chaperone TRiC/CCT binds to the Trp-Asp 40 (WD40) repeat protein WDR68 and promotes its folding, protein kinase DYRK1A binding, and nuclear accumulation. *J Biol Chem* **2014**, *289*, 33320-33332, doi:10.1074/jbc.M114.586115.
142. Bharti, P.; Schliebs, W.; Schievelbusch, T.; Neuhaus, A.; David, C.; Kock, K.; Herrmann, C.; Meyer, H.E.; Wiese, S.; Warscheid, B.; et al. PEX14 is required for microtubule-based peroxisome motility in human cells. *J Cell Sci* **2011**, *124*, 1759-1768, doi:10.1242/jcs.079368.
143. Hauri, S.; Comoglio, F.; Seimiya, M.; Gerstung, M.; Glatter, T.; Hansen, K.; Aebersold, R.; Paro, R.; Gstaiger, M.; Beisel, C. A High-Density Map for Navigating the Human Polycomb Complexome. *Cell Rep* **2016**, *17*, 583-595, doi:10.1016/j.celrep.2016.08.096.
144. Wang, T.; Jiang, X.; Chen, G.; Xu, J. Interaction of amyotrophic lateral sclerosis/frontotemporal lobar degeneration-associated fused-in-sarcoma with proteins involved in metabolic and protein degradation pathways. *Neurobiol Aging* **2015**, *36*, 527-535, doi:10.1016/j.neurobiolaging.2014.07.044.
145. Howlett, A.C.; Gray, A.J.; Hunter, J.M.; Willardson, B.M. Role of molecular chaperones in G protein beta5/regulator of G protein signaling dimer assembly and G protein betagamma dimer specificity. *J Biol Chem* **2009**, *284*, 16386-16399, doi:10.1074/jbc.M900800200.
146. Brehme, M.; Hantschel, O.; Colinge, J.; Kaupe, I.; Planyavsky, M.; Kocher, T.; Mechtler, K.; Bennett, K.L.; Superti-Furga, G. Charting the molecular network of the drug target Bcr-Abl. *Proc Natl Acad Sci U S A* **2009**, *106*, 7414-7419, doi:10.1073/pnas.0900653106.
147. Watson, G.W.; Wickramasekara, S.; Fang, Y.; Maier, C.S.; Williams, D.E.; Dashwood, R.H.; Perez, V.I.; Ho, E. HDAC6 activity is not required for basal autophagic flux in metastatic prostate cancer cells. *Exp Biol Med (Maywood)* **2016**, *241*, 1177-1185, doi:10.1177/1535370215618518.
148. New, M.; Olzscha, H.; Liu, G.; Khan, O.; Stimson, L.; McGouran, J.; Kerr, D.; Coutts, A.; Kessler, B.; Middleton, M.; et al. A regulatory circuit that involves HR23B and HDAC6 governs the biological response to HDAC inhibitors. *Cell Death Differ* **2013**, *20*, 1306-1316, doi:10.1038/cdd.2013.47.
149. Fu, D.; Brophy, J.A.; Chan, C.T.; Atmore, K.A.; Begley, U.; Paules, R.S.; Dedon, P.C.; Begley, T.J.; Samson, L.D. Human AlkB homolog ABH8 Is a tRNA methyltransferase required for wobble uridine modification and DNA damage survival. *Mol Cell Biol* **2010**, *30*, 2449-2459, doi:10.1128/MCB.01604-09.
150. Thompson, J.W.; Nagel, J.; Hoving, S.; Gerrits, B.; Bauer, A.; Thomas, J.R.; Kirschner, M.W.; Schirle, M.; Luchansky, S.J. Quantitative Lys--Gly-Gly (diGly) proteomics coupled with inducible RNAi reveals ubiquitin-mediated proteolysis of DNA damage-inducible transcript 4 (DDIT4) by the E3 ligase HUWE1. *J Biol Chem* **2014**, *289*, 28942-28955, doi:10.1074/jbc.M114.573352.
151. Sumara, G.; Formentini, I.; Collins, S.; Sumara, I.; Windak, R.; Bodenmiller, B.; Ramracheya, R.; Caille, D.; Jiang, H.; Platt, K.A.; et al. Regulation of PKD by the MAPK p38delta in insulin secretion and glucose homeostasis. *Cell* **2009**, *136*, 235-248, doi:10.1016/j.cell.2008.11.018.
152. von Kriegsheim, A.; Baiocchi, D.; Birtwistle, M.; Sumpton, D.; Bienvenut, W.; Morrice, N.; Yamada, K.; Lamond, A.; Kalna, G.; Orton, R.; et al. Cell fate decisions are specified by the dynamic ERK interactome. *Nat Cell Biol* **2009**, *11*, 1458-1464, doi:10.1038/ncb1994.
153. Liu, X.; Zong, W.; Li, T.; Wang, Y.; Xu, X.; Zhou, Z.W.; Wang, Z.Q. The E3 ubiquitin ligase APC/C(C)(dh1) degrades MCPH1 after MCPH1-betaTrCP2-Cdc25A-mediated mitotic entry to ensure neurogenesis. *EMBO J* **2017**, *36*, 3666-3681, doi:10.15252/embj.201694443.
154. Yamauchi, T.; Nishiyama, M.; Moroishi, T.; Yumimoto, K.; Nakayama, K.I. MDM2 mediates nonproteolytic polyubiquitylation of the DEAD-Box RNA helicase DDX24. *Mol Cell Biol* **2014**, *34*, 3321-3340, doi:10.1128/MCB.00320-14.

155. Benleulmi-Chaachoua, A.; Chen, L.; Sokolina, K.; Wong, V.; Jurisica, I.; Emerit, M.B.; Darmon, M.; Espin, A.; Stagljar, I.; Tafelmeyer, P.; et al. Protein interactome mining defines melatonin MT1 receptors as integral component of presynaptic protein complexes of neurons. *J Pineal Res* **2016**, *60*, 95-108, doi:10.1111/jpi.12294.
156. Basei, F.L.; Meirelles, G.V.; Righetto, G.L.; Dos Santos Migueleti, D.L.; Smetana, J.H.; Kobarg, J. New interaction partners for Nek4.1 and Nek4.2 isoforms: from the DNA damage response to RNA splicing. *Proteome Sci* **2015**, *13*, 11, doi:10.1186/s12953-015-0065-6.
157. Foster, M.W.; Thompson, J.W.; Forrester, M.T.; Sha, Y.; McMahon, T.J.; Bowles, D.E.; Moseley, M.A.; Marshall, H.E. Proteomic analysis of the NOS2 interactome in human airway epithelial cells. *Nitric Oxide* **2013**, *34*, 37-46, doi:10.1016/j.niox.2013.02.079.
158. Ahn, Y.; Hwang, C.Y.; Lee, S.R.; Kwon, K.S.; Lee, C. The tumour suppressor PTEN mediates a negative regulation of the E3 ubiquitin-protein ligase Nedd4. *Biochem J* **2008**, *412*, 331-338, doi:10.1042/BJ20071403.
159. Backus, K.M.; Correia, B.E.; Lum, K.M.; Forli, S.; Horning, B.D.; Gonzalez-Paez, G.E.; Chatterjee, S.; Lanning, B.R.; Teijaro, J.R.; Olson, A.J.; et al. Proteome-wide covalent ligand discovery in native biological systems. *Nature* **2016**, *534*, 570-574, doi:10.1038/nature18002.
160. McCracken, S.; Longman, D.; Marcon, E.; Moens, P.; Downey, M.; Nickerson, J.A.; Jessberger, R.; Wilde, A.; Caceres, J.F.; Emili, A.; et al. Proteomic analysis of SRm160-containing complexes reveals a conserved association with cohesin. *J Biol Chem* **2005**, *280*, 42227-42236, doi:10.1074/jbc.M507410200.
161. Gingras, A.C.; Caballero, M.; Zarske, M.; Sanchez, A.; Hazbun, T.R.; Fields, S.; Sonenberg, N.; Hafen, E.; Raught, B.; Aebersold, R. A novel, evolutionarily conserved protein phosphatase complex involved in cisplatin sensitivity. *Mol Cell Proteomics* **2005**, *4*, 1725-1740, doi:10.1074/mcp.M500231-MCP200.
162. Stelzl, U.; Worm, U.; Lalowski, M.; Haenig, C.; Brembeck, F.H.; Goehler, H.; Stroedicke, M.; Zenkner, M.; Schoenherr, A.; Koeppen, S.; et al. A human protein-protein interaction network: a resource for annotating the proteome. *Cell* **2005**, *122*, 957-968, doi:10.1016/j.cell.2005.08.029.
163. Matsumoto, M.; Hatakeyama, S.; Oyamada, K.; Oda, Y.; Nishimura, T.; Nakayama, K.I. Large-scale analysis of the human ubiquitin-related proteome. *Proteomics* **2005**, *5*, 4145-4151, doi:10.1002/pmic.200401280.
164. Saita, S.; Shirane, M.; Natume, T.; Iemura, S.; Nakayama, K.I. Promotion of neurite extension by protrudin requires its interaction with vesicle-associated membrane protein-associated protein. *J Biol Chem* **2009**, *284*, 13766-13777, doi:10.1074/jbc.M807938200.
165. Lai, Y.; Song, M.; Hakala, K.; Weintraub, S.T.; Shiio, Y. Proteomic dissection of the von Hippel-Lindau (VHL) interactome. *J Proteome Res* **2011**, *10*, 5175-5182, doi:10.1021/pr200642c.
166. Darios, F.; Mochel, F.; Stevanin, G. Lipids in the Physiopathology of Hereditary Spastic Paraplegias. *Front Neurosci* **2020**, *14*, 74, doi:10.3389/fnins.2020.00074.
167. Salen, G.; Steiner, R.D. Epidemiology, diagnosis, and treatment of cerebrotendinous xanthomatosis (CTX). *J Inherit Metab Dis* **2017**, *40*, 771-781, doi:10.1007/s10545-017-0093-8.
168. Tan, X.; Behari, J.; Cieply, B.; Michalopoulos, G.K.; Monga, S.P. Conditional deletion of beta-catenin reveals its role in liver growth and regeneration. *Gastroenterology* **2006**, *131*, 1561-1572, doi:10.1053/j.gastro.2006.08.042.
169. Yamamoto, Y.; Moore, R.; Hess, H.A.; Guo, G.L.; Gonzalez, F.J.; Korach, K.S.; Maronpot, R.R.; Negishi, M. Estrogen receptor alpha mediates 17alpha-ethynylestradiol causing hepatotoxicity. *J Biol Chem* **2006**, *281*, 16625-16631, doi:10.1074/jbc.M602723200.
170. Boulias, K.; Katrakili, N.; Bamberg, K.; Underhill, P.; Greenfield, A.; Talianidis, I. Regulation of hepatic metabolic pathways by the orphan nuclear receptor SHP. *EMBO J* **2005**, *24*, 2624-2633, doi:10.1038/sj.emboj.7600728.

171. Inoue, Y.; Yu, A.M.; Yim, S.H.; Ma, X.; Krausz, K.W.; Inoue, J.; Xiang, C.C.; Brownstein, M.J.; Eggertsen, G.; Bjorkhem, I.; et al. Regulation of bile acid biosynthesis by hepatocyte nuclear factor 4alpha. *J Lipid Res* **2006**, *47*, 215-227, doi:10.1194/jlr.M500430-JLR200.
172. Holloway, M.G.; Miles, G.D.; Dombkowski, A.A.; Waxman, D.J. Liver-specific hepatocyte nuclear factor-4alpha deficiency: greater impact on gene expression in male than in female mouse liver. *Mol Endocrinol* **2008**, *22*, 1274-1286, doi:10.1210/me.2007-0564.
173. Li, X.; Massa, P.E.; Hanidu, A.; Peet, G.W.; Aro, P.; Savitt, A.; Mische, S.; Li, J.; Marcu, K.B. IKKalpha, IKKbeta, and NEMO/IKKgamma are each required for the NF-kappa B-mediated inflammatory response program. *J Biol Chem* **2002**, *277*, 45129-45140, doi:10.1074/jbc.M205165200.
174. Leuenberger, N.; Pradervand, S.; Wahli, W. Sumoylated PPARalpha mediates sex-specific gene repression and protects the liver from estrogen-induced toxicity in mice. *J Clin Invest* **2009**, *119*, 3138-3148, doi:10.1172/JCI39019.
175. Wu, Z.; Chiang, J.Y. Transcriptional regulation of human oxysterol 7 alpha-hydroxylase gene (CYP7B1) by Sp1. *Gene* **2001**, *272*, 191-197.
176. Smith, D.S.; Niethammer, M.; Ayala, R.; Zhou, Y.; Gambello, M.J.; Wynshaw-Boris, A.; Tsai, L.H. Regulation of cytoplasmic dynein behaviour and microtubule organization by mammalian Lis1. *Nat Cell Biol* **2000**, *2*, 767-775, doi:10.1038/35041000.
177. Zyss, D.; Ebrahimi, H.; Gergely, F. Casein kinase I delta controls centrosome positioning during T cell activation. *J Cell Biol* **2011**, *195*, 781-797, doi:10.1083/jcb.201106025.
178. Sahni, M.; Zhou, X.M.; Bakiri, L.; Schlessinger, J.; Baron, R.; Levy, J.B. Identification of a novel 135-kDa Grb2-binding protein in osteoclasts. *J Biol Chem* **1996**, *271*, 33141-33147, doi:10.1074/jbc.271.51.33141.
179. Ross, C.A. Huntington's disease: new paths to pathogenesis. *Cell* **2004**, *118*, 4-7, doi:10.1016/j.cell.2004.06.022.
180. Brouillet, E.; Jacquard, C.; Bizat, N.; Blum, D. 3-Nitropropionic acid: a mitochondrial toxin to uncover physiopathological mechanisms underlying striatal degeneration in Huntington's disease. *J Neurochem* **2005**, *95*, 1521-1540, doi:10.1111/j.1471-4159.2005.03515.x.
181. Yamada, M.; Toba, S.; Takitoh, T.; Yoshida, Y.; Mori, D.; Nakamura, T.; Iwane, A.H.; Yanagida, T.; Imai, H.; Yu-Lee, L.Y.; et al. mNUDC is required for plus-end-directed transport of cytoplasmic dynein and dynactins by kinesin-1. *EMBO J* **2010**, *29*, 517-531, doi:10.1038/emboj.2009.378.
182. Niu, Y.; Zhang, C.; Sun, Z.; Hong, Z.; Li, K.; Sun, D.; Yang, Y.; Tian, C.; Gong, W.; Liu, J.J. PtdIns(4)P regulates retromer-motor interaction to facilitate dynein-cargo dissociation at the trans-Golgi network. *Nat Cell Biol* **2013**, *15*, 417-429, doi:10.1038/ncb2710.
183. Yamashita, A.; Kumazawa, T.; Koga, H.; Suzuki, N.; Oka, S.; Sugiura, T. Generation of lysophosphatidylinositol by DDHD domain containing 1 (DDHD1): Possible involvement of phospholipase D/phosphatidic acid in the activation of DDHD1. *Biochim Biophys Acta* **2010**, *1801*, 711-720, doi:10.1016/j.bbalip.2010.03.012.
184. Hammond, E.; Lang, J.; Maeda, Y.; Pleasure, D.; Angus-Hill, M.; Xu, J.; Horiuchi, M.; Deng, W.; Guo, F. The Wnt effector transcription factor 7-like 2 positively regulates oligodendrocyte differentiation in a manner independent of Wnt/beta-catenin signaling. *J Neurosci* **2015**, *35*, 5007-5022, doi:10.1523/JNEUROSCI.4787-14.2015.
185. Liao, H.; Hyman, M.C.; Baek, A.E.; Fukase, K.; Pinsky, D.J. cAMP/CREB-mediated transcriptional regulation of ectonucleoside triphosphate diphosphohydrolase 1 (CD39) expression. *J Biol Chem* **2010**, *285*, 14791-14805, doi:10.1074/jbc.M110.116905.
186. Borsellino, G.; Kleinewietfeld, M.; Di Mitri, D.; Sternjak, A.; Diamantini, A.; Giometto, R.; Hopner, S.; Centonze, D.; Bernardi, G.; Dell'Acqua, M.L.; et al. Expression of ectonucleotidase CD39 by Foxp3+ Treg cells: hydrolysis of extracellular ATP and immune suppression. *Blood* **2007**, *110*, 1225-1232, doi:10.1182/blood-2006-12-064527.

187. Littman, D.R.; Rudensky, A.Y. Th17 and regulatory T cells in mediating and restraining inflammation. *Cell* **2010**, *140*, 845-858, doi:10.1016/j.cell.2010.02.021.
188. Yang, Q.; Nagano, T.; Shah, Y.; Cheung, C.; Ito, S.; Gonzalez, F.J. The PPAR alpha-humanized mouse: a model to investigate species differences in liver toxicity mediated by PPAR alpha. *Toxicol Sci* **2008**, *101*, 132-139, doi:10.1093/toxsci/kfm206.
189. Wu, Y.; Sun, X.; Kaczmarek, E.; Dwyer, K.M.; Bianchi, E.; Usheva, A.; Robson, S.C. RanBPM associates with CD39 and modulates ecto-nucleotidase activity. *Biochem J* **2006**, *396*, 23-30, doi:10.1042/BJ20051568.
190. Gloury, R.; Zotos, D.; Zuidscherwoude, M.; Masson, F.; Liao, Y.; Hasbold, J.; Corcoran, L.M.; Hodgkin, P.D.; Belz, G.T.; Shi, W.; et al. Dynamic changes in Id3 and E-protein activity orchestrate germinal center and plasma cell development. *J Exp Med* **2016**, *213*, 1095-1111, doi:10.1084/jem.20152003.
191. Jo, Y.; Sguigna, P.V.; DeBose-Boyd, R.A. Membrane-associated ubiquitin ligase complex containing gp78 mediates sterol-accelerated degradation of 3-hydroxy-3-methylglutaryl-coenzyme A reductase. *J Biol Chem* **2011**, *286*, 15022-15031, doi:10.1074/jbc.M110.211326.
192. Butnaru, C.M.; Chiritoiu, M.B.; Chiritoiu, G.N.; Petrescu, S.M.; Petrescu, A.J. Inhibition of N-glycan processing modulates the network of EDEM3 interactors. *Biochem Biophys Res Commun* **2017**, *486*, 978-984, doi:10.1016/j.bbrc.2017.03.143.
193. Huber, M.D.; Vesely, P.W.; Datta, K.; Gerace, L. Erlins restrict SREBP activation in the ER and regulate cellular cholesterol homeostasis. *J Cell Biol* **2013**, *203*, 427-436, doi:10.1083/jcb.201305076.
194. Pearce, M.M.; Wormer, D.B.; Wilkens, S.; Wojcikiewicz, R.J. An endoplasmic reticulum (ER) membrane complex composed of SPFH1 and SPFH2 mediates the ER-associated degradation of inositol 1,4,5-trisphosphate receptors. *J Biol Chem* **2009**, *284*, 10433-10445, doi:10.1074/jbc.M809801200.
195. Boldt, K.; van Reeuwijk, J.; Lu, Q.; Koutroumpas, K.; Nguyen, T.M.T.; Texier, Y.; van Beersum, S.E.C.; Horn, N.; Willer, J.R.; Mans, D.A.; et al. An organelle-specific protein landscape identifies novel diseases and molecular mechanisms. *Nature Communications* **2016**, *7*, doi:ARTN 11491
10.1038/ncomms11491.
196. Cao, Q.; Wang, X.J.; Zhao, M.; Yang, R.D.; Malik, R.; Qiao, Y.Y.; Poliakov, A.; Yocum, A.K.; Li, Y.; Chen, W.; et al. The central role of EED in the orchestration of polycomb group complexes. *Nature Communications* **2014**, *5*, doi:ARTN 3127
10.1038/ncomms4127.
197. Lyssand, J.S.; DeFino, M.C.; Tang, X.B.; Hertz, A.L.; Feller, D.B.; Wacker, J.L.; Adams, M.E.; Hague, C. Blood pressure is regulated by an alpha1D-adrenergic receptor/dystrophin signalosome. *J Biol Chem* **2008**, *283*, 18792-18800, doi:10.1074/jbc.M801860200.
198. Lin, Y.F.; Lee, Y.F.; Liang, P.H. Targeting beta-tubulin:CCT-beta complexes incurs Hsp90- and VCP-related protein degradation and induces ER stress-associated apoptosis by triggering capacitative Ca2+ entry, mitochondrial perturbation and caspase overactivation. *Cell Death Dis* **2012**, *3*, e434, doi:10.1038/cddis.2012.173.
199. Pearce, M.M.; Wang, Y.; Kelley, G.G.; Wojcikiewicz, R.J. SPFH2 mediates the endoplasmic reticulum-associated degradation of inositol 1,4,5-trisphosphate receptors and other substrates in mammalian cells. *J Biol Chem* **2007**, *282*, 20104-20115, doi:10.1074/jbc.M701862200.
200. Elzi, D.J.; Song, M.; Hakala, K.; Weintraub, S.T.; Shiio, Y. Proteomic Analysis of the EWS-Fli-1 Interactome Reveals the Role of the Lysosome in EWS-Fli-1 Turnover. *J Proteome Res* **2014**, *13*, 3783-3791, doi:10.1021/pr500387m.
201. Joshi, P.; Greco, T.M.; Guise, A.J.; Luo, Y.; Yu, F.; Nesvizhskii, A.I.; Cristea, I.M. The functional interactome landscape of the human histone deacetylase family. *Mol Syst Biol* **2013**, *9*, 672, doi:10.1038/msb.2013.26.

202. Kathiresan, T.; Harvey, M.; Orchard, S.; Sakai, Y.; Sokolowski, B. A protein interaction network for the large conductance Ca(2+)-activated K(+) channel in the mouse cochlea. *Mol Cell Proteomics* **2009**, *8*, 1972-1987, doi:10.1074/mcp.M800495-MCP200.
203. Teranishi, Y.; Hur, J.Y.; Gu, G.J.; Kihara, T.; Ishikawa, T.; Nishimura, T.; Winblad, B.; Behbahani, H.; Kamali-Moghaddam, M.; Frykman, S.; et al. Erlin-2 is associated with active gamma-secretase in brain and affects amyloid beta-peptide production. *Biochem Biophys Res Commun* **2012**, *424*, 476-481, doi:10.1016/j.bbrc.2012.06.137.
204. Xu, Y.; Cai, M.; Yang, Y.; Huang, L.; Ye, Y. SGTA recognizes a noncanonical ubiquitin-like domain in the Bag6-Ubl4A-Trc35 complex to promote endoplasmic reticulum-associated degradation. *Cell Rep* **2012**, *2*, 1633-1644, doi:10.1016/j.celrep.2012.11.010.
205. Zhao, C.; Denison, C.; Huibregtse, J.M.; Gygi, S.; Krug, R.M. Human ISG15 conjugation targets both IFN-induced and constitutively expressed proteins functioning in diverse cellular pathways. *Proc Natl Acad Sci U S A* **2005**, *102*, 10200-10205, doi:10.1073/pnas.0504754102.
206. Wheldon, L.M.; Haines, B.P.; Rajappa, R.; Mason, I.; Rigby, P.W.; Heath, J.K. Critical role of FLRT1 phosphorylation in the interdependent regulation of FLRT1 function and FGF receptor signalling. *PLoS One* **2010**, *5*, e10264, doi:10.1371/journal.pone.0010264.
207. Haines, B.P.; Wheldon, L.M.; Summerbell, D.; Heath, J.K.; Rigby, P.W. Regulated expression of FLRT genes implies a functional role in the regulation of FGF signalling during mouse development. *Dev Biol* **2006**, *297*, 14-25, doi:10.1016/j.ydbio.2006.04.004.
208. Wu, C.; Ma, M.H.; Brown, K.R.; Geisler, M.; Li, L.; Tzeng, E.; Jia, C.Y.; Jurisica, I.; Li, S.S. Systematic identification of SH3 domain-mediated human protein-protein interactions by peptide array target screening. *Proteomics* **2007**, *7*, 1775-1785, doi:10.1002/pmic.200601006.
209. Luthi-Carter, R.; Strand, A.D.; Hanson, S.A.; Kooperberg, C.; Schilling, G.; La Spada, A.R.; Merry, D.E.; Young, A.B.; Ross, C.A.; Borchelt, D.R.; et al. Polyglutamine and transcription: gene expression changes shared by DRPLA and Huntington's disease mouse models reveal context-independent effects. *Hum Mol Genet* **2002**, *11*, 1927-1937.
210. Gourfinkel-An, I.; Parain, K.; Hartmann, A.; Mangiarini, L.; Brice, A.; Bates, G.; Hirsch, E.C. Changes in GAD67 mRNA expression evidenced by in situ hybridization in the brain of R6/2 transgenic mice. *J Neurochem* **2003**, *86*, 1369-1378, doi:10.1046/j.1471-4159.2003.01916.x.
211. Wei, J.; Davis, K.M.; Wu, H.; Wu, J.Y. Protein phosphorylation of human brain glutamic acid decarboxylase (GAD)65 and GAD67 and its physiological implications. *Biochemistry* **2004**, *43*, 6182-6189, doi:10.1021/bi0496992.
212. Baptista, M.S.; Melo, C.V.; Armelao, M.; Herrmann, D.; Pimentel, D.O.; Leal, G.; Caldeira, M.V.; Bahr, B.A.; Bengtson, M.; Almeida, R.D.; et al. Role of the proteasome in excitotoxicity-induced cleavage of glutamic acid decarboxylase in cultured hippocampal neurons. *PLoS One* **2010**, *5*, e10139, doi:10.1371/journal.pone.0010139.
213. Grigoryan, T.; Stein, S.; Qi, J.; Wende, H.; Garratt, A.N.; Nave, K.A.; Birchmeier, C.; Birchmeier, W. Wnt/Rspondin/beta-catenin signals control axonal sorting and lineage progression in Schwann cell development. *Proc Natl Acad Sci U S A* **2013**, *110*, 18174-18179, doi:10.1073/pnas.1310490110.
214. Hu, X.; Dutta, P.; Tsurumi, A.; Li, J.; Wang, J.; Land, H.; Li, W.X. Unphosphorylated STAT5A stabilizes heterochromatin and suppresses tumor growth. *Proc Natl Acad Sci U S A* **2013**, *110*, 10213-10218, doi:10.1073/pnas.1221243110.
215. Salipante, S.J.; Rojas, M.E.; Korkmaz, B.; Duan, Z.; Wechsler, J.; Benson, K.F.; Person, R.E.; Grimes, H.L.; Horwitz, M.S. Contributions to neutropenia from PFAAP5 (N4BP2L2), a novel protein mediating transcriptional repressor cooperation between Gfi1 and neutrophil elastase. *Mol Cell Biol* **2009**, *29*, 4394-4405, doi:10.1128/MCB.00596-09.

216. Padmanabhan, A.; Li, X.; Bieberich, C.J. Protein kinase A regulates MYC protein through transcriptional and post-translational mechanisms in a catalytic subunit isoform-specific manner. *J Biol Chem* **2013**, *288*, 14158-14169, doi:10.1074/jbc.M112.432377.
217. Tavor, S.; Park, D.J.; Gery, S.; Vuong, P.T.; Gombart, A.F.; Koeffler, H.P. Restoration of C/EBPalpha expression in a BCR-ABL+ cell line induces terminal granulocytic differentiation. *J Biol Chem* **2003**, *278*, 52651-52659, doi:10.1074/jbc.M307077200.
218. Na, C.H.; Jones, D.R.; Yang, Y.; Wang, X.; Xu, Y.; Peng, J. Synaptic protein ubiquitination in rat brain revealed by antibody-based ubiquitome analysis. *J Proteome Res* **2012**, *11*, 4722-4732, doi:10.1021/pr300536k.
219. Lim, J.; Hao, T.; Shaw, C.; Patel, A.J.; Szabo, G.; Rual, J.F.; Fisk, C.J.; Li, N.; Smolyar, A.; Hill, D.E.; et al. A protein-protein interaction network for human inherited ataxias and disorders of Purkinje cell degeneration. *Cell* **2006**, *125*, 801-814, doi:10.1016/j.cell.2006.03.032.
220. Hoque, M.; Young, T.M.; Lee, C.G.; Serrero, G.; Mathews, M.B.; Pe'ery, T. The growth factor granulin interacts with cyclin T1 and modulates P-TEFb-dependent transcription. *Mol Cell Biol* **2003**, *23*, 1688-1702.
221. Klemmer, P.; Smit, A.B.; Li, K.W. Proteomics analysis of immuno-precipitated synaptic protein complexes. *J Proteomics* **2009**, *72*, 82-90, doi:10.1016/j.jprot.2008.10.005.
222. Cao, Q.; Wang, X.J.; Zhao, M.; Yang, R.D.; Malik, R.; Qiao, Y.Y.; Poliakov, A.; Cao, X.H.; Harris, C.; Feng, F.Y.; et al. The central role of EED in orchestration of polycomb group complexes. *Cancer Research* **2014**, *74*, doi:10.1158/1538-7445.Am2014-Lb-132.
223. Tong, J.; Taylor, P.; Moran, M.F. Proteomic analysis of the epidermal growth factor receptor (EGFR) interactome and post-translational modifications associated with receptor endocytosis in response to EGF and stress. *Mol Cell Proteomics* **2014**, *13*, 1644-1658, doi:10.1074/mcp.M114.038596.
224. Korkmaz, B.; Horwitz, M.S.; Jenne, D.E.; Gauthier, F. Neutrophil elastase, proteinase 3, and cathepsin G as therapeutic targets in human diseases. *Pharmacol Rev* **2010**, *62*, 726-759, doi:10.1124/pr.110.002733.
225. Lu, L.; Hu, S.; Wei, R.; Qiu, X.; Lu, K.; Fu, Y.; Li, H.; Xing, G.; Li, D.; Peng, R.; et al. The HECT type ubiquitin ligase NEDL2 is degraded by anaphase-promoting complex/cyclosome (APC/C)-Cdh1, and its tight regulation maintains the metaphase to anaphase transition. *J Biol Chem* **2013**, *288*, 35637-35650, doi:10.1074/jbc.M113.472076.
226. Yadav, R.; Hama, S.; Liu, Y.; Siahmansur, T.; Schofield, J.; Syed, A.A.; France, M.; Pemberton, P.; Adam, S.; Ho, J.H.; et al. Effect of Roux-en-Y Bariatric Surgery on Lipoproteins, Insulin Resistance, and Systemic and Vascular Inflammation in Obesity and Diabetes. *Front Immunol* **2017**, *8*, 1512, doi:10.3389/fimmu.2017.01512.
227. Chen, R.; Wang, Y.; Liu, Y.; Zhang, Q.; Zhang, X.; Zhang, F.; Shieh, C.H.; Yang, D.; Zhang, N. Quantitative study of the interactome of PKCzeta involved in the EGF-induced tumor cell chemotaxis. *J Proteome Res* **2013**, *12*, 1478-1486, doi:10.1021/pr3011292.
228. Pilot-Storck, F.; Chopin, E.; Rual, J.F.; Baudot, A.; Dobrokhotov, P.; Robinson-Rechavi, M.; Brun, C.; Cusick, M.E.; Hill, D.E.; Schaeffer, L.; et al. Interactome mapping of the phosphatidylinositol 3-kinase-mammalian target of rapamycin pathway identifies deformed epidermal autoregulatory factor-1 as a new glycogen synthase kinase-3 interactor. *Mol Cell Proteomics* **2010**, *9*, 1578-1593, doi:10.1074/mcp.M900568-MCP200.
229. Law, I.K.; Liu, L.; Xu, A.; Lam, K.S.; Vanhoutte, P.M.; Che, C.M.; Leung, P.T.; Wang, Y. Identification and characterization of proteins interacting with SIRT1 and SIRT3: implications in the anti-aging and metabolic effects of sirtuins. *Proteomics* **2009**, *9*, 2444-2456, doi:10.1002/pmic.200800738.

230. Jian, J.; Zhao, S.; Tian, Q.; Gonzalez-Gugel, E.; Mundra, J.J.; Uddin, S.M.; Liu, B.; Richbourgh, B.; Brunetti, R.; Liu, C.J. Progranulin directly binds to the CRD2 and CRD3 of TNFR extracellular domains. *FEBS Lett* **2013**, *587*, 3428-3436, doi:10.1016/j.febslet.2013.09.024.
231. Wu, L.X.; Xu, J.H.; Huang, X.W.; Zhang, K.Z.; Wen, C.X.; Chen, Y.Z. Down-regulation of p210(bcr/abl) by curcumin involves disrupting molecular chaperone functions of Hsp90. *Acta Pharmacol Sin* **2006**, *27*, 694-699, doi:10.1111/j.1745-7254.2006.00326.x.
232. Liu, X.; Salokas, K.; Tamene, F.; Jiu, Y.; Weldatsadik, R.G.; Ohman, T.; Varjosalo, M. An AP-MS- and BioID-compatible MAC-tag enables comprehensive mapping of protein interactions and subcellular localizations. *Nat Commun* **2018**, *9*, 1188, doi:10.1038/s41467-018-03523-2.
233. Yifrach, O.; Horovitz, A. Coupling between protein folding and allostery in the GroE chaperonin system. *Proc Natl Acad Sci U S A* **2000**, *97*, 1521-1524, doi:10.1073/pnas.040449997.
234. Cao, J.; Wang, J.; Qi, W.; Miao, H.H.; Wang, J.; Ge, L.; DeBose-Boyd, R.A.; Tang, J.J.; Li, B.L.; Song, B.L. Ufd1 is a cofactor of gp78 and plays a key role in cholesterol metabolism by regulating the stability of HMG-CoA reductase. *Cell Metab* **2007**, *6*, 115-128, doi:10.1016/j.cmet.2007.07.002.
235. Alard, J.E.; Hillion, S.; Guillevin, L.; Saraux, A.; Pers, J.O.; Youinou, P.; Jamin, C. Autoantibodies to endothelial cell surface ATP synthase, the endogenous receptor for hsp60, might play a pathogenic role in vasculatides. *PLoS One* **2011**, *6*, e14654, doi:10.1371/journal.pone.0014654.
236. Kirchhoff, S.R.; Gupta, S.; Knowlton, A.A. Cytosolic heat shock protein 60, apoptosis, and myocardial injury. *Circulation* **2002**, *105*, 2899-2904.
237. Shan, Y.X.; Liu, T.J.; Su, H.F.; Samsamshariat, A.; Mestril, R.; Wang, P.H. Hsp10 and Hsp60 modulate Bcl-2 family and mitochondria apoptosis signaling induced by doxorubicin in cardiac muscle cells. *J Mol Cell Cardiol* **2003**, *35*, 1135-1143.
238. Yagi, M.; Uchiumi, T.; Takazaki, S.; Okuno, B.; Nomura, M.; Yoshida, S.; Kanki, T.; Kang, D. p32/gC1qR is indispensable for fetal development and mitochondrial translation: importance of its RNA-binding ability. *Nucleic Acids Res* **2012**, *40*, 9717-9737, doi:10.1093/nar/gks774.
239. Bernhard, O.K.; Cunningham, A.L.; Sheil, M.M. Analysis of proteins copurifying with the CD4/lck complex using one-dimensional polyacrylamide gel electrophoresis and mass spectrometry: comparison with affinity-tag based protein detection and evaluation of different solubilization methods. *J Am Soc Mass Spectrom* **2004**, *15*, 558-567, doi:10.1016/j.jasms.2003.12.006.
240. Catimel, B.; Rothacker, J.; Catimel, J.; Faux, M.; Ross, J.; Connolly, L.; Clippingdale, A.; Burgess, A.W.; Nice, E. Biosensor-based micro-affinity purification for the proteomic analysis of protein complexes. *J Proteome Res* **2005**, *4*, 1646-1656, doi:10.1021/pr050132x.
241. Oshikawa, K.; Matsumoto, M.; Yada, M.; Kamura, T.; Hatakeyama, S.; Nakayama, K.I. Preferential interaction of TIP120A with Cul1 that is not modified by NEDD8 and not associated with Skp1. *Biochem Biophys Res Commun* **2003**, *303*, 1209-1216.
242. Sircoulomb, F.; Nicolas, N.; Ferrari, A.; Finetti, P.; Bekhouche, I.; Rousselet, E.; Lonigro, A.; Adelaide, J.; Baudelet, E.; Esteyries, S.; et al. ZNF703 gene amplification at 8p12 specifies luminal B breast cancer. *EMBO Mol Med* **2011**, *3*, 153-166, doi:10.1002/emmm.201100121.
243. Cloutier, P.; Lavallee-Adam, M.; Faubert, D.; Blanchette, M.; Coulombe, B. A newly uncovered group of distantly related lysine methyltransferases preferentially interact with molecular chaperones to regulate their activity. *PLoS Genet* **2013**, *9*, e1003210, doi:10.1371/journal.pgen.1003210.
244. Okada, M.; Ohtake, F.; Nishikawa, H.; Wu, W.; Saeki, Y.; Takana, K.; Ohta, T. Liganded ERalpha Stimulates the E3 Ubiquitin Ligase Activity of UBE3C to Facilitate Cell Proliferation. *Mol Endocrinol* **2015**, *29*, 1646-1657, doi:10.1210/me.2015-1125.
245. Lokireddy, S.; Wijesoma, I.W.; Sze, S.K.; McFarlane, C.; Kambadur, R.; Sharma, M. Identification of atrogin-1-targeted proteins during the myostatin-induced skeletal muscle wasting. *Am J Physiol Cell Physiol* **2012**, *303*, C512-529, doi:10.1152/ajpcell.00402.2011.

246. Zhang, S.; Li, J.; Wang, C.C. GroEL-assisted dehydrogenase folding mediated by coenzyme is ATP-independent. *Biochem Biophys Res Commun* **2001**, *285*, 277-282, doi:10.1006/bbrc.2001.5182.
247. Roy, R.; Durie, D.; Li, H.; Liu, B.Q.; Skehel, J.M.; Mauri, F.; Cuorvo, L.V.; Barbareschi, M.; Guo, L.; Holcik, M.; et al. hnRNPA1 couples nuclear export and translation of specific mRNAs downstream of FGF-2/S6K2 signalling. *Nucleic Acids Res* **2014**, *42*, 12483-12497, doi:10.1093/nar/gku953.
248. Takaki, E.; Fujimoto, M.; Sugahara, K.; Nakahari, T.; Yonemura, S.; Tanaka, Y.; Hayashida, N.; Inouye, S.; Takemoto, T.; Yamashita, H.; et al. Maintenance of olfactory neurogenesis requires HSF1, a major heat shock transcription factor in mice. *J Biol Chem* **2006**, *281*, 4931-4937, doi:10.1074/jbc.M506911200.
249. Chen, S.; Prapapanich, V.; Rimerman, R.A.; Honore, B.; Smith, D.F. Interactions of p60, a mediator of progesterone receptor assembly, with heat shock proteins hsp90 and hsp70. *Mol Endocrinol* **1996**, *10*, 682-693, doi:10.1210/mend.10.6.8776728.
250. Vaseva, A.V.; Marchenko, N.D.; Ji, K.; Tsirka, S.E.; Holzmann, S.; Moll, U.M. p53 opens the mitochondrial permeability transition pore to trigger necrosis. *Cell* **2012**, *149*, 1536-1548, doi:10.1016/j.cell.2012.05.014.
251. Suzuki, T.; Kurita, H.; Ichinose, H. GTP cyclohydrolase I utilizes metal-free GTP as its substrate. *Eur J Biochem* **2004**, *271*, 349-355.
252. Kaltenbach, L.S.; Romero, E.; Becklin, R.R.; Chettier, R.; Bell, R.; Phansalkar, A.; Strand, A.; Torcassi, C.; Savage, J.; Hurlburt, A.; et al. Huntingtin interacting proteins are genetic modifiers of neurodegeneration. *PLoS Genet* **2007**, *3*, e82, doi:10.1371/journal.pgen.0030082.
253. Barazi, H.O.; Zhou, L.; Templeton, N.S.; Krutzsch, H.C.; Roberts, D.D. Identification of heat shock protein 60 as a molecular mediator of alpha 3 beta 1 integrin activation. *Cancer Res* **2002**, *62*, 1541-1548.
254. Nikiforov, M.A.; Chandriani, S.; Park, J.; Kotenko, I.; Matheos, D.; Johnsson, A.; McMahon, S.B.; Cole, M.D. TRRAP-dependent and TRRAP-independent transcriptional activation by Myc family oncoproteins. *Mol Cell Biol* **2002**, *22*, 5054-5063, doi:10.1128/mcb.22.14.5054-5063.2002.
255. Sang, L.; Miller, J.J.; Corbit, K.C.; Giles, R.H.; Brauer, M.J.; Otto, E.A.; Baye, L.M.; Wen, X.; Scales, S.J.; Kwong, M.; et al. Mapping the NPHP-JBTS-MKS protein network reveals ciliopathy disease genes and pathways. *Cell* **2011**, *145*, 513-528, doi:10.1016/j.cell.2011.04.019.
256. Crockett, D.K.; Lin, Z.; Elenitoba-Johnson, K.S.; Lim, M.S. Identification of NPM-ALK interacting proteins by tandem mass spectrometry. *Oncogene* **2004**, *23*, 2617-2629, doi:10.1038/sj.onc.1207398.
257. Hittelman, A.B.; Burakov, D.; Iniguez-Lluhi, J.A.; Freedman, L.P.; Garabedian, M.J. Differential regulation of glucocorticoid receptor transcriptional activation via AF-1-associated proteins. *EMBO J* **1999**, *18*, 5380-5388, doi:10.1093/emboj/18.19.5380.
258. Wu, G.R.; Mu, T.C.; Gao, Z.X.; Wang, J.; Sy, M.S.; Li, C.Y. Prion protein is required for tumor necrosis factor alpha (TNFalpha)-triggered nuclear factor kappaB (NF-kappaB) signaling and cytokine production. *J Biol Chem* **2017**, *292*, 18747-18759, doi:10.1074/jbc.M117.787283.
259. Fedorova, O.A.; Moiseeva, T.N.; Nikiforov, A.A.; Tsimokha, A.S.; Livinskaya, V.A.; Hodson, M.; Bottrill, A.; Evteeva, I.N.; Ermolayeva, J.B.; Kuznetzova, I.M.; et al. Proteomic analysis of the 20S proteasome (PSMA3)-interacting proteins reveals a functional link between the proteasome and mRNA metabolism. *Biochem Biophys Res Commun* **2011**, *416*, 258-265, doi:10.1016/j.bbrc.2011.10.126.
260. Verrastro, I.; Tveen-Jensen, K.; Woscholski, R.; Spickett, C.M.; Pitt, A.R. Reversible oxidation of phosphatase and tensin homolog (PTEN) alters its interactions with signaling and regulatory proteins. *Free Radic Biol Med* **2016**, *90*, 24-34, doi:10.1016/j.freeradbiomed.2015.11.004.
261. O'Neill, E.; Rushworth, L.; Baccarini, M.; Kolch, W. Role of the kinase MST2 in suppression of apoptosis by the proto-oncogene product Raf-1. *Science* **2004**, *306*, 2267-2270, doi:10.1126/science.1103233.

262. Miyamoto-Sato, E.; Fujimori, S.; Ishizaka, M.; Hirai, N.; Masuoka, K.; Saito, R.; Ozawa, Y.; Hino, K.; Washio, T.; Tomita, M.; et al. A comprehensive resource of interacting protein regions for refining human transcription factor networks. *PLoS One* **2010**, *5*, e9289, doi:10.1371/journal.pone.0009289.
263. Xie, Y.; Avello, M.; Schirle, M.; McWhinnie, E.; Feng, Y.; Bric-Furlong, E.; Wilson, C.; Nathans, R.; Zhang, J.; Kirschner, M.W.; et al. Deubiquitinase FAM/USP9X interacts with the E3 ubiquitin ligase SMURF1 protein and protects it from ligase activity-dependent self-degradation. *J Biol Chem* **2013**, *288*, 2976-2985, doi:10.1074/jbc.M112.430066.
264. Jin, J.; Li, G.J.; Davis, J.; Zhu, D.; Wang, Y.; Pan, C.; Zhang, J. Identification of novel proteins associated with both alpha-synuclein and DJ-1. *Mol Cell Proteomics* **2007**, *6*, 845-859, doi:10.1074/mcp.M600182-MCP200.
265. Kumar, P.; Ambasta, R.K.; Veereshwarayya, V.; Rosen, K.M.; Kosik, K.S.; Band, H.; Mestril, R.; Patterson, C.; Querfurth, H.W. CHIP and HSPs interact with beta-APP in a proteasome-dependent manner and influence Abeta metabolism. *Hum Mol Genet* **2007**, *16*, 848-864, doi:10.1093/hmg/ddm030.
266. Rubel, C.E.; Schisler, J.C.; Hamlett, E.D.; DeKroon, R.M.; Gautel, M.; Alzate, O.; Patterson, C. Diggin' on u(biquitin): a novel method for the identification of physiological E3 ubiquitin ligase substrates. *Cell Biochem Biophys* **2013**, *67*, 127-138, doi:10.1007/s12013-013-9624-6.
267. Whisenant, T.C.; Peralta, E.R.; Aarreberg, L.D.; Gao, N.J.; Head, S.R.; Ordoukhanian, P.; Williamson, J.R.; Salomon, D.R. The Activation-Induced Assembly of an RNA/Protein Interactome Centered on the Splicing Factor U2AF2 Regulates Gene Expression in Human CD4 T Cells. *PLoS One* **2015**, *10*, e0144409, doi:10.1371/journal.pone.0144409.
268. Galban, S.; Fan, J.; Martindale, J.L.; Cheadle, C.; Hoffman, B.; Woods, M.P.; Temeles, G.; Brieger, J.; Decker, J.; Gorospe, M. von Hippel-Lindau protein-mediated repression of tumor necrosis factor alpha translation revealed through use of cDNA arrays. *Mol Cell Biol* **2003**, *23*, 2316-2328.
269. Jounai, N.; Takeshita, F.; Kobiyama, K.; Sawano, A.; Miyawaki, A.; Xin, K.Q.; Ishii, K.J.; Kawai, T.; Akira, S.; Suzuki, K.; et al. The Atg5 Atg12 conjugate associates with innate antiviral immune responses. *Proc Natl Acad Sci U S A* **2007**, *104*, 14050-14055, doi:10.1073/pnas.0704014104.
270. Lu, N.Z.; Collins, J.B.; Grissom, S.F.; Cidlowski, J.A. Selective regulation of bone cell apoptosis by translational isoforms of the glucocorticoid receptor. *Mol Cell Biol* **2007**, *27*, 7143-7160, doi:10.1128/MCB.00253-07.
271. Hattan, D.; Nesti, E.; Cachero, T.G.; Morielli, A.D. Tyrosine phosphorylation of Kv1.2 modulates its interaction with the actin-binding protein cortactin. *J Biol Chem* **2002**, *277*, 38596-38606, doi:10.1074/jbc.M205005200.
272. Kim, E.; Niethammer, M.; Rothschild, A.; Jan, Y.N.; Sheng, M. Clustering of Shaker-type K+ channels by interaction with a family of membrane-associated guanylate kinases. *Nature* **1995**, *378*, 85-88, doi:10.1038/378085a0.
273. Nitabach, M.N.; Llamas, D.A.; Araneda, R.C.; Intile, J.L.; Thompson, I.J.; Zhou, Y.I.; Holmes, T.C. A mechanism for combinatorial regulation of electrical activity: Potassium channel subunits capable of functioning as Src homology 3-dependent adaptors. *Proc Natl Acad Sci U S A* **2001**, *98*, 705-710, doi:10.1073/pnas.031446198.
274. Chen, N.; Onisko, B.; Napoli, J.L. The nuclear transcription factor RARalpha associates with neuronal RNA granules and suppresses translation. *J Biol Chem* **2008**, *283*, 20841-20847, doi:10.1074/jbc.M802314200.
275. Nie, D.Y.; Zhou, Z.H.; Ang, B.T.; Teng, F.Y.; Xu, G.; Xiang, T.; Wang, C.Y.; Zeng, L.; Takeda, Y.; Xu, T.L.; et al. Nogo-A at CNS paranodes is a ligand of Caspr: possible regulation of K(+) channel localization. *EMBO J* **2003**, *22*, 5666-5678, doi:10.1093/emboj/cdg570.
276. Shao, S.; Yang, Y.; Yuan, G.; Zhang, M.; Yu, X. Signaling molecules involved in lipid-induced pancreatic beta-cell dysfunction. *DNA Cell Biol* **2013**, *32*, 41-49, doi:10.1089/dna.2012.1874.

277. Marechal, A.; Li, J.M.; Ji, X.Y.; Wu, C.S.; Yazinski, S.A.; Nguyen, H.D.; Liu, S.; Jimenez, A.E.; Jin, J.; Zou, L. PRP19 transforms into a sensor of RPA-ssDNA after DNA damage and drives ATR activation via a ubiquitin-mediated circuitry. *Mol Cell* **2014**, *53*, 235-246, doi:10.1016/j.molcel.2013.11.002.
278. Ylikallio, E.; Kim, D.; Isohanni, P.; Auranen, M.; Kim, E.; Lonnqvist, T.; Tyynismaa, H. Dominant transmission of de novo KIF1A motor domain variant underlying pure spastic paraplegia. *Eur J Hum Genet* **2015**, *23*, 1427-1430, doi:10.1038/ejhg.2014.297.
279. Kamal, A.; Stokin, G.B.; Yang, Z.; Xia, C.H.; Goldstein, L.S. Axonal transport of amyloid precursor protein is mediated by direct binding to the kinesin light chain subunit of kinesin-I. *Neuron* **2000**, *28*, 449-459.
280. Corominas, R.; Yang, X.; Lin, G.N.; Kang, S.; Shen, Y.; Ghamsari, L.; Broly, M.; Rodriguez, M.; Tam, S.; Trigg, S.A.; et al. Protein interaction network of alternatively spliced isoforms from brain links genetic risk factors for autism. *Nat Commun* **2014**, *5*, 3650, doi:10.1038/ncomms4650.
281. Arbuckle, M.I.; Komiyama, N.H.; Delaney, A.; Coba, M.; Garry, E.M.; Rosie, R.; Allchorne, A.J.; Forsyth, L.H.; Bence, M.; Carlisle, H.J.; et al. The SH3 domain of postsynaptic density 95 mediates inflammatory pain through phosphatidylinositol-3-kinase recruitment. *EMBO Rep* **2010**, *11*, 473-478, doi:10.1038/embor.2010.63.
282. Yao, Z.; Darowski, K.; St-Denis, N.; Wong, V.; Offensperger, F.; Villedieu, A.; Amin, S.; Malty, R.; Aoki, H.; Guo, H.; et al. A Global Analysis of the Receptor Tyrosine Kinase-Protein Phosphatase Interactome. *Mol Cell* **2017**, *65*, 347-360, doi:10.1016/j.molcel.2016.12.004.
283. Nassa, G.; Giurato, G.; Salvati, A.; Gigantino, V.; Pecoraro, G.; Lamberti, J.; Rizzo, F.; Nyman, T.A.; Tarallo, R.; Weisz, A. The RNA-mediated estrogen receptor alpha interactome of hormone-dependent human breast cancer cell nuclei. *Sci Data* **2019**, *6*, 173, doi:10.1038/s41597-019-0179-2.
284. Tabet, R.; Moutin, E.; Becker, J.A.; Heintz, D.; Fouillen, L.; Flatter, E.; Krezel, W.; Alunni, V.; Koebel, P.; Dembele, D.; et al. Fragile X Mental Retardation Protein (FMRP) controls diacylglycerol kinase activity in neurons. *Proc Natl Acad Sci U S A* **2016**, *113*, E3619-3628, doi:10.1073/pnas.1522631113.
285. Bai, Y.; Markham, K.; Chen, F.; Weerasekera, R.; Watts, J.; Horne, P.; Wakutani, Y.; Bagshaw, R.; Mathews, P.M.; Fraser, P.E.; et al. The in vivo brain interactome of the amyloid precursor protein. *Mol Cell Proteomics* **2008**, *7*, 15-34, doi:10.1074/mcp.M700077-MCP200.
286. Ferguson, D.; Shao, N.; Heller, E.; Feng, J.; Neve, R.; Kim, H.D.; Call, T.; Magazu, S.; Shen, L.; Nestler, E.J. SIRT1-FOXO3a regulate cocaine actions in the nucleus accumbens. *J Neurosci* **2015**, *35*, 3100-3111, doi:10.1523/JNEUROSCI.4012-14.2015.
287. Zhang, X.; Smits, A.H.; van Tilburg, G.B.; Jansen, P.W.; Makowski, M.M.; Ovaa, H.; Vermeulen, M. An Interaction Landscape of Ubiquitin Signaling. *Mol Cell* **2017**, *65*, 941-955 e948, doi:10.1016/j.molcel.2017.01.004.
288. Hutchins, J.R.; Toyoda, Y.; Hegemann, B.; Poser, I.; Heriche, J.K.; Sykora, M.M.; Augsburg, M.; Hudecz, O.; Buschhorn, B.A.; Bulkescher, J.; et al. Systematic analysis of human protein complexes identifies chromosome segregation proteins. *Science* **2010**, *328*, 593-599, doi:10.1126/science.1181348.
289. Splinter, D.; Tanenbaum, M.E.; Lindqvist, A.; Jaarsma, D.; Flotho, A.; Yu, K.L.; Grigoriev, I.; Engelsma, D.; Haasdijk, E.D.; Keijzer, N.; et al. Bicaudal D2, dynein, and kinesin-1 associate with nuclear pore complexes and regulate centrosome and nuclear positioning during mitotic entry. *PLoS Biol* **2010**, *8*, e1000350, doi:10.1371/journal.pbio.1000350.
290. Kopp, P.; Lammers, R.; Aepfelbacher, M.; Woehlke, G.; Rudel, T.; Machuy, N.; Steffen, W.; Linder, S. The kinesin KIF1C and microtubule plus ends regulate podosome dynamics in macrophages. *Mol Biol Cell* **2006**, *17*, 2811-2823, doi:10.1091/mbc.e05-11-1010.

291. Dorner, C.; Ullrich, A.; Haring, H.U.; Lammers, R. The kinesin-like motor protein KIF1C occurs in intact cells as a dimer and associates with proteins of the 14-3-3 family. *J Biol Chem* **1999**, *274*, 33654-33660.
292. Boutchueng-Djidjou, M.; Collard-Simard, G.; Fortier, S.; Hebert, S.S.; Kelly, I.; Landry, C.R.; Faure, R.L. The last enzyme of the de novo purine synthesis pathway 5-aminoimidazole-4-carboxamide ribonucleotide formyltransferase/IMP cyclohydrolase (ATIC) plays a central role in insulin signaling and the Golgi/endosomes protein network. *Mol Cell Proteomics* **2015**, *14*, 1079-1092, doi:10.1074/mcp.M114.047159.
293. Colin, E.; Zala, D.; Liot, G.; Rangone, H.; Borrell-Pages, M.; Li, X.J.; Saudou, F.; Humbert, S. Huntingtin phosphorylation acts as a molecular switch for anterograde/retrograde transport in neurons. *EMBO J* **2008**, *27*, 2124-2134, doi:10.1038/emboj.2008.133.
294. Li, L.; Lurmann, F.; Habre, R.; Urman, R.; Rappaport, E.; Ritz, B.; Chen, J.C.; Gilliland, F.D.; Wu, J. Constrained Mixed-Effect Models with Ensemble Learning for Prediction of Nitrogen Oxides Concentrations at High Spatiotemporal Resolution. *Environ Sci Technol* **2017**, *51*, 9920-9929, doi:10.1021/acs.est.7b01864.
295. Shinoda, T.; Taya, S.; Tsuboi, D.; Hikita, T.; Matsuzawa, R.; Kuroda, S.; Iwamatsu, A.; Kaibuchi, K. DISC1 regulates neurotrophin-induced axon elongation via interaction with Grb2. *J Neurosci* **2007**, *27*, 4-14, doi:10.1523/JNEUROSCI.3825-06.2007.
296. Mandal, M.; Wei, J.; Zhong, P.; Cheng, J.; Duffney, L.J.; Liu, W.; Yuen, E.Y.; Twelvetrees, A.E.; Li, S.; Li, X.J.; et al. Impaired alpha-amino-3-hydroxy-5-methyl-4-isoxazolepropionic acid (AMPA) receptor trafficking and function by mutant huntingtin. *J Biol Chem* **2011**, *286*, 33719-33728, doi:10.1074/jbc.M111.236521.
297. Engelender, S.; Sharp, A.H.; Colomer, V.; Tokito, M.K.; Lanahan, A.; Worley, P.; Holzbaur, E.L.; Ross, C.A. Huntingtin-associated protein 1 (HAP1) interacts with the p150Glued subunit of dynactin. *Hum Mol Genet* **1997**, *6*, 2205-2212, doi:10.1093/hmg/6.13.2205.
298. Kanai, Y.; Okada, Y.; Tanaka, Y.; Harada, A.; Terada, S.; Hirokawa, N. KIF5C, a novel neuronal kinesin enriched in motor neurons. *J Neurosci* **2000**, *20*, 6374-6384.
299. Rahman, A.; Kamal, A.; Roberts, E.A.; Goldstein, L.S. Defective kinesin heavy chain behavior in mouse kinesin light chain mutants. *J Cell Biol* **1999**, *146*, 1277-1288.
300. Brendel, C.; Rehbein, M.; Kreienkamp, H.J.; Buck, F.; Richter, D.; Kindler, S. Characterization of Staufen 1 ribonucleoprotein complexes. *Biochem J* **2004**, *384*, 239-246, doi:10.1042/BJ20040812.
301. Amit, I.; Yakir, L.; Katz, M.; Zwang, Y.; Marmor, M.D.; Citri, A.; Shtiegman, K.; Alroy, I.; Tuvia, S.; Reiss, Y.; et al. Tal, a Tsg101-specific E3 ubiquitin ligase, regulates receptor endocytosis and retrovirus budding. *Genes Dev* **2004**, *18*, 1737-1752, doi:10.1101/gad.294904.
302. Espanel, X.; Sudol, M. Yes-associated protein and p53-binding protein-2 interact through their WW and SH3 domains. *J Biol Chem* **2001**, *276*, 14514-14523, doi:10.1074/jbc.M008568200.
303. Ballif, B.A.; Cao, Z.; Schwartz, D.; Carraway, K.L., 3rd; Gygi, S.P. Identification of 14-3-3epsilon substrates from embryonic murine brain. *J Proteome Res* **2006**, *5*, 2372-2379, doi:10.1021/pr060206k.
304. Matsuzaki, F.; Shirane, M.; Matsumoto, M.; Nakayama, K.I. Protrudin serves as an adaptor molecule that connects KIF5 and its cargoes in vesicular transport during process formation. *Mol Biol Cell* **2011**, *22*, 4602-4620, doi:10.1091/mbc.E11-01-0068.
305. Vinayagam, A.; Stelzl, U.; Foulle, R.; Plassmann, S.; Zenkner, M.; Timm, J.; Assmus, H.E.; Andrade-Navarro, M.A.; Wanker, E.E. A directed protein interaction network for investigating intracellular signal transduction. *Sci Signal* **2011**, *4*, rs8, doi:10.1126/scisignal.2001699.
306. Guo, Z.; Neilson, L.J.; Zhong, H.; Murray, P.S.; Zanivan, S.; Zaidel-Bar, R. E-cadherin interactome complexity and robustness resolved by quantitative proteomics. *Sci Signal* **2014**, *7*, rs7, doi:10.1126/scisignal.2005473.

307. Du, J.; Wei, Y.; Liu, L.; Wang, Y.; Khairova, R.; Blumenthal, R.; Tragon, T.; Hunsberger, J.G.; Machado-Vieira, R.; Drevets, W.; et al. A kinesin signaling complex mediates the ability of GSK-3beta to affect mood-associated behaviors. *Proc Natl Acad Sci U S A* **2010**, *107*, 11573-11578, doi:10.1073/pnas.0913138107.
308. Rong, J.; Li, S.; Sheng, G.; Wu, M.; Coblitz, B.; Li, M.; Fu, H.; Li, X.J. 14-3-3 protein interacts with Huntingtin-associated protein 1 and regulates its trafficking. *J Biol Chem* **2007**, *282*, 4748-4756, doi:10.1074/jbc.M609057200.
309. Rahman, A.; Friedman, D.S.; Goldstein, L.S. Two kinesin light chain genes in mice. Identification and characterization of the encoded proteins. *J Biol Chem* **1998**, *273*, 15395-15403.
310. Gloeckner, C.J.; Boldt, K.; Schumacher, A.; Roepman, R.; Ueffing, M. A novel tandem affinity purification strategy for the efficient isolation and characterisation of native protein complexes. *Proteomics* **2007**, *7*, 4228-4234, doi:10.1002/pmic.200700038.
311. Marx, M.; Diestel, S.; Bozon, M.; Keglowich, L.; Drouot, N.; Bouche, E.; Frebourg, T.; Minz, M.; Saugier-Veber, P.; Castellani, V.; et al. Pathomechanistic characterization of two exonic L1CAM variants located in trans in an obligate carrier of X-linked hydrocephalus. *Neurogenetics* **2012**, *13*, 49-59, doi:10.1007/s10048-011-0307-4.
312. Donier, E.; Gomez-Sanchez, J.A.; Grijota-Martinez, C.; Lakoma, J.; Baars, S.; Garcia-Alonso, L.; Cabedo, H. L1CAM binds ErbB receptors through Ig-like domains coupling cell adhesion and neuregulin signalling. *PLoS One* **2012**, *7*, e40674, doi:10.1371/journal.pone.0040674.
313. Dickson, T.C.; Mintz, C.D.; Benson, D.L.; Salton, S.R. Functional binding interaction identified between the axonal CAM L1 and members of the ERM family. *J Cell Biol* **2002**, *157*, 1105-1112, doi:10.1083/jcb.200111076.
314. Kulahin, N.; Li, S.; Hinsby, A.; Kiselyov, V.; Berezin, V.; Bock, E. Fibronectin type III (FN3) modules of the neuronal cell adhesion molecule L1 interact directly with the fibroblast growth factor (FGF) receptor. *Mol Cell Neurosci* **2008**, *37*, 528-536, doi:10.1016/j.mcn.2007.12.001.
315. Previtali, S.C.; Feltri, M.L.; Archelos, J.J.; Quattrini, A.; Wrabetz, L.; Hartung, H. Role of integrins in the peripheral nervous system. *Prog Neurobiol* **2001**, *64*, 35-49.
316. Schaefer, A.W.; Kamiguchi, H.; Wong, E.V.; Beach, C.M.; Landreth, G.; Lemmon, V. Activation of the MAPK signal cascade by the neural cell adhesion molecule L1 requires L1 internalization. *J Biol Chem* **1999**, *274*, 37965-37973.
317. Masumi, A.; Yamakawa, Y.; Fukazawa, H.; Ozato, K.; Komuro, K. Interferon regulatory factor-2 regulates cell growth through its acetylation. *J Biol Chem* **2003**, *278*, 25401-25407, doi:10.1074/jbc.M213037200.
318. Schmitt-Ulms, G.; Hansen, K.; Liu, J.; Cowdrey, C.; Yang, J.; DeArmond, S.J.; Cohen, F.E.; Prusiner, S.B.; Baldwin, M.A. Time-controlled transcardiac perfusion cross-linking for the study of protein interactions in complex tissues. *Nat Biotechnol* **2004**, *22*, 724-731, doi:10.1038/nbt969.
319. Jeon, A.H.; Bohm, C.; Chen, F.; Huo, H.; Ruan, X.; Ren, C.H.; Ho, K.; Qamar, S.; Mathews, P.M.; Fraser, P.E.; et al. Interactome analyses of mature gamma-secretase complexes reveal distinct molecular environments of presenilin (PS) paralogs and preferential binding of signal peptide peptidase to PS2. *J Biol Chem* **2013**, *288*, 15352-15366, doi:10.1074/jbc.M112.441840.
320. Cheng, L.; Lemmon, S.; Lemmon, V. RanBPM is an L1-interacting protein that regulates L1-mediated mitogen-activated protein kinase activation. *J Neurochem* **2005**, *94*, 1102-1110, doi:10.1111/j.1471-4159.2005.03254.x.
321. Wallace, A.S.; Schmidt, C.; Schachner, M.; Wegner, M.; Anderson, R.B. L1cam acts as a modifier gene during enteric nervous system development. *Neurobiol Dis* **2010**, *40*, 622-633, doi:10.1016/j.nbd.2010.08.006.

322. Tchernev, V.T.; Mansfield, T.A.; Giot, L.; Kumar, A.M.; Nandabalan, K.; Li, Y.; Mishra, V.S.; Detter, J.C.; Rothberg, J.M.; Wallace, M.R.; et al. The Chediak-Higashi protein interacts with SNARE complex and signal transduction proteins. *Mol Med* **2002**, *8*, 56-64.
323. Polman, J.A.; de Kloet, E.R.; Datson, N.A. Two populations of glucocorticoid receptor-binding sites in the male rat hippocampal genome. *Endocrinology* **2013**, *154*, 1832-1844, doi:10.1210/en.2012-2187.
324. Fahrig, T.; Landa, C.; Pesheva, P.; Kuhn, K.; Schachner, M. Characterization of binding properties of the myelin-associated glycoprotein to extracellular matrix constituents. *EMBO J* **1987**, *6*, 2875-2883.
325. Strenge, K.; Brossmer, R.; Ihrig, P.; Schauer, R.; Kelm, S. Fibronectin is a binding partner for the myelin-associated glycoprotein (siglec-4a). *FEBS Lett* **2001**, *499*, 262-267.
326. Thomas, S.M.; Brugge, J.S. Cellular functions regulated by Src family kinases. *Annu Rev Cell Dev Biol* **1997**, *13*, 513-609, doi:10.1146/annurev.cellbio.13.1.513.
327. Tsuchida, T.; Parker, K.C.; Turner, R.V.; McFarland, H.F.; Coligan, J.E.; Biddison, W.E. Autoreactive CD8+ T-cell responses to human myelin protein-derived peptides. *Proc Natl Acad Sci U S A* **1994**, *91*, 10859-10863.
328. Zhao, L.; Mandler, M.D.; Yi, H.; Feng, Y. Quaking I controls a unique cytoplasmic pathway that regulates alternative splicing of myelin-associated glycoprotein. *Proc Natl Acad Sci U S A* **2010**, *107*, 19061-19066, doi:10.1073/pnas.1007487107.
329. Stiles, T.L.; Dickendesher, T.L.; Gaultier, A.; Fernandez-Castaneda, A.; Mantuano, E.; Giger, R.J.; Gonias, S.L. LDL receptor-related protein-1 is a sialic-acid-independent receptor for myelin-associated glycoprotein that functions in neurite outgrowth inhibition by MAG and CNS myelin. *J Cell Sci* **2013**, *126*, 209-220, doi:10.1242/jcs.113191.
330. Jensen, N.A.; West, M.J.; Celis, J.E. Oligodendrocyte programmed cell death and central myelination deficiency induced in transgenic mice by synergism between c-Myc and Oct-6. *J Biol Chem* **1999**, *274*, 29921-29926, doi:10.1074/jbc.274.42.29921.
331. LeBlanc, S.E.; Ward, R.M.; Svaren, J. Neuropathy-associated Egr2 mutants disrupt cooperative activation of myelin protein zero by Egr2 and Sox10. *Mol Cell Biol* **2007**, *27*, 3521-3529, doi:10.1128/MCB.01689-06.
332. Kim, J.Y.; Kang, Y.S.; Lee, J.W.; Kim, H.J.; Ahn, Y.H.; Park, H.; Ko, Y.G.; Kim, S. p38 is essential for the assembly and stability of macromolecular tRNA synthetase complex: implications for its physiological significance. *Proc Natl Acad Sci U S A* **2002**, *99*, 7912-7916, doi:10.1073/pnas.122110199.
333. Mak, A.B.; Ni, Z.; Hewel, J.A.; Chen, G.I.; Zhong, G.; Karamboulas, K.; Blakely, K.; Smiley, S.; Marcon, E.; Roudeva, D.; et al. A lentiviral functional proteomics approach identifies chromatin remodeling complexes important for the induction of pluripotency. *Mol Cell Proteomics* **2010**, *9*, 811-823, doi:10.1074/mcp.M000002-MCP201.
334. Foerster, S.; Kacprowski, T.; Dhople, V.M.; Hammer, E.; Herzog, S.; Saafan, H.; Bien-Moller, S.; Albrecht, M.; Volker, U.; Ritter, C.A. Characterization of the EGFR interactome reveals associated protein complex networks and intracellular receptor dynamics. *Proteomics* **2013**, *13*, 3131-3144, doi:10.1002/pmic.201300154.
335. Nalvarte, I.; Schwend, T.; Gustafsson, J.A. Proteomics analysis of the estrogen receptor alpha receptosome. *Mol Cell Proteomics* **2010**, *9*, 1411-1422, doi:10.1074/mcp.M900457-MCP200.
336. Kang, J.; Kim, T.; Ko, Y.G.; Rho, S.B.; Park, S.G.; Kim, M.J.; Kwon, H.J.; Kim, S. Heat shock protein 90 mediates protein-protein interactions between human aminoacyl-tRNA synthetases. *J Biol Chem* **2000**, *275*, 31682-31688, doi:10.1074/jbc.M909965199.
337. Brooks, C.; Wei, Q.; Feng, L.; Dong, G.; Tao, Y.; Mei, L.; Xie, Z.J.; Dong, Z. Bak regulates mitochondrial morphology and pathology during apoptosis by interacting with mitofusins. *Proc Natl Acad Sci U S A* **2007**, *104*, 11649-11654, doi:10.1073/pnas.0703976104.

338. Lee, Y.J.; Wang, Q.; Rio, D.C. Coordinate regulation of alternative pre-mRNA splicing events by the human RNA chaperone proteins hnRNPA1 and DDX5. *Genes Dev* **2018**, *32*, 1060-1074, doi:10.1101/gad.316034.118.
339. Shirasaki, D.I.; Greiner, E.R.; Al-Ramahi, I.; Gray, M.; Boontheung, P.; Geschwind, D.H.; Botas, J.; Coppola, G.; Horvath, S.; Loo, J.A.; et al. Network organization of the huntingtin proteomic interactome in mammalian brain. *Neuron* **2012**, *75*, 41-57, doi:10.1016/j.neuron.2012.05.024.
340. Chen, K.H.; Dasgupta, A.; Ding, J.; Indig, F.E.; Ghosh, P.; Longo, D.L. Role of mitofusin 2 (Mfn2) in controlling cellular proliferation. *FASEB J* **2014**, *28*, 382-394, doi:10.1096/fj.13-230037.
341. Varaljai, R.; Islam, A.B.; Beshiri, M.L.; Rehman, J.; Lopez-Bigas, N.; Benevolenskaya, E.V. Increased mitochondrial function downstream from KDM5A histone demethylase rescues differentiation in pRB-deficient cells. *Genes Dev* **2015**, *29*, 1817-1834, doi:10.1101/gad.264036.115.
342. Mick, D.U.; Dennerlein, S.; Wiese, H.; Reinhold, R.; Pacheu-Grau, D.; Lorenzi, I.; Sasarman, F.; Weraarpachai, W.; Shoubridge, E.A.; Warscheid, B.; et al. MITRAC links mitochondrial protein translocation to respiratory-chain assembly and translational regulation. *Cell* **2012**, *151*, 1528-1541, doi:10.1016/j.cell.2012.11.053.
343. Gunther, M.; Laithier, M.; Brison, O. A set of proteins interacting with transcription factor Sp1 identified in a two-hybrid screening. *Mol Cell Biochem* **2000**, *210*, 131-142.
344. Shen, X.; Valencia, C.A.; Szostak, J.W.; Dong, B.; Liu, R. Scanning the human proteome for calmodulin-binding proteins. *Proc Natl Acad Sci U S A* **2005**, *102*, 5969-5974, doi:10.1073/pnas.0407928102.
345. Li, X.; Tran, K.M.; Aziz, K.E.; Sorokin, A.V.; Chen, J.; Wang, W. Defining the Protein-Protein Interaction Network of the Human Protein Tyrosine Phosphatase Family. *Mol Cell Proteomics* **2016**, *15*, 3030-3044, doi:10.1074/mcp.M116.060277.
346. Kong, B.; Wang, Q.; Fung, E.; Xue, K.; Tsang, B.K. p53 is required for cisplatin-induced processing of the mitochondrial fusion protein L-Opa1 that is mediated by the mitochondrial metallopeptidase Oma1 in gynecologic cancers. *J Biol Chem* **2014**, *289*, 27134-27145, doi:10.1074/jbc.M114.594812.
347. Floyd, B.J.; Wilkerson, E.M.; Veling, M.T.; Minogue, C.E.; Xia, C.; Beebe, E.T.; Wrobel, R.L.; Cho, H.; Kremer, L.S.; Alston, C.L.; et al. Mitochondrial Protein Interaction Mapping Identifies Regulators of Respiratory Chain Function. *Mol Cell* **2016**, *63*, 621-632, doi:10.1016/j.molcel.2016.06.033.
348. Ko, H.S.; Lee, Y.; Shin, J.H.; Karuppagounder, S.S.; Gadad, B.S.; Koleske, A.J.; Pletnikova, O.; Troncoso, J.C.; Dawson, V.L.; Dawson, T.M. Phosphorylation by the c-Abl protein tyrosine kinase inhibits parkin's ubiquitination and protective function. *Proc Natl Acad Sci U S A* **2010**, *107*, 16691-16696, doi:10.1073/pnas.1006083107.
349. Johnson, B.N.; Berger, A.K.; Cortese, G.P.; Lavoie, M.J. The ubiquitin E3 ligase parkin regulates the proapoptotic function of Bax. *Proc Natl Acad Sci U S A* **2012**, *109*, 6283-6288, doi:10.1073/pnas.1113248109.
350. Gong, Y.; Zack, T.I.; Morris, L.G.; Lin, K.; Hukkelhoven, E.; Raheja, R.; Tan, I.L.; Turcan, S.; Veeriah, S.; Meng, S.; et al. Pan-cancer genetic analysis identifies PARK2 as a master regulator of G1/S cyclins. *Nat Genet* **2014**, *46*, 588-594, doi:10.1038/ng.2981.
351. Hosp, F.; Vossfeldt, H.; Heinig, M.; Vasiljevic, D.; Arumughan, A.; Wyler, E.; Genetic; Environmental Risk for Alzheimer's Disease, G.C.; Landthaler, M.; Hubner, N.; et al. Quantitative interaction proteomics of neurodegenerative disease proteins. *Cell Rep* **2015**, *11*, 1134-1146, doi:10.1016/j.celrep.2015.04.030.
352. Avraham, E.; Rott, R.; Liani, E.; Szargel, R.; Engelender, S. Phosphorylation of Parkin by the cyclin-dependent kinase 5 at the linker region modulates its ubiquitin-ligase activity and aggregation. *J Biol Chem* **2007**, *282*, 12842-12850, doi:10.1074/jbc.M608243200.

353. Lin, D.C.; Xu, L.; Chen, Y.; Yan, H.; Hazawa, M.; Doan, N.; Said, J.W.; Ding, L.W.; Liu, L.Z.; Yang, H.; et al. Genomic and Functional Analysis of the E3 Ligase PARK2 in Glioma. *Cancer Res* **2015**, *75*, 1815-1827, doi:10.1158/0008-5472.CAN-14-1433.
354. Staropoli, J.F.; McDermott, C.; Martinat, C.; Schulman, B.; Demireva, E.; Abeliovich, A. Parkin is a component of an SCF-like ubiquitin ligase complex and protects postmitotic neurons from kainate excitotoxicity. *Neuron* **2003**, *37*, 735-749, doi:10.1016/s0896-6273(03)00084-9.
355. Fallon, L.; Belanger, C.M.; Corera, A.T.; Kontogiannea, M.; Regan-Klapisz, E.; Moreau, F.; Voortman, J.; Haber, M.; Rouleau, G.; Thorarinsdottir, T.; et al. A regulated interaction with the UIM protein Eps15 implicates parkin in EGF receptor trafficking and PI(3)K-Akt signalling. *Nat Cell Biol* **2006**, *8*, 834-842, doi:10.1038/ncb1441.
356. Jiang, Q.; Ren, Y.; Feng, J. Direct binding with histone deacetylase 6 mediates the reversible recruitment of parkin to the centrosome. *J Neurosci* **2008**, *28*, 12993-13002, doi:10.1523/JNEUROSCI.2860-08.2008.
357. Yang, H.; Zhou, X.; Liu, X.; Yang, L.; Chen, Q.; Zhao, D.; Zuo, J.; Liu, W. Mitochondrial dysfunction induced by knockdown of mortalin is rescued by Parkin. *Biochem Biophys Res Commun* **2011**, *410*, 114-120, doi:10.1016/j.bbrc.2011.05.116.
358. Sha, D.; Chin, L.S.; Li, L. Phosphorylation of parkin by Parkinson disease-linked kinase PINK1 activates parkin E3 ligase function and NF-kappaB signaling. *Hum Mol Genet* **2010**, *19*, 352-363, doi:10.1093/hmg/ddp501.
359. Palacino, J.J.; Sagi, D.; Goldberg, M.S.; Krauss, S.; Motz, C.; Wacker, M.; Klose, J.; Shen, J. Mitochondrial dysfunction and oxidative damage in parkin-deficient mice. *J Biol Chem* **2004**, *279*, 18614-18622, doi:10.1074/jbc.M401135200.
360. Khan, M.; Syed, G.H.; Kim, S.J.; Siddiqui, A. Hepatitis B Virus-Induced Parkin-Dependent Recruitment of Linear Ubiquitin Assembly Complex (LUBAC) to Mitochondria and Attenuation of Innate Immunity. *PLoS Pathog* **2016**, *12*, e1005693, doi:10.1371/journal.ppat.1005693.
361. Junn, E.; Lee, S.S.; Suhr, U.T.; Mouradian, M.M. Parkin accumulation in aggresomes due to proteasome impairment. *J Biol Chem* **2002**, *277*, 47870-47877, doi:10.1074/jbc.M203159200.
362. Imai, Y.; Soda, M.; Hatakeyama, S.; Akagi, T.; Hashikawa, T.; Nakayama, K.I.; Takahashi, R. CHIP is associated with Parkin, a gene responsible for familial Parkinson's disease, and enhances its ubiquitin ligase activity. *Mol Cell* **2002**, *10*, 55-67, doi:10.1016/s1097-2765(02)00583-x.
363. Haenig, C.; Atias, N.; Taylor, A.K.; Mazza, A.; Schaefer, M.H.; Russ, J.; Riechers, S.P.; Jain, S.; Coughlin, M.; Fontaine, J.F.; et al. Interactome Mapping Provides a Network of Neurodegenerative Disease Proteins and Uncovers Widespread Protein Aggregation in Affected Brains. *Cell Rep* **2020**, *32*, 108050, doi:10.1016/j.celrep.2020.108050.
364. Jung, Y.Y.; Son, D.J.; Lee, H.L.; Kim, D.H.; Song, M.J.; Ham, Y.W.; Kim, Y.; Han, S.B.; Park, M.H.; Hong, J.T. Loss of Parkin reduces inflammatory arthritis by inhibiting p53 degradation. *Redox Biol* **2017**, *12*, 666-673, doi:10.1016/j.redox.2017.04.007.
365. Xin, D.; Gu, H.; Liu, E.; Sun, Q. Parkin negatively regulates the antiviral signaling pathway by targeting TRAF3 for degradation. *J Biol Chem* **2018**, *293*, 11996-12010, doi:10.1074/jbc.RA117.001201.
366. Essandoh, K.; Wang, X.; Huang, W.; Deng, S.; Gardner, G.; Mu, X.; Li, Y.; Kranias, E.G.; Wang, Y.; Fan, G.C. Tumor susceptibility gene 101 ameliorates endotoxin-induced cardiac dysfunction by enhancing Parkin-mediated mitophagy. *J Biol Chem* **2019**, *294*, 18057-18068, doi:10.1074/jbc.RA119.008925.
367. McLelland, G.L.; Goiran, T.; Yi, W.; Dorval, G.; Chen, C.X.; Lauinger, N.D.; Krahn, A.I.; Valimehr, S.; Rakovic, A.; Rouiller, I.; et al. Mfn2 ubiquitination by PINK1/parkin gates the p97-dependent release of ER from mitochondria to drive mitophagy. *Elife* **2018**, *7*, doi:10.7554/eLife.32866.

368. Ben-Tekaya, H.; Kahn, R.A.; Hauri, H.P. ADP ribosylation factors 1 and 4 and group VIA phospholipase A(2) regulate morphology and intraorganellar traffic in the endoplasmic reticulum-Golgi intermediate compartment. *Mol Biol Cell* **2010**, *21*, 4130-4140, doi:10.1091/mbc.E10-01-0022.
369. Jenkins, C.M.; Wolf, M.J.; Mancuso, D.J.; Gross, R.W. Identification of the calmodulin-binding domain of recombinant calcium-independent phospholipase A2beta. implications for structure and function. *J Biol Chem* **2001**, *276*, 7129-7135, doi:10.1074/jbc.M010439200.
370. Lei, X.; Zhang, S.; Barbour, S.E.; Bohrer, A.; Ford, E.L.; Koizumi, A.; Papa, F.R.; Ramanadham, S. Spontaneous development of endoplasmic reticulum stress that can lead to diabetes mellitus is associated with higher calcium-independent phospholipase A2 expression: a role for regulation by SREBP-1. *J Biol Chem* **2010**, *285*, 6693-6705, doi:10.1074/jbc.M109.084293.
371. Arroyo, R.; Sune, G.; Zanzoni, A.; Duran-Frigola, M.; Alcalde, V.; Stracker, T.H.; Soler-Lopez, M.; Aloy, P. Systematic identification of molecular links between core and candidate genes in breast cancer. *J Mol Biol* **2015**, *427*, 1436-1450, doi:10.1016/j.jmb.2015.01.014.
372. Fernandez-Castaneda, A.; Arandjelovic, S.; Stiles, T.L.; Schlobach, R.K.; Mowen, K.A.; Gonias, S.L.; Gaultier, A. Identification of the low density lipoprotein (LDL) receptor-related protein-1 interactome in central nervous system myelin suggests a role in the clearance of necrotic cell debris. *J Biol Chem* **2013**, *288*, 4538-4548, doi:10.1074/jbc.M112.384693.
373. Jensen, N.A.; Pedersen, K.M.; Celis, J.E.; West, M.J. Failure of central nervous system myelination in MBP/c-myc transgenic mice: evidence for c-myc cytotoxicity. *Oncogene* **1998**, *16*, 2123-2129, doi:10.1038/sj.onc.1201739.
374. Dhaunchak, A.S.; Nave, K.A. A common mechanism of PLP/DM20 misfolding causes cysteine-mediated endoplasmic reticulum retention in oligodendrocytes and Pelizaeus-Merzbacher disease. *Proc Natl Acad Sci U S A* **2007**, *104*, 17813-17818, doi:10.1073/pnas.0704975104.
375. Lee, K.E.; Nam, S.; Cho, E.A.; Seong, I.; Limb, J.K.; Lee, S.; Kim, J. Identification of direct regulatory targets of the transcription factor Sox10 based on function and conservation. *BMC Genomics* **2008**, *9*, 408, doi:10.1186/1471-2164-9-408.
376. Konig, S.M.; Rissler, V.; Terkelsen, T.; Lambrughi, M.; Papaleo, E. Alterations of the interactome of Bcl-2 proteins in breast cancer at the transcriptional, mutational and structural level. *PLoS Comput Biol* **2019**, *15*, e1007485, doi:10.1371/journal.pcbi.1007485.
377. Raman, M.; Sergeev, M.; Garnaas, M.; Lydeard, J.R.; Huttlin, E.L.; Goessling, W.; Shah, J.V.; Harper, J.W. Systematic proteomics of the VCP-UBXD adaptor network identifies a role for UBXN10 in regulating ciliogenesis. *Nat Cell Biol* **2015**, *17*, 1356-1369, doi:10.1038/ncb3238.
378. Ravasi, T.; Suzuki, H.; Cannistraci, C.V.; Katayama, S.; Bajic, V.B.; Tan, K.; Akalin, A.; Schmeier, S.; Kanamori-Katayama, M.; Bertin, N.; et al. An atlas of combinatorial transcriptional regulation in mouse and man. *Cell* **2010**, *140*, 744-752, doi:10.1016/j.cell.2010.01.044.
379. Baron, Y.; Pedrioli, P.G.; Tyagi, K.; Johnson, C.; Wood, N.T.; Fountaine, D.; Wightman, M.; Alexandru, G. VAPB/ALS8 interacts with FFAT-like proteins including the p97 cofactor FAF1 and the ASNA1 ATPase. *BMC Biol* **2014**, *12*, 39, doi:10.1186/1741-7007-12-39.
380. Frye, M.; Gardner, C.; Li, E.R.; Arnold, I.; Watt, F.M. Evidence that Myc activation depletes the epidermal stem cell compartment by modulating adhesive interactions with the local microenvironment. *Development* **2003**, *130*, 2793-2808, doi:10.1242/dev.00462.
381. Montenegro, G.; Rebelo, A.P.; Connell, J.; Allison, R.; Babalini, C.; D'Aloia, M.; Montieri, P.; Schule, R.; Ishiura, H.; Price, J.; et al. Mutations in the ER-shaping protein reticulon 2 cause the axon-degenerative disorder hereditary spastic paraplegia type 12. *J Clin Invest* **2012**, *122*, 538-544, doi:10.1172/JCI60560.
382. Hatchi, E.; Skourti-Stathaki, K.; Ventz, S.; Pinello, L.; Yen, A.; Kamieniarz-Gdula, K.; Dimitrov, S.; Pathania, S.; McKinney, K.M.; Eaton, M.L.; et al. BRCA1 recruitment to transcriptional pause sites

is required for R-loop-driven DNA damage repair. *Mol Cell* **2015**, *57*, 636-647, doi:10.1016/j.molcel.2015.01.011.
383. Kogai, T.; Liu, Y.Y.; Richter, L.L.; Mody, K.; Kagechika, H.; Brent, G.A. Retinoic acid induces expression of the thyroid hormone transporter, monocarboxylate transporter 8 (Mct8). *J Biol Chem* **2010**, *285*, 27279-27288, doi:10.1074/jbc.M110.123158.
384. Milewska, M.; McRedmond, J.; Byrne, P.C. Identification of novel spartin-interactors shows spartin is a multifunctional protein. *J Neurochem* **2009**, *111*, 1022-1030, doi:10.1111/j.1471-4159.2009.06382.x.
385. Blandin, G.; Marchand, S.; Charton, K.; Daniele, N.; Gicquel, E.; Boucheteil, J.B.; Bentaib, A.; Barrault, L.; Stockholm, D.; Bartoli, M.; et al. A human skeletal muscle interactome centered on proteins involved in muscular dystrophies: LGMD interactome. *Skelet Muscle* **2013**, *3*, 3, doi:10.1186/2044-5040-3-3.
386. Suter, B.; Fontaine, J.F.; Yildirimman, R.; Rasko, T.; Schaefer, M.H.; Rasche, A.; Porras, P.; Vazquez-Alvarez, B.M.; Russ, J.; Rau, K.; et al. Development and application of a DNA microarray-based yeast two-hybrid system. *Nucleic Acids Res* **2013**, *41*, 1496-1507, doi:10.1093/nar/gks1329.
387. Renvoise, B.; Parker, R.L.; Yang, D.; Bakowska, J.C.; Hurley, J.H.; Blackstone, C. SPG20 protein spartin is recruited to midbodies by ESCRT-III protein Ist1 and participates in cytokinesis. *Mol Biol Cell* **2010**, *21*, 3293-3303, doi:10.1091/mbc.E09-10-0879.
388. Charlaftis, N.; Suddason, T.; Wu, X.; Anwar, S.; Karin, M.; Gallagher, E. The MEKK1 PHD ubiquitinates TAB1 to activate MAPKs in response to cytokines. *EMBO J* **2014**, *33*, 2581-2596, doi:10.15252/embj.201488351.
389. Li, J.; Bennett, K.; Stukalov, A.; Fang, B.; Zhang, G.; Yoshida, T.; Okamoto, I.; Kim, J.Y.; Song, L.; Bai, Y.; et al. Perturbation of the mutated EGFR interactome identifies vulnerabilities and resistance mechanisms. *Mol Syst Biol* **2013**, *9*, 705, doi:10.1038/msb.2013.61.
390. Miranda, C.; Roccato, E.; Raho, G.; Pagliardini, S.; Pierotti, M.A.; Greco, A. The TFG protein, involved in oncogenic rearrangements, interacts with TANK and NEMO, two proteins involved in the NF-kappaB pathway. *J Cell Physiol* **2006**, *208*, 154-160, doi:10.1002/jcp.20644.
391. Papin, J.; Subramaniam, S. Bioinformatics and cellular signaling. *Curr Opin Biotechnol* **2004**, *15*, 78-81, doi:10.1016/j.copbio.2004.01.003.
392. Cid, C.; Garcia-Bonilla, L.; Camafeita, E.; Burda, J.; Salinas, M.; Alcazar, A. Proteomic characterization of protein phosphatase 1 complexes in ischemia-reperfusion and ischemic tolerance. *Proteomics* **2007**, *7*, 3207-3218, doi:10.1002/pmic.200700214.
393. Gupta, T.; Hotwani, C.; Kannan, S.; Master, Z.; Rangarajan, V.; Murthy, V.; Budrukkar, A.; Ghosh-Laskar, S.; Agarwal, J.P. Prospective longitudinal assessment of parotid gland function using dynamic quantitative pertechnate scintigraphy and estimation of dose-response relationship of parotid-sparing radiotherapy in head-neck cancers. *Radiat Oncol* **2015**, *10*, 67, doi:10.1186/s13014-015-0371-2.
394. Lee, N.R.; Shin, H.B.; Kim, H.I.; Choi, M.S.; Inn, K.S. Negative regulation of RIG-I-mediated antiviral signaling by TRK-fused gene (TFG) protein. *Biochem Biophys Res Commun* **2013**, *437*, 168-172, doi:10.1016/j.bbrc.2013.06.061.
395. Sokabe, T.; Fukumi-Tominaga, T.; Yonemura, S.; Mizuno, A.; Tominaga, M. The TRPV4 channel contributes to intercellular junction formation in keratinocytes. *J Biol Chem* **2010**, *285*, 18749-18758, doi:10.1074/jbc.M110.103606.
396. Xu, H.; Zhao, H.; Tian, W.; Yoshida, K.; Roullet, J.B.; Cohen, D.M. Regulation of a transient receptor potential (TRP) channel by tyrosine phosphorylation. SRC family kinase-dependent tyrosine phosphorylation of TRPV4 on TYR-253 mediates its response to hypotonic stress. *J Biol Chem* **2003**, *278*, 11520-11527, doi:10.1074/jbc.M211061200.
397. Takaki, E.; Fujimoto, M.; Nakahari, T.; Yonemura, S.; Miyata, Y.; Hayashida, N.; Yamamoto, K.; Vallee, R.B.; Mikuriya, T.; Sugahara, K.; et al. Heat shock transcription factor 1 is required for

maintenance of ciliary beating in mice. *J Biol Chem* **2007**, *282*, 37285-37292, doi:10.1074/jbc.M704562200.

398. Fontaine, S.N.; Rauch, J.N.; Nordhues, B.A.; Assimon, V.A.; Stothert, A.R.; Jinwal, U.K.; Sabbagh, J.J.; Chang, L.; Stevens, S.M., Jr.; Zuiderweg, E.R.; et al. Isoform-selective Genetic Inhibition of Constitutive Cytosolic Hsp70 Activity Promotes Client Tau Degradation Using an Altered Co-chaperone Complement. *J Biol Chem* **2015**, *290*, 13115-13127, doi:10.1074/jbc.M115.637595.

399. Ebmeier, C.C.; Taatjes, D.J. Activator-Mediator binding regulates Mediator-cofactor interactions. *Proc Natl Acad Sci U S A* **2010**, *107*, 11283-11288, doi:10.1073/pnas.0914215107.

400. Villace, P.; Marion, R.M.; Ortin, J. The composition of Staufen-containing RNA granules from human cells indicates their role in the regulated transport and translation of messenger RNAs. *Nucleic Acids Res* **2004**, *32*, 2411-2420, doi:10.1093/nar/gkh552.

401. Stefani, F.; Zhang, L.; Taylor, S.; Donovan, J.; Rollinson, S.; Doyotte, A.; Brownhill, K.; Bennion, J.; Pickering-Brown, S.; Woodman, P. UBAP1 is a component of an endosome-specific ESCRT-I complex that is essential for MVB sorting. *Curr Biol* **2011**, *21*, 1245-1250, doi:10.1016/j.cub.2011.06.028.

402. Weber, B.; Schaper, C.; Wang, Y.; Scholz, J.; Bein, B. Interaction of the ubiquitin carboxyl terminal esterase L1 with alpha(2)-adrenergic receptors inhibits agonist-mediated p44/42 MAP kinase activation. *Cell Signal* **2009**, *21*, 1513-1521, doi:10.1016/j.cellsig.2009.05.011.

403. Cottrell, B.A.; Galvan, V.; Banwait, S.; Gorostiza, O.; Lombardo, C.R.; Williams, T.; Schilling, B.; Peel, A.; Gibson, B.; Koo, E.H.; et al. A pilot proteomic study of amyloid precursor interactors in Alzheimer's disease. *Ann Neurol* **2005**, *58*, 277-289, doi:10.1002/ana.20554.

404. Kabuta, T.; Mitsui, T.; Takahashi, M.; Fujiwara, Y.; Kabuta, C.; Konya, C.; Tsuchiya, Y.; Hatanaka, Y.; Uchida, K.; Hohjoh, H.; et al. Ubiquitin C-terminal hydrolase L1 (UCH-L1) acts as a novel potentiator of cyclin-dependent kinases to enhance cell proliferation independently of its hydrolase activity. *J Biol Chem* **2013**, *288*, 12615-12626, doi:10.1074/jbc.M112.435701.

405. Caballero, O.L.; Resto, V.; Patturajan, M.; Meerzaman, D.; Guo, M.Z.; Engles, J.; Yochem, R.; Ratovitski, E.; Sidransky, D.; Jen, J. Interaction and colocalization of PGP9.5 with JAB1 and p27(Kip1). *Oncogene* **2002**, *21*, 3003-3010, doi:10.1038/sj.onc.1205390.

406. Bheda, A.; Yue, W.; Gullapalli, A.; Whitehurst, C.; Liu, R.; Pagano, J.S.; Shackelford, J. Positive reciprocal regulation of ubiquitin C-terminal hydrolase L1 and beta-catenin/TCF signaling. *PLoS One* **2009**, *4*, e5955, doi:10.1371/journal.pone.0005955.

407. You, H.; Ge, Y.; Zhang, J.; Cao, Y.; Xing, J.; Su, D.; Huang, Y.; Li, M.; Qu, S.; Sun, F.; et al. Derlin-1 promotes ubiquitylation and degradation of the epithelial Na(+) channel, ENaC. *J Cell Sci* **2017**, *130*, 1027-1036, doi:10.1242/jcs.198242.

408. Deribe, Y.L.; Wild, P.; Chandrashaker, A.; Curak, J.; Schmidt, M.H.H.; Kalaidzidis, Y.; Milutinovic, N.; Kratchmarova, I.; Buerkle, L.; Fetchko, M.J.; et al. Regulation of epidermal growth factor receptor trafficking by lysine deacetylase HDAC6. *Sci Signal* **2009**, *2*, ra84, doi:10.1126/scisignal.2000576.

409. Woodman, B.; Butler, R.; Landles, C.; Lupton, M.K.; Tse, J.; Hockly, E.; Moffitt, H.; Sathasivam, K.; Bates, G.P. The Hdh(Q150/Q150) knock-in mouse model of HD and the R6/2 exon 1 model develop comparable and widespread molecular phenotypes. *Brain Res Bull* **2007**, *72*, 83-97, doi:10.1016/j.brainresbull.2006.11.004.

410. Li, L.; Tao, Q.; Jin, H.; van Hasselt, A.; Poon, F.F.; Wang, X.; Zeng, M.S.; Jia, W.H.; Zeng, Y.X.; Chan, A.T.; et al. The tumor suppressor UCHL1 forms a complex with p53/MDM2/ARF to promote p53 signaling and is frequently silenced in nasopharyngeal carcinoma. *Clin Cancer Res* **2010**, *16*, 2949-2958, doi:10.1158/1078-0432.CCR-09-3178.

411. Wobst, H.; Forster, S.; Laurini, C.; Sekulla, A.; Dreiseidler, M.; Hohfeld, J.; Schmitz, B.; Diestel, S. UCHL1 regulates ubiquitination and recycling of the neural cell adhesion molecule NCAM. *FEBS J* **2012**, *279*, 4398-4409, doi:10.1111/febs.12029.
412. Nagamine, S.; Kabuta, T.; Furuta, A.; Yamamoto, K.; Takahashi, A.; Wada, K. Deficiency of ubiquitin carboxy-terminal hydrolase-L1 (UCH-L1) leads to vulnerability to lipid peroxidation. *Neurochem Int* **2010**, *57*, 102-110, doi:10.1016/j.neuint.2010.04.015.
413. McKeon, J.E.; Sha, D.; Li, L.; Chin, L.S. Parkin-mediated K63-polyubiquitination targets ubiquitin C-terminal hydrolase L1 for degradation by the autophagy-lysosome system. *Cell Mol Life Sci* **2015**, *72*, 1811-1824, doi:10.1007/s00018-014-1781-2.
414. Liu, Y.; Fallon, L.; Lashuel, H.A.; Liu, Z.; Lansbury, P.T., Jr. The UCH-L1 gene encodes two opposing enzymatic activities that affect alpha-synuclein degradation and Parkinson's disease susceptibility. *Cell* **2002**, *111*, 209-218.
415. Ilic, A.; Lu, S.; Bhatia, V.; Begum, F.; Klonisch, T.; Agarwal, P.; Xu, W.; Davie, J.R. Ubiquitin C-terminal hydrolase isozyme L1 is associated with shelterin complex at interstitial telomeric sites. *Epigenetics Chromatin* **2017**, *10*, 54, doi:10.1186/s13072-017-0160-2.
416. Fritsch, J.; Stephan, M.; Tchikov, V.; Winoto-Morbach, S.; Gubkina, S.; Kabelitz, D.; Schutze, S. Cell fate decisions regulated by K63 ubiquitination of tumor necrosis factor receptor 1. *Mol Cell Biol* **2014**, *34*, 3214-3228, doi:10.1128/MCB.00048-14.
417. Loch, C.M.; Strickler, J.E. A microarray of ubiquitylated proteins for profiling deubiquitylase activity reveals the critical roles of both chain and substrate. *Biochim Biophys Acta* **2012**, *1823*, 2069-2078, doi:10.1016/j.bbamcr.2012.05.006.
418. Osaka, H.; Wang, Y.L.; Takada, K.; Takizawa, S.; Setsuie, R.; Li, H.; Sato, Y.; Nishikawa, K.; Sun, Y.J.; Sakurai, M.; et al. Ubiquitin carboxy-terminal hydrolase L1 binds to and stabilizes monoubiquitin in neuron. *Hum Mol Genet* **2003**, *12*, 1945-1958.
419. Seigneurin-Berny, D.; Verdel, A.; Curtet, S.; Lemercier, C.; Garin, J.; Rousseaux, S.; Khochbin, S. Identification of components of the murine histone deacetylase 6 complex: Link between acetylation and ubiquitination signaling pathways. *Molecular and Cellular Biology* **2001**, *21*, 8035-8044, doi:Doi 10.1128/Mcb.21.23.8035-8044.2001.
420. Cao, Z.; Wu, X.; Yen, L.; Sweeney, C.; Carraway, K.L., 3rd. Neuregulin-induced ErbB3 downregulation is mediated by a protein stability cascade involving the E3 ubiquitin ligase Nrdp1. *Mol Cell Biol* **2007**, *27*, 2180-2188, doi:10.1128/MCB.01245-06.
421. Row, P.E.; Liu, H.; Hayes, S.; Welchman, R.; Charalabous, P.; Hofmann, K.; Clague, M.J.; Sanderson, C.M.; Urbe, S. The MIT domain of UBPY constitutes a CHMP binding and endosomal localization signal required for efficient epidermal growth factor receptor degradation. *J Biol Chem* **2007**, *282*, 30929-30937, doi:10.1074/jbc.M704009200.
422. Rigden, D.J.; Liu, H.; Hayes, S.D.; Urbe, S.; Clague, M.J. Ab initio protein modelling reveals novel human MIT domains. *FEBS Lett* **2009**, *583*, 872-878, doi:10.1016/j.febslet.2009.02.012.
423. Mizuno, E.; Kitamura, N.; Komada, M. 14-3-3-dependent inhibition of the deubiquitinating activity of UBPY and its cancellation in the M phase. *Exp Cell Res* **2007**, *313*, 3624-3634, doi:10.1016/j.yexcr.2007.07.028.
424. Kato, M.; Miyazawa, K.; Kitamura, N. A deubiquitinating enzyme UBPY interacts with the Src homology 3 domain of Hrs-binding protein via a novel binding motif PX(V/I)(D/N)RXXKP. *J Biol Chem* **2000**, *275*, 37481-37487, doi:10.1074/jbc.M007251200.
425. Troilo, A.; Alexander, I.; Muehl, S.; Jaramillo, D.; Knobeloch, K.P.; Krek, W. HIF1alpha deubiquitination by USP8 is essential for ciliogenesis in normoxia. *EMBO Rep* **2014**, *15*, 77-85, doi:10.1002/embr.201337688.
426. Sowa, M.E.; Bennett, E.J.; Gygi, S.P.; Harper, J.W. Defining the human deubiquitinating enzyme interaction landscape. *Cell* **2009**, *138*, 389-403, doi:10.1016/j.cell.2009.04.042.

427. Ge, C.; Che, L.; Ren, J.; Pandita, R.K.; Lu, J.; Li, K.; Pandita, T.K.; Du, C. BRUCE regulates DNA double-strand break response by promoting USP8 deubiquitination of BRIT1. *Proc Natl Acad Sci U S A* **2015**, *112*, E1210-1219, doi:10.1073/pnas.1418335112.
428. Zhang, J.; Zhang, P.; Wei, Y.; Piao, H.L.; Wang, W.; Maddika, S.; Wang, M.; Chen, D.; Sun, Y.; Hung, M.C.; et al. Deubiquitylation and stabilization of PTEN by USP13. *Nat Cell Biol* **2013**, *15*, 1486-1494, doi:10.1038/ncb2874.
429. Ali, N.; Zhang, L.; Taylor, S.; Mironov, A.; Urbe, S.; Woodman, P. Recruitment of UBPY and ESCRT exchange drive HD-PTP-dependent sorting of EGFR to the MVB. *Curr Biol* **2013**, *23*, 453-461, doi:10.1016/j.cub.2013.02.033.
430. Alexopoulou, Z.; Lang, J.; Perrett, R.M.; Elschami, M.; Hurry, M.E.; Kim, H.T.; Mazaraki, D.; Szabo, A.; Kessler, B.M.; Goldberg, A.L.; et al. Deubiquitinase Usp8 regulates alpha-synuclein clearance and modifies its toxicity in Lewy body disease. *Proc Natl Acad Sci U S A* **2016**, *113*, E4688-4697, doi:10.1073/pnas.1523597113.
431. Hans, F.; Fiesel, F.C.; Strong, J.C.; Jackel, S.; Rasse, T.M.; Geisler, S.; Springer, W.; Schulz, J.B.; Voigt, A.; Kahle, P.J. UBE2E ubiquitin-conjugating enzymes and ubiquitin isopeptidase Y regulate TDP-43 protein ubiquitination. *J Biol Chem* **2014**, *289*, 19164-19179, doi:10.1074/jbc.M114.561704.
432. Klein, J.B.; Barati, M.T.; Wu, R.; Gozal, D.; Sachleben, L.R., Jr.; Kausar, H.; Trent, J.O.; Gozal, E.; Rane, M.J. Akt-mediated valosin-containing protein 97 phosphorylation regulates its association with ubiquitinated proteins. *J Biol Chem* **2005**, *280*, 31870-31881, doi:10.1074/jbc.M501802200.
433. Lee, J.N.; Zhang, X.; Feramisco, J.D.; Gong, Y.; Ye, J. Unsaturated fatty acids inhibit proteasomal degradation of Insig-1 at a postubiquitination step. *J Biol Chem* **2008**, *283*, 33772-33783, doi:10.1074/jbc.M806108200.
434. Rutledge, A.C.; Qiu, W.; Zhang, R.; Kohen-Avramoglu, R.; Nemat-Gorgani, N.; Adeli, K. Mechanisms targeting apolipoprotein B100 to proteasomal degradation: evidence that degradation is initiated by BiP binding at the N terminus and the formation of a p97 complex at the C terminus. *Arterioscler Thromb Vasc Biol* **2009**, *29*, 579-585, doi:10.1161/ATVBAHA.108.181859.
435. Fujita, K.; Nakamura, Y.; Oka, T.; Ito, H.; Tamura, T.; Tagawa, K.; Sasabe, T.; Katsuta, A.; Motoki, K.; Shiwaku, H.; et al. A functional deficiency of TERA/VCP/p97 contributes to impaired DNA repair in multiple polyglutamine diseases. *Nat Commun* **2013**, *4*, 1816, doi:10.1038/ncomms2828.
436. Zhang, Z.; Wang, Y.; Li, C.; Shi, Z.; Hao, Q.; Wang, W.; Song, X.; Zhao, Y.; Jiao, S.; Zhou, Z. The Transitional Endoplasmic Reticulum ATPase p97 Regulates the Alternative Nuclear Factor NF-kappaB Signaling via Partial Degradation of the NF-kappaB Subunit p100. *J Biol Chem* **2015**, *290*, 19558-19568, doi:10.1074/jbc.M114.630061.
437. Magadan, J.G.; Bonifacino, J.S. Transmembrane domain determinants of CD4 Downregulation by HIV-1 Vpu. *J Virol* **2012**, *86*, 757-772, doi:10.1128/JVI.05933-11.
438. Grebien, F.; Vedadi, M.; Getlik, M.; Giambruno, R.; Grover, A.; Avellino, R.; Skucha, A.; Vittori, S.; Kuznetsova, E.; Smil, D.; et al. Pharmacological targeting of the Wdr5-MLL interaction in C/EBPalpha N-terminal leukemia. *Nat Chem Biol* **2015**, *11*, 571-578, doi:10.1038/nchembio.1859.
439. Ballar, P.; Ors, A.U.; Yang, H.; Fang, S. Differential regulation of CFTRDeltaF508 degradation by ubiquitin ligases gp78 and Hrd1. *Int J Biochem Cell Biol* **2010**, *42*, 167-173, doi:10.1016/j.biocel.2009.10.005.
440. Pleasure, I.T.; Black, M.M.; Keen, J.H. Valosin-containing protein, VCP, is a ubiquitous clathrin-binding protein. *Nature* **1993**, *365*, 459-462, doi:10.1038/365459a0.
441. Fernandez-Arenas, E.; Calleja, E.; Martinez-Martin, N.; Gharbi, S.I.; Navajas, R.; Garcia-Medel, N.; Penela, P.; Alcami, A.; Mayor, F., Jr.; Albar, J.P.; et al. beta-Arrestin-1 mediates the TCR-triggered re-routing of distal receptors to the immunological synapse by a PKC-mediated mechanism. *EMBO J* **2014**, *33*, 559-577, doi:10.1002/embj.201386022.

442. den Besten, W.; Verma, R.; Kleiger, G.; Oania, R.S.; Deshaies, R.J. NEDD8 links cullin-RING ubiquitin ligase function to the p97 pathway. *Nat Struct Mol Biol* **2012**, *19*, 511-516, S511, doi:10.1038/nsmb.2269.
443. Huang, D.; Qiu, F.; Yang, L.; Li, Y.; Cheng, M.; Wang, H.; Ma, G.; Wang, Y.; Hu, M.; Ji, W.; et al. The polymorphisms and haplotypes of WWOX gene are associated with the risk of lung cancer in southern and eastern Chinese populations. *Mol Carcinog* **2013**, *52 Suppl 1*, E19-27, doi:10.1002/mc.21934.
444. Madsen, L.; Molbaek, K.; Larsen, I.B.; Nielsen, S.V.; Poulsen, E.G.; Walmod, P.S.; Hofmann, K.; Seeger, M.; Chien, C.Y.; Chen, R.H.; et al. Human ASPL/TUG interacts with p97 and complements the proteasome mislocalization of a yeast ubx4 mutant, but not the ER-associated degradation defect. *BMC Cell Biol* **2014**, *15*, 31, doi:10.1186/1471-2121-15-31.
445. Yoshida, Y.; Adachi, E.; Fukiya, K.; Iwai, K.; Tanaka, K. Glycoprotein-specific ubiquitin ligases recognize N-glycans in unfolded substrates. *EMBO Rep* **2005**, *6*, 239-244, doi:10.1038/sj.embor.7400351.
446. Li, J.M.; Wu, H.; Zhang, W.; Blackburn, M.R.; Jin, J. The p97-UFD1L-NPL4 protein complex mediates cytokine-induced IkappaBalpha proteolysis. *Mol Cell Biol* **2014**, *34*, 335-347, doi:10.1128/MCB.01190-13.
447. Rudra, D.; deRoos, P.; Chaudhry, A.; Niec, R.E.; Arvey, A.; Samstein, R.M.; Leslie, C.; Shaffer, S.A.; Goodlett, D.R.; Rudensky, A.Y. Transcription factor Foxp3 and its protein partners form a complex regulatory network. *Nat Immunol* **2012**, *13*, 1010-1019, doi:10.1038/ni.2402.
448. Grossmann, A.; Benlasfer, N.; Birth, P.; Hegele, A.; Wachsmuth, F.; Apelt, L.; Stelzl, U. Phospho-tyrosine dependent protein-protein interaction network. *Mol Syst Biol* **2015**, *11*, 794, doi:10.15252/msb.20145968.
449. Alexandru, G.; Graumann, J.; Smith, G.T.; Kolawa, N.J.; Fang, R.; Deshaies, R.J. UBXD7 binds multiple ubiquitin ligases and implicates p97 in HIF1alpha turnover. *Cell* **2008**, *134*, 804-816, doi:10.1016/j.cell.2008.06.048.
450. Soetandyo, N.; Ye, Y. The p97 ATPase dislocates MHC class I heavy chain in US2-expressing cells via a Ufd1-Npl4-independent mechanism. *J Biol Chem* **2010**, *285*, 32352-32359, doi:10.1074/jbc.M110.131649.
451. Yu, M.; Wang, J.; Li, W.; Yuan, Y.Z.; Li, C.Y.; Qian, X.H.; Xu, W.X.; Zhan, Y.Q.; Yang, X.M. Proteomic screen defines the hepatocyte nuclear factor 1alpha-binding partners and identifies HMGB1 as a new cofactor of HNF1alpha. *Nucleic Acids Res* **2008**, *36*, 1209-1219, doi:10.1093/nar/gkm1131.
452. Rao, R.; Balusu, R.; Fiskus, W.; Mudunuru, U.; Venkannagari, S.; Chauhan, L.; Smith, J.E.; Hembruff, S.L.; Ha, K.; Atadja, P.; et al. Combination of pan-histone deacetylase inhibitor and autophagy inhibitor exerts superior efficacy against triple-negative human breast cancer cells. *Mol Cancer Ther* **2012**, *11*, 973-983, doi:10.1158/1535-7163.MCT-11-0979.
453. Ikeda, Y.; Demartino, G.N.; Brown, M.S.; Lee, J.N.; Goldstein, J.L.; Ye, J. Regulated endoplasmic reticulum-associated degradation of a polytopic protein: p97 recruits proteasomes to Insig-1 before extraction from membranes. *J Biol Chem* **2009**, *284*, 34889-34900, doi:10.1074/jbc.M109.044875.
454. Min, T.; Bodas, M.; Mazur, S.; Vij, N. Critical role of proteostasis-imbalance in pathogenesis of COPD and severe emphysema. *J Mol Med (Berl)* **2011**, *89*, 577-593, doi:10.1007/s00109-011-0732-8.
455. Kondo, H.; Rabouille, C.; Newman, R.; Levine, T.P.; Pappin, D.; Freemont, P.; Warren, G. p47 is a cofactor for p97-mediated membrane fusion. *Nature* **1997**, *388*, 75-78, doi:10.1038/40411.
456. Murakami, K.; Ichinohe, Y.; Koike, M.; Sasaoka, N.; Iemura, S.; Natsume, T.; Kakizuka, A. VCP Is an integral component of a novel feedback mechanism that controls intracellular localization of catalase and H2O2 Levels. *PLoS One* **2013**, *8*, e56012, doi:10.1371/journal.pone.0056012.

457. Chiang, D.Y.; Lebesgue, N.; Beavers, D.L.; Alsina, K.M.; Damen, J.M.; Voigt, N.; Dobrev, D.; Wehrens, X.H.; Scholten, A. Alterations in the interactome of serine/threonine protein phosphatase type-1 in atrial fibrillation patients. *J Am Coll Cardiol* **2015**, *65*, 163-173, doi:10.1016/j.jacc.2014.10.042.
458. Yakovlev, V.A. Nitric oxide-dependent downregulation of BRCA1 expression promotes genetic instability. *Cancer Res* **2013**, *73*, 706-715, doi:10.1158/0008-5472.CAN-12-3270.
459. Al-Hakim, A.K.; Goransson, O.; Deak, M.; Toth, R.; Campbell, D.G.; Morrice, N.A.; Prescott, A.R.; Alessi, D.R. 14-3-3 cooperates with LKB1 to regulate the activity and localization of QSK and SIK. *J Cell Sci* **2005**, *118*, 5661-5673, doi:10.1242/jcs.02670.
460. Inano, S.; Sato, K.; Katsuki, Y.; Kobayashi, W.; Tanaka, H.; Nakajima, K.; Nakada, S.; Miyoshi, H.; Knies, K.; Takaori-Kondo, A.; et al. RFWD3-Mediated Ubiquitination Promotes Timely Removal of Both RPA and RAD51 from DNA Damage Sites to Facilitate Homologous Recombination. *Mol Cell* **2017**, *66*, 622-634 e628, doi:10.1016/j.molcel.2017.04.022.
461. Yen, C.H.; Yang, Y.C.; Ruscetti, S.K.; Kirken, R.A.; Dai, R.M.; Li, C.C. Involvement of the ubiquitin-proteasome pathway in the degradation of nontyrosine kinase-type cytokine receptors of IL-9, IL-2, and erythropoietin. *J Immunol* **2000**, *165*, 6372-6380, doi:10.4049/jimmunol.165.11.6372.
462. Vij, N.; Fang, S.; Zeitlin, P.L. Selective inhibition of endoplasmic reticulum-associated degradation rescues DeltaF508-cystic fibrosis transmembrane regulator and suppresses interleukin-8 levels: therapeutic implications. *J Biol Chem* **2006**, *281*, 17369-17378, doi:10.1074/jbc.M600509200.
463. Schulze, A.; Standera, S.; Buerger, E.; Kikkert, M.; van Voorden, S.; Wiertz, E.; Koning, F.; Kloetzel, P.M.; Seeger, M. The ubiquitin-domain protein HERP forms a complex with components of the endoplasmic reticulum associated degradation pathway. *J Mol Biol* **2005**, *354*, 1021-1027, doi:10.1016/j.jmb.2005.10.020.
464. Gitcho, M.A.; Strider, J.; Carter, D.; Taylor-Reinwald, L.; Forman, M.S.; Goate, A.M.; Cairns, N.J. VCP mutations causing frontotemporal lobar degeneration disrupt localization of TDP-43 and induce cell death. *J Biol Chem* **2009**, *284*, 12384-12398, doi:10.1074/jbc.M900992200.
465. Meyer, H.H.; Wang, Y.; Warren, G. Direct binding of ubiquitin conjugates by the mammalian p97 adaptor complexes, p47 and Ufd1-Npl4. *EMBO J* **2002**, *21*, 5645-5652.
466. Wang, Q.; Liu, Y.; Soetandyo, N.; Baek, K.; Hegde, R.; Ye, Y. A ubiquitin ligase-associated chaperone holdase maintains polypeptides in soluble states for proteasome degradation. *Mol Cell* **2011**, *42*, 758-770, doi:10.1016/j.molcel.2011.05.010.
467. Kobayashi, T.; Tanaka, K.; Inoue, K.; Kakizuka, A. Functional ATPase activity of p97/valosin-containing protein (VCP) is required for the quality control of endoplasmic reticulum in neuronally differentiated mammalian PC12 cells. *J Biol Chem* **2002**, *277*, 47358-47365, doi:10.1074/jbc.M207783200.
468. McClurg, U.L.; Harle, V.J.; Nabbi, A.; Batalha-Pereira, A.; Walker, S.; Coffey, K.; Gaughan, L.; McCracken, S.R.; Robson, C.N. Ubiquitin-specific protease 12 interacting partners Uaf-1 and WDR20 are potential therapeutic targets in prostate cancer. *Oncotarget* **2015**, *6*, 37724-37736, doi:10.18632/oncotarget.6075.
469. Wimuttisuk, W.; West, M.; Davidge, B.; Yu, K.; Salomon, A.; Singer, J.D. Novel Cul3 binding proteins function to remodel E3 ligase complexes. *BMC Cell Biol* **2014**, *15*, 28, doi:10.1186/1471-2121-15-28.
470. Yin, J.; Schoeffler, A.J.; Wickliffe, K.; Newton, K.; Starovasnik, M.A.; Dueber, E.C.; Harris, S.F. Structural Insights into WD-Repeat 48 Activation of Ubiquitin-Specific Protease 46. *Structure* **2015**, *23*, 2043-2054, doi:10.1016/j.str.2015.08.010.
471. Li, X.; Wang, W.; Wang, J.; Malovannaya, A.; Xi, Y.; Li, W.; Guerra, R.; Hawke, D.H.; Qin, J.; Chen, J. Proteomic analyses reveal distinct chromatin-associated and soluble transcription factor complexes. *Mol Syst Biol* **2015**, *11*, 775, doi:10.15252/msb.20145504.

472. Freibaum, B.D.; Chitta, R.K.; High, A.A.; Taylor, J.P. Global analysis of TDP-43 interacting proteins reveals strong association with RNA splicing and translation machinery. *J Proteome Res* **2010**, *9*, 1104-1120, doi:10.1021/pr901076y.
473. Hanein, S.; Martin, E.; Boukhris, A.; Byrne, P.; Goizet, C.; Hamri, A.; Benomar, A.; Lossos, A.; Denora, P.; Fernandez, J.; et al. Identification of the SPG15 gene, encoding spastizin, as a frequent cause of complicated autosomal-recessive spastic paraplegia, including Kjellin syndrome. *Am J Hum Genet* **2008**, *82*, 992-1002, doi:10.1016/j.ajhg.2008.03.004.
474. Shirane, M.; Ogawa, M.; Motoyama, J.; Nakayama, K.I. Regulation of apoptosis and neurite extension by FKBP38 is required for neural tube formation in the mouse. *Genes Cells* **2008**, *13*, 635-651, doi:10.1111/j.1365-2443.2008.01194.x.
475. Raiborg, C.; Wenzel, E.M.; Pedersen, N.M.; Olsvik, H.; Schink, K.O.; Schultz, S.W.; Vietri, M.; Nisi, V.; Bucci, C.; Brech, A.; et al. Repeated ER-endosome contacts promote endosome translocation and neurite outgrowth. *Nature* **2015**, *520*, 234-238, doi:10.1038/nature14359.
